# D-Fructose-based spiro-fused PHOX ligands: synthesis and application in enantioselective allylic alkylation

**DOI:** 10.3762/bjoc.14.182

**Published:** 2018-08-08

**Authors:** Michael R Imrich, Jochen Kraft, Cäcilia Maichle-Mössmer, Thomas Ziegler

**Affiliations:** 1 Institute of Organic Chemistry, University of Tübingen, Auf der Morgenstelle 18, 72076 Tübingen, Germany; 2 Institute of Inorganic Chemistry, University of Tübingen, Auf der Morgenstelle 18, 72076 Tübingen, Germany

**Keywords:** Fürst–Plattner rule, oxazoline, Ritter reaction, Tsuji–Trost reaction, Ullmann coupling

## Abstract

Phosphinooxazoline (PHOX) ligands are an important class of ligands in asymmetric catalysis. We synthesized ten novel D-fructose-derived spiro-fused PHOX ligands with different steric and electronic demand. The application of two of them was tested in asymmetric allylic alkylation. The ligands are prepared in two steps from readily available 1,2-*O*-isopropylidene protected β-D-fructopyranoses by the BF_3_·OEt_2_-promoted Ritter reaction with 2-bromobenzonitrile to construct the oxazoline moiety followed by Ullmann coupling of the resulting aryl bromides with diphenylphosphine. Both steps proceeded mostly in good to high yields (57–86% for the Ritter reaction and 35–89% for the Ullmann coupling). The Ritter reaction gave two anomers, which could be separated by column chromatography. The prepared ligands showed promising results (er of up to 84:16) in Tsuji–Trost reactions with diphenylallyl acetate as model substrate.

## Introduction

**Figure 1:**
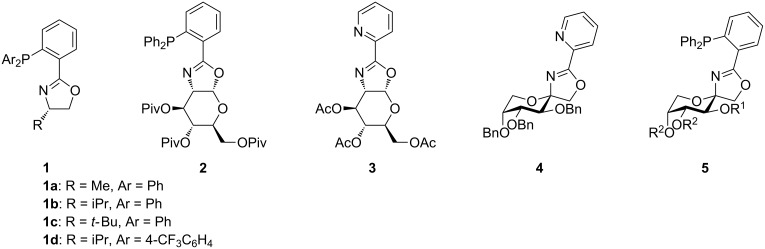
General structure of PHOX ligands **1** and structures of annulated glucosamine-based PHOX and PyOx ligands **2** and **3** and spiro-fused PyOx and PHOX ligands **4** and **5**.

The vast majority of biologically active compounds like vitamins and natural products occur as single enantiomers in nature. Usually only one enantiomer generates the desired biologic effect in living organisms, while the other enantiomer could be inactive, cause whole other biological responses or might even have the opposite effect. Hence, for the total synthesis of natural products or pharmaceuticals it is crucial to generate chirality with high enantioselectivity [1–2]. Probably the most effective approach in stereoselective synthesis is enantioselective catalysis, because cheap prochiral starting materials can be converted into chiral enantiopure products and no undesirable side products are formed [3–4]. Therefore, the development of new ligands is crucial for further progress in stereoselective synthesis [5]. Privileged ligands often used are phosphinooxazoline ligands (PHOX ligands **1**, (Figure 1)) which were developed in 1993 independently by Helmchen, Pfaltz and Williams [6–8]. Palladium– and iridium–PHOX complexes were already applied as efficient catalysts in various asymmetric reactions, for instance allylic substitution and enantioselective hydrogenation [9]. They were also applied in the stereoselective synthesis of complex natural products [10–12]. PHOX ligands are nonsymmetrical ligands which can coordinate to a metal center through their N- and P-moieties. They are usually prepared from amino acids or from the corresponding amino alcohols [9,13]. Some examples of literature-known PHOX ligands are shown in Figure 1 (**1a**–**d**). These ligands gave up to 96% ee by their application in allylic substitution with dimethyl malonate as nucleophile [13–14]. The design of new PHOX ligands is still subject to current research and the synthesis of a lot of different PHOX ligands have been reported during the last years [15–18]. Kunz reported the preparation of a carbohydrate based PHOX ligand **2** [19]. The starting material was D-glucosamine and the sugar was linked to the aromatic system via an annulated oxazoline. Palladium complexes of **2** were used in allylic substitution of allyl acetates with dimethyl malonate as nucleophile and ee values from 69% to 98% were obtained [19]. Recently, we presented the synthesis of carbohydrate pyridyloxazoline (PyOx) ligands in which the sugar moiety was linked to pyridine via an annulated oxazoline, **3**, as well as via a spiro-fused oxazoline, **4**. We found that the spiro-fused ligands gave higher enantioselectivities (up to 93% ee) than the annulated ligands (up to 66% ee) in allylic substitution [20–22]. This led us to extend the concept of spiro-fused carbohydrate oxazolines for asymmetric synthesis by developing new types of carbohydrate-based PHOX ligands. Herein, we present ten novel spiro-PHOX ligands containing diphenylphosphino groups, **5**, which can be synthesized in four to six steps starting from D-fructose. Two of these ligands were applied in enantioselective catalysis.

## Results and Discussion

**Scheme 1:**
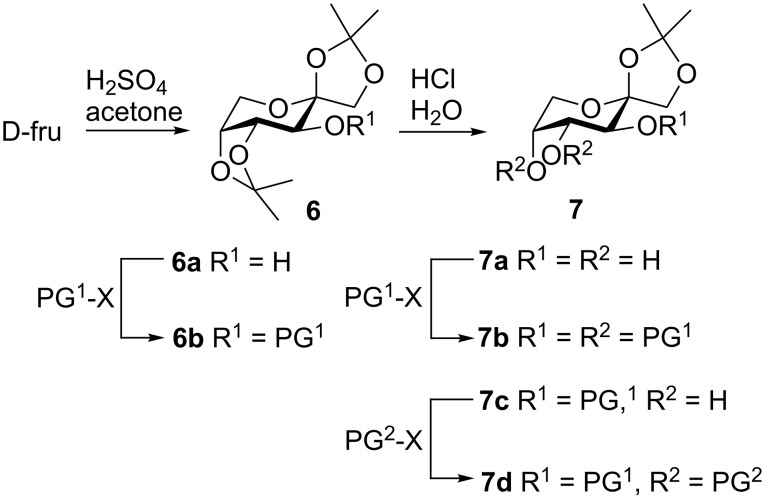
Preparation of 1,2-isopropylidene-protected D-fructose derivatives with different substitution pattern at positions 3, 4 and 5. PG: protective group, X: leaving group.

Starting from D-fructose, 1,2-isopropylidene-protected pyranosides with different protective groups (PG) at C-3, C-4 and C-5 can be prepared in two to four steps (Scheme 1). First, D-fructose was converted to **6a** as previously described in [23]. Next, the isopropylidene group at positions 4 and 5 were removed under acidic conditions and the resulting intermediate **7a** was converted to **7b** with identical protecting groups at positions 3, 4 and 5 [24–28]. In order to obtain D-fructose derivatives with different protective groups at position 3, 4 and 5 the hydroxy group of **6a** was first protected to afford **6b**. After removing the isopropylidene group, positions 4 and 5 of the resulting diol **7c** were protected to afford **7d**.

A convenient method for constructing anomeric 2-oxazolines is the Ritter reaction of suitable carbohydrate derivatives with nitriles under Lewis-acidic conditions [29–31]. Recently, Vangala and Shinde reported the synthesis of spirocyclic 2-substituted 2-oxazoline ribosides from 1,2-isopropylidene-protected furanosides [32]. In our case, however, only small yields were obtained by the application of the Vangala protocol (activation of the carbohydrate with TMSOTf in toluene and different nitriles as nucleophiles; see Supporting Information File 1 for details). With slight modifications, however, (BF_3_·OEt_2_ as Lewis acid instead of TMSOTf and CH_2_Cl_2_ as solvent) the reaction proceeded smoothly in good to high yields. We chose 2-bromobenzonitrile (**8**) as a nucleophile because we planned to modify the aryl bromide by transition metal-catalyzed cross-coupling reactions afterwards. Unfortunately, the nitrile must be used in a high excess of 15 equiv because the yield decreases heavily otherwise. Up to 14 equiv of **8** can be re-isolated after the reaction though. We applied the modified Ritter reaction to nine different 1,2-isopropylidene-proctected fructose derivatives as depicted in Table 1.

**Table 1:** Synthesis of spiro-fused oxazolines **10** and **11** via Ritter reaction.

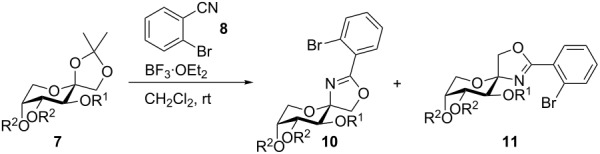								

entry	**7**	R^1^	R^2^	equiv BF_3_	time	**10**, %^a^	**11**, %^a^	β:α

1	**7e**	Bn	C(CH_3_)_2_	1.0	40 min	**10a**, 58	**11a**, –^b^	100:0
2	**7f**	Bn	Bn	1.0	35 min	**10b**, 69	**11b**, 5	93:7
3	**7g**	Bn	CH_3_	1.0	35 min	**10c**, 62	**11c**, 9	87:13
4	**7h**	CH_3_	Bn	1.0	60 min	**10d**, 74	**11d**, 12	86:14
5	**7i**	CH_3_	CH_3_	1.0	75 min	**10e**, 66	**11e**, 20	77:23
6	**7j**	Bz	Bz	4.0	4 d	**10f**, 45	**11f**, 16	74:26
7	**7k**	Piv	Piv	4.0	3 d	**10g**, 56	**11g**, 22	72:28
8	**7l**	Ac	C(CH_3_)_2_	1.5	8 h	**10h**, 42	**11h**, 18	70:30
9	**7m**	Ac	Ac	3.0	4 d	**10i**, 50	**11i**, 26	66:34

^a^Isolated yield, ^b^not detected.

**Scheme 2:**
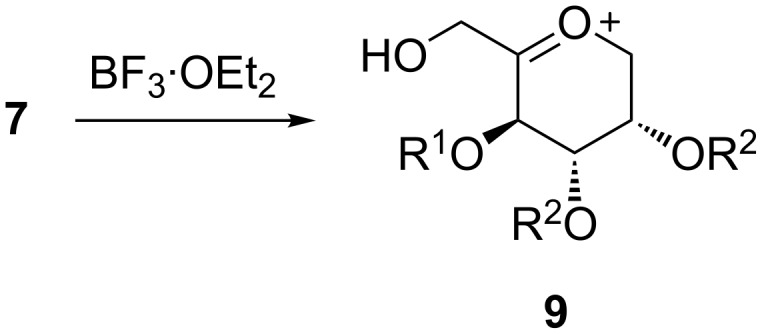
Activation of **7** to oxocarbenium ion **9** in the Ritter reaction.

With ether protective groups (Table 1, entries 1–5), the reaction proceeded in about an hour or faster and only one equiv BF_3_·OEt_2_ had to be added. When the fructose derivative was protected with ester groups (Table 1, entries 6–9), the reaction was significantly slower (up to 4 days reaction time) and higher amounts of BF_3_·OEt_2_ had to be added. This observation can be explained as follows. In the first step, the 1,2-isopropylidene group is cleaved by the Lewis acid and an oxocarbenium ion (**9**, Scheme 2) is generated [30,32]. With electron-withdrawing groups like acetyl, benzoyl or pivaloyl the carbohydrate gets more electron deficient and the generation of **9** is hindered. In the literature this fact is used to explain the different reactivities between “armed” and “disarmed” glycosyl donors in glycosylation reactions [33].

**Scheme 3:**
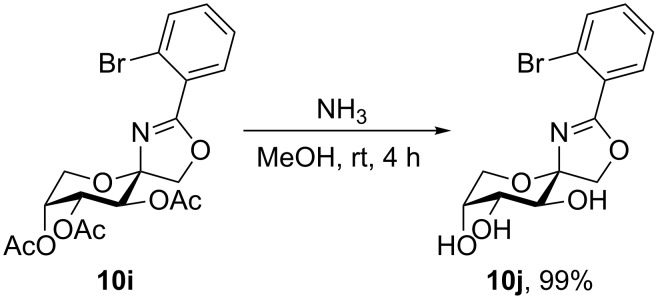
Zemplén deacetylation of **10i**.

Due to the fact that **9** can be attacked from two sides by nitriles, the oxazolines occur in two isomeric forms, the β-anomers (**10**) and the α-anomers (**11**), which were separated by column chromatography. No crystals suitable for X-ray crystallography could be obtained from the direct products of the Ritter reaction. To get a more polar molecule which is more appropriate to form crystals suitable for X-ray crystallography, a deprotected derivative of **10** was prepared. The acetyl groups of **10i** can easily be removed by Zemplén deacetylation (Scheme 3) [34]. Instead of the classical protocol with sodium methoxide, ammonia in methanol was applied, because oxazolines are sensitive to acid and with ammonia no acid has to be added to neutralize the reaction mixture [35]. The deprotected oxazoline **10j** was isolated in nearly quantitative yield.

**Figure 2:**
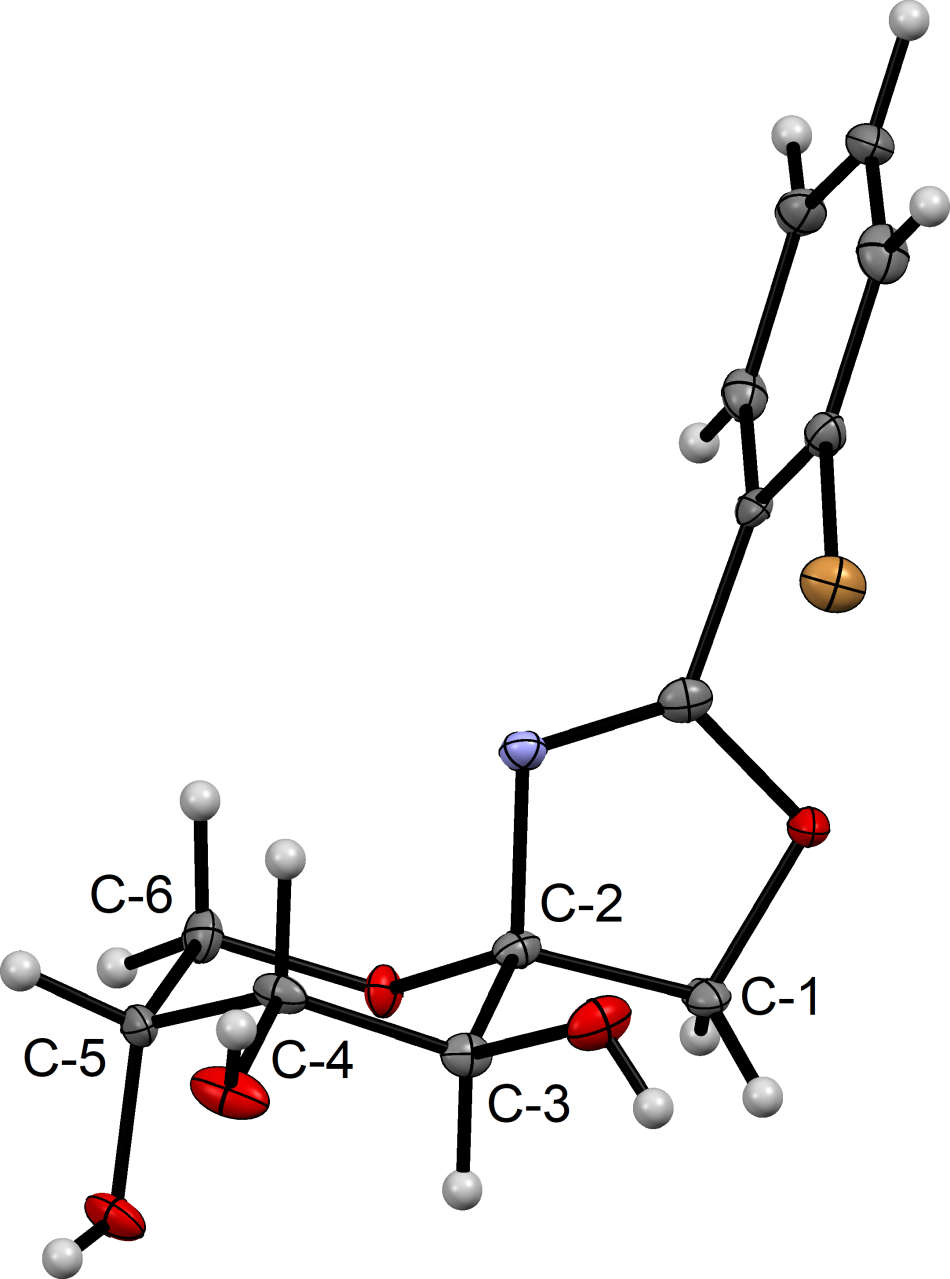
Molecular structure of **10j**. Ellipsoids are given at the 50% probability level. Grey = carbon, red = oxygen, white = hydrogen, purple = nitrogen, orange = bromine.

**Scheme 4:**
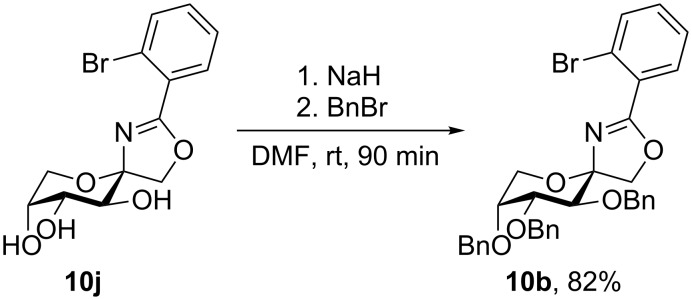
Benzylation of **10j** to give **10b**.

By covering a saturated solution of **10j** in 2-propanol with *n*-heptane crystals suitable for X-ray crystallography were thus obtained. The compound crystallizes in the orthorhombic space group *P*2_1_2_1_2_1_. The molecular structure is shown in Figure 2, detailed crystal data and structure refinements of the X-ray analysis are given in Supporting Information File 1. The configuration at the anomeric center is β and the fructose ring adopts _5_*C*^2^ conformation. To confirm that the configuration of the major product of the Ritter reaction with ether-protected carbohydrates is β as well, **10j** was benzylated and **10b** was obtained (Scheme 4). This proves the structure of **10b** as well.

**Scheme 5:**
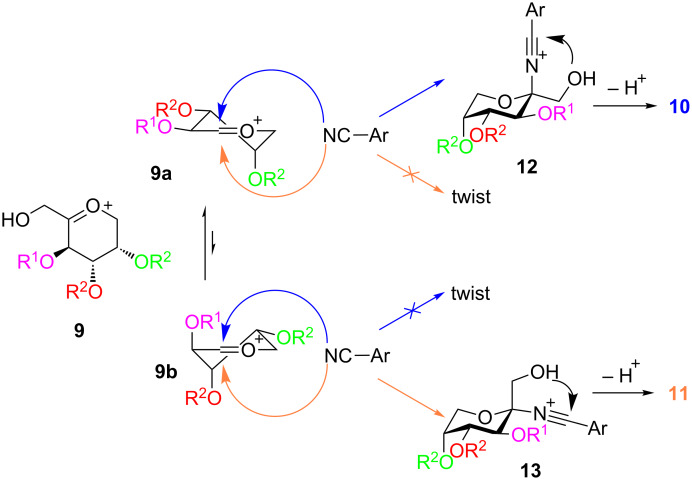
Plausible mechanism of the Ritter reaction. For better clarity C-2 is not shown in conformers **9a** and **9b**.

The ratio of the anomers produced by the Ritter reaction depends on the substitution pattern of the pyranoside used and varies from only β and 93:7 β:α, respectively (Table 1, entries 1 and 2) to a β:α ratio of 66:34 (Table 1, entry 9). These ratios can be explained by the mechanism of the Ritter reaction. The oxocarbenium ion **9** exists as an equilibrium of two conformer half-chair forms **9a** and **9b** (Scheme 5). Theoretical investigations of substituted cyclic oxocarbenium ions showed for oxocarbenium ions with electronegative substituents that two positions from the ring oxygen the conformer with this substituent in an axial position is favored. This can be explained by a through space electrostatic interaction of the partially negatively charged substituent and the positively charged ring. With the axial substituent these charges are closer together and the conformer is preferred [36–38]. By application of these assumptions to our system, we suggest that **9a** should be the main conformer. A second argument for the dominance of **9a** is that it has two equatorial substituents whereas **9b** has only one substituent in equatorial position. The sterical demand of R^1^ has a special influence on the equilibrium because in **9a** R^1^ has an equatorial position, whereas in **9b** R^1^ is axial. This means that for bulky R^1^ substituents the equilibrium will be further forced towards **9a**. Since in both conformers one R^2^ is axial and the other is equatorial the bulkiness of R^2^ should have no influence on the equilibrium. Both conformers can be attacked by **8** from two different sides, where each one leads to an addition product in chair conformation or to a product in twist conformation. Due to the fact that the lower energy level of the chair conformation compared to the twist conformation is already present in the transition state the generation of the twist conformer is kinetically disfavored [39–40]. This kinetic phenomenon is sometimes called the Fürst–Plattner rule [41–42]. Intramolecular cyclization of intermediates **12** and **13** creates the oxazolines **10** and **11**. With this model at hand the β:α ratios of entries 1 to 5 in Table 1 can be explained. For R^1^ = Bn the equilibrium between **9a** and **9b** is positioned far in favor to **9a**. Nucleophilic attack of the nitrile according to the Fürst–Plattner rule provides **10** in a high excess (Table 1, entries 1–3). With the smaller substituent R^1^ = CH_3_ conformer **9b** gets more important and more **11** is produced (Table 1, entries 4 and 5). By comparing entries 2 and 3 as well as 4 and 5 we noticed that the size of R^2^ has an influence on the ratio too. This could be explained by steric hindrance of R^2^ at the nucleophilic attack at **9b**.

**Scheme 6:**
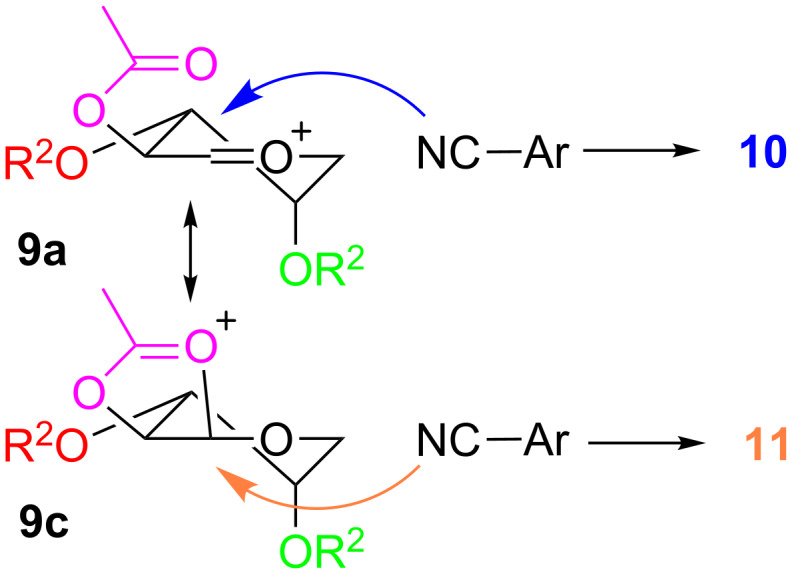
Neighboring group participation of ester protective groups. For better clarity C-2 is not shown in conformers **9a** and **9c**.

However, the described model does not work for entries 6–9 in Table 1, because we observed significant amounts of **11** even with very bulky protective groups like benzoyl or pivaloyl. In carbohydrate chemistry a well-known phenomenon is participation of neighboring groups. An oxocarbenium ion is often stabilized by protective groups. Esters are a class of protective groups which often participate in such a manner [43–44]. **7j**–**m** bear at least one ester protective group. We propose that when R^1^ is such a substituent the oxocarbenium ion **9a** is stabilized as indicated in Scheme 6. In the stabilized species **9c** the formerly favored nucleophilic attack from the top is blocked so that the addition of the nitrile has to occur from the bottom and higher amounts of **11** are generated. Neighboring group participation in conformer **9b** leads to the same anomer as predicted by the Fürst–Plattner rule.

Neighboring group participation provides an alternative explanation for the already mentioned longer reaction times for the acylated sugars **7j**–**m**. Because of the high delocalization of the positive charge in **9c** this oxocarbenium ion is more stable compared to **9b**. Cyclic oxocarbenium ions of the type of **9c** are known to be stable intermediates which can even be isolated in the absence of nucleophiles [45–47]. Due to the higher stability of the cationic intermediate the nucleophilic attack of the nitrile is slower compared to the unstabilized **9b**.

The Stoltz and co-workers reported the preparation of a series of PHOX ligands using Buchwald’s copper-catalyzed C–P bond construction [48–49]. This reaction allows the Ullmann coupling of aryl halides with secondary phosphines to afford tertiary phosphines. We chose Stoltz’s protocol to introduce the diphenylphosphine moiety to our β-configurated bromoaryloxazolines **10a**–**i** as well as to oxazoline **11i** due to the fact that the peracetylated sugar could be obtained as an α-anomer in relatively high yield. The reaction afforded the spiro-fused PHOX ligands in fair to good yields. For ether-protected substances yields ranged from 67% to 89% (Table 2, entries 1–5). Coupling of aryl bromides with ester-protected substances gave also acceptable yields in a range from 35% to 69% (Table 2, entries 6–10), they were considerably lower. It is known that phosphines can be used as nucleophiles for deacetylation reactions [50–51]. We suppose that the lower yields of **5f**–**i** and **14a** can be explained by partial or full deacylation of the protective groups. This explanation is also in good accordance with the fact that the yield of the ester protected PHOX ligand strongly decreased with longer reaction times (see Supporting Information File 1 for details).

**Table 2:** Ullmann reaction of bromoaryloxazolines with diphenylphosphine.

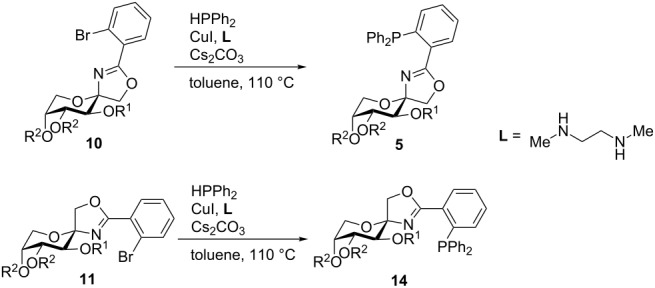						

entry	reactant	R^1^	R^2^	anomer	time	product, %^a^

1	**10a**	Bn	C(CH_3_)_2_	β	18 h	**5a**, 67
2	**10b**	Bn	Bn	β	21 h	**5b**, 89
3	**10c**	Bn	CH_3_	β	16 h	**5c**, 83
4	**10d**	CH_3_	Bn	β	16 h	**5d**, 80
5	**10e**	CH_3_	CH_3_	β	14 h	**5e**, 81
6	**10f**	Bz	Bz	β	8 h	**5f**, 69
7	**10g**	Piv	Piv	β	4 h	**5g**, 66
8	**10h**	Ac	C(CH_3_)_2_	β	7 h	**5h**, 60
9	**10i**	Ac	Ac	β	7 h	**5i**, 65
10	**11i**	Ac	Ac	α	8 h	**14a**, 35

^a^Isolated yield.

**Scheme 7:**
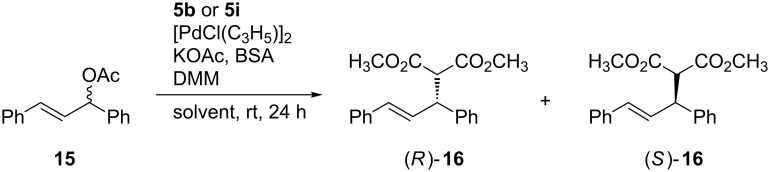
Pd catalyzed Tsuji–Trost reation. BSA: *N*,*O*-bis(trimethylsilyl)acetamide, DMM: dimethyl malonate.

With the fructose-based spiro-fused PHOX ligands in hand, we turned to some preliminary tests in order to evaluate the usefulness of our ligands in asymmetric catalysis. We chose to test one ligand with ether protective groups (**5b**) and one with ester groups (**5i**). As a model system for the Pd-catalyzed Tsuji–Trost reaction we chose diphenylallyl acetate **15** (Scheme 7) with dimethyl malonate. The latter allylic alkylation is well investigated and has often been used as a benchmark test for the selectivity of novel ligands like Kunz’ PHOX ligand **2** or our PyOx ligands **3** and **4** [14,19–22,52–53]. Palladium complexes of both ligands **5b** and **5i** were suitable to catalyze the allylic substitution in an enantioselective manner (Table 3). Conversions were quantitative or at least high with both ligands in a number of tested solvents. Promising enantiomeric ratios ranging from 76:24 to 84:16 were obtained. For the tested solvents it seems that the solvent just has a small influence on the enantioselectivity. Interestingly, in diethyl ether **5b** showed the smallest excess of (*R*)-**16** (entry 3, Table 3) whereas **5i** lead to the highest er in our preliminary tests. The reason for this will to be studied in further investigations, as well as the application of other spiro-fused PHOX ligands in asymmetric catalysis.

**Table 3:** Pd-catalyzed Tsuji–Trost alkylation using ligands **5b** and **5i**.

entry	ligand	solvent	conversion^a^	er^b^ (*R*:*S*)

1	**5b**	CH_2_Cl_2_	>99% (98%)^c^	82:18
2	**5b**	toluene	>99%	82:18
3	**5b**	Et_2_O	>99%	76:24
4	**5b**	MeCN	>99%	82:18
5	**5i**	CH_2_Cl_2_	>99%	77:23
6	**5i**	toluene	>99%	78:22
7	**5i**	Et_2_O	83% (80%)^c^	84:16
8	**5i**	MeCN	>99%	77:23

^a^Determined by ^1^H NMR spectroscopy; ^b^enantiomeric ratio measured by chiral HPLC, absolute configuration was assigned by comparison of optical rotation values with literature data [54]; ^c^isolated yield.

## Conclusion

In conclusion, we developed a short and efficient synthesis for D-fructose-based spiro-fused PHOX ligands. The described ligands can be prepared from literature-known carbohydrate derivatives in two steps. Preliminary tests of the spiro-fused PHOX ligands in Tsuji–Trost reaction showed promising results. Different metal complexes as well as further application of the ligands in asymmetric catalysis are currently under investigation. This will hopefully provide insight into the mechanism of the Tsuji–Trost reaction with our ligands which will lead to further improvement of our spiro-PHOX ligands.

## Supporting Information

CCDC 1831148 contains the supplementary crystallographic data for **10j**. These data can be obtained free of charge from The Cambridge Crystallographic Data Centre via http://www.ccdc.cam.ac.uk/data_request/cif.

File 1 Experimental procedures, additional experiments, copies of ^1^H, ^13^C{^1^H} and ^31^P NMR of all new compounds, crystallographic data and copies of HPLC chromatograms.
