# Supplementary material for: D-Fructose-based spiro-fused PHOX ligands: synthesis and application in enantioselective allylic alkylation
Source: Beilstein J Org Chem. 2018 Aug 8;14:2082–9. doi: 10.3762/bjoc.14.182 (PMC6122323; doi:10.3762/bjoc.14.182)
Supplement: File 1 — Experimental procedures, additional experiments, copies of 1H, 13C{1H} and 31P NMR of all new compounds, crystallographic data and copies of HPLC chromatograms. [file Beilstein_J_Org_Chem-14-2082-s001.pdf]

Supporting Information

for

D-Fructose-based spiro-fused PHOX ligands: synthesis and application in enantioselective allylic alkylation

Michael R. Imrich[1], Jochen Kraft[1], Cäcilia Maichle-Mössmer[2] and Thomas Ziegler*[1]

Address: [1]Institute of Organic Chemistry, University of Tübingen, Auf der Morgenstelle 18, 72076 Tübingen, Germany and [2]Institute of Inorganic Chemistry, University of Tübingen, Auf der Morgenstelle 18, 72076 Tübingen, Germany

Email: Thomas Ziegler - thomas.ziegler@uni-tuebingen.de

*Corresponding author

Experimental procedures, additional experiments, copies of ^{1}H, ^{13}C{1H} and ^{31}P NMR of all new compounds, crystallographic data and copies of HPLC chromatograms

Table of contents

1 Experimental procedures S3

1.1 General S3

1.2 Synthesis of 1,2-isopropylidene protected sugars S3

1.3 General procedure for the preparation of oxazolines S5

1.4 Zemplén deacetylation of **10i** and benzylation of **10j** S16

1.5 General procedure for the Ullmann coupling: S17

1.6 General procedure for the Tsuji–Trost reaction S24

2 Optimization of reaction conditions for the Ritter reaction S26

3 Ullmann coupling with longer reaction times S27

4 Crystal data for **10j** S28

5 NMR spectra S41

6 HPLC chromatograms S113

7 References S121

1 Experimental procedures

1.1 General

All reactions were carried out under an atmosphere of nitrogen. Dry toluene was distilled from sodium, dry CH_2Cl_2 and DMF were distilled from P_4O_{10}. Dry solvents were stored over molecular sieves under an atmosphere of nitrogen. For reaction monitoring TLC plates from Macherey-Nagel "Polygram Sil G/U_{254}" were used. Preparative column chromatography was performed with silica gel "60 M" which was purchased from Macherey-Nagel, solvents used as eluents were from technical grade and distilled prior to their use. Petroleum ether (PE) refers to the fraction boiling at 60–90 °C. NMR spectra were recorded on a Bruker "Avance 400" spectrometer and calibrated to the solvent signal ($CDCl_3$: ^{1}H 7.27 ppm, ^{13}C 77.0 ppm; CD_3OD: ^{1}H 3.3 ppm, ^{13}C 49.2 ppm). For peak assignment additional NMR spectra (DEPT-135, ^{1}H,^{1}H-COSY, ^{1}H,^{13}C-HMBC, ^{1}H,^{13}C-HSQC) were used, atoms are numbered according to the carbohydrate nomenclature. High-resolution mass spectra were measured on a Bruker "maXis 4G" with electrospray ionization and a time of flight detector. Optical rotations were determined at Perkin-Elmer "Polarimeter 341". Melting points were measured at a Büchi "Melting Point M-560" apparatus. Elemental analysis was performed on a HEKAtech "Euro 3000 CHN". X-ray data was collected on a Bruker "SMART APEX II DUO" diffractometer. Enantiomeric ratio was determined by HLPC, using a Sykam "S 1121" chromatograph equipped with a Dr Maisch "Reprosil Chiral-NR, 8 µm, 150 × 4.6 mm" column and *n*-hexane/2-propanol (9/1) as eluent with a flow rate of 1.6 mL/min.

1.2 Synthesis of 1,2-isopropylidene protected sugars

6a, **7e**, **7f**, **7i**, **7 j**, **7l** and **7m** were prepared as described in the literature [1-7].

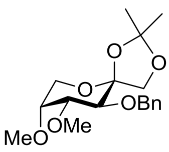

3-*O*-Benzyl-1,2-*O*-isopropylidene-4,5-di-*O*-methyl-β-D-fructopyranose (**7g**): NaH (544 mg, 13.6 mmol; 60% dispersion in mineral oil) was added to a solution of 3-*O*-benzyl-1,2-*O*-isopropylidene-β-D-fructopyranose [8] (1.06 g, 3.40 mmol) in dry DMF (30 mL) over a period of 10 min. The mixture was cooled in an ice-bath and MeI (0.85 mL, 14 mmol) was added. The ice-bath was removed and the reaction was stirred at room temperature for 5 h. The mixture was quenched with MeOH (5 mL) and the solvent was evaporated in vacuo. The residue was re-dissolved in EtOAc (50 mL), washed

with water (30 mL) and brine (30 mL) and dried over Na_2SO_4, filtered and concentrated. Column chromatography (PE/EtOAc, 3/1) of the residue afforded **7g** (955 mg, 83%) as a colorless oil. R_f = 0.29 (PE/EtOAc, 2/1); $[\alpha]^{20}_D$ -96.1 (c = 1.0, $CHCl_3$); ^{1}H NMR (400 MHz, $CDCl_3$) δ = 7.34 – 7.13 (m, 5 H, H-Ar), 4.91 (d, *J* = 11.6 Hz, 1 H, C*H*$_2$Ph), 4.53 (d, *J* = 11.6 Hz, 1 H, C*H*$_2$Ph), 3.90 – 3.78 (m, 3 H, H-1a, H-1b, H-6a), 3.72 – 3.64 (m, 2 H, H-4, H-6b), 3.63 – 3.57 (m, 2 H, H-3, H-5), 3.43 (s, 3 H, OCH_3), 3.41 (s, 3 H, OCH_3), 1.41 (s, 3 H, CH_3), 1.34 (s, 3 H, CH_3); ^{13}C{^{1}H} NMR (101 MHz , $CDCl_3$) δ = 138.5, 128.2, 127.7, 127.5 (C-Ar), 111.8 (*C*(CH_3)$_2$), 105.7 (C-2), 81.4 (C-5), 75.6 (C-3), 75.3 (*C*H_2Ph), 74.9 (C-4), 71.8 (C-1), 59.8 (C-6), 57.4 (OCH_3), 57.3 (OCH_3), 27.0 (CH_3), 26.1 (CH_3); HRMS (ESI-TOF) m/z $[M+Na]^+$: calcd for $C_{18}H_{26}O_6Na$: 361.16216, found: 361.16240; Anal calcd for $C_{18}H_{26}O_6$: C 63.89, H 7.74, found: C 63.72, H 7.66.

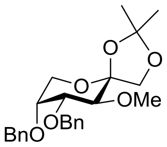

4,5-Di-*O*-benzyl-1,2-*O*-isopropylidene-3-*O*-methyl-β-D-fructopyranose (**7h**): NaH (349 mg, 8.73 mmol; 60% dispersion in mineral oil) was added to a solution of 1,2-*O*-isopropylidene-3-*O*-methyl-β-D-fructopyranose [9] (511 mg, 2.20 mmol) in dry DMF (15 mL) over a period of 10 min. The mixture was cooled in an ice-bath and BnBr (1.04 mL, 8.73 mmol) was added. The ice-bath was removed and the reaction was stirred at room temperature for 3 h. The reaction mixture was poured into ice-water (20 mL). The aqueous layer was extracted with CH_2Cl_2 (3 × 30 mL). The combined organic layers were dried over Na_2SO_4, filtered and the solvent was evaporated in vacuo. Column chromatography (PE/EtOAc, 6/1) of the residue afforded **7h** (825 mg, 91%) as a colorless crystalline solid. R_f = 0.29 (PE/EtOAc, 6/1); $[\alpha]^{20}_D$ -86.3 (c = 1.0, $CHCl_3$); mp = 108 °C (CH_2Cl_2); ^{1}H NMR (400 MHz, $CDCl_3$) δ = 7.41 – 7.24 (m, 10 H, H-Ar), 4.77 – 4.68 (m, 2 H, C*H*$_2$Ph), 4.67 – 4.58 (m, 2 H, C*H*$_2$Ph), 4.09 – 4.01 (m, 2 H, H-1a, H-1b), 3.86 – 3.65 (m, 5 H, H-3, H-4, H-5, H-6a, H-6b), 3.64 (s, 3 H, OCH_3), 1.49 (s, 3 H, CH_3), 1.44 (s, 3 H, CH_3); ^{13}C{^{1}H} NMR (101 MHz, $CDCl_3$) δ = 138.7, 138.4, 128.5, 128.5, 128.0, 127.8, 127.6 (C-Ar),

111.9 ($C(CH_3)_2$), 106.1 (C-2), 80.1 (C-4 or C-5), 77.3 (C-3), 73.4 (C-4 or C-5), 72.0 (C-1), 72.0 (CH_2Ph), 71.6 (CH_2Ph), 61.8 (OCH_3), 61.3 (C-6), 27.2 (CH_3), 26.2 (CH_3); HRMS (ESI-TOF) m/z $C_{24}H_{30}O_6Na$ $[M+Na]^+$: calcd for: 437.19346, found: 437.19369; Anal calcd for $C_{24}H_{30}O_6$: C 69.55, H 7.30, found: C 69.74, H 7.39.

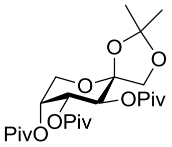

1,2-*O*-Isopropylidene-3,4,5-tri-*O*-pivaloyl-β-D-fructopyranose (**7k**): To an ice-cooled solution of 1,2-*O*-isopropylidene-β-D-fructopyranose (**7a**) (2.73 g, 12.4 mmol) in pyridine (40 mL) pivaloyl chloride (7.6 mL, 62 mmol) was added. The resulting mixture was stirred at room temperature for 3 days. The solvent was evaporated in vacuo, the residue was re-dissolved in EtOAc (50 mL), washed with water (30 mL), dried over Na_2SO_4, filtered and concentrated. Column chromatography (PE/EtOAc, 10/1) of the residue afforded **7k** (4.13 g, 70%) as a colorless crystalline solid. R_f = 0.59 (PE/EtOAc, 4/1); $[\alpha]^{20}_D$ -107.5 (c = 1.0, $CHCl_3$); mp = 130 °C (PE/EtOAc, 10/1); ^1H NMR (400 MHz ,$CDCl_3$) δ = 5.46 (d, *J* = 10.5 Hz, 1 H, H-3), 5.40 – 5.34 (m, 1 H, H-4), 5.33 – 5.30 (m, 1 H, H-5), 4.11 (dd, *J* = 1.2, 13.0 Hz, 1 H, H-6a), 3.98 (d, *J* = 9.3 Hz, 1 H, H-1a), 3.84 (d, *J* = 9.3 Hz, 1 H, H-1b), 3.73 (dd, *J* = 1.9, 13.0 Hz, 1 H, H-6b), 1.48 (s, 3 H, CH_3), 1.46 (s, 3 H, CH_3), 1.26 (s, 9 H, $C(CH_3)_3$), 1.20 (s, 9 H, $C(CH_3)_3$), 1.12 (s, 9 H, $C(CH_3)_3$); $^{13}C\{^1H\}$ NMR (101 MHz , $CDCl_3$) δ = 177.8, 177.4, 177.3 (CO), 112.0 ($C(CH_3)_2$), 104.8 (C-2), 71.9 (C-1), 69.3 (C-4), 69.0 (C-5), 66.5 (C-3), 62.5 (C-6), 38.9 (CH_3), 38.9 (CH_3), 38.7 (CH_3), 27.2 ($C(CH_3)_3$), 27.1 ($C(CH_3)_3$), 27.1 ($C(CH_3)_3$), 26.4 ($C(CH_3)_3$), 26.4 ($C(CH_3)_3$), 26.1 ($C(CH_3)_3$); HRMS (ESI-TOF) m/z $[M+Na]^+$: calcd for $C_{24}H_{40}O_9Na$: 495.25645, found: 495.25660; Anal calcd for $C_{24}H_{40}O_9$: C 61.00, H 8.53, found: C 60.77, H 8.59.

1.3 General procedure for the preparation of oxazolines:

A mixture of 1,2-isopropylidene protected sugar (1.0 equiv), 2-bromobenzonitrile (15 equiv) and $BF_3{\cdot}OEt_2$ (1 to 4 equiv, see Table 1 for details) in dry CH_2Cl_2 was stirred at room

temperature until TLC showed full consumption of the starting material (see **Table 1** for reaction times). The reaction was quenched with an excess of Et_3N and the solvent evaporated in vacuo. The residue was purified by column chromatography.

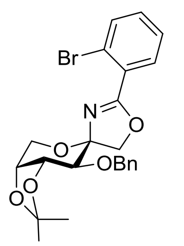

(3a'*R*,4*R*,7'*S*,7a'*R*)-7'-(Benzyloxy)-2-(2-bromophenyl)-2',2'-dimethyl-3a',4',7',7a'-tetrahydro-5*H*-spiro[oxazole-4,6'-[1,3]dioxolo[4,5-c]pyran] (**10a):** Prepared from **7e** (764 mg, 2.18 mmol) with 2-bromobenzonitrile (5.95 g, 32.7 mmol) and $BF_3{\cdot}OEt_2$ (0.60 mL, 2.2 mmol; 48% in Et_2O) in 5 mL CH_2Cl_2. Column chromatography (PE/EtOAc, 8/1 + 2% Et_3N) afforded **10a** (600 mg, 58%) as a colorless oil. R_f = 0.24 (PE/EtOAc, 10/1 + 2% Et_3N); $[\alpha]^{20}_D$ -145.2 (c = 1.0, $CHCl_3$); ^{1}H NMR (400 MHz, $CDCl_3$) δ = 7.73 – 7.58 (m, 2 H, H-Ar), 7.36 – 7.20 (m, 7 H, H-Ar), 4.97 (d, *J* = 12.1 Hz, 1 H, C*H*$_2$Ph), 4.68 (d, *J* = 12.2 Hz, 1 H, C*H*$_2$Ph), 4.60 (dd, *J* = 5.9, 7.1 Hz, 1 H, H-4), 4.51 (dd, *J* = 2.8, 13.3 Hz, 1 H, H-6a), 4.36 – 4.27 (m, 2 H, H-1a, H-5), 4.15 (d, *J* = 9.4 Hz, 1 H, H-1b), 4.06 (d, *J* = 13.3 Hz, 1 H, H-6b), 3.60 (d, *J* = 7.2 Hz, 1 H, H-3), 1.57 (s, 3 H, CH_3), 1.40 (s, 3 H, CH_3); $^{13}C\{^1H\}$ NMR (101 MHz, $CDCl_3$) δ = 166.3 (OCN), 138.4, 134.0, 132.1, 131.7, 129.6, 128.4, 128.0, 127.7, 127.2, 122.2 (C-Ar), 109.0 (*C*$(CH_3)_2$), 101.7 (C-2), 78.4 (C-4), 78.4 (C-3), 74.7 (C-1), 74.4 (C-5), 72.6 (*C*H_2Ph), 61.7 (C-6), 28.5 (CH_3), 26.5 (CH_3); HRMS (ESI-TOF) m/z $[M+H]^+$: calcd for $C_{23}H_{25}NBrO_5$: 474.09106, found: 474.09062; Anal calcd for $C_{23}H_{24}NBrO_5$: C 58.24, H 5.10, N 2.95, found: C 58.51, H 5.29, N 2.89.

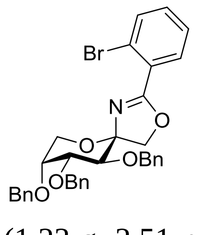

(5*R*,8*R*,9*R*,10*S*)-8,9,10-Tris(benzyloxy)-2-(2-bromophenyl)-3,6-dioxa-1-aza-spiro[4.5]dec-1-ene **10b** and (5*S*,8*R*,9*R*,10*S*)-8,9,10-tris(benzyloxy)-2-(2-bromophenyl)-3,6-dioxa-1-azaspiro[4.5]dec-1-ene (**11b**): Prepared from **7f** (1.23 g, 2.51 mmol) with 2-bromobenzonitrile (6.83 g, 37.5 mmol) and $BF_3{\cdot}OEt_2$ (0.64 mL, 2.51 mmol; 48% in Et_2O) in 7 mL CH_2Cl_2. Column chromatography (PE/EtOAc, 8/1 + 2% Et_3N) afforded both anomers in two different fractions. Eluted first: **10b** (1.07 g, 69%) as a

colorless oil. R_f = 0.63 (PE/EtOAc, 2/1 + 2% Et_3N); $[\alpha]^{20}_D$ -100.6 (c = 1.0, $CHCl_3$); ^1H NMR (400 MHz, $CDCl_3$): δ = 7.58 (dd, *J* = 1.9, 7.5 Hz, 1 H, H-Ar), 7.50 (dd, *J* = 1.2, 7.8 Hz, 1 H, H-Ar), 7.30 – 7.29 (m, 2 H, H-Ar), 7.24 – 7.12 (m, 15 H, H-Ar), 4.96 (d, *J* = 11.9 Hz, 1 H, C*H*$_2$Ph), 4.70 – 4.61 (m, 3 H, C*H*$_2$Ph), 4.52 (s, 2 H, C*H*$_2$Ph), 4.14 – 4.10 (m, 3 H, H-1a, H-1b, H-4), 4.03 (d, *J* = 12.2 Hz, 1 H, H-6a), 3.96 (d, *J* = 9.6 Hz, 1 H, H-3), 3.78 - 3.73 (m, 2 H, H-5, H-6b). $^{13}C\{^1H\}$ NMR (101 MHz, $CDCl_3$): δ = 166.1 (OCN), 138.8, 138.6, 138.5, 134.0, 132.0, 131.8, 129.8, 128.5, 128.5, 128.4, 128.1, 127.8, 127.7, 127.6, 127.1, 122.1 (C-Ar), 103.4 (C-2), 80.6 (C-4), 77.6 (C-3), 75.2 (*C*H$_2$Ph), 75.0 (C-1), 73.9 (C-5), 72.2 (*C*H$_2$Ph), 71.9 (*C*H$_2$Ph), 63.2 (C-6); HRMS (ESI-TOF) m/z $[M+H]^+$: calcd for $C_{34}H_{33}NBrO_5$: 614.15366, found: 614.15327; Anal. calcd for $C_{34}H_{32}NBrO_5$: C 66.45, H 5.25, N 2.28, found: C 66.69, H 5.30, N 2.24. Eluted second: **11b** (71 mg, 5%) as a colorless crystalline solid. R_f = 0.48 (PE/EtOAc, 2/1 + 2% Et_3N); $[\alpha]^{20}_D$ -17.5 (c = 1.0, $CHCl_3$); mp = 118 °C (CH_2Cl_2); ^1H NMR (400 MHz, $CDCl_3$) δ = 7.73 – 7.64 (m, 1 H, H-Ar), 7.60 – 7.52 (m, 1 H, H-Ar), 7.39 – 7.31 (m, 2 H, H-Ar), 7.30 – 7.12 (m, 15 H, H-Ar), 4.78 – 4.66 (m, 3 H, C*H*$_2$Ph), 4.63 – 4.56 (m, 3 H, C*H*$_2$Ph), 4.52 (d, *J* = 9.4 Hz, 1 H, H-1a), 4.28 – 4.24 (m, 2 H, H-1b, H-3), 4.05 (dd, *J* = 3.3, 13.0 Hz, 1 H, H-6a), 3.71 (m, 1 H, H-5), 3.43 (dd, *J* = 3.2, 9.4 Hz, 1 H, H-4), 3.35 (dd, *J* = 1.4, 13.0 Hz, 1 H, H-6b); $^{13}C\{^1H\}$ NMR (101 MHz, $CDCl_3$) δ = 166.8 (OCN), 138.5, 138.3, 138.1, 133.9, 132.0, 129.1, 128.3, 128.3, 128.2, 127.9, 127.6, 127.6, 127.4, 127.0, 121.8 (C-Ar), 104.0 (C-2), 78.7 (C-3), 78.3 (C-4), 75.1 (*C*H$_2$Ph), 72.4 (C-5), 72.2 (*C*H$_2$Ph), 71.1 (C-1), 71.1 (*C*H$_2$Ph), 61.7 (C-6); HRMS (ESI-TOF) m/z $[M+H]^+$: calcd for $C_{34}H_{33}NBrO_5$: 614.15366, found: 614.15375; Anal calcd for $C_{34}H_{32}NBrO_5$: C 66.45, H 5.25, N 2.28, found: C 66.06, H 5.28, N 2.27.

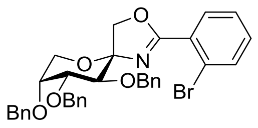

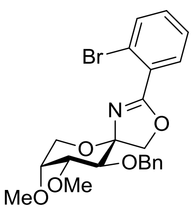

(5*R*,8*R*,9*R*,10*S*)-10-(Benzyloxy)-2-(2-bromophenyl)-8,9-dimethoxy-3,6-dioxa-1-azaspiro [4.5] dec-1-ene (**10c)** and (5*S*,8*R*,9*R*,10*S*)-10-(benzyloxy)-2-(2-bromophenyl)-8,9-dimethoxy-3,6-dioxa-1-azaspiro[4.5]dec-1-ene (**11c**): Prepared from **7g** (675 mg, 2.0 mmol) with 2-bromobenzonitrile (5.45 g, 29.9 mmol) and $BF_3·OEt_2$ (0.53 mL, 1.99 mmol; 48% in Et_2O) in 4 mL CH_2Cl_2. Column chromatography (PE/EtOAc, 3/1 → 1/1 + 2% Et_3N) afforded both anomers in two different fractions. Eluted first: **10c** (569 mg, 62%) as a colorless crystalline solid. R_f = 0.49 (PE/EtOAc, 3/2 + 2% Et_3N); $[\alpha]^{20}_D$ -150.4 (c = 1.0, $CHCl_3$); mp = 99 °C (EtOAc); ^1H NMR (400 MHz, $CDCl_3$) δ = 7.67 (dd, *J* = 2.0, 7.5 Hz, 1 H, H-Ar), 7.60 (dd, *J* = 1.3, 7.9 Hz, 1 H, H-Ar), 7.33 – 7.19 (m, 7 H, H-Ar), 5.00 (d, *J* = 12.0 Hz, 1 H, CH_2Ph), 4.66 (d, *J* = 12.0 Hz, 1 H, CH_2Ph), 4.17 (s, 2 H, H-1a, H-1b), 4.15 – 4.10 (m, 1 H, H-6a), 3.99 – 3.90 (m, 2 H, H-4, H-6b), 3.85 (d, *J* = 9.7 Hz, 1 H, H-3), 3.76 – 3.71 (m, 1 H, H-5), 3.50 (s, 3 H, OCH_3), 3.49 (s, 3 H, OCH_3); $^{13}C\{^1H\}$ NMR (101 MHz, $CDCl_3$) δ = 166.1 (OCN), 138.7, 133.8, 131.9, 131.6, 129.7, 128.2, 127.6, 127.4, 127.0, 122.0 (C-Ar), 103.0 (C-2), 81.7 (C-4), 77.3 (C-3), 76.1 (C-5), 74.9 (*C*H_2Ph), 74.9 (C-1), 61.5 (C-6), 57.7 (OCH_3), 57.3 (OCH_3); HRMS (ESI-TOF) m/z $[M+H]^+$: calcd for $C_{22}H_{25}NBrO_5$: 462.09106, found: 462.09093; Anal calcd for $C_{22}H_{24}NBrO_5$: C 57.15, H 5.23, N 3.03, found: C 57.04, H 5.29, N 2.85. Eluted second: **11c** (79 mg, 9%) as a colorless crystalline solid. R_f = 0.25 (PE/*i*PrOH, 7/1 + 2% Et_3N); $[\alpha]^{20}_D$ -22.5 (c = 1.0, $CHCl_3$); mp = 130 °C (EtOAc); ^1H NMR (400 MHz, $CDCl_3$) δ = 7.73 – 7.64 (m, 1 H, H-Ar), 7.61 – 7.51 (m, 1 H, H-Ar), 7.29 – 7.08 (m, 7 H, H-Ar), 4.77 – 4.67 (m, 2 H, CH_2Ph), 4.57 (d, *J* = 9.3 Hz, 1 H, H-1a), 4.29 (d, *J* = 9.3 Hz, 1 H, H-1b), 4.17 – 4.07 (m, 2 H, H-3, H-6a), 3.63 – 3.59 (m, 1 H, H-5), 3.45 (s, 3 H, OCH_3), 3.41 (s, 3 H, OCH_3), 3.36 (dd, *J* = 1.1, 13.2 Hz, 1 H, H-6b), 3.22 (dd, *J* = 3.2, 9.6 Hz, 1 H, H-4); $^{13}C\{^1H\}$ NMR (101 MHz, $CDCl_3$) δ = 166.9 (OCN), 138.5, 133.9, 132.1, 132.0, 129.0, 128.1, 127.7, 127.4, 127.0, 121.8 (C-Ar), 103.9 (C-2), 80.7 (C-4), 78.4 (C-3), 75.2

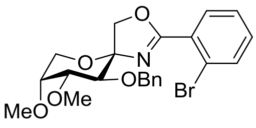

(*C*H_2Ph), 74.8 (C-5), 70.8 (C-1), 60.5 (C-6), 58.0 (OCH_3), 56.9 (OCH_3); HRMS (ESI-TOF) m/z $[M+H]^+$: calcd for $C_{22}H_{25}NBrO_5$: 462.09106, found: 462.09117; Anal calcd for $C_{22}H_{24}NBrO_5$: C 57.15, H 5.23, N 3.03, found: C 57.07, H 5.30, N 2.93.

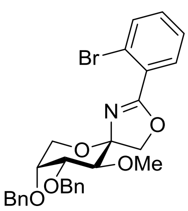

(5*R*,8*R*,9*R*,10*S*)-8,9-Bis(benzyloxy)-2-(2-bromophenyl)-10-methoxy-3,6-dioxa-1-azaspiro[4.5]dec-1-ene (**10d**) and (5*S*,8*R*,9*R*,10*S*)-8,9-bis(benzyloxy)-2-(2-bromophenyl)-10-methoxy-3,6-dioxa-1-azaspiro[4.5]dec-1-ene (**11d**): Prepared from **7h** (517 mg, 1.25 mmol) with 2-bromobenzonitrile (3.41 g, 18.7 mmol) and $BF_3 \cdot OEt_2$ (0.33 mL, 1.3 mmol; 48% in Et_2O) in 2 mL CH_2Cl_2. Column chromatography (PE/EtOAc, 8/1 → 1/1 + 2% Et_3N) afforded both anomers in two different fractions. Eluted first: **10d** (498 mg, 74%) as a colorless oil. R_f = 0.37 (PE/EtOAc, 2/1 + 2% Et_3N); $[\alpha]^{20}_D$ -123.3 (c = 1.0, $CHCl_3$); ^1H NMR (400 MHz, $CDCl_3$) δ = 7.70 (dd, *J* = 1.8, 7.6 Hz, 1 H, H-Ar), 7.5 (dd, *J* = 1.2, 7.6 Hz, 1 H, H-Ar), 7.35 – 7.15 (m, 12 H, H-Ar), 4.74 – 4.62 (m, 2 H, C*H_2*Ph), 4.62 – 4.51 (m, 2 H, C*H_2*Ph), 4.33 – 4.23 (m, 2 H, H-1a, H-1b), 4.12 – 4.01 (m, 2 H, H-4, H-6a), 3.82 – 3.70 (m, 3 H, H-3, H-5, H-6b), 3.59 (s, 3 H, OCH_3); $^{13}C\{^1H\}$ NMR (101 MHz, $CDCl_3$) δ = 165.6 (OCN), 138.3, 134.3, 134.0, 133.8, 133.1, 131.8, 129.1, 128.3, 127.6, 127.5, 127.5, 126.9, 122.2 (C-Ar), 103.2 (C-2), 80.3 (C-4), 79.5 (C-3), 74.6 (C-1), 73.7 (C-5), 71.9 (*C*H_2Ph), 71.6 (*C*H_2Ph), 62.8 (C-6), 61.6 (OCH_3); HRMS (ESI-TOF) m/z $[M+H]^+$: calcd for $C_{28}H_{29}NBrO_5$: 538.12236, found: 538.12259; Anal calcd for $C_{28}H_{28}NBrO_5$: C 62.46, H 5.24, N 2.60, found: C 62.37, H 5.22, N 2.64. Eluted second: **11d** (79 mg, 12%) as a colorless crystalline solid.

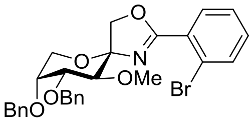

R_f = 0.65 (PE/EtOAc, 2/1 + 2% Et_3N); $[\alpha]^{20}_D$ -25.3 (c = 1.0, $CHCl_3$); mp = 104 °C (CH_2Cl_2), ^1H NMR (400 MHz, $CDCl_3$) δ = 7.72 (dd, *J* = 1.9, 7.8 Hz, 1 H, H-Ar), 7.57 (dd, *J* = 1.3, 7.8 Hz, 1 H, H-Ar), 7.36 – 7.16 (m, 12 H, H-Ar), 4.73 – 4.67 (m, 1 H, C*H_2*Ph), 4.64 – 4.56 (m, 3 H, C*H_2*Ph), 4.45 (d, *J* = 9.4 Hz, 1 H, H-1a), 4.21 (d, *J* = 9.4 Hz, 1 H, H-1b), 4.03 (dd, *J* = 3.5, 12.9 Hz, 1 H, H-6a), 3.95 (d, *J* = 9.2 Hz, 1 H, H-3), 3.68 – 3.66 (m, 1 H, H-5), 3.50 (s, 3 H,

OCH_3), 3.37 (dd, *J* = 3.2, 9.2 Hz, 1 H, H-4), 3.31 (dd, *J* = 1.5, 12.9 Hz, 1 H, H-6b); $^{13}C\{^1H\}$ NMR (101 MHz, $CDCl_3$) δ = 166.6 (OCN), 138.4, 138.1, 133.9, 132.0, 131.8, 129.3, 128.3, 127.8, 127.6, 127.5, 127.0, 121.8 (C-Ar), 104.0 (C-2), 80.3 (C-3), 77.9 (C-4), 72.4 (C-5), 72.1 ($\mathit{C}H_2Ph$), 71.1 (C-1), 71.0 ($\mathit{C}H_2Ph$), 61.7 (C-6), 61.0 (OCH_3); HRMS (ESI-TOF) m/z $[M+H]^+$: calcd for $C_{28}H_{29}NBrO_5$: 538.12236, found: 538.12267; Anal calcd for $C_{28}H_{28}NBrO_5$: C 62.46, H 5.24, N 2.60, found: C 62.43, H 5.42, N 2.54.

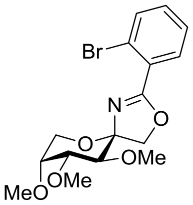

(5*R*,8*R*,9*R*,10*S*)-2-(2-Bromophenyl)-8,9,10-trimethoxy-3,6-dioxa-1-aza-spiro[4.5]dec-1-ene (**10e)** and (5*S*,8*R*,9*R*,10*S*)-2-(2-bromophenyl)-8,9,10-trimethoxy-3,6-dioxa-1-azaspiro[4.5]dec-1-ene (**11e**): Prepared from **7i** (551 mg, 2.10 mmol) with 2-bromobenzonitrile (5.70 g, 31.5 mmol) and $BF_3\cdot OEt_2$ (0.55 mL, 2.1 mmol; 48% in Et_2O) in 3 mL CH_2Cl_2. Column chromatography (PE/EtOAc, 4/1 → 1/1 + 2% Et_3N) afforded both anomers in two different fractions. Eluted first: **10e** (532 mg, 66%) as a colorless crystalline solid. R_f = 0.44 (PE/EtOAc, 1/1 + 2% Et_3N); $[\alpha]^{20}_D$ -173.5 (c = 1.0, $CHCl_3$); mp = 117 °C (CH_2Cl_2); ^1H NMR (400 MHz, $CDCl_3$) δ = 7.77 (dd, *J* = 2.0, 7.6 Hz, 1 H, H-Ar), 7.64 (dd, *J* = 1.2, 7.9 Hz, 1 H, H-Ar), 7.37 – 7.25 (m, 2 H, H-Ar), 4.37 – 4.29 (m, 2 H, H-1a, H-1b), 4.13 (dd, *J* = 1.1, 12.7 Hz, 1 H, H-6a), 3.98 – 3.88 (m, 2 H, H-4, H-6b), 3.76 – 3.72 (m, 1 H, H-5), 3.63 – 3.58 (m, 4 H, H-3, OCH_3), 3.51 (s, 6 H, 2 × OCH_3); $^{13}C\{^1H\}$ NMR (101 MHz, $CDCl_3$) δ = 165.8 (OCN), 133.9, 131.9, 131.8, 129.2, 126.9, 122.1 (C-Ar), 103.0 (C-2), 81.5 (C-4), 79.2 (C-3), 76.2 (C-5), 74.5 (C-1), 61.5 (OCH_3), 61.3 (C-6), 57.6 (OCH_3), 57.2 (OCH_3); HRMS (ESI-TOF) m/z $[M+H]^+$: calcd for $C_{16}H_{21}NBrO_5$: 386.05976, found: 386.05989; Anal calcd for $C_{16}H_{20}NBrO_5$: C 49.76, H 5.22, N 3.63, found: C 49.75, H 5.25, N 3.57. Eluted second: **11e** (160 mg, 20%) as a colorless oil. R_f = 0.23 (PE/EtOAc, 1/1 + 2% Et_3N); $[\alpha]^{20}_D$ -38.2 (c = 1.0, $CHCl_3$); ^1H NMR (400 MHz, $CDCl_3$) δ = 7.78 (dd, *J* = 2.9, 7.8 Hz, 1 H, H-Ar), 7.63 (dd, *J* = 1.2, 7.8 Hz, 1 H, H-Ar), 7.37 – 7.26 (m, 2 H, H-Ar), 4.56 (d, *J* = 9.4 Hz, 1 H, H-

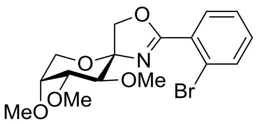

1a), 4.32 (d, J = 9.4 Hz, 1 H, H-1b), 4.16 (dd, J = 3.1, 13.2 Hz, 1 H, H-6a), 3.89 (d, J = 9.5 Hz, 1 H, H-3), 3.66 – 3.64 (m, 1 H, H-5), 3.55 (s, 3 H, OCH_3), 3.52 (s, 3 H, OCH_3), 3.46 (s, 3 H, OCH_3), 3.40 (dd, J = 1.4, 13.2 Hz, 1 H, H-6b), 3.24 (dd, J = 3.3, 9.5 Hz, 1 H, H-4); $^{13}C\{^1H\}$ NMR (101 MHz, $CDCl_3$) δ = 166.7 (OCN), 133.8, 132.0, 131.7, 129.2, 127.0, 121.7 (C-Ar), 103.9 (C-2), 80.3 (C-4), 79.8 (C-3), 74.6 (C-5), 70.8 (C-1), 61.0 (OCH_3), 60.4 (C-6), 57.7 (OCH_3), 56.8 (OCH_3); HRMS (ESI-TOF) m/z $[M+H]^+$: calcd for $C_{16}H_{21}NBrO_5$: 386.05976, found: 386.05976; Anal calcd for $C_{16}H_{20}NBrO_5$: C 49.76, H 5.22, N 3.63, found: C 49.60, H 5.28, N 3.37.

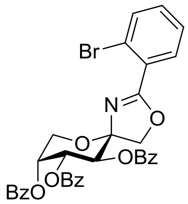

(5R,8R,9R,10S)-2-(2-Bromophenyl)-3,6-dioxa-1-azaspiro[4.5]dec-1-ene-8,9,10-triyl tribenzoate (**10f**) and (5S,8R,9R,10S)-2-(2-bromophenyl)-3,6-dioxa-1-azaspiro[4.5]dec-1-ene-8,9,10-triyl tribenzoate (**11f**): Prepared from **7j** (1.12 g, 2.10 mmol) with 2-bromobenzonitrile (5.73 g, 31.5 mmol) and $BF_3{\cdot}OEt_2$ (2.20 mL, 8.40 mmol; 48% in Et_2O) in 4 mL CH_2Cl_2. Column chromatography (PE/EtOAc, 5/1 + 2% Et_3N) afforded both anomers in two different fractions. Eluted first: **10f** (618 mg, 45%) as a colorless crystalline solid. R_f = 0.30 (toluene/EtOAc, 45/1 + 2% Et_3N); $[\alpha]^{20}_D$ -158.4 (c = 1.0, $CHCl_3$); mp = 74 °C (CH_2Cl_2); ^1H NMR (400 MHz, $CDCl_3$) δ = 8.09 – 8.01 (m, 2 H, H-Ar), 7.93 – 7.84 (m, 2 H, H-Ar), 7.78 – 7.67 (m, 3 H, H-Ar), 7.62 (dd, J = 1.4, 7.8 Hz, 1 H, H-Ar), 7.57 – 7.47 (m, 1 H, H-Ar), 7.45 – 7.09 (m, 10 H, H-Ar), 6.12 – 6.00 (m, 2 H, H-3, H-4), 5.80 (d, J = 2.1 Hz, 1 H, H-5), 4.64 (dd, J = 1.2, 13.1 Hz, 1 H, H-6a), 4.44 – 4.30 (m, 2 H, H-1a, H-1b), 4.06 (dd, J = 1.8, 13.1 Hz, 1 H, H-6b); $^{13}C\{^1H\}$ NMR (101 MHz, $CDCl_3$) δ = 166.7 (OCN), 166.1, 165.8, 165.5 (CO), 134.2, 133.5, 133.3, 133.1, 132.4, 131.6, 129.9, 129.9, 129.7, 129.6, 129.1, 128.8, 128.8, 128.5, 128.4, 128.2, 127.1, 122.2 (C-Ar), 102.6 (C-2), 74.2 (C-1), 70.6 (C-5), 70.4 (C-3 or C-4), 69.9 (C-3 or C-4), 63.7 (C-6); HRMS (ESI-TOF) m/z $[M+H]^+$: calcd for $C_{34}H_{27}NBrO_8$: 656.09146, found: 656.09228; Anal

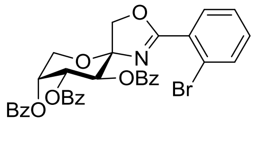

calcd for $C_{34}H_{26}NBrO_8$: C 62.21, H 3.99, N 2.13, found: C 62.15, H 4.11, N 2.08. Eluted second: **11f** (221 mg, 16%) as a colorless crystalline solid. R_f = 0.27 (PE/EtOAc, 4/1 + 2% Et_3N); $[\alpha]^{20}_D$ -100.8 (c = 1.0, $CHCl_3$); mp = 85 °C (CH_2Cl_2); ^{1}H NMR (400 MHz, $CDCl_3$) δ = 7.98 –7.84 (m, 6 H, H-Ar), 7.69 – 7.64 (m, 1 H, H-Ar), 7.62 – 7.57 (m, 1 H, H-Ar), 7.51 – 7.37 (m, 3 H, H-Ar), 7.36 – 7.15 (m, 8 H, H-Ar), 5.73 (dd, *J* = 3.8, 5.7 Hz, 1 H, H-4), 5.57 (m, 1 H, H-5), 5.00 (d, *J* = 5.7 Hz, 1 H, H-3), 4.67 – 4.53 (m, 2 H, H-1a, H-1b), 4.30 (dd, *J* = 3.6, 12.8 Hz, 1 H, H-6a), 4.18 (dd, *J* = 3.8, 12.8 Hz, 1 H, H-6b); $^{13}C\{^1H\}$ NMR (101 MHz, $CDCl_3$) δ = 166.0 (OCN), 166.0 , 165.7, 165.5 (CO), 134.2, 133.6, 133.4, 133.3, 132.8, 131.8, 130.2, 129.9, 129.8, 129.7, 129.4, 129.3, 129.1, 128.5, 128.5, 128.4, 128.4, 127.3, 122.2 (C-Ar), 100.7 (C-2), 77.2 (C-3), 70.3 (C-4), 67.1 (C-1), 66.8 (C-5), 63.1 (C-6); HRMS (ESI-TOF) m/z $[M+H]^+$: calcd for $C_{34}H_{27}NBrO_8$: 656.09146, found: 656.09181; Anal calcd for $C_{34}H_{26}NBrO_8$: C 62.21, H 3.99, N 2.13, found: C 61.88, H 4.16, N 2.06.

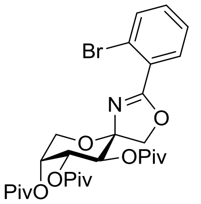

(5*R*,8*R*,9*R*,10*S*)-2-(2-Bromophenyl)-3,6-dioxa-1-azaspiro[4.5]dec-1-ene-8,9,10-triyl tris(2,2-dimethylpropanoate) (**10g**) and (5*S*,8*R*,9*R*,10*S*)-2-(2-bromophenyl)-3,6-dioxa-1-azaspiro[4.5]dec-1-ene-8,9,10-triyl tris(2,2-dimethylpropanoate) (**11g**): Prepared from **7k** (1.00 g, 2.12 mmol) with 2-bromobenzonitrile (5.79 g, 31.8 mmol) and $BF_3 \cdot OEt_2$ (2.24 mL, 8.48 mmol; 48% in Et_2O) in 4 mL CH_2Cl_2. Column chromatography (PE/EtOAc, 11/1 + 2% Et_3N) afforded both anomers in two different fractions. Eluted first: **10g** (703 mg, 56%) as a colorless crystalline solid. R_f = 0.40 (PE/EtOAc, 8/1 + 2% Et_3N); $[\alpha]^{20}_D$ -130.7 (c = 1.0, $CHCl_3$); mp = 55 °C (EtOAc); ^{1}H NMR (400 MHz, $CDCl_3$) δ = 7.78 (dd, *J* = 2.1, 7.2 Hz, 1 H, H-Ar), 7.69 (dd, *J* = 1.3, 7.8 Hz, 1 H, H-Ar), 7.39 – 7.32 (m, 2 H, H-Ar), 5.66 (dd, *J* = 3.3, 10.3 Hz, 1 H, H-4), 5.59 (d, *J* = 10.3 Hz, 1 H, H-3), 5.47 – 5.39 (m, 1 H, H-5), 4.47 (dd, *J* = 1.2, 13.0 Hz, 1 H, H-6a), 4.29 (d, *J* = 10.3 Hz, 1 H, H-1a), 4.19 (d, *J* = 10.3 Hz, 1 H, H-1b), 3.79 (dd, *J* = 1.9, 13.0 Hz, 1 H, H-6b), 1.29 (s, 9 H, $C(CH_3)_3$), 1.14 (s, 9 H, $C(CH_3)_3$), 1.12 (s, 9 H, $C(CH_3)_3$); $^{13}C\{^1H\}$ NMR (101 MHz,

$CDCl_3$) δ = 177.7, 177.4, 177.2 (CO), 166.0 (OCN), 134.4, 132.4, 131.7, 128.5, 127.1, 122.2 (H-Ar), 102.5 (C-2), 74.1 (C-1), 69.5 (C-4), 69.4 (C-5), 68.8 (C-3), 63.8 (C-6), 38.9, 38.9, 38.7 (*C*(CH_3)$_3$), 27.2, 27.1, 27.0 (C(*C*H_3)$_3$); HRMS (ESI-TOF) m/z $[M+Na]^+$: calcd for $C_{28}H_{38}NBrO_8Na$: 618.16730, found: 618.16673; Anal calcd for $C_{28}H_{38}NBrO_8$: C 56.38, H 6.42, N 2.35, found: C 56.61, H 6.50, N 2.25. Eluted second: **11g** (274 mg, 22%) as a colorless oil. R_f = 0.35 (PE/EtOAc, 8/1 + 2% Et_3N); $[\alpha]^{20}_D$ -77.7 (c = 1.0, $CHCl_3$); ^{1}H NMR (400 MHz, $CDCl_3$) δ = 7.75 (dd, *J* = 2.1, 7.5 Hz, 1 H, H-Ar), 7.67 (dd, *J* = 1.5, 7.6 Hz, 1 H, H-Ar), 7.40 – 7.30 (m, 2 H, H-Ar), 5.37 (dd, *J* = 3.4, 6.1 Hz, 1 H, H-4), 5.27 – 5.24 (m, 1 H, H-5), 4.60 (d, *J* = 6.1 Hz, 1 H, H-3), 4.40 (d, *J* = 11.1 Hz, 1 H, H-1a), 4.30 (d, *J* = 11.1 Hz, 1 H, H-1b), 4.15 (dd, *J* = 3.9, 12.8 Hz, 1 H, H-6a), 3.93 (dd, *J* = 3.8, 12.8 Hz, 1 H, H-6b), 1.25 (s, 9 H, C(CH_3)$_3$), 1.24 (s, 9 H, C(CH_3)$_3$), 1.16 (s, 9 H, C(CH_3)$_3$); ^{13}C{^{1}H} NMR (101 MHz, $CDCl_3$) δ = 177.8, 177.3, 177.2 (CO), 165.7 (OCN), 134.3, 132.7, 131.9, 128.2, 127.2, 122.1 (H-Ar), 100.2 (C-2), 77.3 (C-3), 69.7 (C-4), 66.4 (C-1), 66.0 (C-5), 63.0 (C-6), 38.9, 38.9 38.8 (*C*(CH_3)$_3$), 27.1, 27.1, 27.1 (C(*C*H_3)$_3$); HRMS (ESI-TOF) m/z $[M+H]^+$: calcd for $C_{28}H_{39}NBrO_8$: 596.18536, found: 596.18532; Anal calcd for $C_{28}H_{38}NBrO_8$: C 56.38, H 6.42, N 2.35, found: C 56.06, H 6.42, N 2.13.

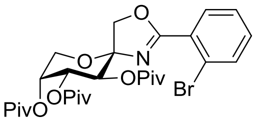

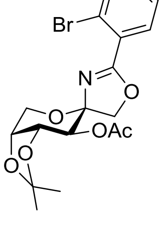

(3a'*R*,4*R*,7'*S*,7a'*R*)-2-(2-Bromophenyl)-2',2'-dimethyl-3a',4',7',7a'-tetrahydro-5*H* -spiro[oxazole-4,6'-[1,3]dioxolo[4,5-*c*]pyran]-7'-yl acetate (**10h**) and (3a'*R*,4*S*,7'*S*,7a'*R*)-2-(2-bromophenyl)-2',2'-dimethyl-3a',4',7',7a'-tetrahydro-5*H* -spiro[oxazole-4,6'-[1,3]dioxolo[4,5-c]pyran]-7'-yl acetate (**11h**): Prepared from **7l** (644 mg, 2.13 mmol) with 2-bromobenzonitrile (5.82 g, 32.0 mmol) and $BF_3{\cdot}OEt_2$ (0.85 mL, 3.2 mmol; 48% in Et_2O) in 4 mL CH_2Cl_2. Column chromatography (PE/EtOAc, 6/1 → 4/1 + 2% Et_3N) afforded both anomers in two different fractions. Eluted first: **10h** (381 mg, 42%) as a colorless crystalline solid. R_f = 0.36 (PE/EtOAc, 4/1 + 2% Et_3N); $[\alpha]^{20}_D$ -161.0

(c = 1.0, $CHCl_3$); mp = 94 °C (EtOAc); ^{1}H NMR (400 MHz, $CDCl_3$) δ = 7.69 – 7.62 (m, 2 H, H-Ar), 7.37 – 7.25 (m, 2 H, H-Ar), 5.28 (d, *J* = 7.9 Hz, 1 H, H-3), 4.52 – 4.42 (m, 2 H, H-4, H-6a), 4.31 – 4.29 (m, 1 H, H-5), 4.23 (d, *J* = 10.3 Hz, 1 H, H-1a), 4.17 (d, *J* = 10.3 Hz, 1 H, H-1b), 4.11 (d, *J* = 13.3 Hz, 1 H, H-6b), 2.09 – 2.06 (m, 3 H, CH_3), 1.57 (s, 3 H, $C(CH_3)_2$), 1.35 (s, 3 H, $C(CH_3)_2$); $^{13}C\{^1H\}$ NMR (101 MHz , $CDCl_3$) δ = 170.7 (CO), 166.6 (OCN),

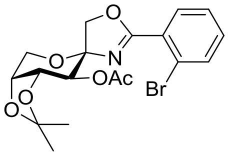

134.1, 132.3, 131.5, 129.1, 127.2, 122.0 (C-Ar), 109.5 (*C*($CH_3)_2$), 101.1 (C-2), 75.3 (C-4), 74.2 (C-5), 74.2 (C-1), 72.7 (C-3), 61.7 (C-6), 27.9, 26.5 (C(*C*$H_3)_2$), 21.1 (CH_3); HRMS (ESI-TOF) m/z $[M+H]^+$: calcd for $C_{18}H_{21}NBrO_6$: 426.05468, found: 426.05462; Anal calcd for $C_{18}H_{20}NBrO_6$: C 50.72, H 4.73, N 3.29, found: C 50.84, H 4.81, N 3.15. Eluted second: **11h** (163 mg, 18%) as a colorless oil. R_f = 0.36 (PE/EtOAc, 2/1 + 2% Et_3N); $[\alpha]^{20}_D$ -80.0 (c = 1.0, $CHCl_3$); ^{1}H NMR (400 MHz, $CDCl_3$) δ = 7.70 (dd, *J* = 2.1, 7.2 Hz, 1 H, H-Ar), 7.65 (dd, *J* = 1.5, 7.6 Hz, 1 H, H-Ar), 7.39 – 7.31 (m, 2 H, H-Ar), 4.76 (dd, *J* = 2.1, 7.8 Hz, 1 H, H-4), 4.64 (d, *J* = 2.1 Hz, 1 H, H-3), 4.56 (d, *J* = 11.2 Hz, 1 H, H-1a), 4.28 – 4.19 (m, 2 H, H-1b, H-5), 3.86 (d, *J* = 13.8 Hz, 1 H, H-6a), 3.62 (dd, *J* = 2.0, 13.8 Hz, 1 H, H-6b), 2.09 (s, 3 H, CH_3), 1.52 (s, 3 H, $C(CH_3)_2$), 1.36 (s, 3 H, $C(CH_3)_2$); $^{13}C\{^1H\}$ NMR (101 MHz, $CDCl_3$) δ = 170.5 (CO), 166.6 (OCN), 134.1, 132.6, 131.7, 128.8, 127.4, 121.7 (C-Ar), 109.6 (*C*($CH_3)_2$), 98.3 (C-2), 73.8 (C-3), 70.2 (C-5), 69.8 (C-4), 67.1 (C-1), 61.7 (C-6), 26.2, 24.4 (C(*C*$H_3)_2$), 21.0 (CH_3); HRMS (ESI-TOF) m/z $[M+Na]^+$: calcd for $C_{18}H_{20}NBrO_6Na$: 448.03662, found: 448.03646; Anal calcd for $C_{18}H_{20}NBrO_6$: C 50.72, H 4.73, N 3.29, found: C 50.73, H 4.88, N 3.20.

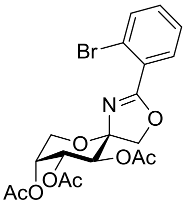

(5*R*,8*R*,9*R*,10*S*)-2-(2-Bromophenyl)-3,6-dioxa-1-azaspiro[4.5]dec-1-ene-8,9,10-triyl triacetate (**10i**) and (5*S*,8*R*,9*R*,10*S*)-2-(2-bromophenyl)-3,6-dioxa-1-azaspiro[4.5]dec-1-ene-8,9,10-triyl triacetate (**11i**): Prepared from **7m** (670 mg, 1.93 mmol) with 2-bromobenzonitrile (5.28 g, 29.0 mmol) and $BF_3{\cdot}OEt_2$ (1.50

mL, 5.79 mmol; 48% in Et_2O) in 3 mL CH_2Cl_2. Column chromatography (PE/EtOAc, 2/1 + 2% Et_3N) afforded both anomers in two different fractions. Eluted first: **10i** (458 mg, 50%) as a colorless crystalline solid. R_f = 0.34 (PE/EtOAc, 2/1 + 2% Et_3N); $[\alpha]^{20}_{D}$ -140.6 (c = 1.0, $CHCl_3$); mp = 51 °C (CH_2Cl_2); ^1H NMR (400 MHz, $CDCl_3$) δ = 7.74 (dd, *J* = 1.8, 7.5 Hz, 1 H, H-Ar), 7.66 (dd, *J* = 1.1, 7.7 Hz, 1 H, H-Ar), 7.40 – 7.29 (m, 2 H, H-Ar), 5.60 – 5.50 (m, 2 H, H-3, H-4), 5.46 – 5.47 (m, 1 H, H-5), 4.45 (dd, *J* = 0.9, 13.1 Hz, 1 H, H-6a), 4.30 – 4.28 (m, 2 H, H-1a, H-1b), 3.81 (dd, *J* = 1.7, 13.1 Hz, 1 H, H-6b), 2.17 (s, 3 H, CH_3), 2.04 (s, 3 H, CH_3), 1.98 (s, 3 H, CH_3); $^{13}C\{^1H\}$ NMR (101 MHz, $CDCl_3$) δ = 170.6, 170.5, 170.0 (CO),

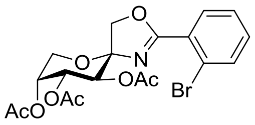

166.8 (OCN), 134.2, 132.5, 131.8, 129.0, 127.3, 122.1 (C-Ar), 102.3 (C-2), 74.3 (C-1), 69.6 (C-3), 69.5 (C-4 or C-5), 69.2 (C-4 or C-5), 63.6 (C-6), 21.1, 20.9, 20.8 (CH_3); HRMS (ESI-TOF) m/z $[M+Na]^+$: calcd for $C_{19}H_{20}NBrO_8Na$: 492.02645, found: 492.02629; Anal calcd for $C_{19}H_{20}NBrO_8$: C 48.53, H 4.29, N 2.98, found: C 48.56, H 4.45, N 2.95. Eluted second: **11i** (235 mg, 26%) as a colorless oil. R_f = 0.40 (PE/EtOAc, 1/1 + 2% Et_3N); $[\alpha]^{20}_{D}$ -92.7 (c = 1.0, $CHCl_3$); ^1H NMR (400 MHz, $CDCl_3$) δ = 7.71 – 7.63 (m, 2 H, H-Ar), 7.40 – 7.30 (m, 2 H, H-Ar), 5.32 – 5.28 (m, 1 H, H-4), 5.26 – 5.23 (m, 1 H, H-5), 4.65 (d, *J* = 6.4 Hz, 1 H, H-3), 4.40 (d, *J* = 11.4 Hz, 1 H, H-1a), 4.23 (d, *J* = 11.4 Hz, 1 H, H-1b), 4.06 (dd, *J* = 2.9, 13.0 Hz, 1 H, H-6a), 4.06 (dd, *J* = 3.6, 13.0 Hz 1 H, H-6b), 2.12 (s, 6 H, 2 × CH_3), 2.09 (s, 3 H, CH_3); $^{13}C\{^1H\}$ NMR (101 MHz, $CDCl_3$) δ = 170.5, 170.1, 170.0 (CO), 165.9 (OCN), 134.2, 132.8, 131.8, 128.5, 127.4, 122.1 (C-Ar), 100.5 (C-2), 77.0 (C-3), 70.3 (C-4), 66.5 (C-1), 66.1 (C-5), 62.9 (C-6), 20.9, 20.9, 20.9 (CH_3); HRMS (ESI-TOF) m/z $[M+H]^+$: calcd for $C_{19}H_{21}NBrO_8$: 470.04451, found: 470.04456; Anal calcd for $C_{19}H_{20}NBrO_8$: C 48.53, H 4.29, N 2.98, found: C 48.61, H 4.49, N 2.80.

1.4 Zemplén deacetylation of 10i and benzylation of 10j

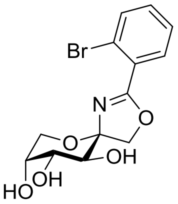

(5*R*,8*R*,9*R*,10*S*)-2-(2-Bromophenyl)-3,6-dioxa-1-azaspiro[4.5]dec-1-ene-8,9,10-triol (**10j**): To a solution of **10i** (89 mg, 0.19 mmol) in 5 mL MeOH was added NH_3 (1 mL, 7 mmol; 7 M in MeOH). The resulting mixture was stirred at room temperature for 4 h. After evaporation of the solvent in vacuo **10j** (64 mg, 99%) was obtained as colorless crystalline solid. Crystals for X-ray crystallography were prepared by covering a saturated solution of **10j** in 2-propanol with *n*-heptane. R_f = 0.56 (2-propanol/EtOAc, 1/1 + 2% Et_3N); $[\alpha]^{20}_D$ -173.1 (c = 1.0, CH_3OH); mp = 140 °C (2-propanol/*n*-heptane); ^1H NMR (400 MHz, CD_3OD) δ = 7.68 (ddd, *J* = 1.8, 7.6, 19.3 Hz, 2 H, H-Ar), 7.46 - 7.29 (m, 2 H, H-Ar), 4.51 (d, *J* = 9.7 Hz, 1 H, H-1a), 4.25 (d, *J* = 9.7 Hz, 1 H, H-1b), 4.19 (dd, *J* = 1.3, 12.5 Hz, 1 H, H-6a), 3.97 (dd, *J* = 3.4, 9.7 Hz, 1 H, H-4), 3.94 - 3.89 (m, 1 H, H-5), 3.82 (d, *J* = 9.7 Hz, 1 H, H-3), 3.69 (dd, *J* = 1.9, 12.5 Hz, 1 H, H-6b); $^{13}C\{^1H\}$ NMR (101 MHz , CD_3OD) δ = 168.6 (OCN), 135.0, 133.6, 132.7, 131.1, 128.5, 122.8 (C-Ar), 104.8 (C-2), 76.2 (C-1), 72.4 (C-4), 71.5 (C-3 or C-5), 71.4 (C-3 or C-5), 67.0 (C-6); HRMS (ESI-TOF) m/z $[M+Na]^+$: calcd for $C_{13}H_{14}NBrO_5Na$: 365.99476, found: 365.99493; Anal calcd for $C_{13}H_{14}NBrO_5$: C 61.59, H 7.00, N 2.32, found: C 61.87, H 7.15, N 2.19.

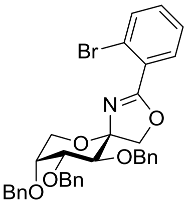

(5*R*,8*R*,9*R*,10*S*)-8,9,10-Tris(benzyloxy)-2-(2-bromophenyl)-3,6-dioxa-1-aza-spiro[4.5]dec-1-ene **10b** from **10j**: A solution of **10j** (32 mg, 0.093 mmol) and NaH (22 mg, 0.56 mmol; 60% dispersion in mineral oil) was stirred at room temperature for 10 min. BnBr (66 µL, 0.558 mmol) was added and the resulting mixture was stirred at room temperature for 90 min. The reaction was quenched with MeOH (2 mL), the solvent evaporated in vacuo, the residue was dissolved in EtOAc (10 mL), washed with water (5 mL), dried over Na_2SO_4 and concentrated. Column chromatography (PE/EtOAc, 5/1 + 2% Et_3N) offered **10b** (47 mg, 82%) as colorless oil. Analytical data is in accordance with the data described above.

1.5 General procedure for the Ullmann coupling:

A mixture of CuI (0.125 equiv), *N,N*'-dimethylethylenediamine (0.875 equiv) and diphenylphosphine (1.88 equiv) in dry toluene (0.5 M with respect to $HPPh_2$) was stirred at room temperature for 30 minutes. Cs_2CO_3 (3.75 equiv) and bromophenyloxazoline (1.00 equiv; 0.2 M in dry toluene) were added and the reaction mixture was heated to 110 °C (oil bath temperature) until TLC showed full consumption of the starting material (see table Table 2 for reaction times). The mixture was cooled to room temperature, filtered through a pad of celite and concentrated in vacuo. The residue was purified by column chromatography, after evaporation of the eluent in vacuo spiro-PHOX-ligands were obtained as colorless crystalline solids.

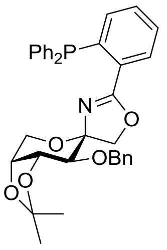

(3a'*R*,4*R*,7'*S*,7a'*R*)-7'-(Benzyloxy)-2-(2-(diphenylphosphanyl)phenyl)-2',2'-dimethyl-3a',4',7',7a'-tetrahydro-5*H*-spiro[oxazole-4,6'-[1,3]dioxolo[4,5-c]pyran] (**5a**): Prepared from **10a** (391 mg, 0.824 mmol) with CuI (19.6 mg, 0.103 mmol), *N,N*'-dimethylethylenediamine (78 µL, 0.72 mmol), $HPPh_2$ (270 µL, 1.55 mmol) and Cs_2CO_3 (1.01 g, 3.09 mmol), yield after column chromatography (PE/EtOAc, 8/1 + 2% Et_3N): 319 mg, 67%; R_f = 0.28 (PE/EtOAc, 8/1 + 2% Et_3N); $[\alpha]^{20}_{D}$ -122.0 (c = 1.0, $CHCl_3$); mp = 80 °C (CH_2Cl_2); ^{1}H NMR (400 MHz, $CDCl_3$) δ = 8.04 – 8.01 m, 1 H, H-Ar), 7.44 – 7.39 (m, 1 H, H-Ar), 7.38 – 7.17 (m, 16 H, H-Ar), 6.92 – 6.88 (m, 1 H, H-Ar), 4.85 (d, *J* = 12.3 Hz, 1 H, CH_2Ph), 4.59 (d, *J* = 12.3 Hz, 1 H, CH_2Ph), 4.30 (d, *J* = 9.3 Hz, 1 H, H-1a), 4.09 – 3.99 (m, 2 H, H-1b, H-6a), 3.94 – 3.91 (m, 1 H, H-5), 3.85 (d, *J* = 13.1 Hz, 1 H, H-6b), 3.76 – 3.69 (m, 1 H, H-4), 3.46 (d, *J* = 7.3 Hz, 1 H, H-3), 1.52 (s, 3 H, CH_3), 1.36 (s, 3 H, CH_3); ^{13}C{^{1}H} NMR (101 MHz, $CDCl_3$) δ = 164.6 (OCN), 139.5 (d, *J* = 5.1 Hz), 139.3 (d, *J* = 9.4 Hz), 139.2 (d, *J* = 9.4 Hz), 138.7, 134.8, 134.2 (d, *J* = 20.5 Hz), 133.6 (d, *J* = 19.8 Hz), 131.6 (d, *J* = 19.8 Hz), 131.2, 129.9, 128.5, 128.2, 127.9, 127.5 (C-Ar), 108.5 (*C*(CH_3), 102.0 (C-2), 78.2 (C-4), 78.1 (C-3), 74.7 (C-5), 73.9 (C-1), 71.8 (*C*H_2Ph), 62.1 (C-6), 28.4, 26.4

(CH_3); ^{31}P NMR (162 MHz, $CDCl_3$) δ = -5.00; HRMS (ESI-TOF) m/z $[M+H]^+$: calcd for $C_{35}H_{35}NO_5P$: 580.22474, found: 580.22505; Anal calcd for $C_{35}H_{34}NO_5P$: C 72.53, H 5.91, N 2.42, found: C 72.37, H 6.03, N 2.48.

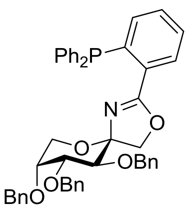

(5*R*,8*R*,9*R*,10*S*)-8,9,10-Tris(benzyloxy)-2-(2-(diphenylphosphanyl)phenyl)-3,6-dioxa-1-azaspiro[4.5]-dec-1-ene (**5b**): Prepared from **10b** (470 mg, 0.765 mmol) with CuI (18.2 mg, 0.096 mmol), *N*,*N*'-dimethylethylenediamine (72 µL, 0.67 mmol), $HPPh_2$ (250 µL, 1.44 mmol) and Cs_2CO_3 (935 mg, 2.87 mmol), yield after column chromatography (toluene/EtOAc, 35/1 + 2.5% Et_3N): 490 mg, 89%; R_f = 0.53 (toluene/EtOAc, 35/1 + 2.5% Et_3N); $[\alpha]^{20}_D$ -129.1 (c=1.0, $CHCl_3$); mp = 53 °C (CH_2Cl_2); ^{1}H NMR (400 MHz, $CDCl_3$) δ = 7.87 – 7 .85 (m, 1 H, H-Ar), 7.57 – 7.52 (m, 1 H, H-Ar), 7.35 – 7.07 (m, 24 H, H-Ar), 6.94 (t, *J* = 7.0 Hz, 2 H, H-Ar), 6.76 (dd, *J* = 3.8, 7.3 Hz, 1 H, H-Ar), 4.82 (d, *J* = 12.6 Hz, 1 H, CH_2Ph), 4.63 – 4.51 (m, 3 H, CH_2Ph), 4.32 (q, *J* = 11.6 Hz, 2 H, CH_2Ph), 4.05 (d, *J* = 9.4 Hz, 1 H, H-1a), 3.96 (d, *J* = 9.4 Hz, 1H, H-1b), 3.81 (d, *J* = 9.6 Hz, 1 H, H-3), 3.55 – 3.45 (m, 3 H, H-4, H-5, H-6a), 3.35 (d, *J* = 12.2 Hz, 1 H, H-6b); ^{13}C{^{1}H} NMR (101 MHz, $CDCl_3$) δ = 164.5 (O*C*N), 139.5 (d, *J* = 5.1 Hz), 139.4 (d, *J* = 9.4 Hz), 139.3 (d, *J* = 10.1 Hz), 139.1, 138.9, 138.7, 134.8, 134.3 (d, *J* = 20.7 Hz), 134.1 (d, *J* = 19.9 Hz), 134.0 (d, *J* = 19.9 Hz), 133.8, 133.3, 132.0, 131.8, 131.1, 129.8, 129.0, 128.9, 128.4, 128.4, 128.3, 128.3, 128.0, 127.7, 127.7, 127.7, 127.5, 127.5, 127.3, 125.5, 117.2, 116.0 (C-Ar), 104.0 (C-2), 80.4 (C-4), 77.4 (C-3), 74.5 (C-5), 74.3 (C-1), 74.1, 72.1, 71.8 (*C*H_2Ph), 63.4 (C-6); ^{31}P NMR (162 MHz, $CDCl_3$) δ = -5.82; HRMS (ESI-TOF) m/z $[M+H]^+$: calcd for $C_{46}H_{42}NO_5P$: 720.28734, found: 720.28755; Anal calcd for $C_{46}H_{42}NO_5P$: C 76.76, H 5.88, N 1.95, found: C 76.67, H 5.89, N 1.98.

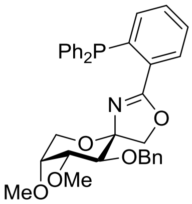

(5*R*,8*R*,9*R*,10*S*)-10-(Benzyloxy)-2-(2-(diphenylphosphanyl)phenyl)-8,9-di-methoxy-3,6-dioxa-1-azaspiro[4.5]dec-1-ene (**5c**): Prepared from **10c** (206

mg, 0.446 mmol) with CuI (10.6 mg, 0.056 mmol), *N*,*N*'-dimethylethylenediamine (41 µL, 0.39 mmol), $HPPh_2$ (155 µL, 0.838 mmol) and Cs_2CO_3 (544 mg, 1.67 mmol), yield after column chromatography (PE/EtOAc, 7/2 + 2% Et_3N): 210 mg, 83%; R_f = 0.30 (PE/EtOAc, 4/1 + 2% Et_3N); $[\alpha]^{20}_D$ -140.8 (c = 1.0, $CHCl_3$); mp = 61 °C (CH_2Cl_2); ^{1}H NMR (400MHz, $CDCl_3$) δ = 8.00 – 7.95 (m, 1 H, H-Ar), 7.35 – 7.30 (m, 1 H, H-Ar), 7.26 – 7.10 (m, 16 H, H-Ar), 6.82 – 6.78 (m, 1 H, H-Ar), 4.79 (d, *J* = 12.2 Hz, 1 H, CH_2Ph), 4.49 (d, *J* = 12.2 Hz, 1 H, CH_2Ph), 4.07 (d, *J* = 9.4 Hz, 1 H, H-1a), 4.01 (d, *J* = 9.4 Hz, 1 H, H-1b), 3.67 – 3.60 (m, 2 H, H-3, H-6a), 3.57 – 3.49 (m, 1 H, H-6b), 3.39 – 3.32 (m, 4 H, H-5, OCH_3), 3.25 – 3.17 (m, 4 H, H-4, OCH_3); ^{13}C{^{1}H} NMR (101MHz , $CDCl_3$) δ = 164.5 (OCN), 139.4 (d, *J* = 12.5 Hz), 139.2 (d, *J* = 6.6 Hz), 139.0 (d, *J* = 10.3 Hz), 134.7 (d, *J* = 19.5 Hz), 133.9 (d, *J* = 20.1 Hz), 133.7 (d, *J* = 19.5 Hz), 132.0, 131.7, 130.9, 129.6, 128.3, 128.2, 128.2, 128.1, 128.1, 128.0, 127.2, 127.1 (C-Ar), 103.6 (C-2), 81.1 (C-4), 76.5 (C-3), 74.1 (C-5), 73.8 (*C*H_2Ph), 61.7 (C-6), 57.6, 57.3 (OCH_3); ^{31}P NMR (162 MHz, $CDCl_3$) δ = -5.76; HRMS (ESI-TOF) m/z $[M+H]^+$: calcd for $C_{34}H_{35}NO_5P$: 568.22474, found: 568.22450; Anal calcd for $C_{34}H_{34}NO_5P$: C 71.94, H 6.04, N 2.47, found: C 71.71, H 6.36, N 2.45.

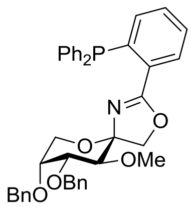

(5*R*,8*R*,9*R*,10*S*)-8,9-Bis(benzyloxy)-2-(2-(diphenylphosphanyl)phenyl)-10-methoxy-3,6-dioxa-1-azaspiro[4.5]dec-1-ene (**5d**): Prepared from **10d** (198 mg, 0.368 mmol) with CuI (8.7 mg, 0.046 mmol), *N*,*N*'-dimethylethylenediamine (37 µL, 0.32 mmol), $HPPh_2$ (120 µL, 0.692 mmol) and Cs_2CO_3 (450 mg, 1.38 mmol), yield after column chromatography (PE/EtOAc, 4/1 + 2% Et_3N): 190 mg, 80%; R_f = 0.45 (PE/EtOAc, 4/1 + 2% Et_3N); $[\alpha]^{20}_D$ -108.8 (c = 1.0, $CHCl_3$); mp = 65 °C (CH_2Cl_2); ^{1}H NMR (400 MHz, $CDCl_3$) δ = 7.95 – 7.92 (m, 1 H, H-Ar), 7.35 – 7.04 (m, 22 H, H-Ar), 6.97 – 6.88 (m, 2 H, H-Ar), 6.81 – 6.79 (m, 1 H, H-Ar), 4.67 – 4.56 (m, 2 H, CH_2Ph), 4.43 (s, 2 H, CH_2Ph), 4.22 (d, *J* = 9.5 Hz, 1 H, H-1a), 4.14 (d, *J* = 9.5 Hz, 1 H, H-1b), 3.59 (d, *J* = 9.7 Hz, 1 H, H-3), 3.54 (dd, *J* = 2.9, 9.7 Hz, 1 H, H-4), 3.47 (s, 3 H, OCH_3), 3.46 –

3.44 (m, 1 H, H-5), 3.41 (dd, J = 1.7, 12.5 Hz, 1 H, H-6a), 3.18 (d, J = 12.5 Hz, 1 H, H-6b); $^{13}C\{^{1}H\}$ NMR (101 MHz , $CDCl_3$) δ = 164.4 (OCN), 139.5 (d, J = 9.9 Hz), 139.2 (d, J = 10.5 Hz), 139.0 (d, J = 10.3 Hz), 134.7 (d, J = 19.4 Hz), 133.5 (d, J = 19.5 Hz), 131.3 (d, J = 19.4 Hz), , 129.9, 128.3, 128.2, 128.2, 128.1, 128.1, 128.1, 128.0, 127.8, 127.5, 127.5, 127.4 (C-Ar), 103.8 (C-2), 79.8 (C-4), 79.7 (C-3), 74.7 (C-5), 73.9 (C-1), 72.0, 71.6 (CH_2Ph), 63.0 (C-6), 61.3 (OCH_3); ^{31}P NMR (162 MHz, $CDCl_3$) δ = -5.54; HRMS (ESI-TOF) m/z $[M+H]^+$: calcd for $C_{40}H_{39}NO_5P$: 644.25604, found: 644.25645; Anal calcd for $C_{40}H_{38}NO_5P$: C 74.63, H 5.95, N 2.18, found: C 74.73, H 6.19, N 2.25.

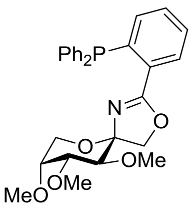

(5*R*,8*R*,9*R*,10*S*)-2-(2-(Diphenylphosphanyl)phenyl)-8,9,10-trimethoxy-3,6-dioxa-1-azaspiro[4.5]dec-1-ene (**5e**): Prepared from **10e** (300 mg, 0.779 mmol) with CuI (18.6 mg, 0.098 mmol), *N*,*N*'-dimethylethylenediamine (70 μL, 0.68 mmol), $HPPh_2$ (255 μL, 1.47 mmol) and Cs_2CO_3 (953 mg, 2.93 mmol), yield after column chromatography (PE/EtOAc, 7/1 + 2% Et_3N): 310 mg, 81%; R_f = 0.25 (PE/EtOAc, 4/1 + 2% Et_3N); $[\alpha]^{20}_D$ -168.1 (c = 1.0, $CHCl_3$); mp = 68 °C (CH_2Cl_2); ^{1}H NMR (400 MHz, $CDCl_3$) δ = 7.93 – 7.91 (m, 1 H, H-Ar), 7.32 – 7.29 (m, 1 H, H-Ar), 7.27 – 7.19 (m, 7 H, H-Ar), 7.19 – 7.09 (m, 4 H, H-Ar), 6.81 – 6.78 (m, 1 H, H-Ar), 4.19 (d, J = 9.4 Hz, 1 H, H-1a), 4.12 (d, J = 9.4 Hz, 1 H, H-1b), 3.58 (dd, J = 1.7, 12.7 Hz, 1 H, H-6a), 3.46 – 3.36 (m, 5 H, H-3, H-6b, OCH_3), 3.35 (s, 3 H, OCH_3), 3.32 – 3.28 (m, 1 H, H-5), 3.26 (s, 3 H, OCH_3), 3.14 (dd, J = 3.4, 9.7 Hz, 1 H, H-4); $^{13}C\{^{1}H\}$ NMR (101 MHz , $CDCl_3$) δ = 164.6 (OCN), 139.3 (d, J = 11.7 Hz), 139.2 (d, J = 14.7 Hz), 138.9 (d, J = 13.9 Hz), 133.9 (d, J = 33.0 Hz), 133.8 (d, J = 7.8 Hz), 131.6 (d, J = 20.5 Hz), 131.0, 129.8, 128.3, 128.2, 128.2, 128.1 (C-Ar), 103.5 (C-2), 81.0 (C-4), 79.2 (C-3), 76.8 (C-5), 73.9 (C-1), 61.4 (C-6), 61.0, 57.5, 57.4 (OCH_3); ^{31}P NMR (162 MHz, $CDCl_3$) δ = -5.58; HRMS (ESI-TOF) m/z $[M+H]^+$: calcd for $C_{28}H_{31}NO_5P$: 492.19344, found: 492.19378; Anal calcd for $C_{28}H_{30}NO_5P$: C 68.42, H 6.15, N 2.85, found: C 68.32, H 6.23, N 2.91.

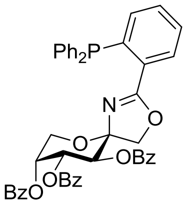

(5*R*,8*R*,9*R*,10*S*)-2-(2-(Diphenylphosphanyl)phenyl)-3,6-dioxa-1-azaspiro [4.5]dec-1-ene-8,9,10-triyl tribenzoate (**5f**): Prepared from **10f** (250 mg, 0.381 mmol) with CuI (9.1 mg, 0.048 mmol), *N,N*'-dimethylethylenediamine (36 μL, 0.33 mmol), $HPPh_2$ (125 μL, 0.716 mmol) and Cs_2CO_3 (466 mg, 1.43 mmol), yield after column chromatography (PE/EtOAc, 7/1 + 2% Et_3N): 199 mg, 69%; R_f = 0.63 (toluene/EtOAc, 4/1 + 2.5% Et_3N); $[\alpha]^{20}_D$ -178.3 (c=1.0, $CHCl_3$); mp = 107°C (CH_2Cl_2); ^1H NMR (400 MHz, $CDCl_3$) δ = 8.01 – 7.95 (m, 4 H, H-Ar), 7.91 – 7.89 (m, 1 H, H-Ar), 7.76 – 7.71 (m, 2 H, H-Ar), 7.62 – 7.55 (m, 4 H, H-Ar), 7.51 – 7.04 (m, 17 H, H-Ar), 6.94 – 6.90 (m, 1 H, H-Ar), 5.91 (d, *J* = 10.1 Hz, 1 H, H-3), 5.74 (dd, *J* = 3.5, 10.1 Hz, 1 H, H-4), 5.50 – 5.44 (m, 1 H, H-5), 4.31 – 4.19 (m, 2 H, H-1a, H-1b), 3.58 (dd, *J* = 1.7, 12.8 Hz, 1 H, H-6a), 3.33 (d, *J* = 12.8 Hz, 1 H, H-6b); $^{13}C\{^1H\}$ NMR (101 MHz , $CDCl_3$) δ = 166.1, 165.8, 165.3 (CO), 165.2 (OCN), 140.4 (d, *J* = 27.9 Hz), 139.1 (d, *J* = 38.9 Hz), 138.7 (d, *J* = 33.0 Hz), 134.5 (d, *J* = 21.3 Hz), 133.8 , 133.5 (d, *J* = 15.4 Hz), 133.3, 133.2 (d, *J* = 19.8 Hz), 132.9, 132.1, 131.4, 130.8, 130.2, 129.8, 129.6, 129.4, 129.0, 128.5, 128.4, 128.4, 128.3, 128.2, 128.1, 127.6, 125.3 (C-Ar), 103.0 (C-2), 73.9 (C-1), 71.2 (C-3), 70.4 (C-5), 69.7 (C-4), 63.1 (C-6); ^{31}P NMR (162 MHz, $CDCl_3$) δ = -6.18; HRMS (ESI-TOF) m/z $[M+H]^+$: calcd for $C_{46}H_{37}NO_8P$: 762.22513, found: 762.22575; Anal calcd for $C_{46}H_{36}NO_8P$: C 72.53, H 4.76, N 1.84, found: C 72.15, H 4.71, N 1.76.

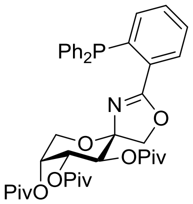

(5*R*,8*R*,9*R*,10*S*)-2-(2-(diphenylphosphanyl)phenyl)-3,6-dioxa-1-azaspiro [4.5]dec-1-ene-8,9,10-triyl tris(2,2-dimethylpropanoate) (**5g**): Prepared from **10g** (265 mg, 0.444 mmol) with CuI (10.6 mg, 0.056 mmol), *N,N*'-dimethylethylenediamine (41 μL, 0.39 mmol), $HPPh_2$ (145 μL, 0.835 mmol) and Cs_2CO_3 (542 mg, 1.67 mmol), yield after column chromatography (PE/EtOAc, 15/1 + 2% Et_3N): 206 mg, 66%; R_f = 0.42 (PE/EtOAc, 10/1 + 2% Et_3N); $[\alpha]^{20}_D$ -95.8 (c = 1.0, $CHCl_3$); mp = 184 °C ($CHCl_3$); ^1H NMR (400 MHz, $CDCl_3$) δ = 8.02 – 7.94 (m, 1 H, H-Ar), 7.46 – 7.28 (m, 10 H,

H-Ar), 7.25 – 7.15 (m, 2 H, H-Ar), 6.98 – 6.90 (m, 1 H, H-Ar), 5.44 (d, J = 10.1 Hz, 1 H, H-3), 5.32 (dd, J = 3.3, 10.1 Hz, 1 H, H-4), 5.20 – 5.12 (m, 1 H, H-5), 4.14 (d, J = 5.1 Hz, 2 H, H-1a, H-1b), 3.35 (dd, J = 1.7, 13.0 Hz, 1 H, H-6a), 3.28 (dd, J = 0.6, 13.0 Hz, H-6b), 1.25 (s, 9 H, $C(CH_3)_3$), 1.14 (s, 9 H, $C(CH_3)_3$), 1.12 (s, 9 H, $C(CH_3)_3$); $^{13}C\{^1H\}$ NMR (101 MHz , $CDCl_3$) δ = 177.8, 177.4, 176.9 (CO), 164.6 (OCN), 140.4 (d, J = 28.6 Hz), 138.8 (d, J = 46.2 Hz), 138.6 (d, J = 41.1 Hz), 134.9 (d, J = 20.5 Hz), 134.1 (d, J = 20.5 Hz), 133.6 (d, J = 19.8 Hz), 130.8, 130.6, 129.6, 128.5, 128.3, 128.3, 128.1, 128.0 (C-Ar), 103.0 (C-2), 73.8 (C-1), 70.2 (C-4), 69.2 (C-5), 68.8 (C-3), 63.4 (C-6), 39.0, 38.9, 38.7 ($C(CH_3)_3$), 27.2, 27.0, 27.0 ($C(CH_3)_3$); ^{31}P NMR (162 MHz, $CDCl_3$) δ = -6.52; HRMS (ESI-TOF) m/z $[M+H]^+$: calcd for $C_{40}H_{49}NO_8P$: 702.31903, found: 702.31905; Anal calcd for $C_{40}H_{48}NO_8P$: C 68.46, H 6.89, N 2.00, found: C 68.12, H 6.98, N 1.96.

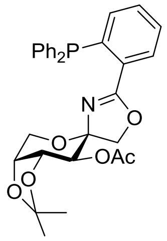

(3a'*R*,4*R*,7'*S*,7a'*R*)-2-(2-(Diphenylphosphanyl)phenyl)-2',2'-dimethyl-3a',4',7',7a'-tetrahydro-5H-spiro[oxazole-4,6'-[1,3]dioxolo[4,5-c]pyran]-7'-yl acetate (**5h**): Prepared from **10h** (190 mg, 0.446 mmol) with CuI (10.6 mg, 0.103 mmol), *N,N*'-dimethylethylenediamine (42 µL, 0.39 mmol), $HPPh_2$ (156 µL, 0.838 mmol) and Cs_2CO_3 (545 mg, 1.67 mmol), yield after column chromatography (PE/EtOAc, 7/1 + 2% Et_3N): 143 mg, 60%; R_f = 0.41 (PE/EtOAc, 4/1 + 2% Et_3N); $[\alpha]^{20}_D$ -156.4 (c = 1.0, $CHCl_3$); mp = 75 °C ($CHCl_3$); ^1H NMR (400 MHz, $CDCl_3$) δ = 8.01 – 7.99 (m, 1 H, H-Ar), 7.46 – 7.28 (m, 8 H, H-Ar), 7.26 – 7.16 (m, 4 H, H-Ar), 6.93 – 6.93 (m, 1 H, H-Ar), 5.13 (d, J = 8.1 Hz, 1 H, H-3), 4.20 – 4.06 (m, 2 H, H-1a, H-1b), 3.98 – 3.83 (m, 3 H, H-5, H-6a, H-6b), 3.67 (dd, J = 5.0, 7.9 Hz, 1 H, H-4), 2.05 (s, 3 H, CH_3), 1.53 (s, 3 H, $C(CH_3)_2$), 1.33 (s, 3 H, $C(CH_3)_2$); $^{13}C\{^1H\}$ NMR (101 MHz , $CDCl_3$) δ = 170.8 (CO), 164.9 (OCN), 139.6 (d, J = 27.9 Hz), 139.1 (d, J = 16.1 Hz), 138.9 (d, J = 15.4 Hz), 134.8 (d, J = 19.8 Hz), 133.6 (d, J = 19.0 Hz), 133.4 (d, J = 19.8 Hz), 131.2, 131.1, 130.9, 129.7, 128.4, 128.4, 128.3, 128.1 (C-Ar), 109.0 ($C(CH_3)_2$), 101.3 (C-2), 75.2 (C-4), 74.4 (C-5), 73.4 (C-1),

72.7 (C-3), 61.8 (C-6), 27.7, 26.3 (C(*C*H_3)$_2$), 21.0 (CH_3); ^{31}P NMR (162 MHz, $CDCl_3$) δ = -4.89; HRMS (ESI-TOF) m/z $[M+H]^+$: calcd for $C_{30}H_{31}NO_6P$: 532.18835, found: 532.18858; Anal calcd for $C_{30}H_{30}NO_6P$: C 67.79, H 5.69, N 2.64, found: C 67.66, H 5.83, N 2.55.

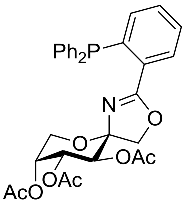

(5*R*,8*R*,9*R*,10*S*)-2-(2-(Diphenylphosphanyl)phenyl)-3,6-dioxa-1-azaspiro [4.5]dec-1-ene-8,9,10-triyl triacetate (**5i**): Prepared from **10i** (300 mg, 0.638 mmol) with CuI (15.2 mg, 0.080 mmol), *N*,*N*'-dimethylethylenediamine (60 μL, 0.56 mmol), $HPPh_2$ (209 μL, 1.20 mmol) and Cs_2CO_3 (778 mg, 2.39 mmol), yield after column chromatography (PE/EtOAc, 5/1 + 2% Et_3N): 240 mg, 65%; R_f = 0.35 (PE/EtOAc, 4/1 + 2% Et_3N); $[\alpha]^{20}_D$ -114.0 (c = 1.0, $CHCl_3$); mp = 89 °C (CH_2Cl_2); ^{1}H NMR (400 MHz, $CDCl_3$) δ = 8.01 – 7.98 (m, 1 H, H-Ar), 7.45 – 7.27 (m, 10 H, H-Ar), 7.25 – 7.18 (m, 2 H, H-Ar, H-Ar), 6.95 – 6.93 (m, 1 H, H-Ar), 5.42 (d, *J* = 10.3 Hz, 1 H, H-3), 5.30 (dd, *J* = 3.4, 10.2 Hz, 1 H, H-4), 5.21 – 5.17 (m, 1 H, H-5), 4.16 (s, 2 H, H-1a, H-1b), 3.42 – 3.30 (m, 2 H, H-6a, H-6b), 2.12 (s, 3 H, CH_3), 2.02 (s, 3 H, CH_3), 1.98 (s, 3 H, CH_3); ^{13}C{^{1}H} NMR (101 MHz , $CDCl_3$) δ = 170.8, 170.5, 169.9 (CO), 165.5 (OCN), 140.4 (d, *J* = 28.6 Hz), 138.8 (d, *J* = 8.8 Hz), 138.7 (d, *J* = 13.2 Hz), 134.3 (d, *J* = 19.8 Hz), 133.6 (d, *J* = 19.8 Hz), 133.5 (d, *J* = 21.6 Hz), 131.5, 131.0, 130.8, 130.0, 128.6, 128.5, 128.5, 128.5, 128.3, 128.2 (C-Ar), 102.8 (C-2), 73.7 (C-1), 70.4 (C-4), 69.5 (C-5), 68.9 (C-3), 63.2 (C-6), 21.1, 21.0, 20.9 (CH_3); ^{31}P NMR (162 MHz, $CDCl_3$) δ = -5.77; HRMS (ESI-TOF) m/z $[M+H]^+$: calcd for $C_{31}H_{31}NO_8P$: 576.17818, found: 576.17838; Anal calcd for $C_{31}H_{30}NO_8P$: C 64.69, H 5.25, N 2.43, found: C 64.57, H 5.40, N 2.50.

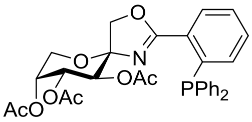

(5*S*,8*R*,9*R*,10*S*)-2-(2-(Diphenylphosphanyl)phenyl)-3,6-dioxa-1-aza-spiro[4.5]dec-1-ene-8,9,10-triyl triacetate (**14a**): Prepared from **11i** (315 mg, 0.670 mmol) with CuI (16.0 mg, 0.082 mmol), *N*,*N*'-dimethylethylenediamine (63 μL, 0.59 mmol), $HPPh_2$ (220 μL, 1.26 mmol) and Cs_2CO_3 (819 mg, 2.51 mmol), yield after

column chromatography (PE/EtOAc, 5/1 + 2% Et_3N): 136 mg, 35%; R_f = 0.38 (PE/EtOAc, 2/1 + 2% Et_3N); $[\alpha]^{20}_D$ -72.6 (c=1.0, $CHCl_3$); mp = 63 °C (CH_2Cl_2); ^{1}H NMR (400 MHz, $CDCl_3$) δ = 7.88 – 7.80 (m, 1 H, H-Ar), 7.29 – 7.13 (m, 12 H, H-Ar), 6.84 – 6.81 (m, 1 H, H-Ar), 4.96 – 4.93 (m, 1 H, H-Ar), 4.90 (dd, *J* = 3.9, 6.2 Hz, 1 H, H-4), 4.38 (d, *J* = 6.2 Hz, 1 H, H-3), 4.09 (d, *J* = 11.5 Hz, 1 H, H-1a), 3.78 (d, *J* = 11.5 Hz, 1 H, H-1b), 3.68 (dd, *J* = 3.3, 13.1 Hz, 1 H, H-6a), 3.33 (dd, *J* = 2.6, 13.1 Hz, 1 H, H-6b), 2.01 (s, 3 H, CH_3), 2.00 (s, 3 H, CH_3) 1.98 (s, 3 H, CH_3); $^{13}C\{^1H\}$ NMR (101MHz , $CDCl_3$) δ = 170.3, 170.0, 169.7 (CO), 165.9 (OCN), 139.6 (d, *J* = 27.9 Hz), 137.6 (d, *J* = 11.7 Hz), 137.5 (d, *J* = 11.7 Hz), 134.4 (d, *J* = 13.9 Hz), 133.8 (d, *J* = 27.9 Hz), 133.6 (d, *J* = 20.5 Hz), 131.6, 130.5, 130.3, 128.8, 128.7, 128.7, 128.7, 128.6, 128.5, 128.2 (H-Ar), 100.5 (C-2), 76.2 (C-3), 70.5 (C-4), 66.1 (C-1), 66.0 (C-5), 62.2 (C-6), 20.8, 20.8, 20.8 (CH_3); ^{31}P NMR (162 MHz, $CDCl_3$) δ = -6.22; HRMS (ESI-TOF) m/z $[M+H]^+$: calcd for $C_{31}H_{31}NO_8P$: 576.17818, found: 576.17854; Anal calcd for $C_{31}H_{30}NO_8P$: C 64.69, H 5.25, N 2.43, found: C 64.78, H 5.25, N 2.43.

1.6 General procedure for the Tsuji–Trost reaction

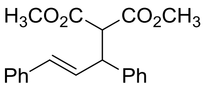

Small scale experiments: 0.5 mL solution of the PHOX ligand (13.2 mM in dry CH_2Cl_2) and 0.5 mL solution of $[PdCl(C_3H_5)]_2$ (6.6 mM in dry CH_2Cl_2) were mixed and stirred at room temperature for 20 min. The solvent was evaporated in vacuo. A solution of dimethyl malonate (20 µL, 174 µmol), *rac*-diphenylallyl acetate (14.6 mg, 58 µmol), KOAc (0.3 mg, 3 µmol) and *N,O*-bis(trimethylsilyl)acetamide (43 µL, 174 µmol) in 1 mL dry solvent was added to the residue. The reaction mixture was stirred at room temperature for 24 h. The reaction was quenched with saturated NH_4Cl solution, the aqueous layer was extracted with CH_2Cl_2 (3 × 2 mL). The combined organic layers were dried over Na_2SO_4, filtered through a pad of silica and concentrated in vacuo. Conversion was determined by ^{1}H NMR in $CDCl_3$. Enantiomeric ratio was determined by HPLC (*n*-hexane/2-propanol (9/1); 1.6 mL/min), t_R = 5.2 min for the (*R*)-**16**, t_R = 6.8 min for (*S*)-**16**.

Experiments with isolated yield: A solution of **5b** (31.7 mg, 0.044 mmol) or **5i** (25.3 mg, 0.044 mmol) and $[PdCl(C_3H_5)]_2$ (7.3 mg, 0.02 mmol) in 3 mL solvent was stirred at room temperature for 20 min. Dimethyl malonate (138 μL, 1.20 mmol), KOAc (2.0 mg, 0.02 mmol), *N,O*-bis(trimethylsilyl)acetamide (294 μL, 1.20 mmol) and *rac*-diphenylallyl acetate (100 mg, 0.40 mmol in 4 mL solvent) were added and the mixture was stirred at room temperature for 24 h. The reaction was quenched with saturated NH_4Cl solution (3 mL) and the aqueous layer was extracted with CH_2Cl_2 (3 × 5 mL). The combined organic layers were dried over Na_2SO_4, filtered and concentrated. Column chromatography (PE/EtOAc, 6/1) of the residue gave **16** as a colorless oil.

2 Optimization of reaction conditions for the Ritter reaction

Table S1. Ritter reaction with different conditions.

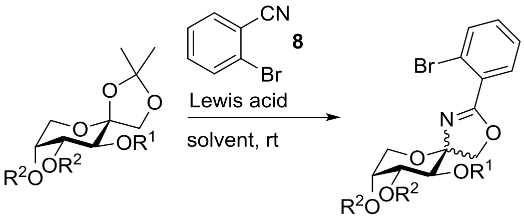

7 10/11

entry	reactant	equiv **8**	solvent	Lewis-acid (equiv)	time	yield (α+β)
1	**7e**	15	toluene	TMSOTf (1.0)	75 min	37%
2	**7e**	12	toluene	TMSOTf (1.0)	90 min	31%
3	**7e**	5	toluene	TMSOTf (1.0)	90 min	13%
4	**7e**[a]	5	toluene	TMSOTf (1.1)	90 min	21%
5	**7e**[b]	5	toluene	TMSOTf (1.1)	90 min	10%
6	**7l**	15	toluene	TMSOTf (1.0)	4 h	36%
7	**7m**	15	toluene	TMSOTf (1.0)	18 h	23%
8	**7e**	15	CH_2Cl_2	$Zn(OTf)_2$ (1.0)	30 h[c]	n.r.[d]
9	**7e**	15	CH_2Cl_2	$BF_3 \cdot OEt_2$ (1.0)	40 min	58%

[a] Slow addition of **7e** to a solution of **8** and TMSOTf in toluene, [b] Slow addition of **7e** and TMSOTf to a solution of **8** in toluene, [c] 25 h rt, then 5 h reflux, [d] no reaction.

3 Ullmann coupling with longer reaction times

Table S2. Yields of Ullmann coupling with **10f** and **10i** with longer reaction times.

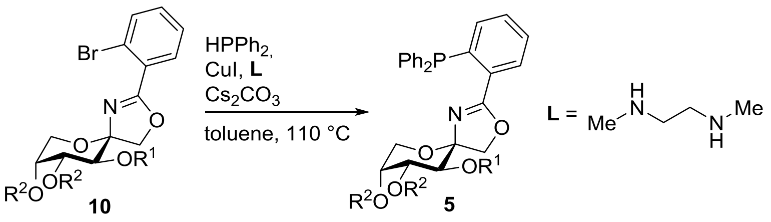

entry	reactant	time	yield
1	**10f**	19 h	37%
2	**10i**	18 h	0%[a]

[a] HRMS (ESI-TOF) indicated formation of: 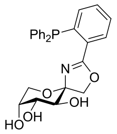

, m/z $[M+H]^+$: calcd for $C_{25}H_{25}O_5$: 450.14649, found: 450.14684.

4 Crystal data for 10j

Crystals for X-ray crystallography were prepared by covering a saturated solution of **10j** in 2-propanol with *n*-heptane at room temperature. Single crystals were selected, coated with Parabar 10312 (previously known as Paratone N, Hampton Research) and fixed on a microloop. Data were collected on a Bruker APEX DUO instrument equipped with an IμS microfocus sealed tube and QUAZAR optics for Mo K_{α} radiation (λ = 0.71073 Å). The Data collection strategy was determined using COSMO [10] employing ω- and ϕ scans. Raw data were processed using APEX [11] and SAINT [12], corrections for absorption effects were applied using SADABS [13]. The structure was solved by direct methods and refined against all data by full-matrix least-squares methods on F^2 using SHELXTL [14] and Shelxle [15].

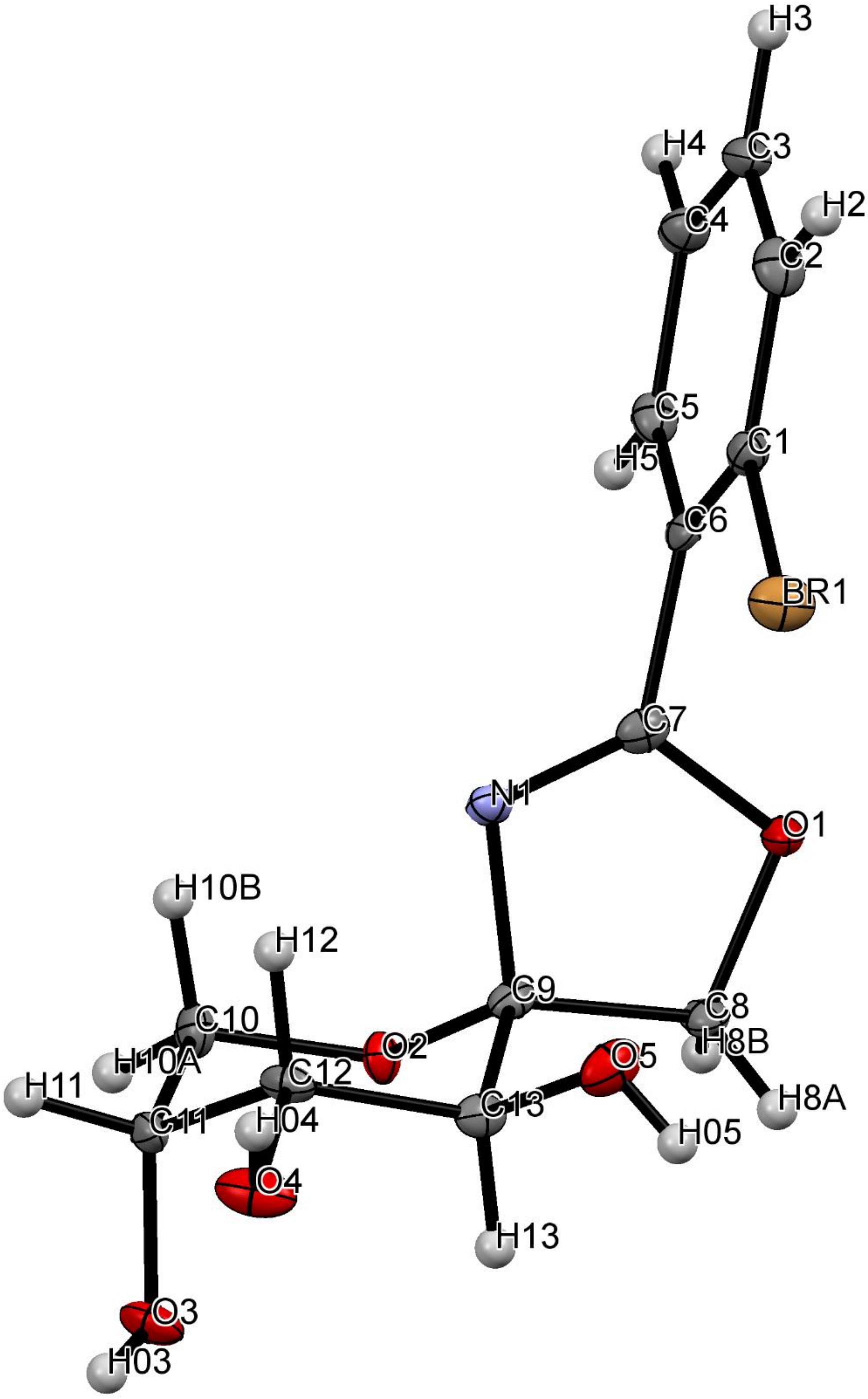

Figure S1. Molecular structure of **10j**.

Table S3. Crystal data and structure refinement for **10j**.

Identification code	mo_MI126_0m	
Empirical formula	C13 H14 Br N O5	
Formula weight	344.16	
Temperature	100(2) K	
Wavelength	0.71073 Å	
Crystal system	Orthorhombic	
Space group	P 21 21 21	
Unit cell dimensions	a = 6.1808(9) Å	a= 90°.
	b = 7.4746(11) Å	b= 90°.
	c = 28.259(4) Å	g = 90°.
Volume	1305.6(3) Å^3	
Z	4	
Density (calculated)	1.751 Mg/m^3	
Absorption coefficient	3.168 mm^{-1}	
F(000)	696	
Crystal size	0.347 x 0.081 x 0.033 mm^3	
Theta range for data collection	1.441 to 28.685°.	
Index ranges	-8<=h<=8, -10<=k<=10, -30<=l<=37	
Reflections collected	10618	
Independent reflections	3354 [R(int) = 0.0615]	

Completeness to theta = 25.242°	99.9%
Refinement method	Full-matrix least-squares on F^2
Data / restraints / parameters	3354 / 0 / 193
Goodness-of-fit on F^2	0.952
Final R indices [I>2sigma(I)]	R1 = 0.0355, wR2 = 0.0618
R indices (all data)	R1 = 0.0463, wR2 = 0.0649
Absolute structure parameter	0.001(9)
Extinction coefficient	n/a
Largest diff. peak and hole	0.646 and -0.438 e.Å^{-3}

Table S4. Atomic coordinates (x 10^4) and equivalent isotropic displacement parameters (Å^2 × 10^3) for **10j.** U(eq) is defined as one third of the trace of the orthogonalized U^{ij} tensor.

	x	y	z	U(eq)
C(1)	6251(6)	10061(5)	5411(1)	12(1)
C(2)	5805(7)	10710(5)	4962(1)	16(1)
C(3)	7330(7)	10431(6)	4609(1)	16(1)
C(4)	9235(7)	9525(5)	4699(1)	15(1)
C(5)	9643(6)	8893(5)	5154(1)	13(1)
C(6)	8141(6)	9134(5)	5514(1)	10(1)

C(7)	8566(6)	8373(5)	5993(1)	12(1)
C(8)	10096(6)	8369(5)	6709(1)	12(1)
C(9)	8835(7)	6633(5)	6617(1)	10(1)
C(10)	9272(8)	3467(5)	6528(1)	13(1)
C(11)	7335(7)	3036(5)	6838(1)	12(1)
C(12)	5775(6)	4618(5)	6835(1)	12(1)
C(13)	6976(6)	6320(5)	6967(1)	11(1)
N(1)	8038(5)	6816(4)	6130(1)	11(1)
O(1)	9664(4)	9455(4)	6292(1)	12(1)
O(2)	10284(4)	5140(3)	6650(1)	11(1)
O(3)	8150(5)	2677(4)	7302(1)	16(1)
O(4)	4043(5)	4291(4)	7163(1)	19(1)
O(5)	5519(5)	7799(4)	6958(1)	18(1)
Br(1)	4126(1)	10349(1)	5892(1)	17(1)

Table S5. Bond lengths [Å] and angles [°] for **10j**.

C(1)-C(2)	1.385(5)
C(1)-C(6)	1.389(5)
C(1)-Br(1)	1.902(4)
C(2)-C(3)	1.389(6)
C(2)-H(2)	0.9500
C(3)-C(4)	1.382(6)
C(3)-H(3)	0.9500
C(4)-C(5)	1.392(5)
C(4)-H(4)	0.9500
C(5)-C(6)	1.389(5)
C(5)-H(5)	0.9500
C(6)-C(7)	1.493(5)
C(7)-N(1)	1.269(5)
C(7)-O(1)	1.353(4)
C(8)-O(1)	1.455(4)
C(8)-C(9)	1.536(5)
C(8)-H(8A)	0.9900
C(8)-H(8B)	0.9900
C(9)-O(2)	1.434(4)

C(9)-N(1)	1.467(5)
C(9)-C(13)	1.534(5)
C(10)-O(2)	1.440(4)
C(10)-C(11)	1.518(6)
C(10)-H(10A)	0.9900
C(10)-H(10B)	0.9900
C(11)-O(3)	1.430(5)
C(11)-C(12)	1.525(6)
C(11)-H(11)	1.0000
C(12)-O(4)	1.438(4)
C(12)-C(13)	1.520(6)
C(12)-H(12)	1.0000
C(13)-O(5)	1.426(5)
C(13)-H(13)	1.0000
O(3)-H(03)	0.67(4)
O(4)-H(04)	0.74(4)
O(5)-H(05)	0.74(4)
C(2)-C(1)-C(6)	122.2(4)
C(2)-C(1)-Br(1)	118.5(3)
C(6)-C(1)-Br(1)	119.2(3)

C(1)-C(2)-C(3)	118.1(4)
C(1)-C(2)-H(2)	121.0
C(3)-C(2)-H(2)	121.0
C(4)-C(3)-C(2)	121.2(4)
C(4)-C(3)-H(3)	119.4
C(2)-C(3)-H(3)	119.4
C(3)-C(4)-C(5)	119.4(4)
C(3)-C(4)-H(4)	120.3
C(5)-C(4)-H(4)	120.3
C(6)-C(5)-C(4)	120.7(4)
C(6)-C(5)-H(5)	119.7
C(4)-C(5)-H(5)	119.7
C(5)-C(6)-C(1)	118.3(4)
C(5)-C(6)-C(7)	119.8(4)
C(1)-C(6)-C(7)	121.9(3)
N(1)-C(7)-O(1)	119.1(3)
N(1)-C(7)-C(6)	125.6(4)
O(1)-C(7)-C(6)	115.3(3)
O(1)-C(8)-C(9)	104.0(3)
O(1)-C(8)-H(8A)	111.0

C(9)-C(8)-H(8A)	111.0
O(1)-C(8)-H(8B)	111.0
C(9)-C(8)-H(8B)	111.0
H(8A)-C(8)-H(8B)	109.0
O(2)-C(9)-N(1)	110.1(3)
O(2)-C(9)-C(13)	107.9(3)
N(1)-C(9)-C(13)	111.5(3)
O(2)-C(9)-C(8)	109.2(3)
N(1)-C(9)-C(8)	104.5(3)
C(13)-C(9)-C(8)	113.6(3)
O(2)-C(10)-C(11)	112.8(3)
O(2)-C(10)-H(10A)	109.0
C(11)-C(10)-H(10A)	109.0
O(2)-C(10)-H(10B)	109.0
C(11)-C(10)-H(10B)	109.0
H(10A)-C(10)-H(10B)	107.8
O(3)-C(11)-C(10)	107.0(3)
O(3)-C(11)-C(12)	111.9(3)
C(10)-C(11)-C(12)	109.3(3)
O(3)-C(11)-H(11)	109.5

C(10)-C(11)-H(11)	109.5
C(12)-C(11)-H(11)	109.5
O(4)-C(12)-C(13)	110.3(3)
O(4)-C(12)-C(11)	109.6(3)
C(13)-C(12)-C(11)	109.8(3)
O(4)-C(12)-H(12)	109.0
C(13)-C(12)-H(12)	109.0
C(11)-C(12)-H(12)	109.0
O(5)-C(13)-C(12)	109.7(3)
O(5)-C(13)-C(9)	110.1(3)
C(12)-C(13)-C(9)	109.6(3)
O(5)-C(13)-H(13)	109.2
C(12)-C(13)-H(13)	109.2
C(9)-C(13)-H(13)	109.2
C(7)-N(1)-C(9)	106.6(3)
C(7)-O(1)-C(8)	105.3(3)
C(9)-O(2)-C(10)	112.9(3)
C(11)-O(3)-H(03)	112(4)
C(12)-O(4)-H(04)	108(3)
C(13)-O(5)-H(05)	108(4)

Table S6. Anisotropic displacement parameters ($Å^2 \times 10^3$) for **10j**. The anisotropic displacement factor exponent takes the form: $-2p^2[h^2 a^{*2}U^{11} + ... + 2 h k a^* b^* U^{12}]$.

	U^{11}	U^{22}	U^{33}	U^{23}	U^{13}	U^{12}
C(1)	14(2)	11(2)	10(2)	-1(2)	2(2)	-2(2)
C(2)	12(2)	14(2)	21(2)	0(2)	-2(2)	3(2)
C(3)	24(2)	14(2)	10(2)	2(2)	-3(2)	-3(2)
C(4)	17(2)	15(2)	12(2)	-1(2)	3(2)	-3(2)
C(5)	12(2)	11(2)	17(2)	0(2)	-3(2)	1(2)
C(6)	13(2)	10(2)	9(2)	-1(2)	-2(2)	-4(2)
C(7)	9(2)	16(2)	10(2)	-2(2)	2(2)	2(2)
C(8)	12(2)	14(2)	10(2)	1(2)	-4(2)	-2(2)
C(9)	9(2)	12(2)	9(2)	-2(2)	-2(2)	3(2)
C(10)	14(2)	10(2)	16(2)	-1(2)	-1(2)	-3(2)
C(11)	16(2)	9(2)	10(2)	2(2)	-2(2)	-4(2)
C(12)	8(2)	17(2)	9(2)	4(2)	0(2)	-3(2)
C(13)	8(2)	14(2)	10(2)	0(2)	0(2)	5(2)
N(1)	12(2)	13(2)	8(2)	0(2)	1(1)	-1(2)
O(1)	17(2)	10(1)	10(1)	1(1)	-4(1)	-2(1)
O(2)	10(1)	8(1)	15(1)	-1(1)	0(1)	3(1)

O(3)	13(2)	21(2)	14(2)	8(1)	0(1)	-3(1)
O(4)	7(1)	35(2)	16(2)	9(1)	-1(1)	-2(2)
O(5)	14(2)	22(2)	16(2)	-6(1)	-1(1)	7(1)
Br(1)	12(1)	23(1)	17(1)	1(1)	2(1)	4(1)

Table S7. Hydrogen coordinates (x 10^4) and isotropic displacement parameters (Å^2 × 10 ^{3}).

for **10j**.

	x	y	z	U(eq)
H(2)	4494	11328	4898	19
H(3)	7059	10871	4299	19
H(4)	10257	9335	4453	18
H(5)	10964	8290	5219	16
H(8A)	9573	8970	6999	14
H(8B)	11664	8126	6742	14
H(10A)	10349	2493	6557	16
H(10B)	8798	3515	6193	16
H(11)	6585	1949	6713	14
H(12)	5158	4754	6510	14
H(13)	7582	6191	7293	13

H(03)	7430(70)	2230(60)	7436(14)	5(13)
H(04)	3010(70)	4480(70)	7042(14)	16(13)
H(05)	5680(90)	8310(60)	7180(15)	24(15)

5 NMR spectra

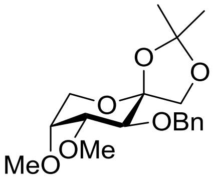

7g

^{1}H NMR (400 MHz, $CDCl_3$)

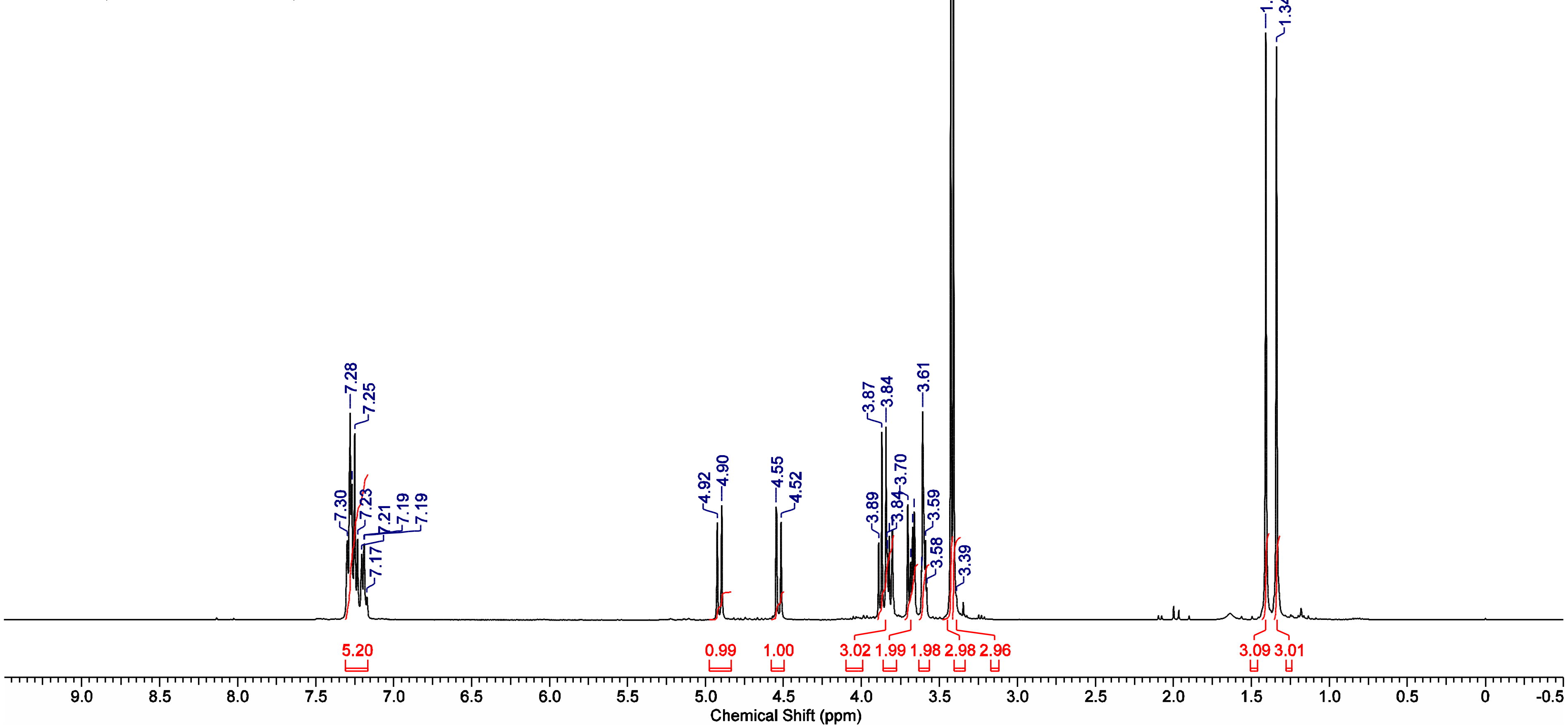

^{13}C NMR (101 MHz, $CDCl_3$)

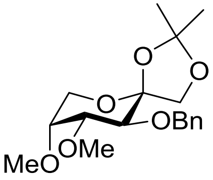

7g

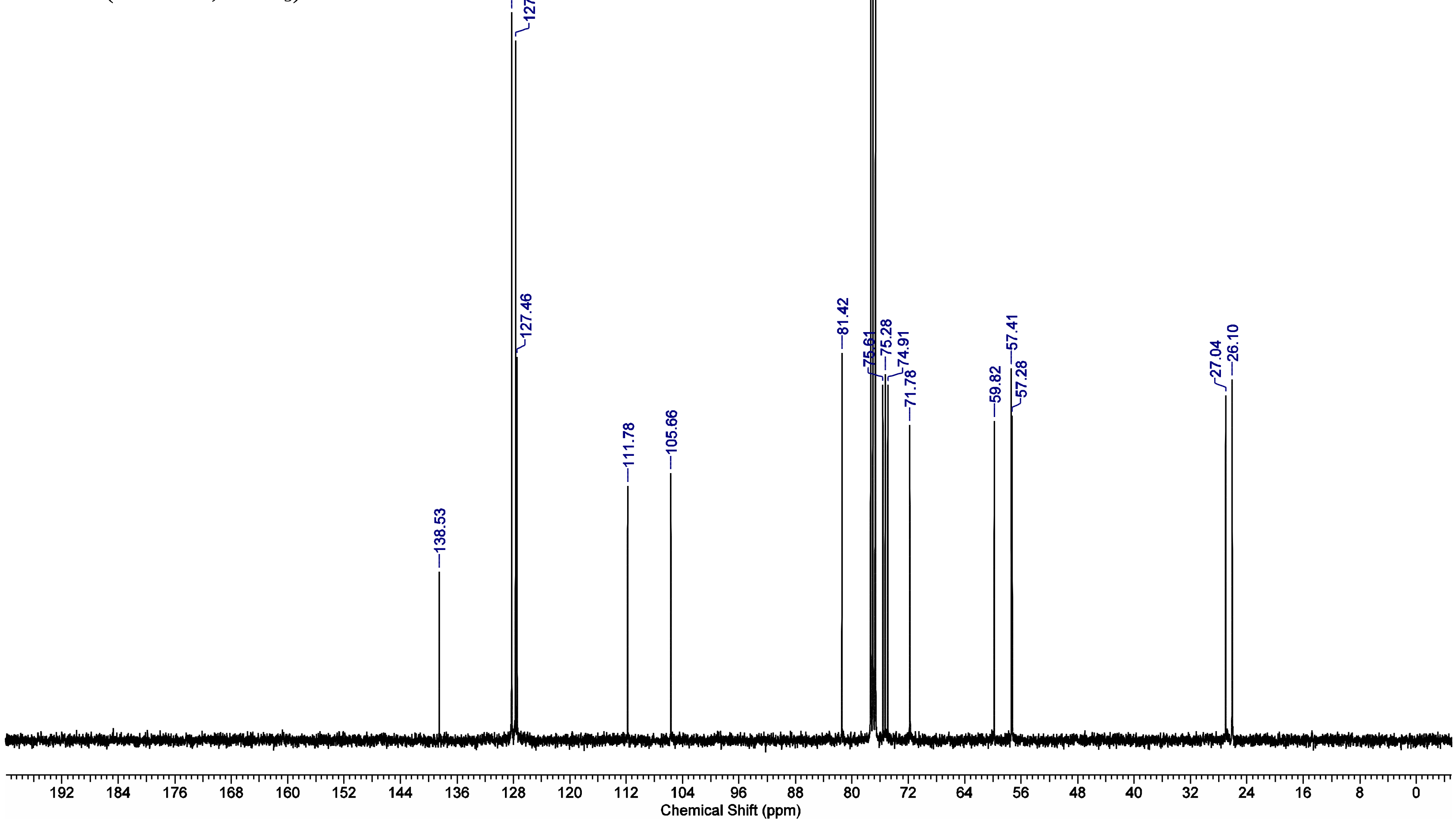

^{1}H NMR (400 MHz, $CDCl_3$)

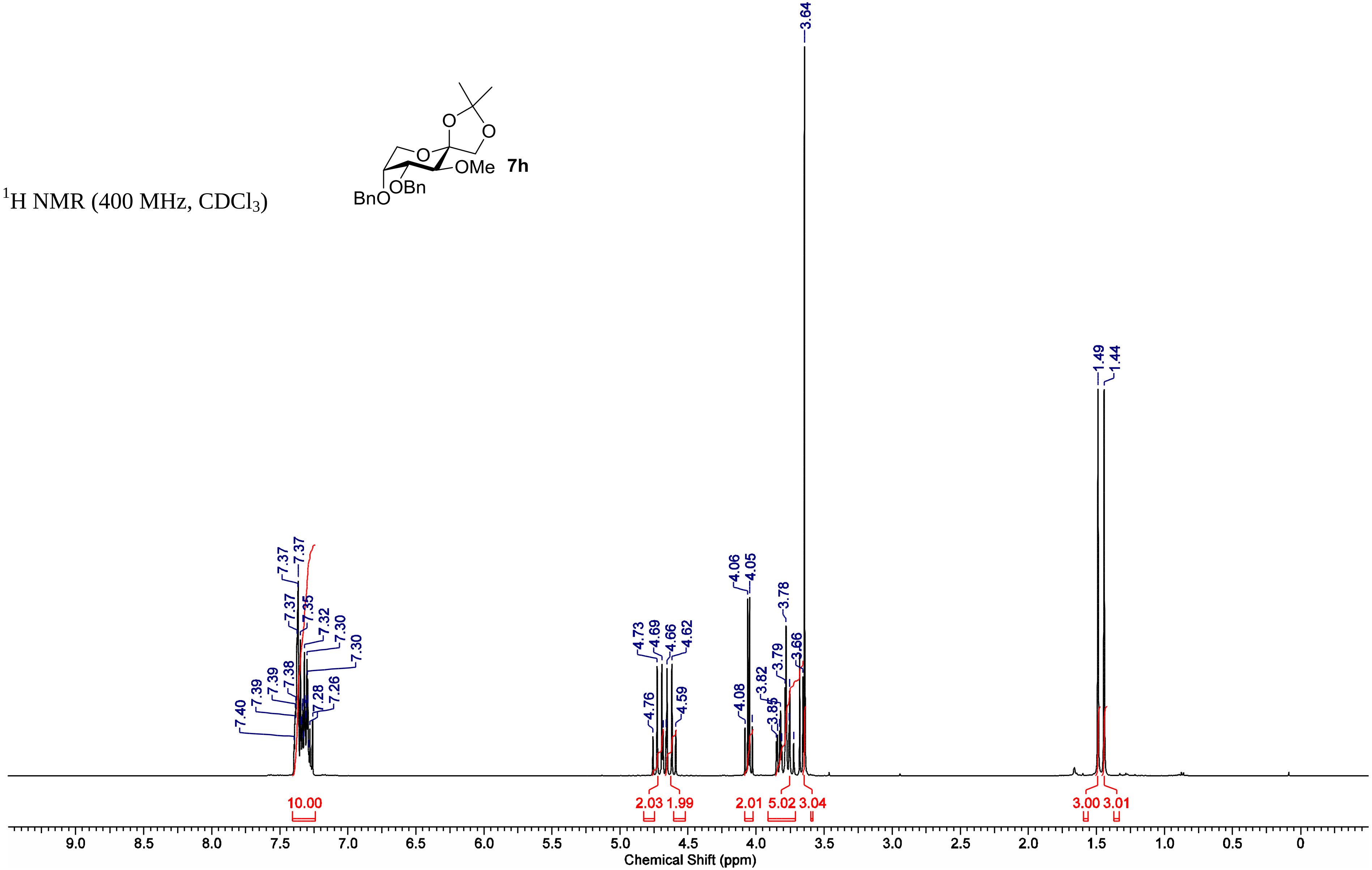

^{13}C NMR (101 MHz, $CDCl_3$)

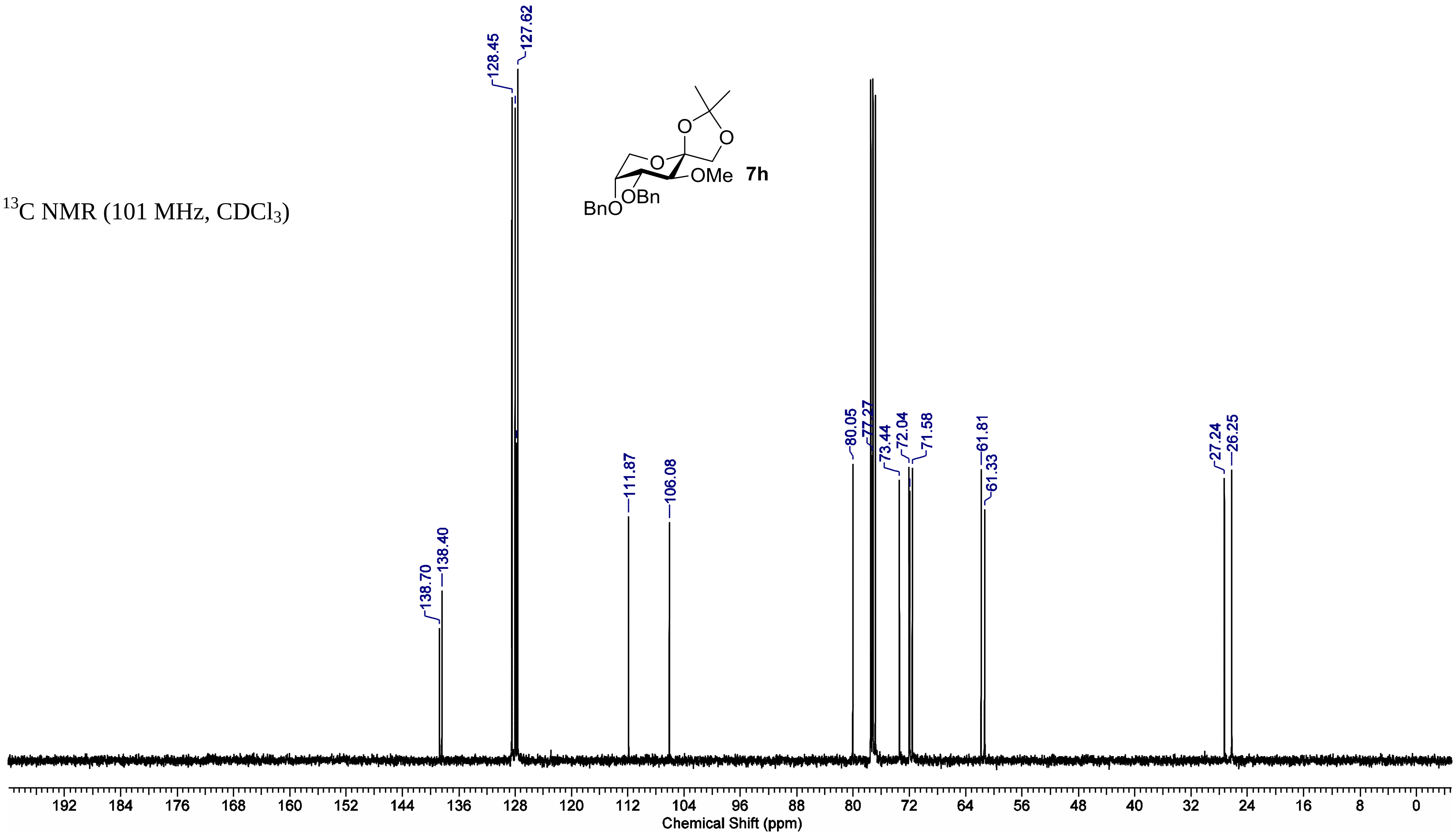

^{1}H NMR (400 MHz, $CDCl_3$)

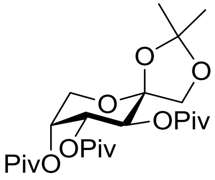

7k

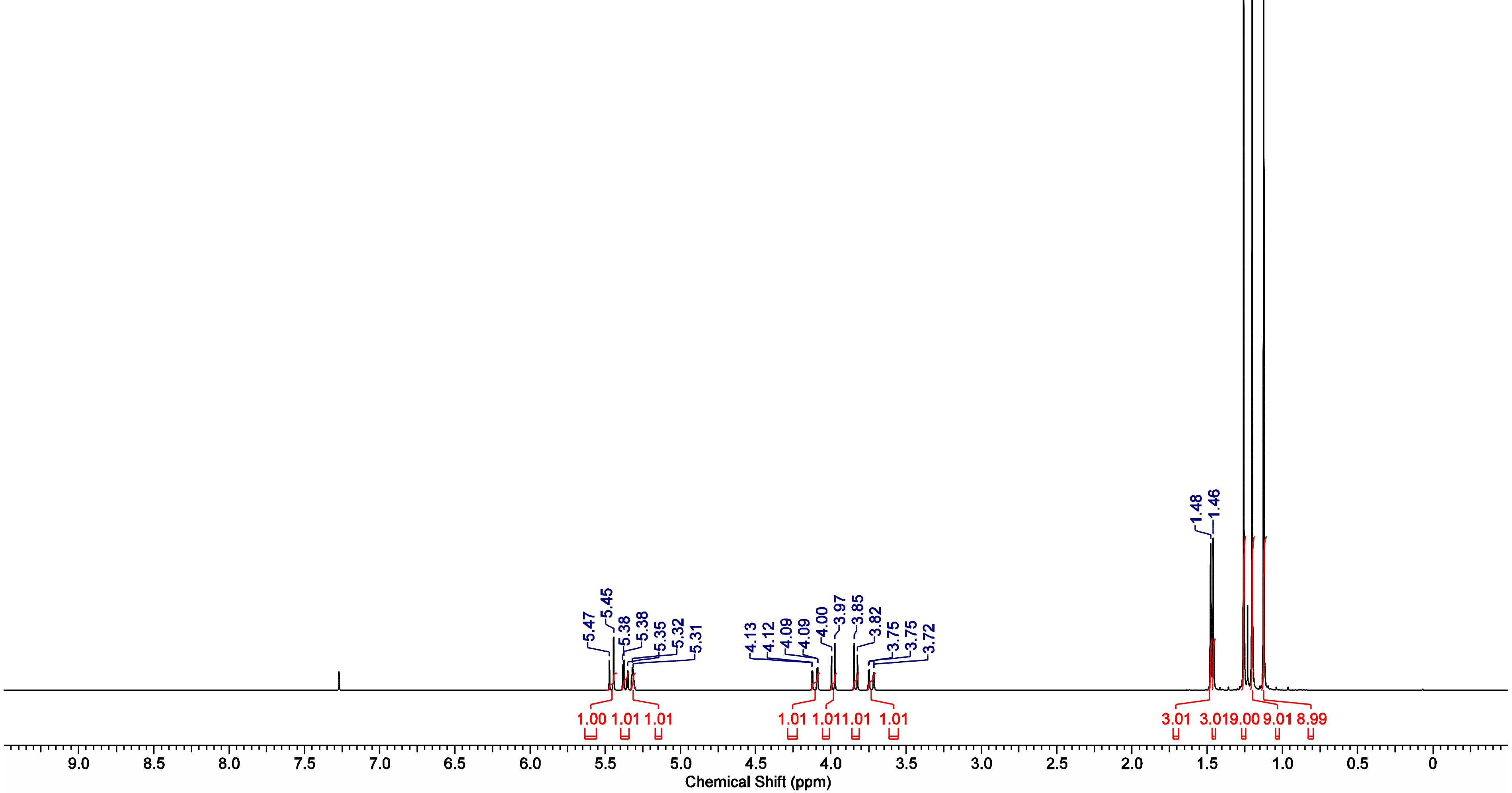

^{13}C NMR (101 MHz, $CDCl_3$)

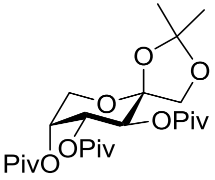

7k

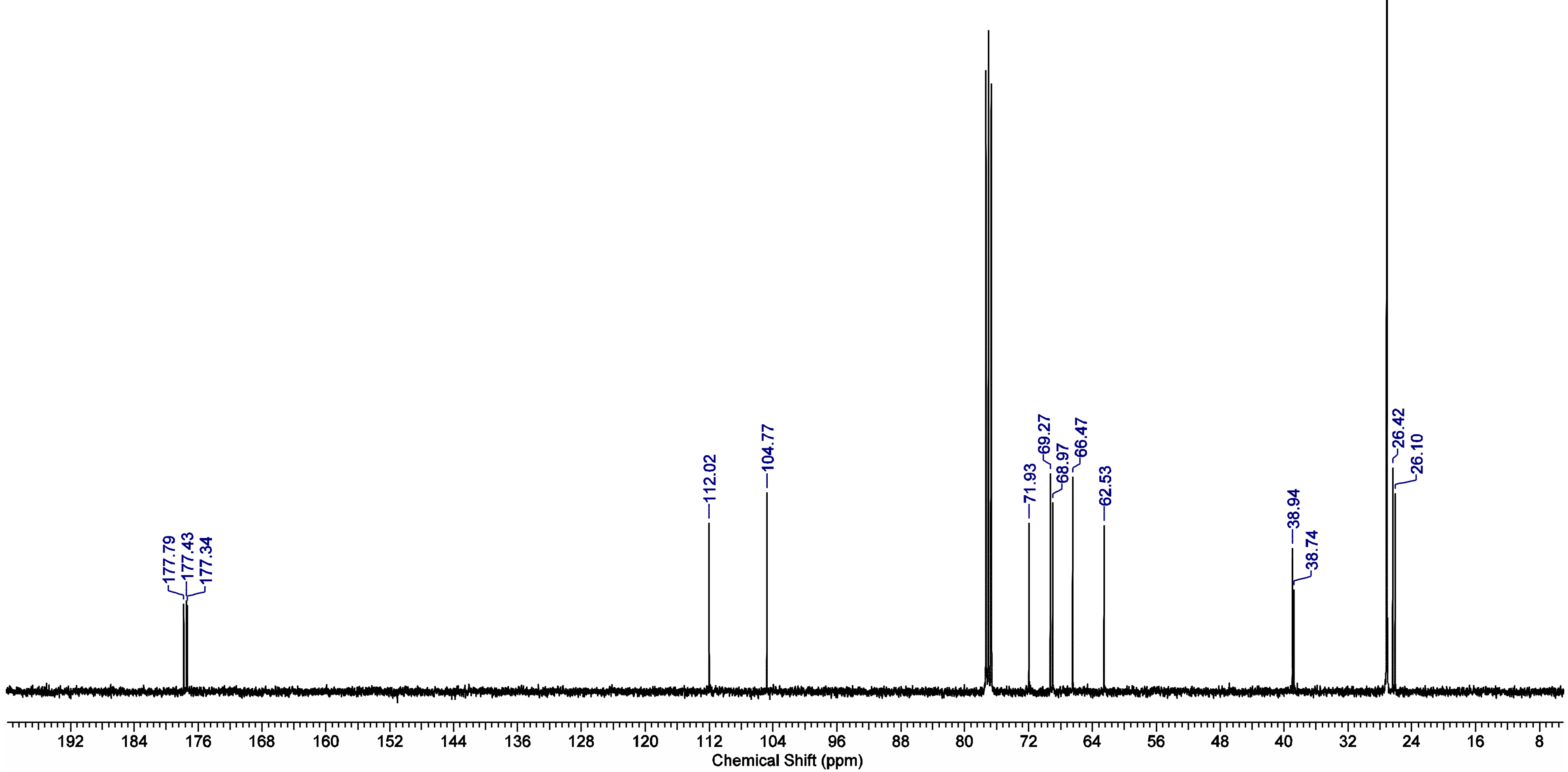

10a

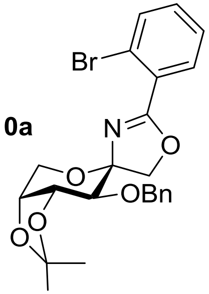

^{1}H NMR (400 MHz, $CDCl_3$)

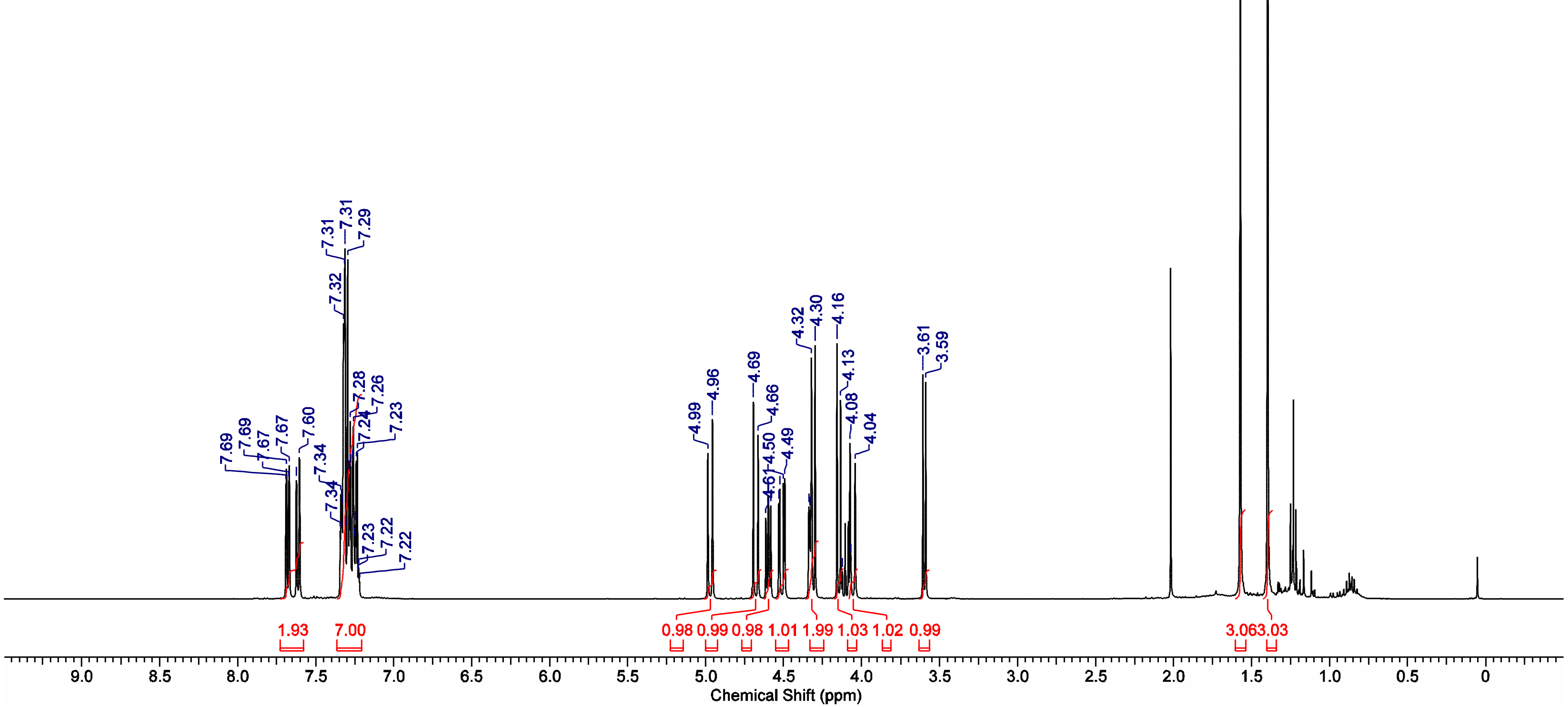

^{13}C NMR (101 MHz, $CDCl_3$)

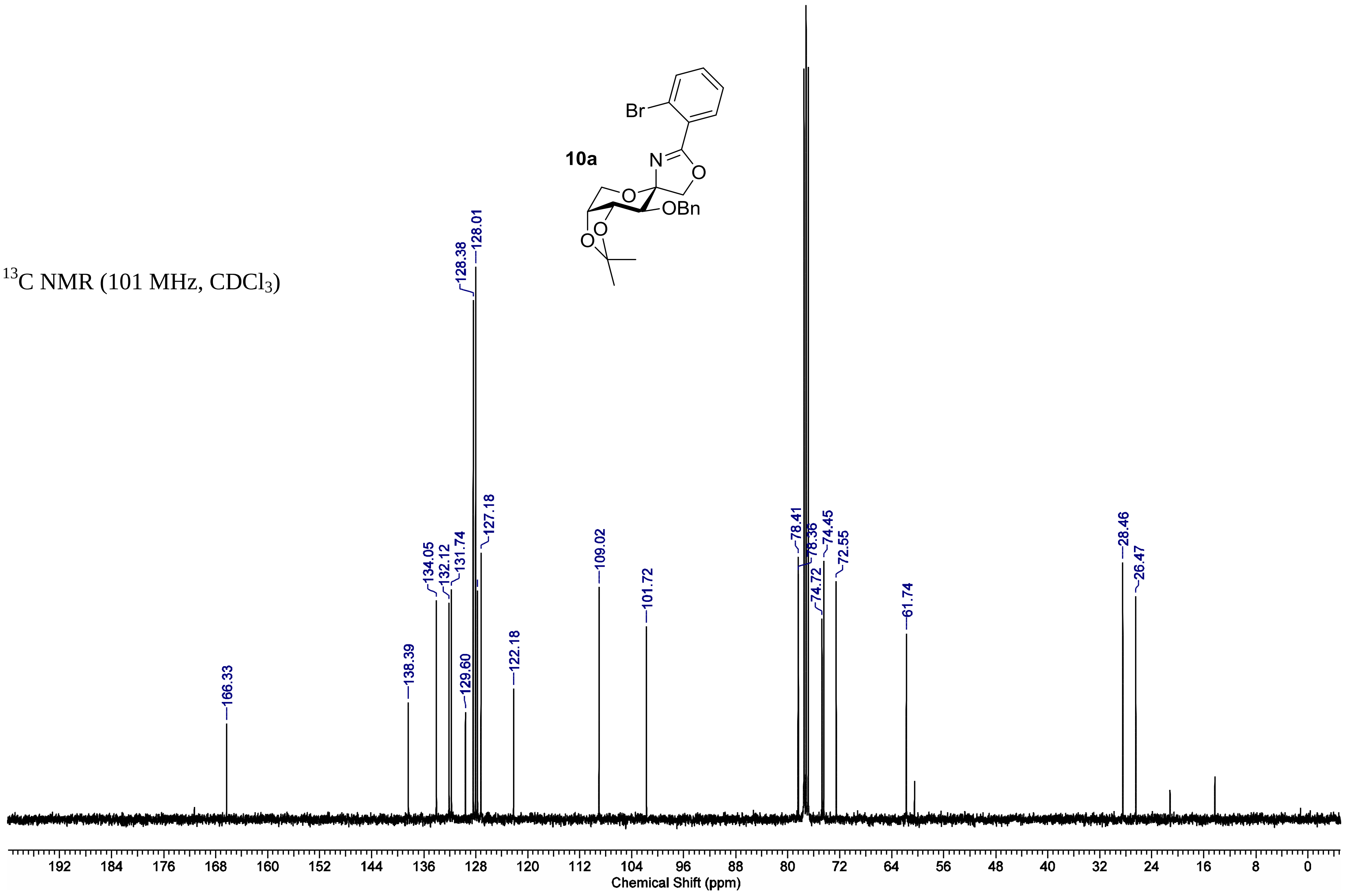

^{1}H NMR (400 MHz, $CDCl_3$)

prepared by the Ritter reaction

10b

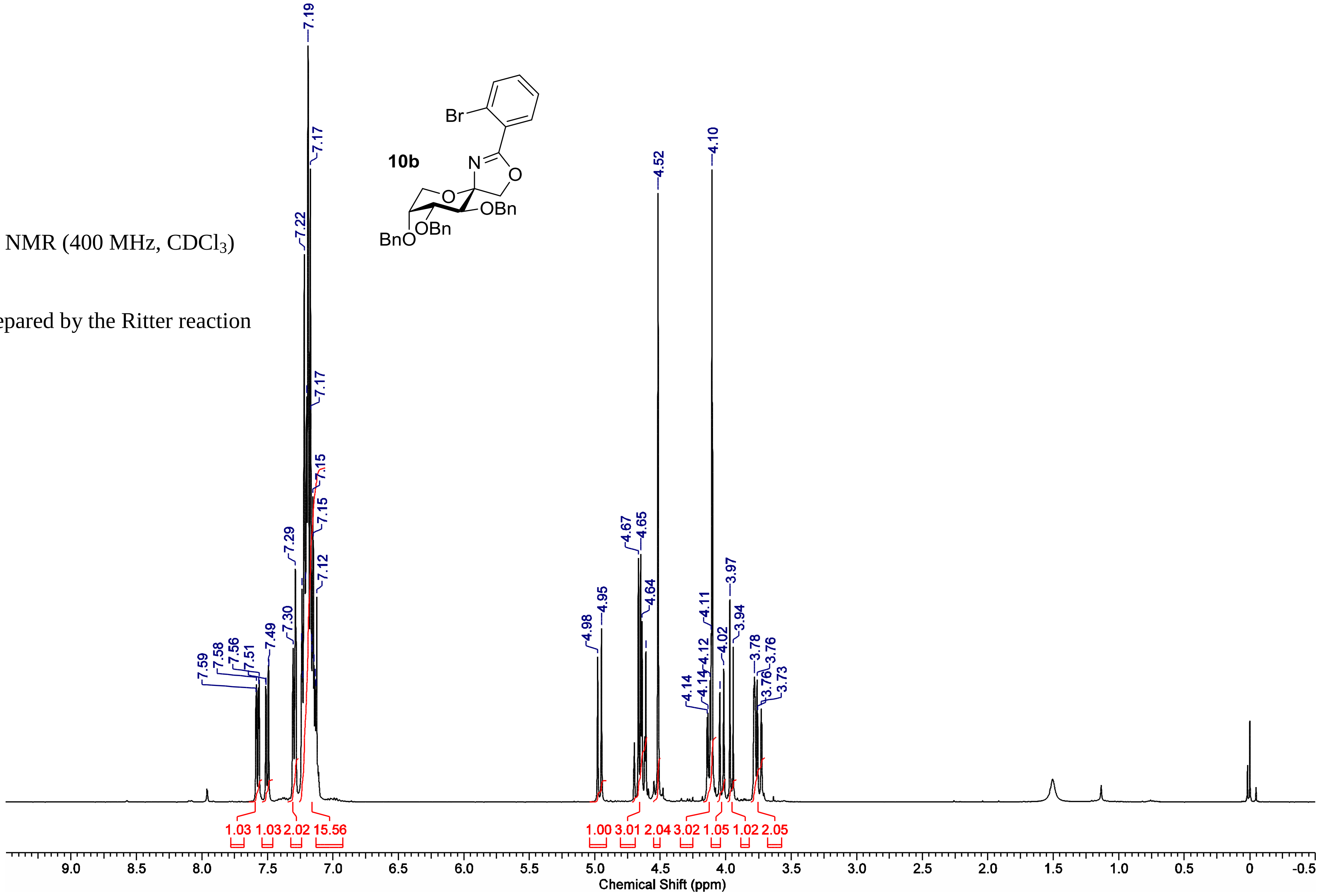

^{13}C NMR (101 MHz, $CDCl_3$)

prepared by the Ritter reaction

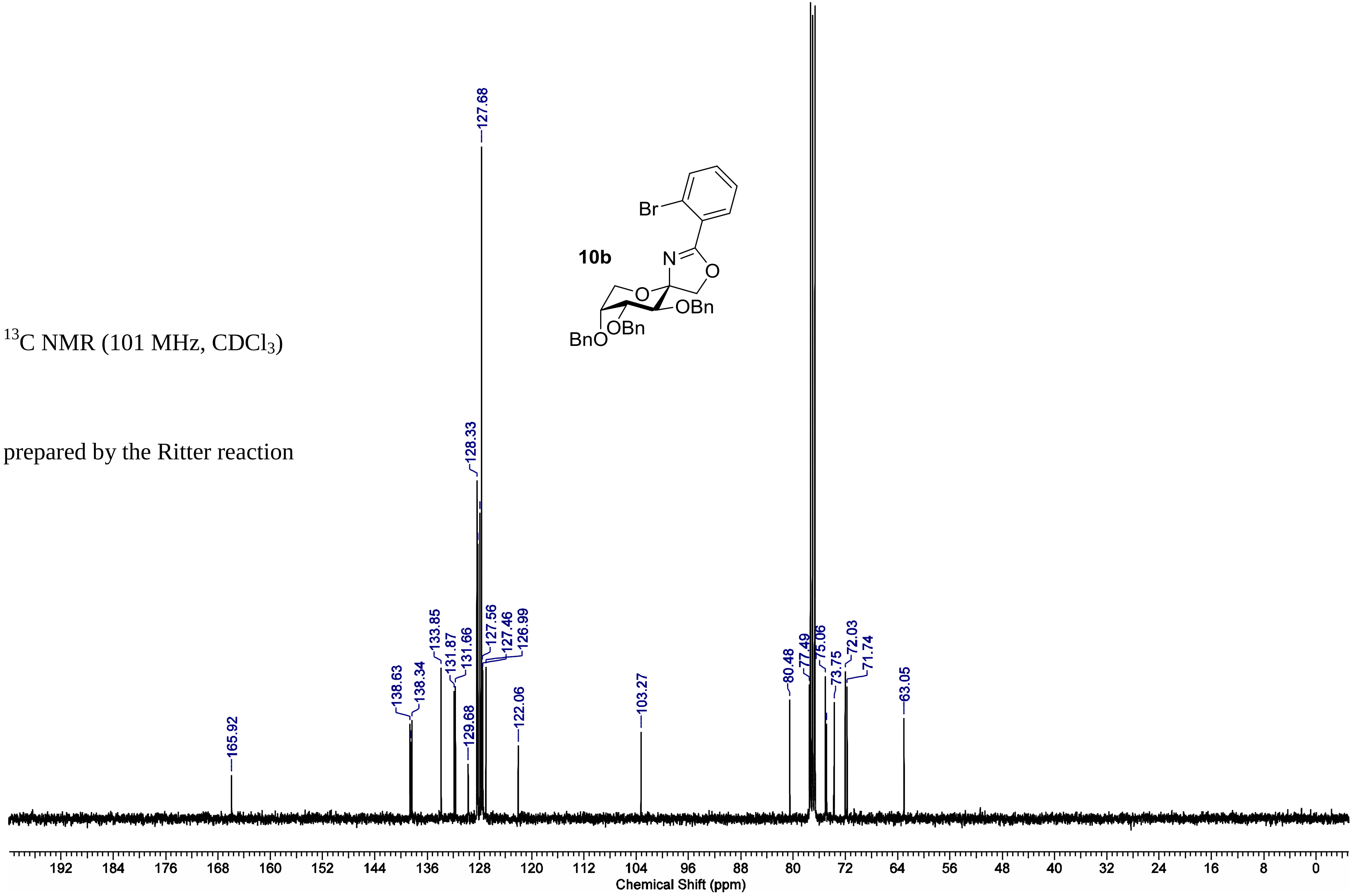

^{1}H NMR (400 MHz, $CDCl_3$)

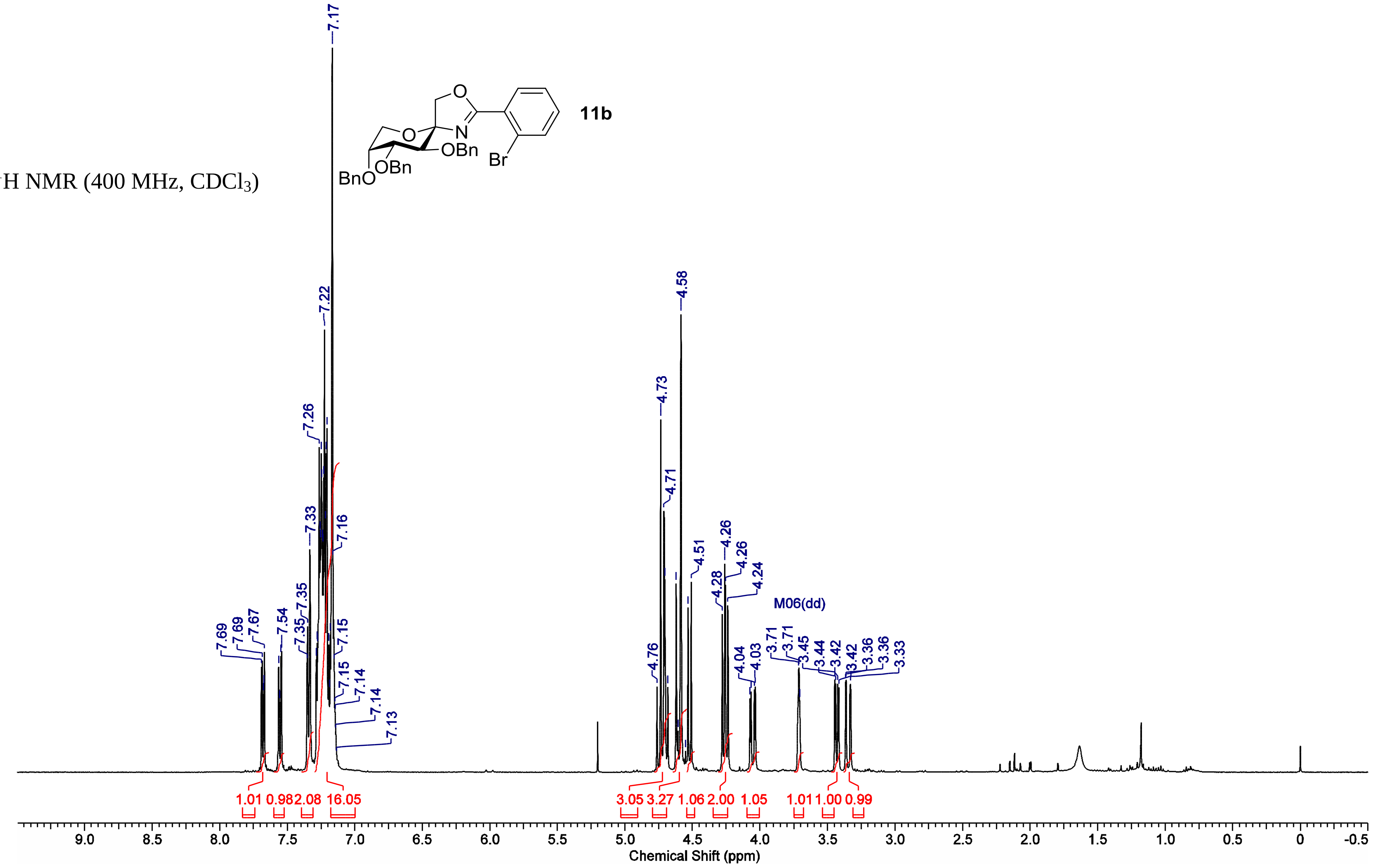

^{13}C NMR (101 MHz, CDCl$_3$)

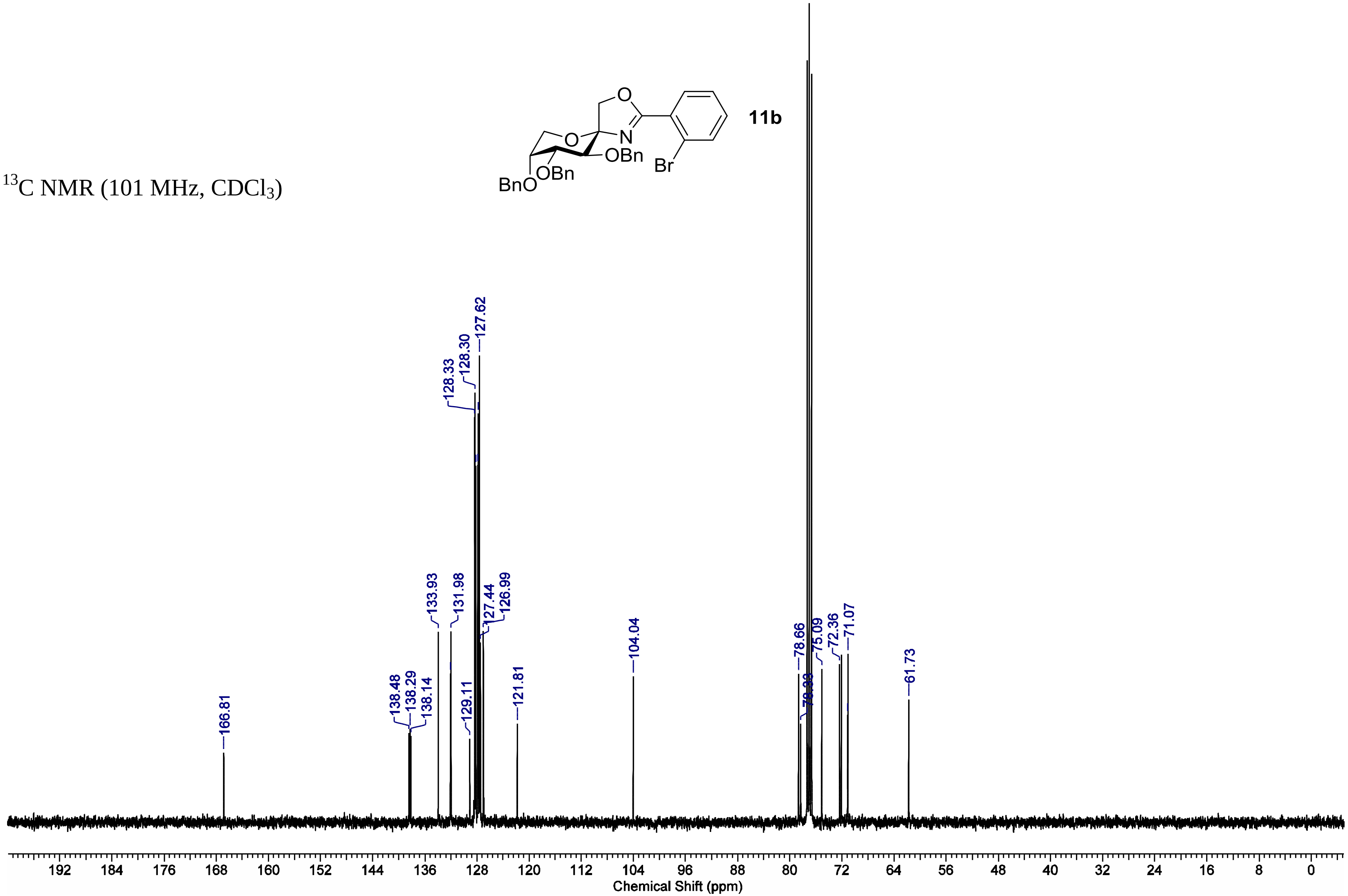

^{1}H NMR (400 MHz, $CDCl_3$)

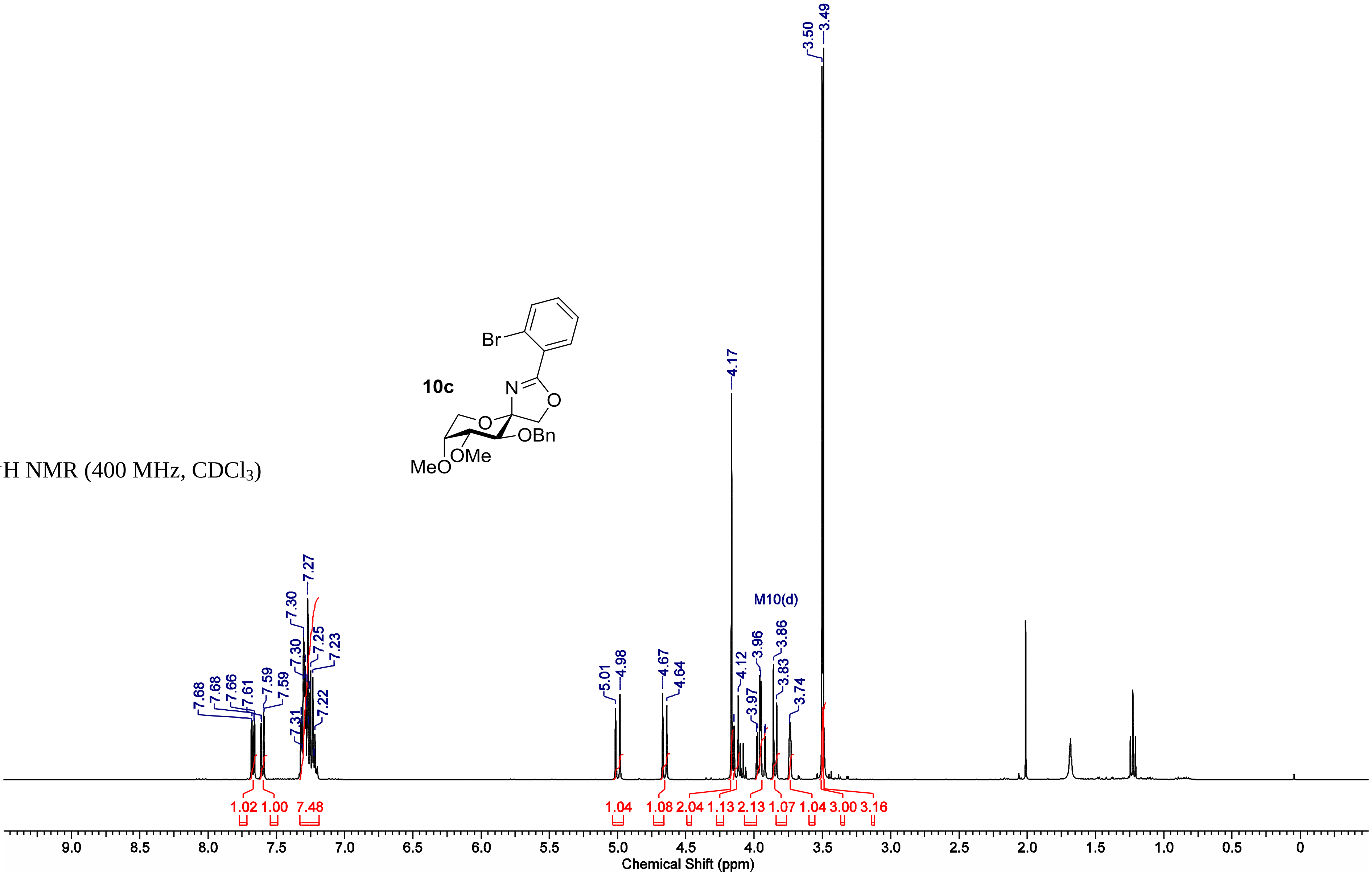

^{13}C NMR (101 MHz, $CDCl_3$)

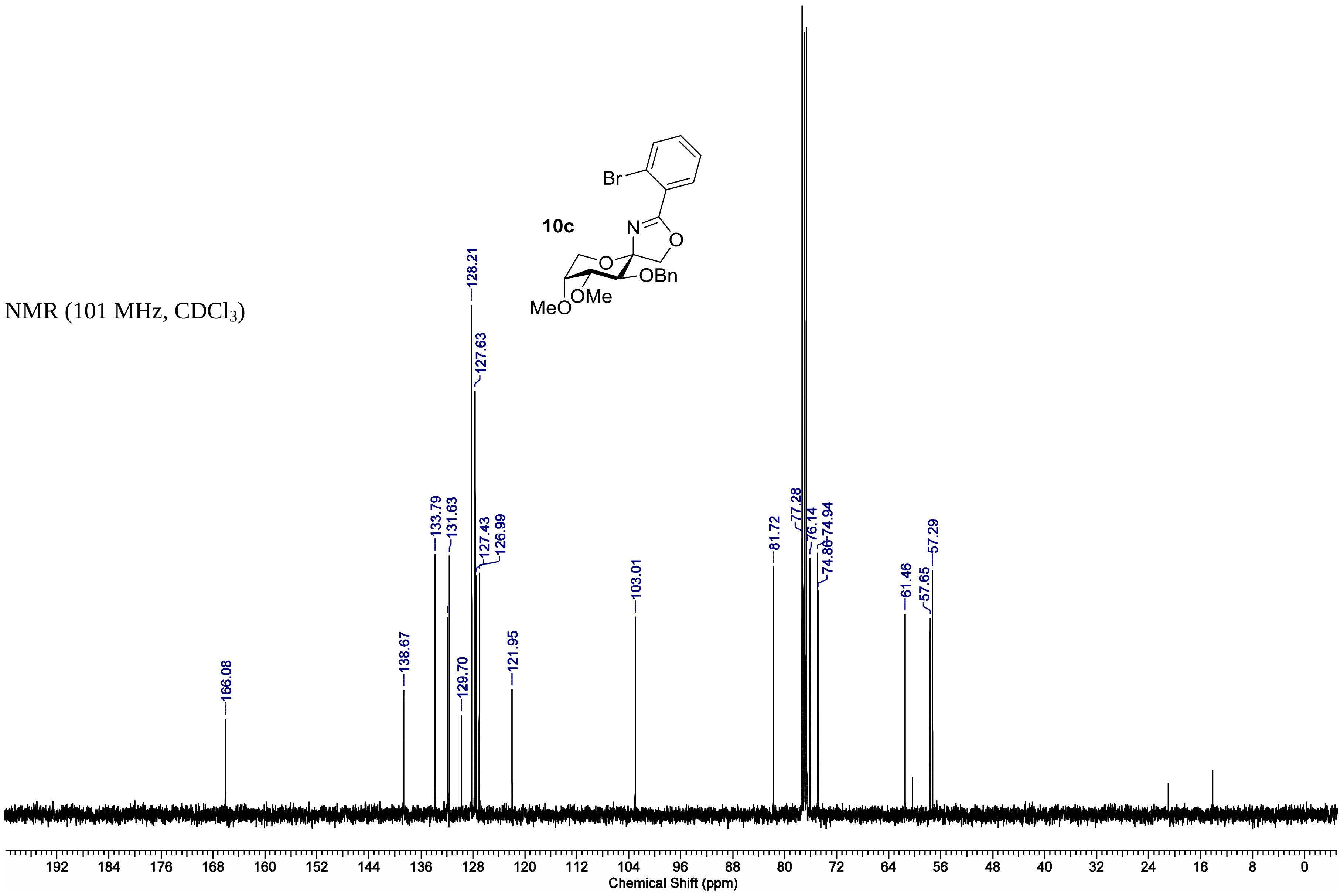

^{1}H NMR (400 MHz, $CDCl_3$)

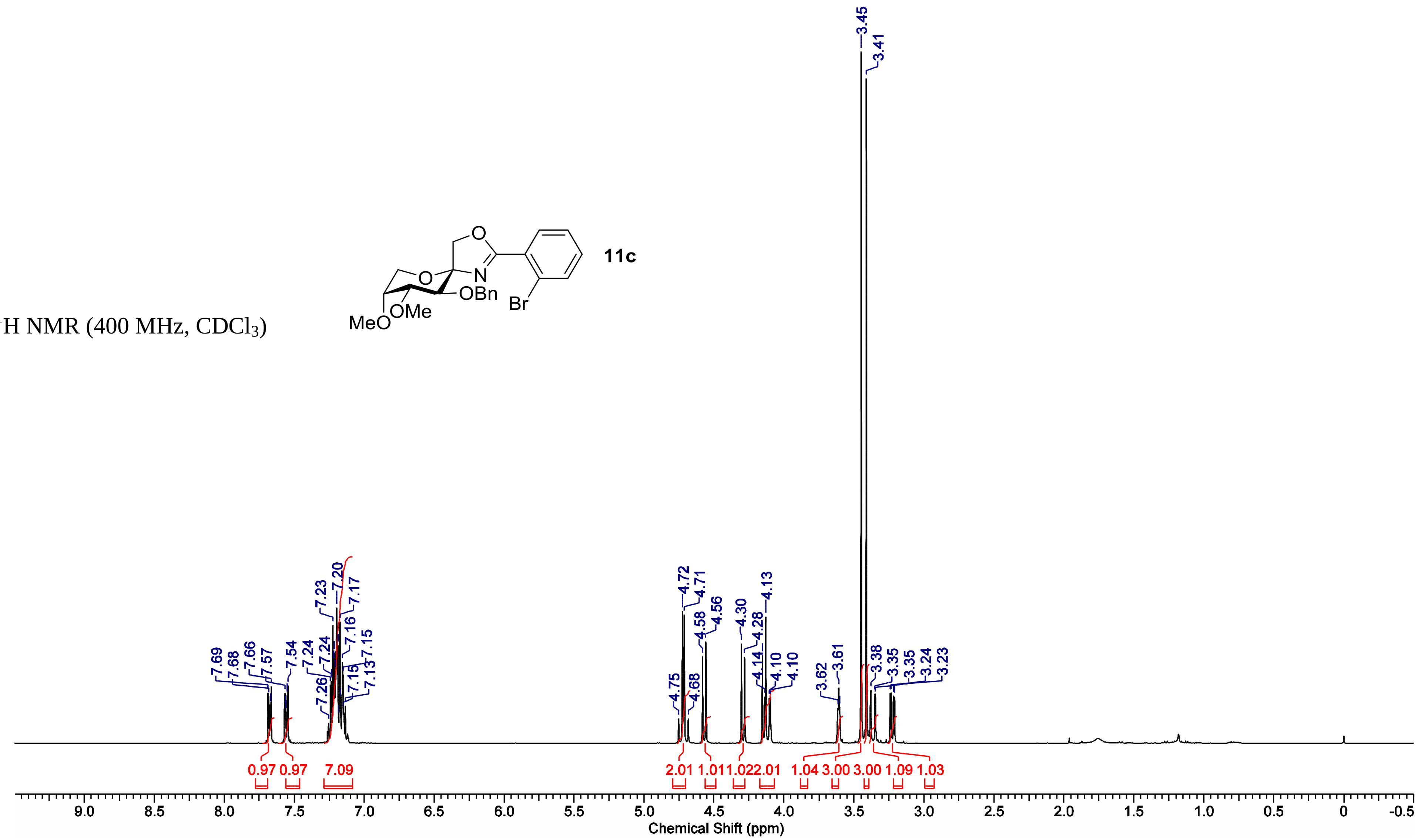

^{13}C NMR (101 MHz, $CDCl_3$)

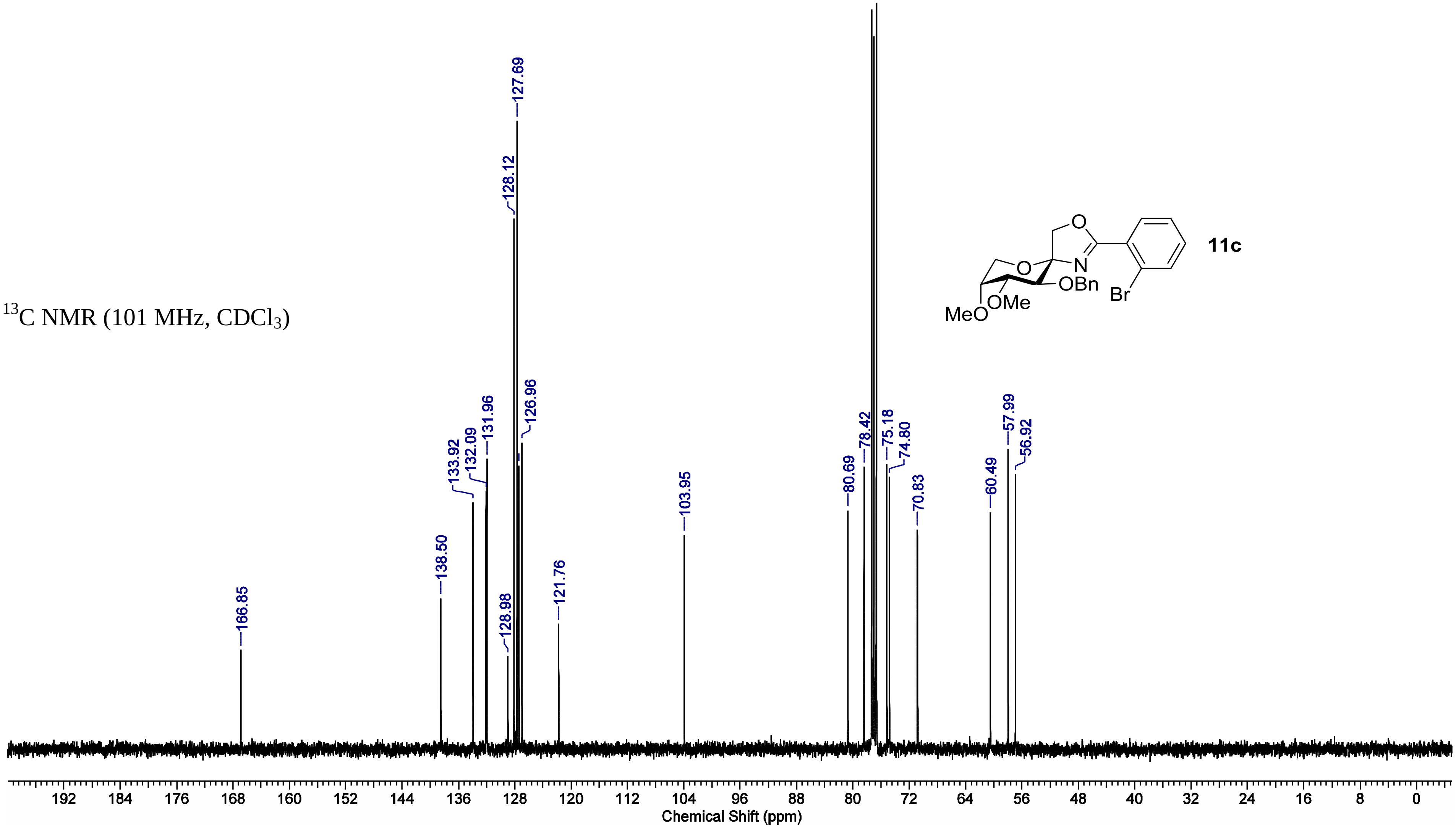

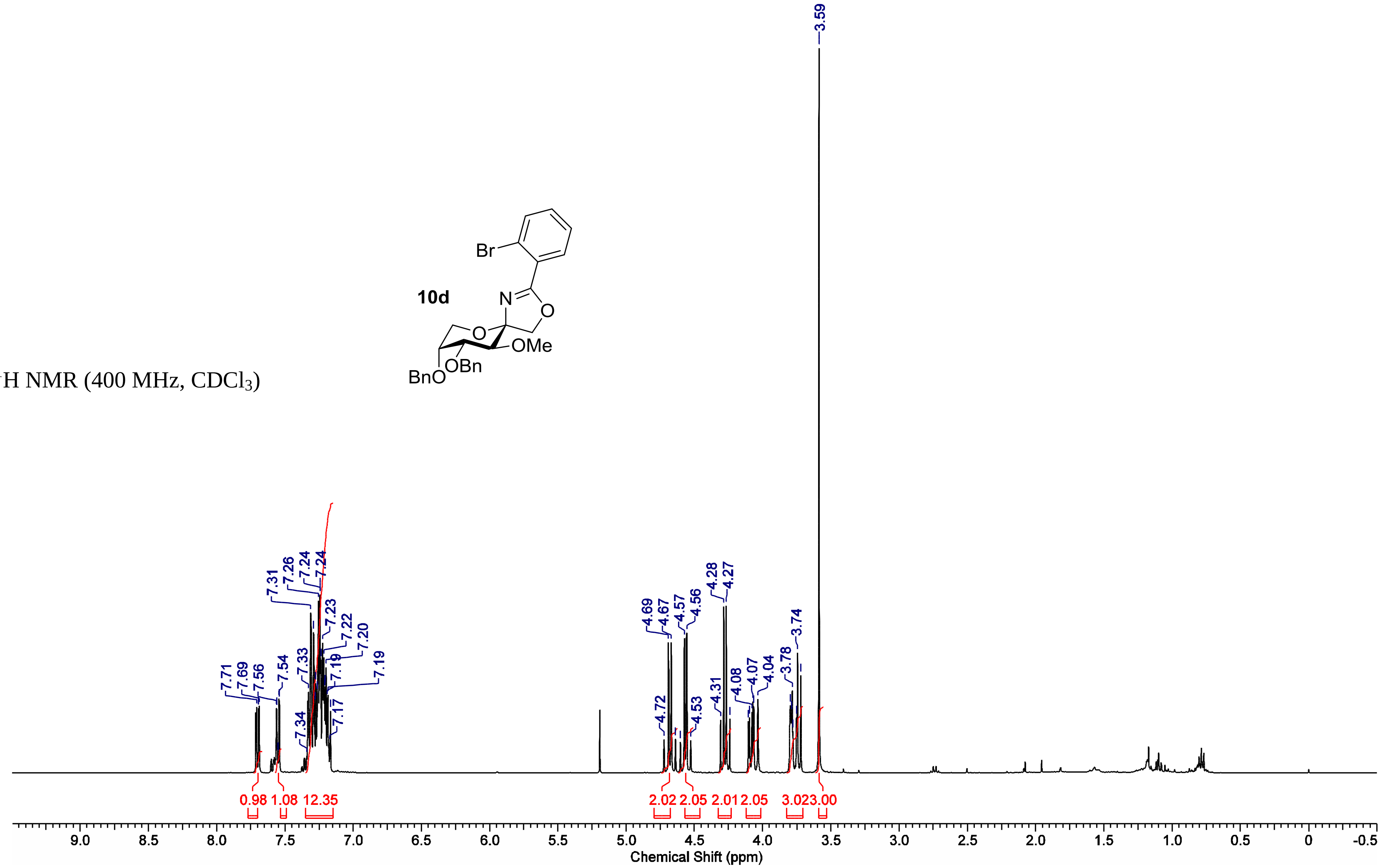

^{1}H NMR (400 MHz, $CDCl_3$)

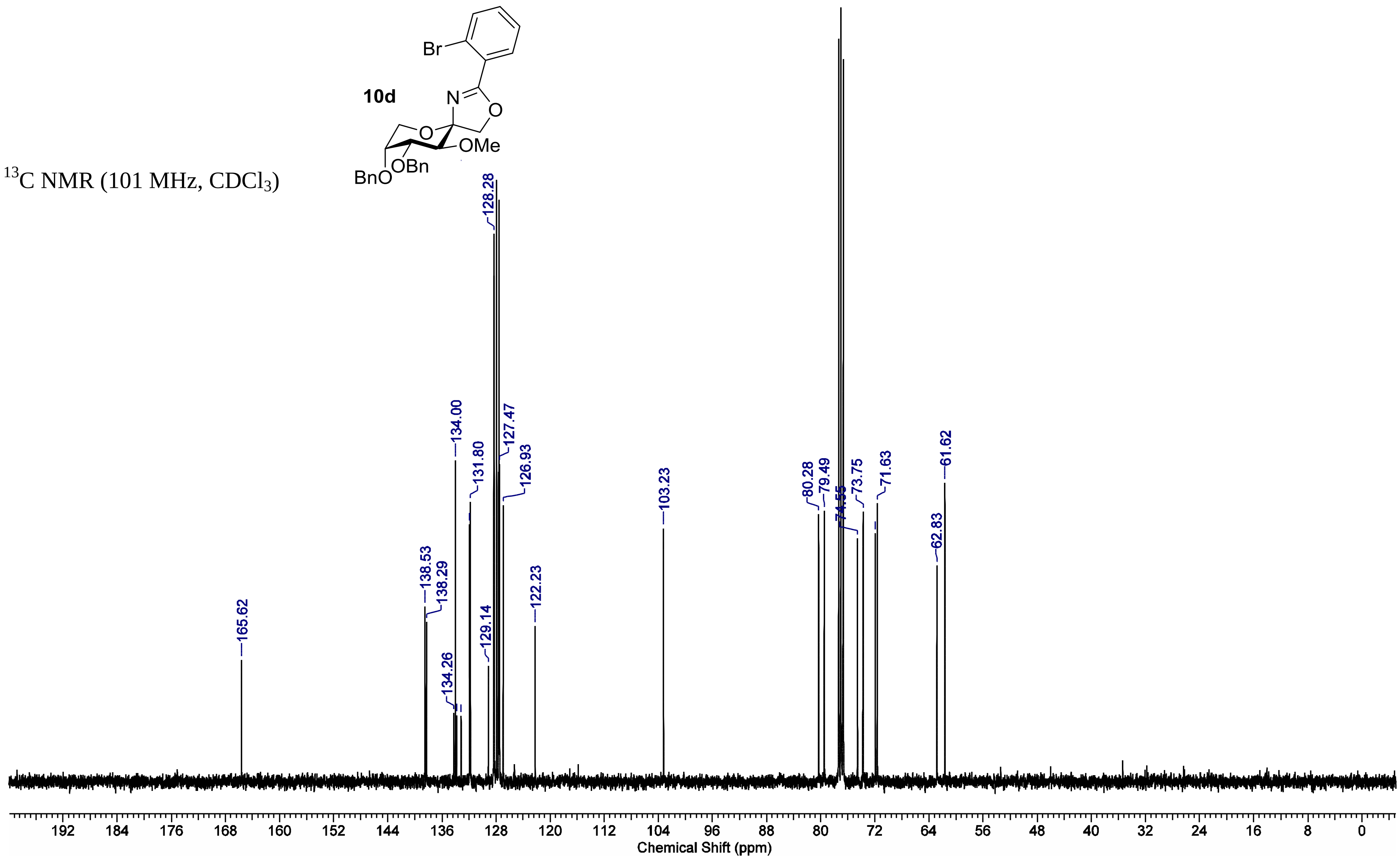
10d
Br
N
O
O
OMe
OBn
BnO
^{13}C NMR (101 MHz, CDCl$_3$)
165.62
138.53
138.29
134.26
134.00
131.80
129.14
128.28
127.47
126.93
122.23
103.23
80.28
79.49
74.55
73.75
71.63
62.83
61.62
192
184
176
168
160
152
144
136
128
120
112
104
96
88
80
72
64
56
48
40
32
24
16
8
0
Chemical Shift (ppm)

^{1}H NMR (400 MHz, $CDCl_3$)

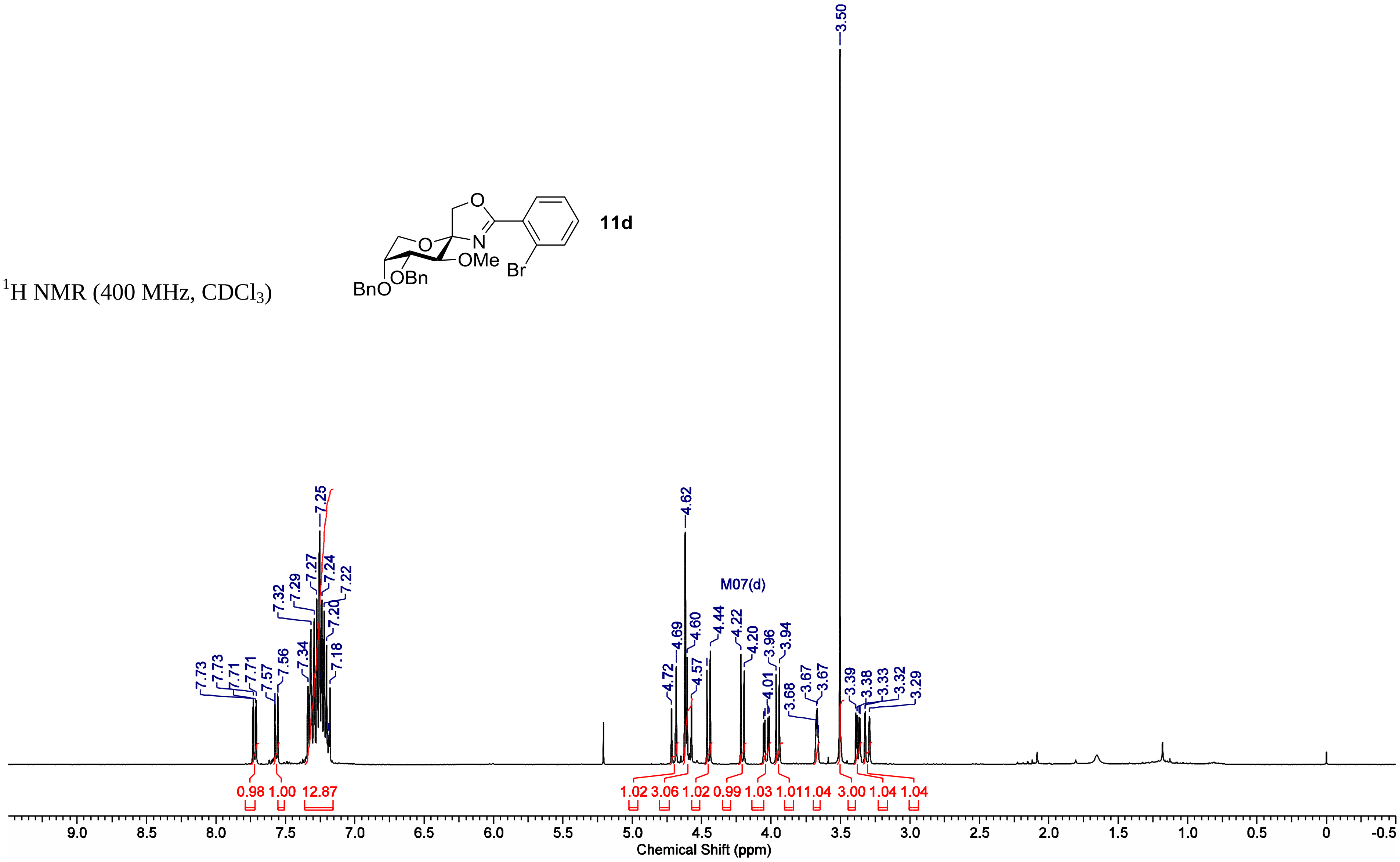

^{13}C NMR (101 MHz, $CDCl_3$)

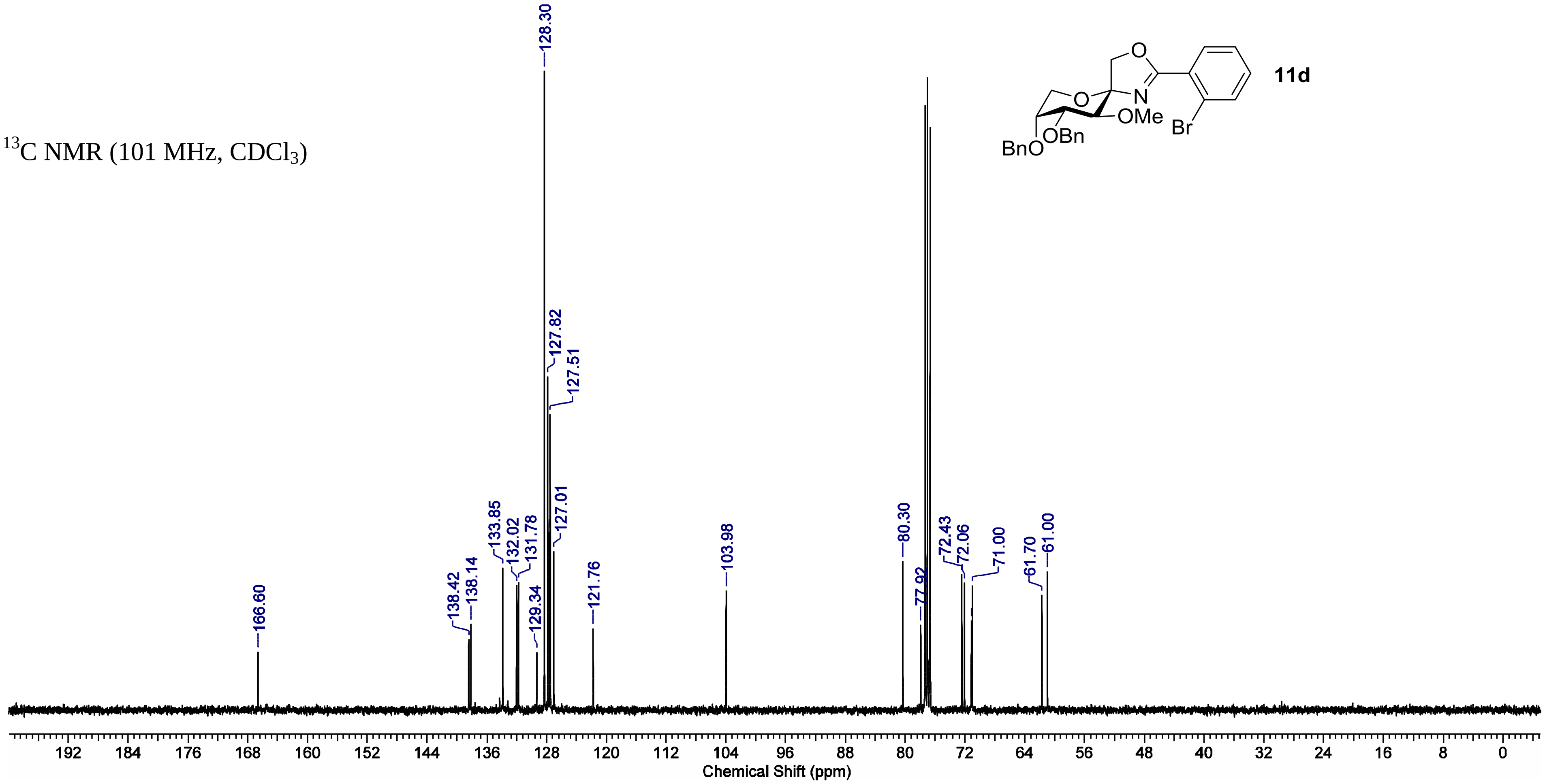

11d

^{1}H NMR (400 MHz, $CDCl_3$)

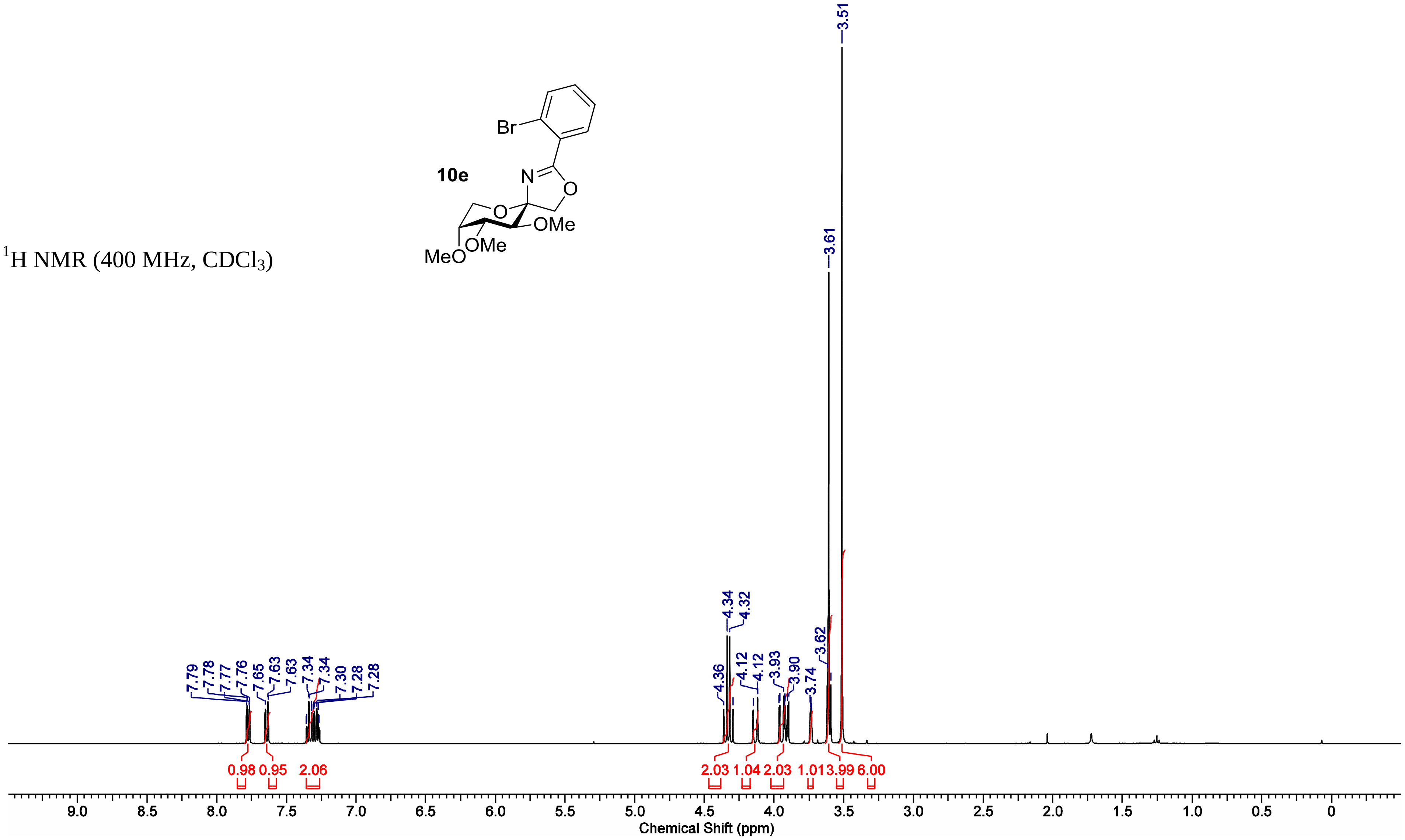

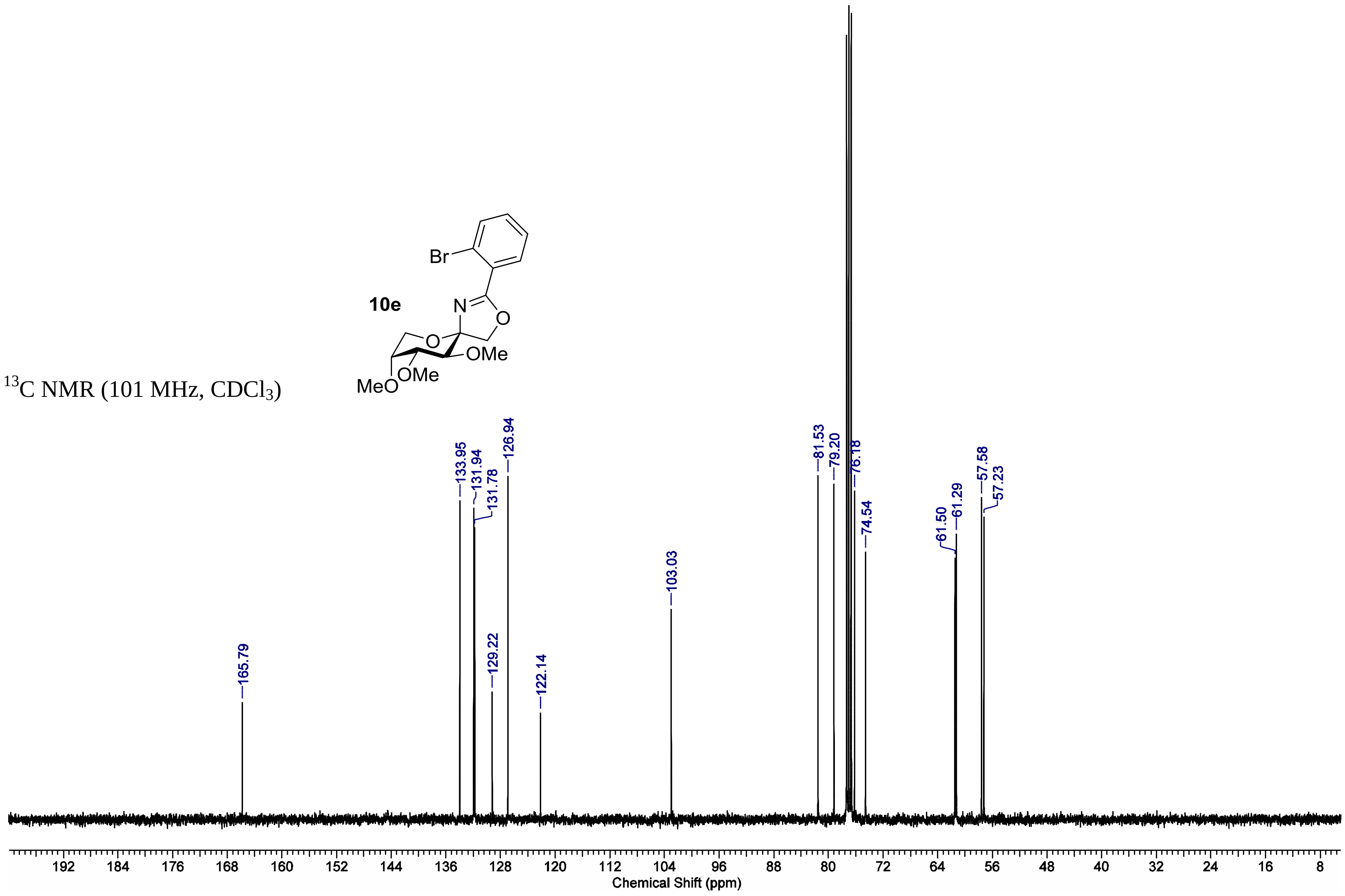

^{13}C NMR (101 MHz, $CDCl_3$)

^{1}H NMR (400 MHz, $CDCl_3$)

11e

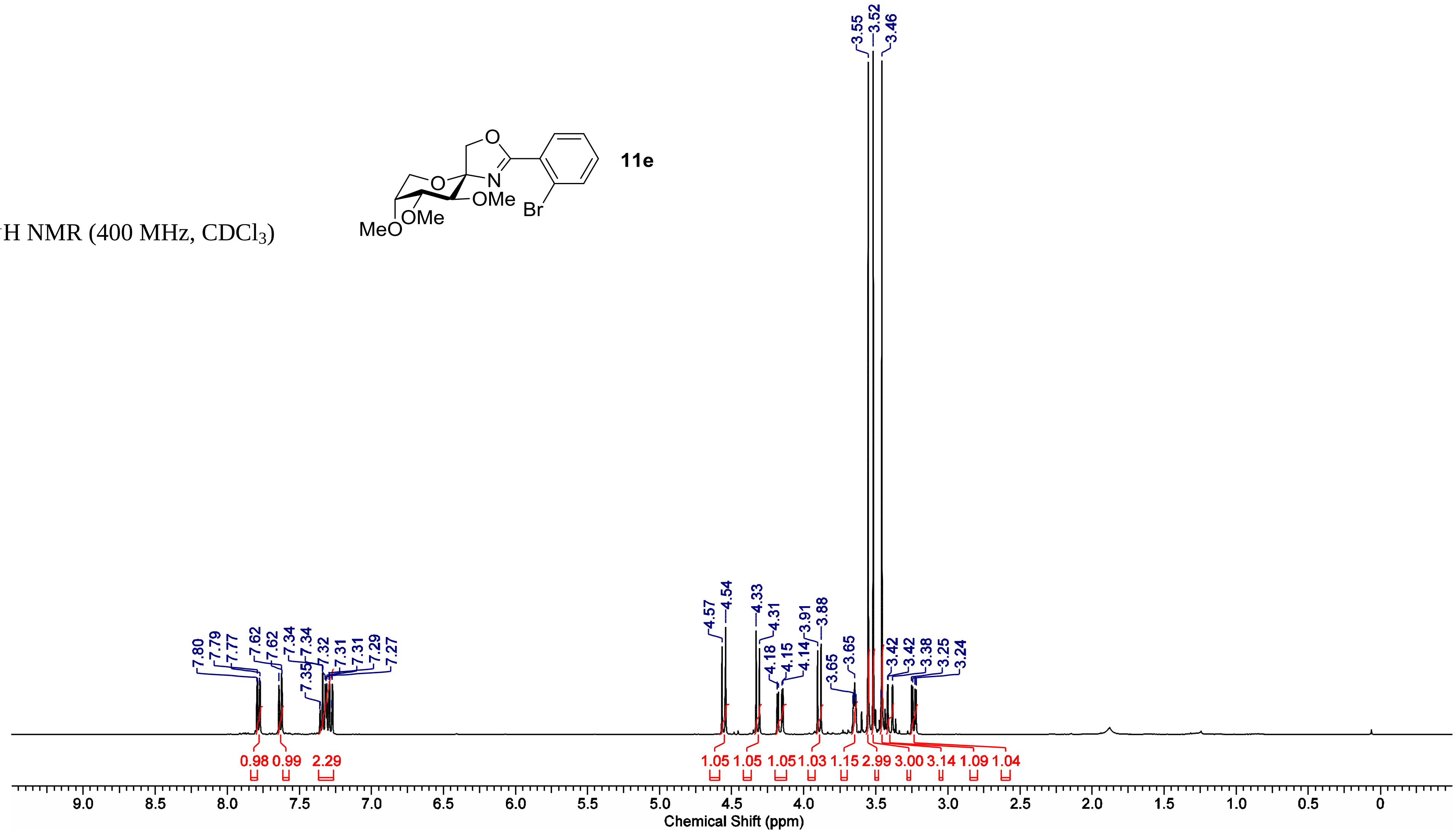

^{13}C NMR (101 MHz, $CDCl_3$)

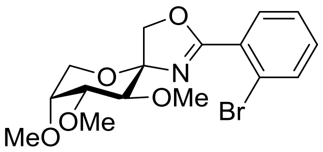

11e

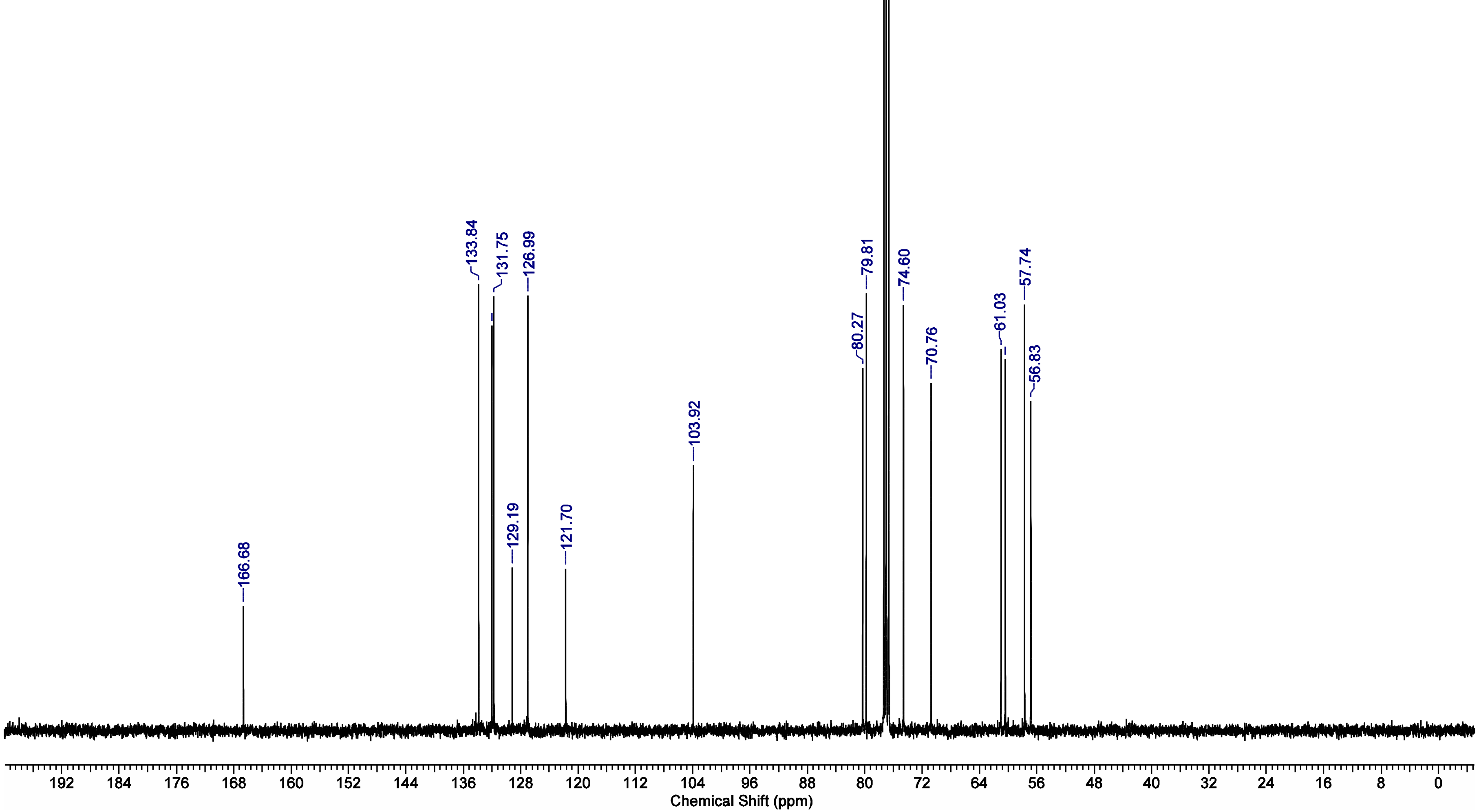

^{1}H NMR (400 MHz, $CDCl_3$)

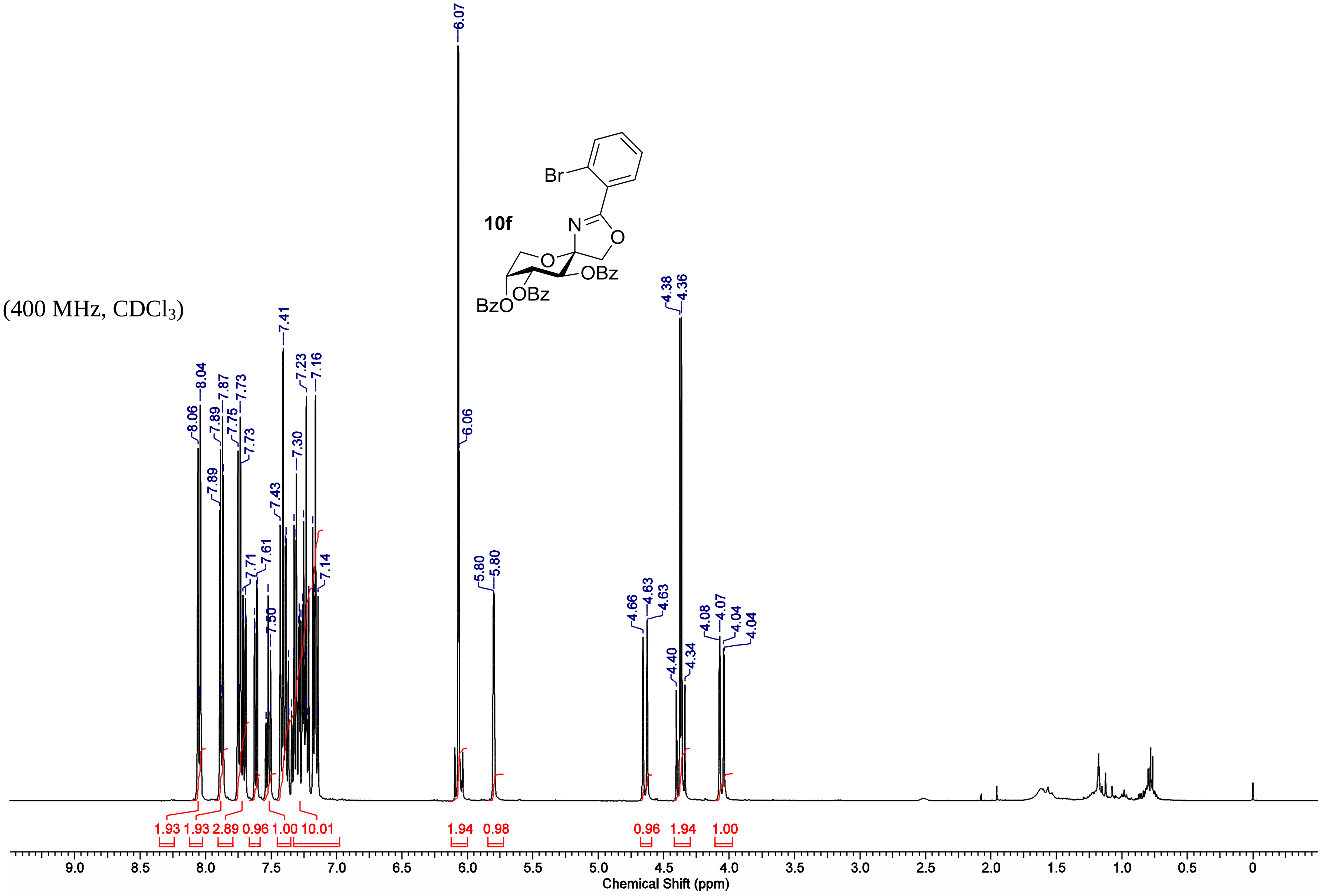

^{13}C NMR (101 MHz, $CDCl_3$)

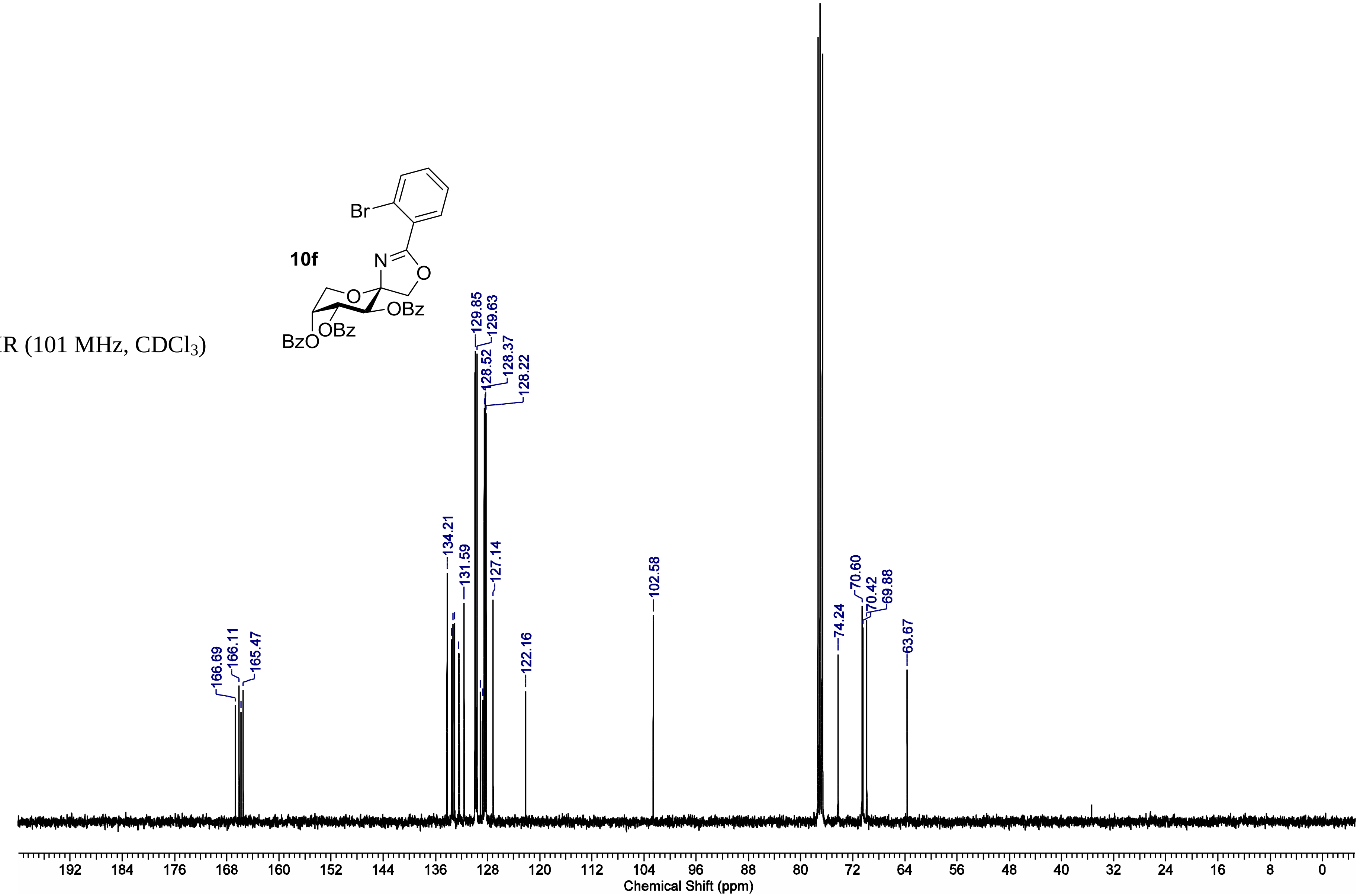

^{1}H NMR (400 MHz, $CDCl_3$)

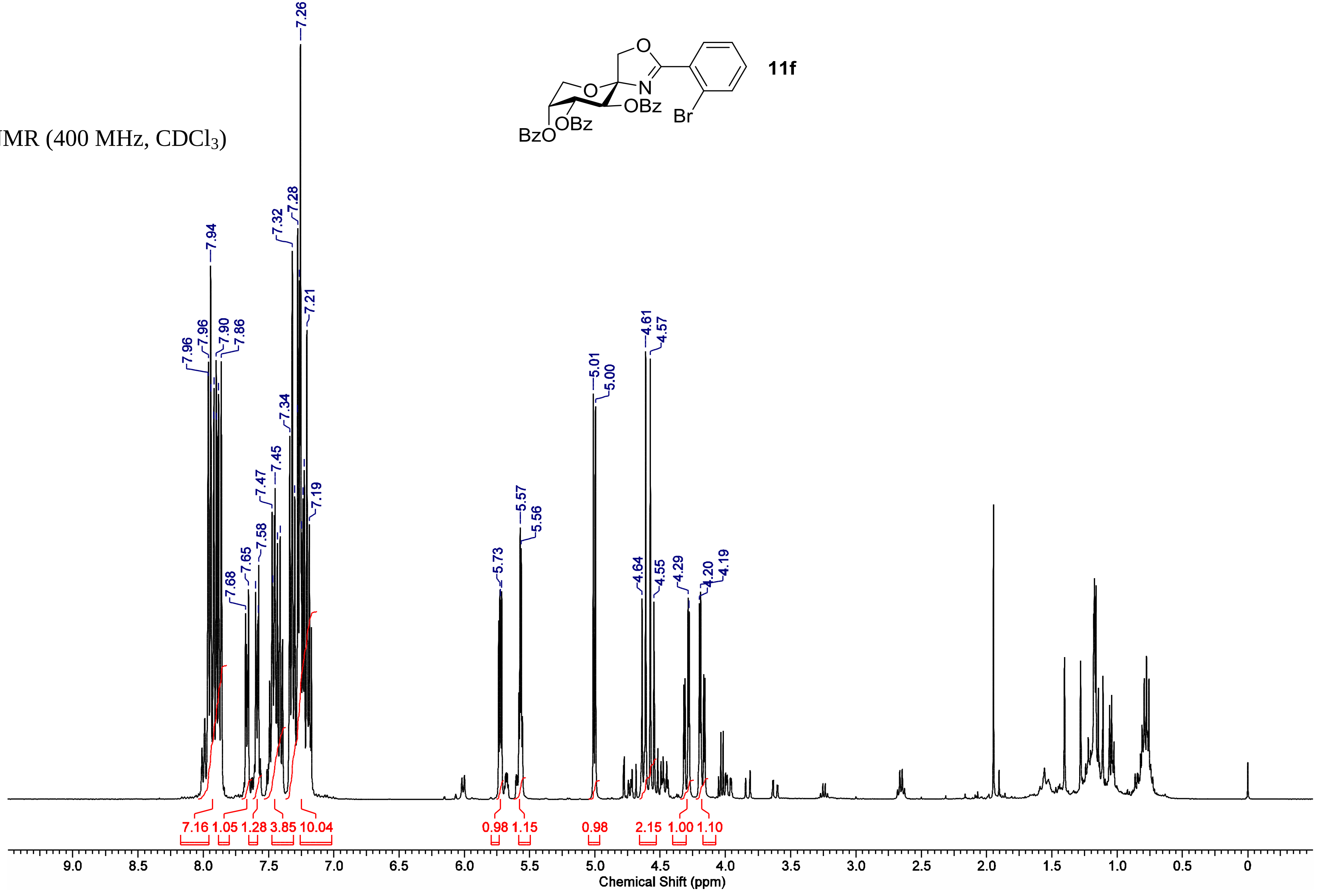

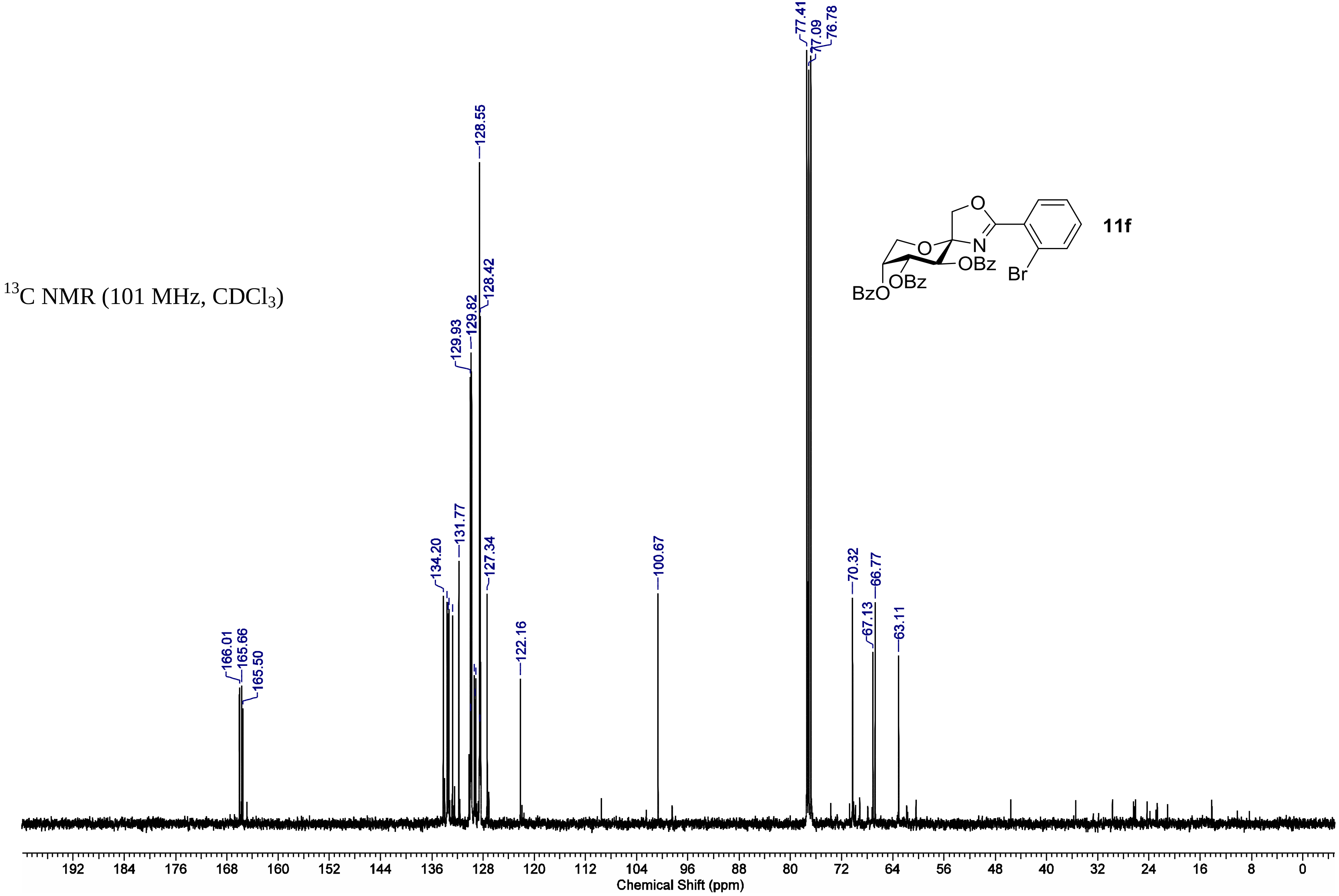

13C NMR (101 MHz, CDCl3)
11f
O
N
O
OBz
OBz
BzO
Br
166.01
165.66
165.50
134.20
131.77
129.93
129.82
128.55
128.42
127.34
122.16
100.67
77.41
77.09
76.78
70.32
67.13
66.77
63.11
192
184
176
168
160
152
144
136
128
120
112
104
96
88
80
72
64
56
48
40
32
24
16
8
0
Chemical Shift (ppm)

10g

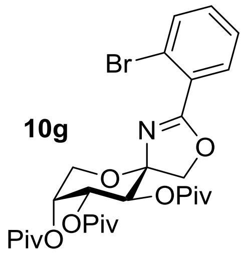

^{1}H NMR (400 MHz, $CDCl_3$)

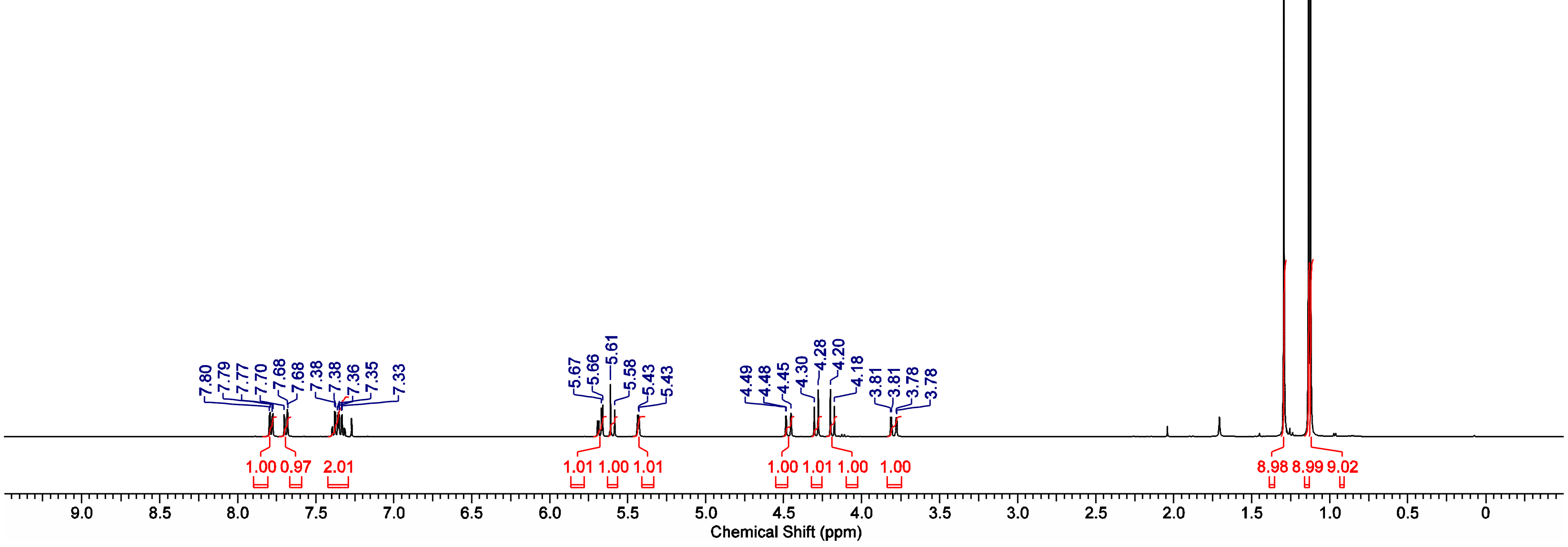

^{13}C NMR (101 MHz, $CDCl_3$)

10g

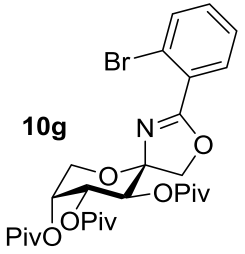

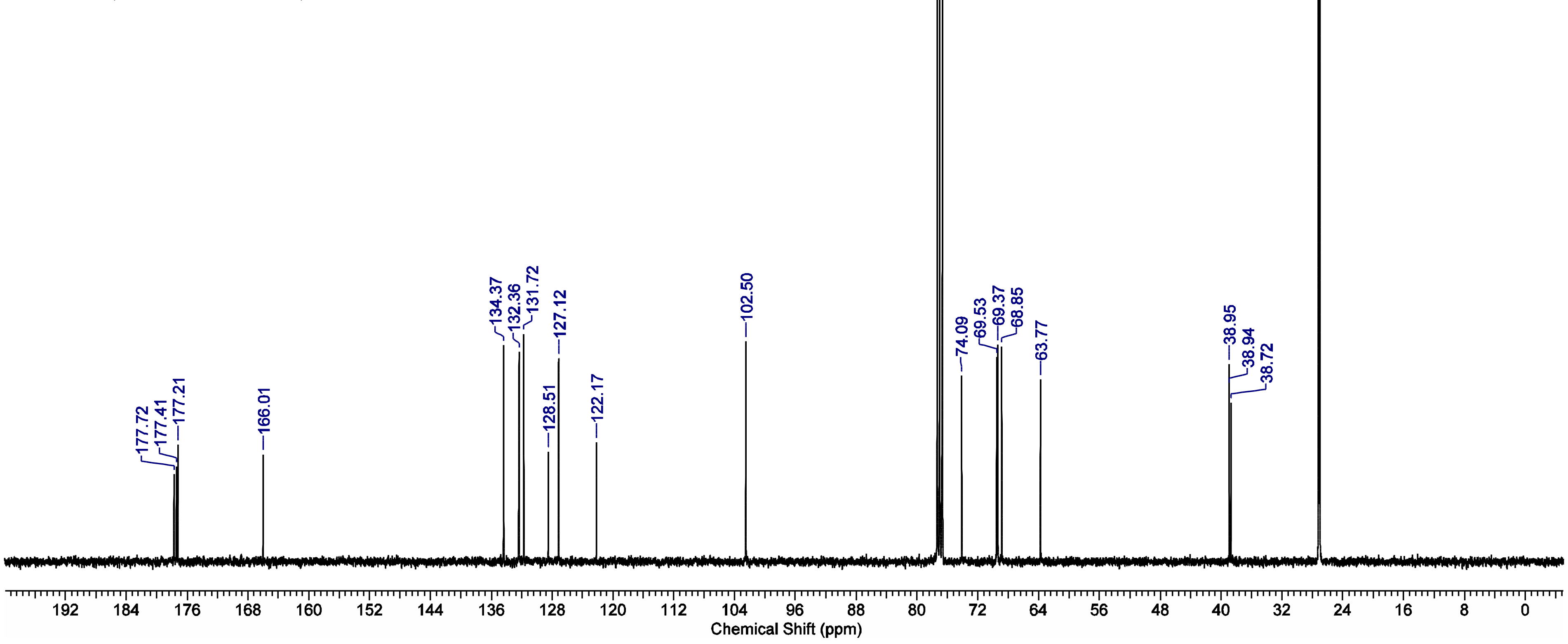

^{1}H NMR (400 MHz, $CDCl_3$)

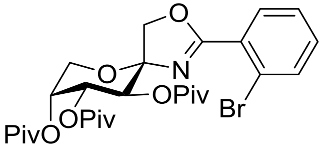

11g

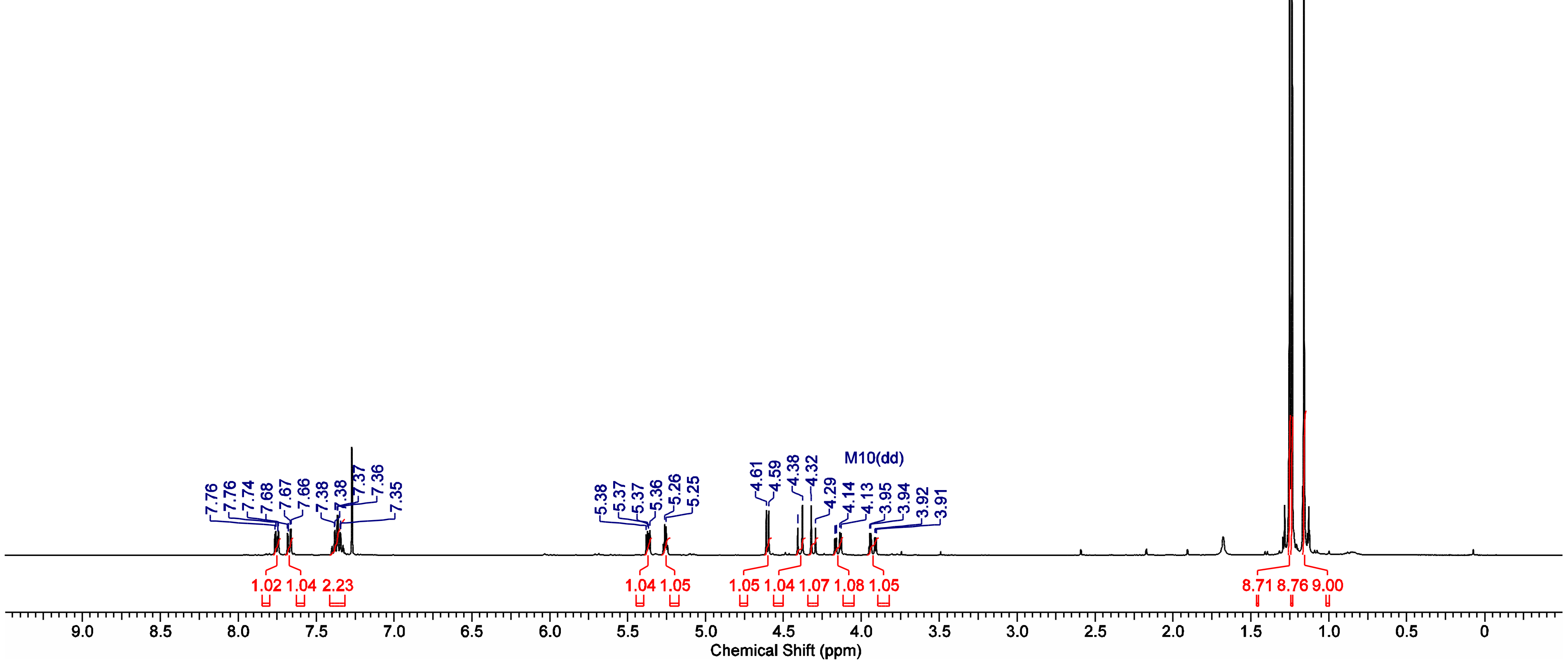

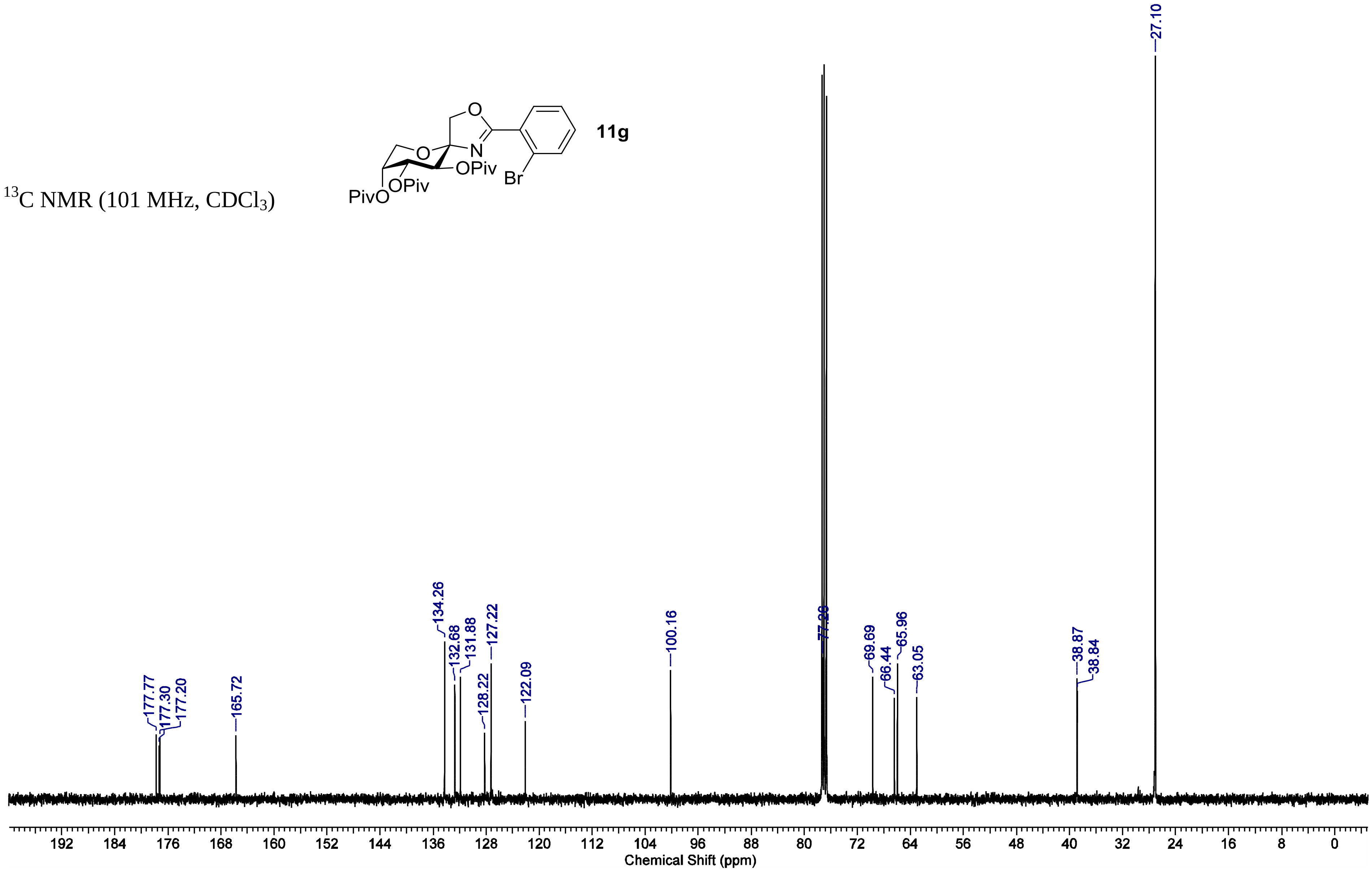
11g
OPiv
PivO
OPiv
Br
O
N
^{13}C NMR (101 MHz, CDCl$_3$)
177.77
177.30
177.20
165.72
134.26
132.68
131.88
128.22
127.22
122.09
100.16
69.69
66.44
65.96
63.05
38.87
38.84
27.10
Chemical Shift (ppm)

10h

^{1}H NMR (400 MHz, $CDCl_3$)

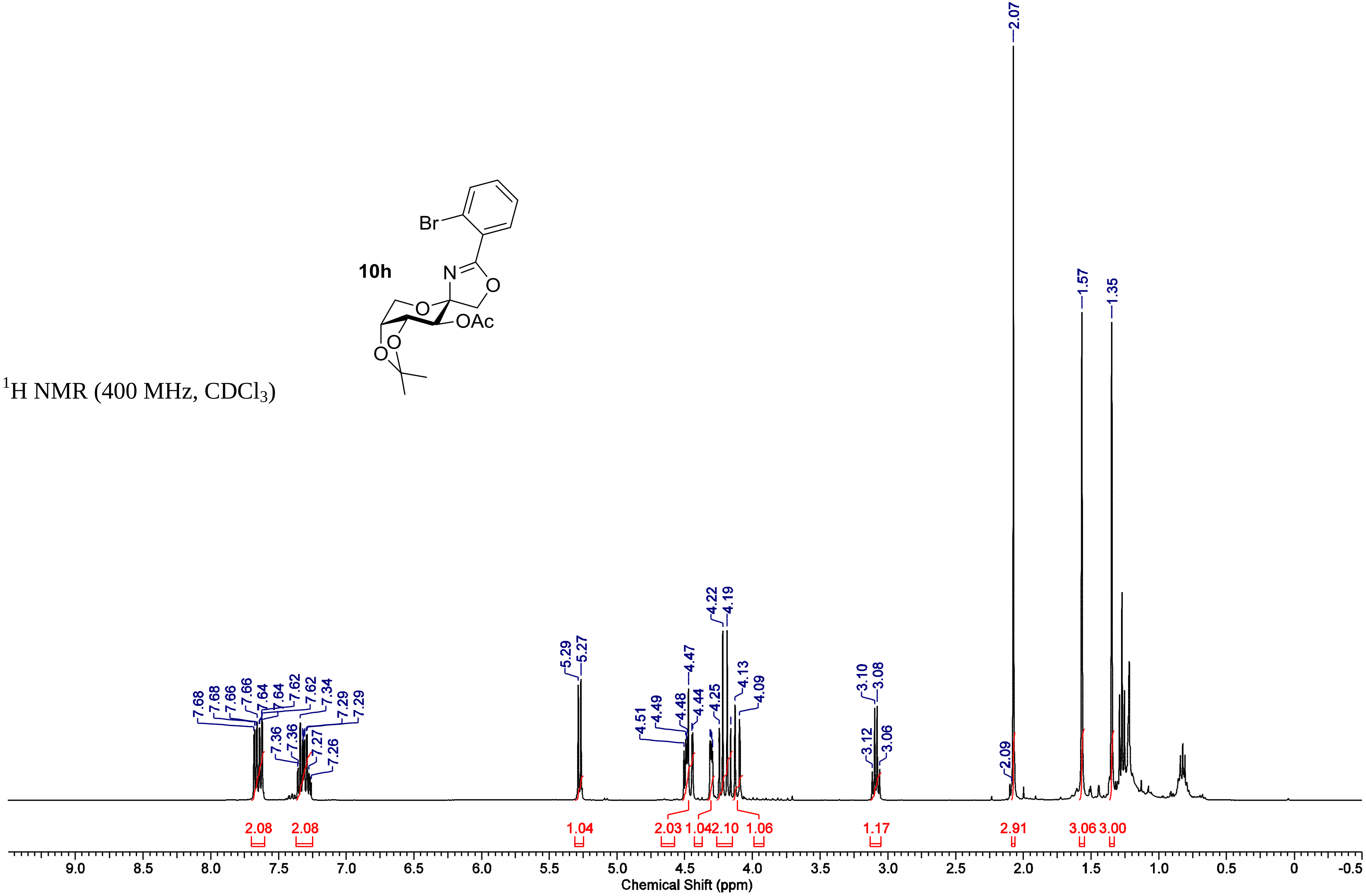

^{13}C NMR (101 MHz, $CDCl_3$)

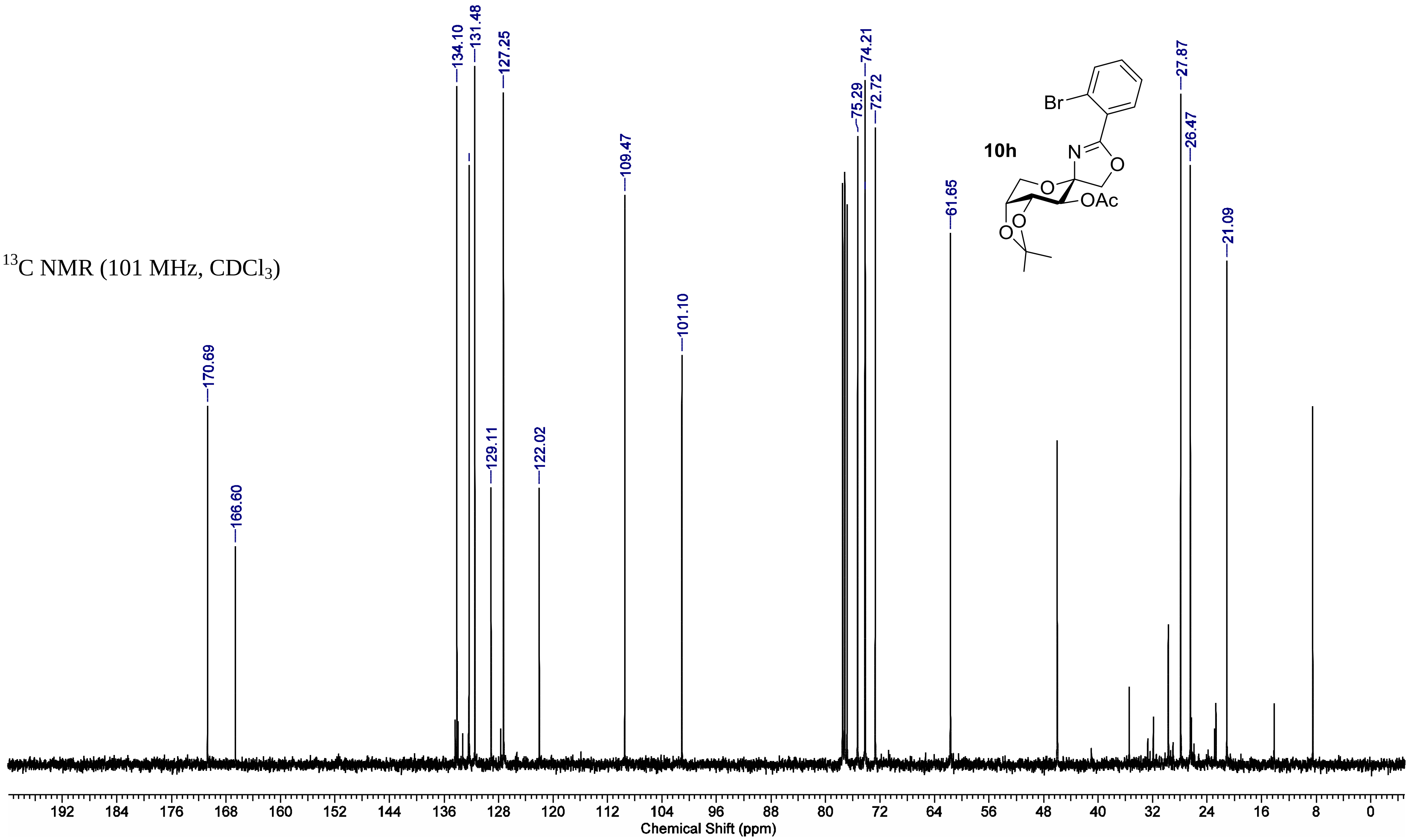

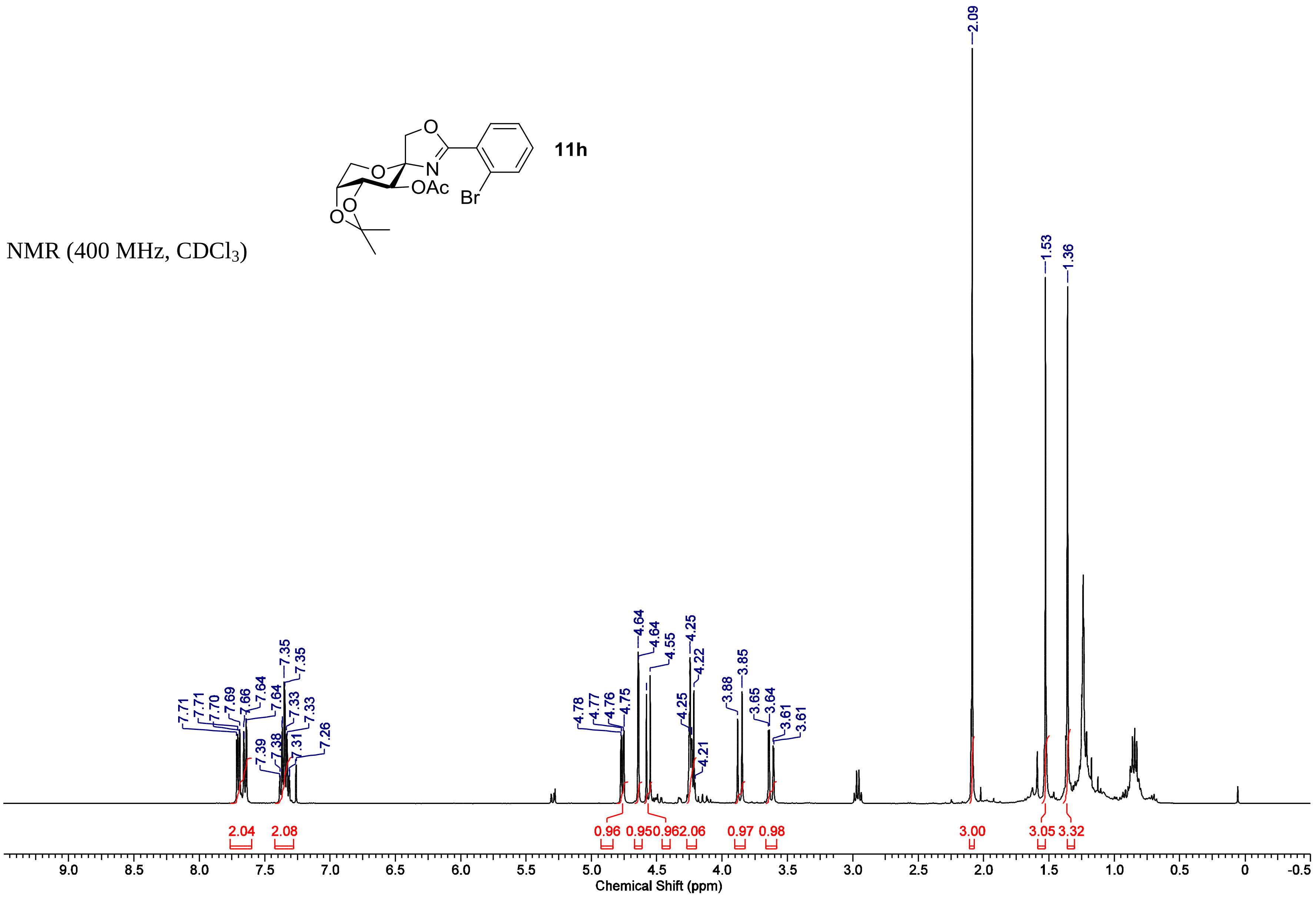

^{1}H NMR (400 MHz, $CDCl_3$)

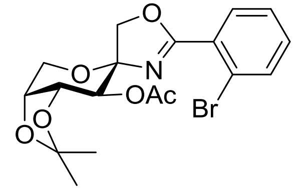

11h

^{13}C NMR (101 MHz, $CDCl_3$)

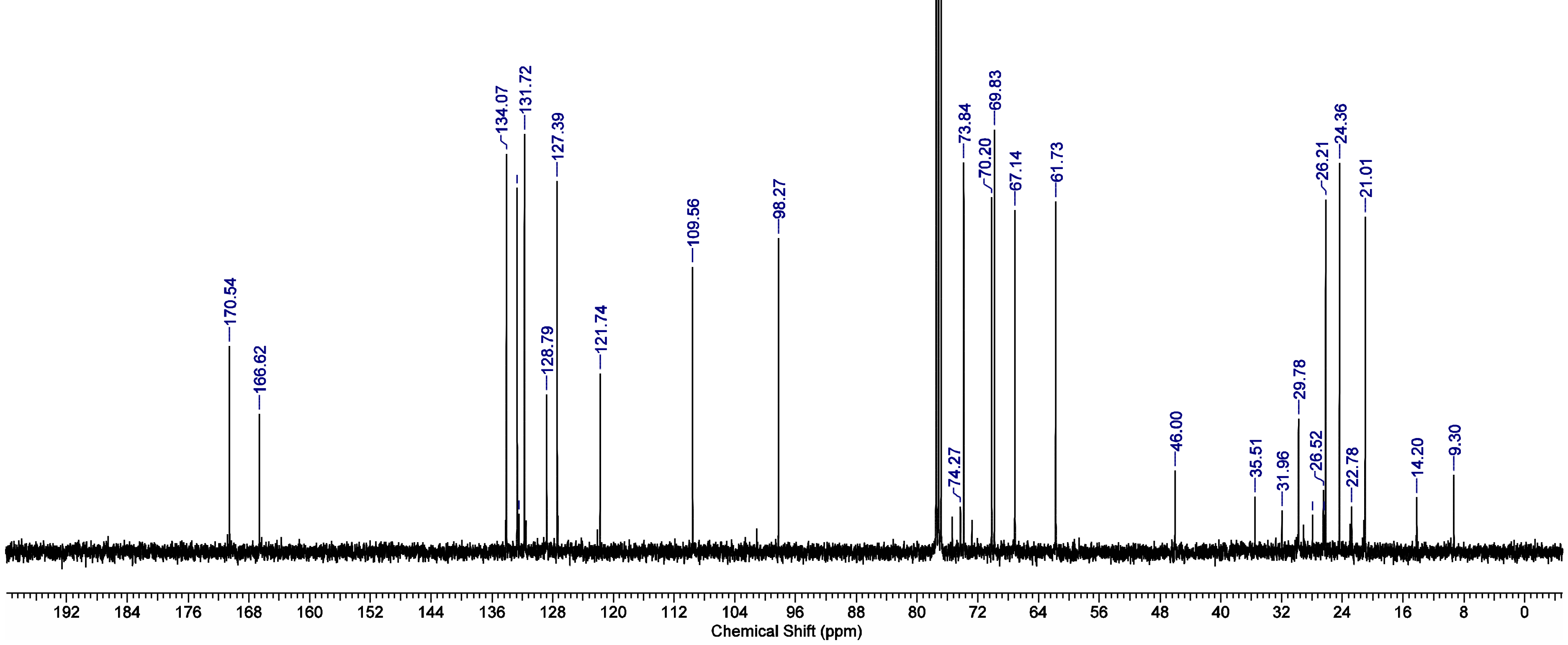

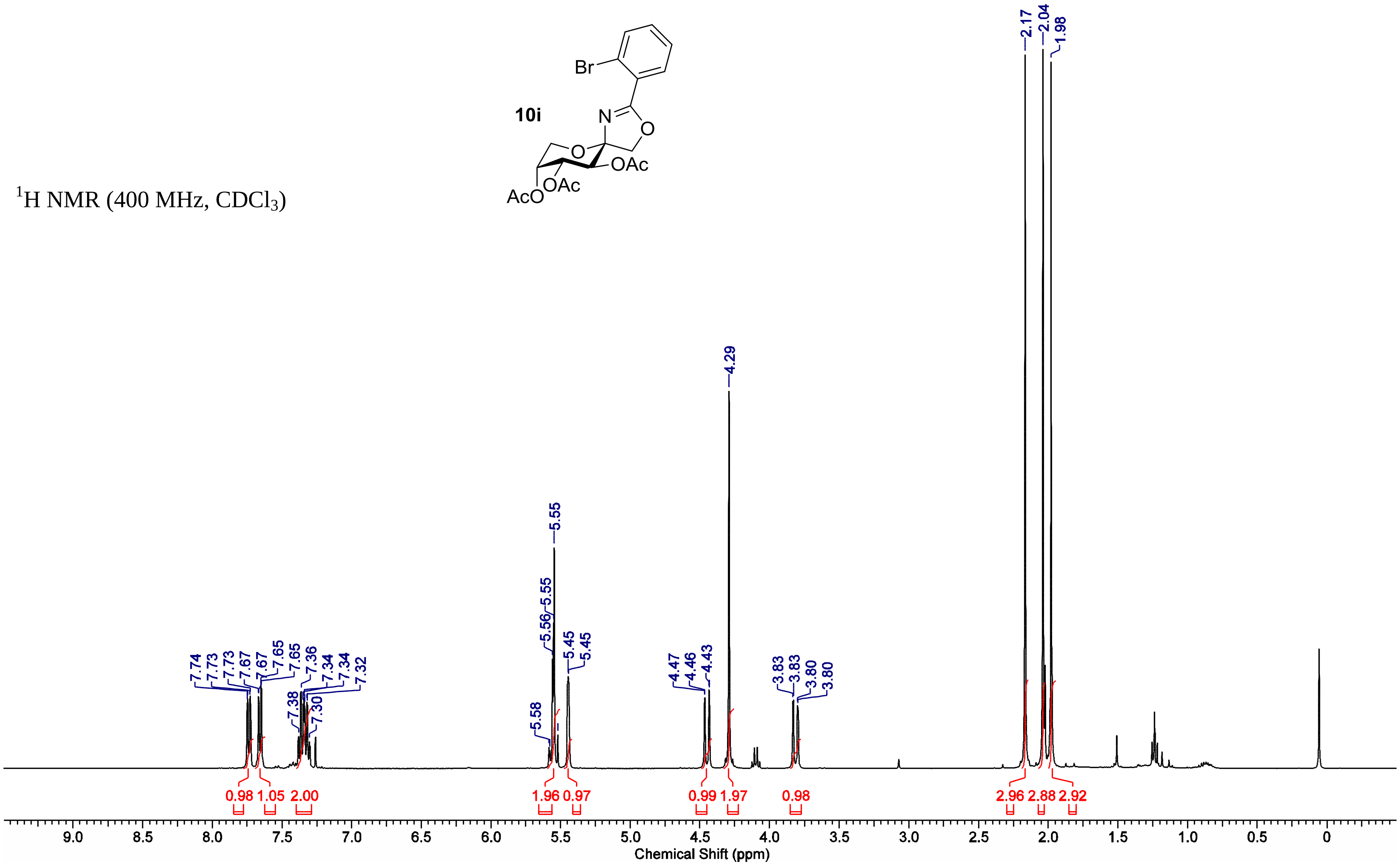
10i
Br
N
O
O
OAc
OAc
AcO
^{1}H NMR (400 MHz, CDCl3)
7.74
7.73
7.73
7.67
7.67
7.65
7.65
7.38
7.36
7.34
7.34
7.32
7.30
5.58
5.56
5.55
5.55
5.45
5.45
4.47
4.46
4.43
4.29
3.83
3.83
3.80
3.80
2.17
2.04
1.98
0.98
1.05
2.00
1.96
0.97
0.99
1.97
0.98
2.96
2.88
2.92
9.0
8.5
8.0
7.5
7.0
6.5
6.0
5.5
5.0
4.5
4.0
3.5
3.0
2.5
2.0
1.5
1.0
0.5
0
Chemical Shift (ppm)

^{13}C NMR (101 MHz, $CDCl_3$)

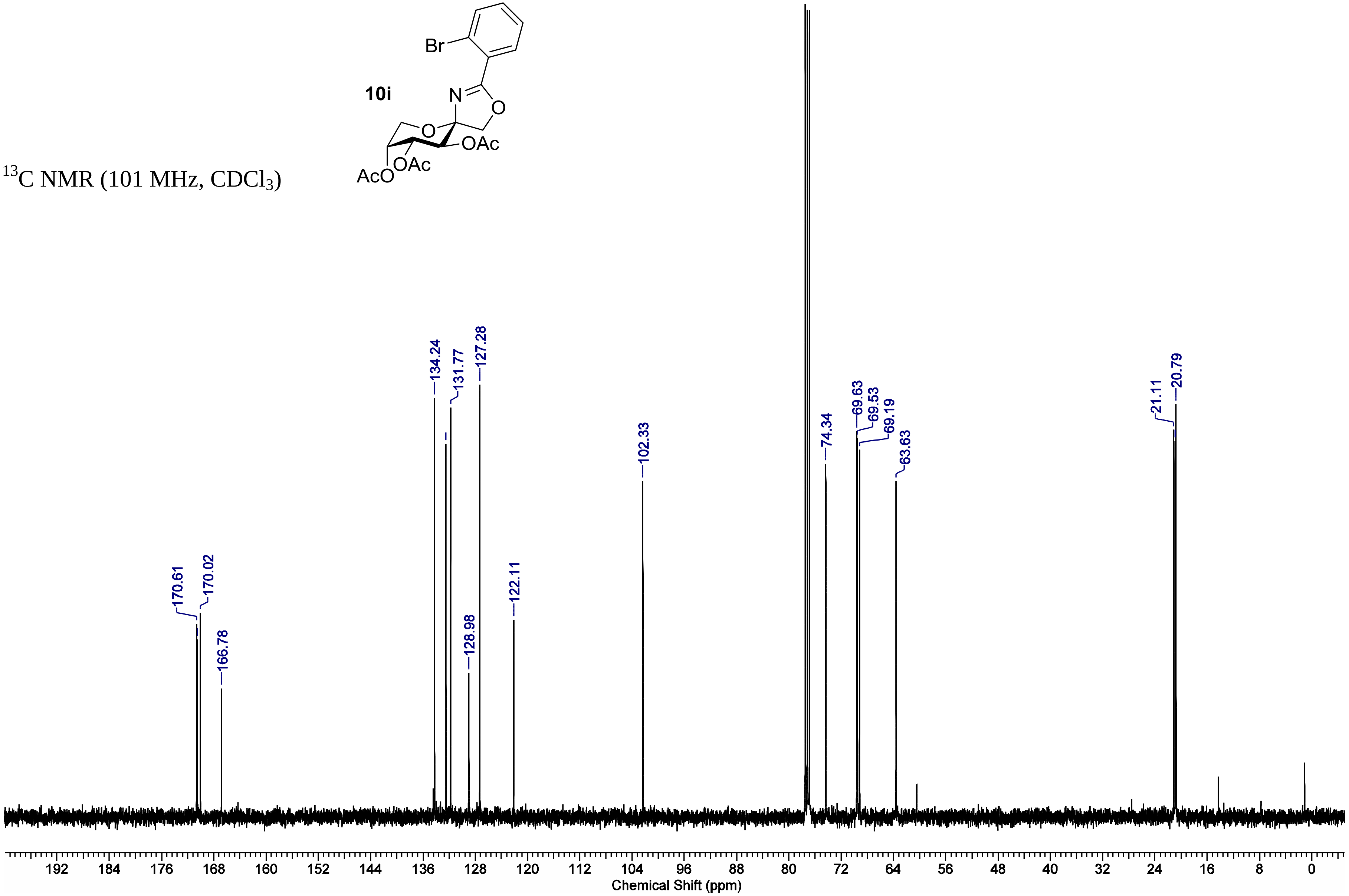

^1H NMR (400 MHz, $CDCl_3$)

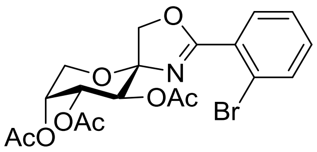

11i

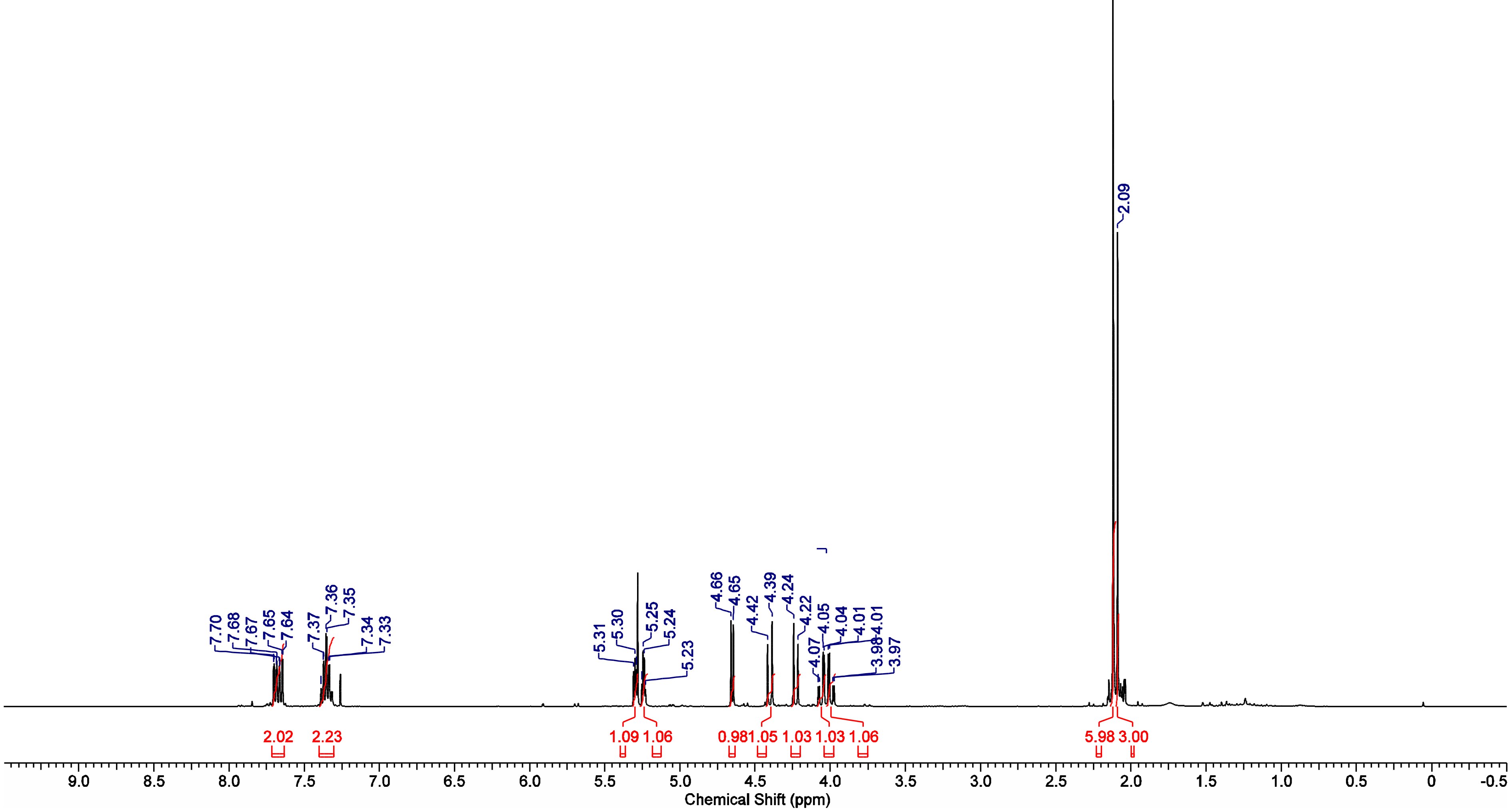

^{13}C NMR (101 MHz, $CDCl_3$)

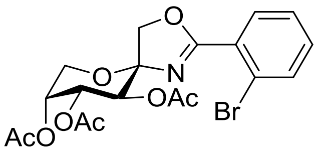

11i

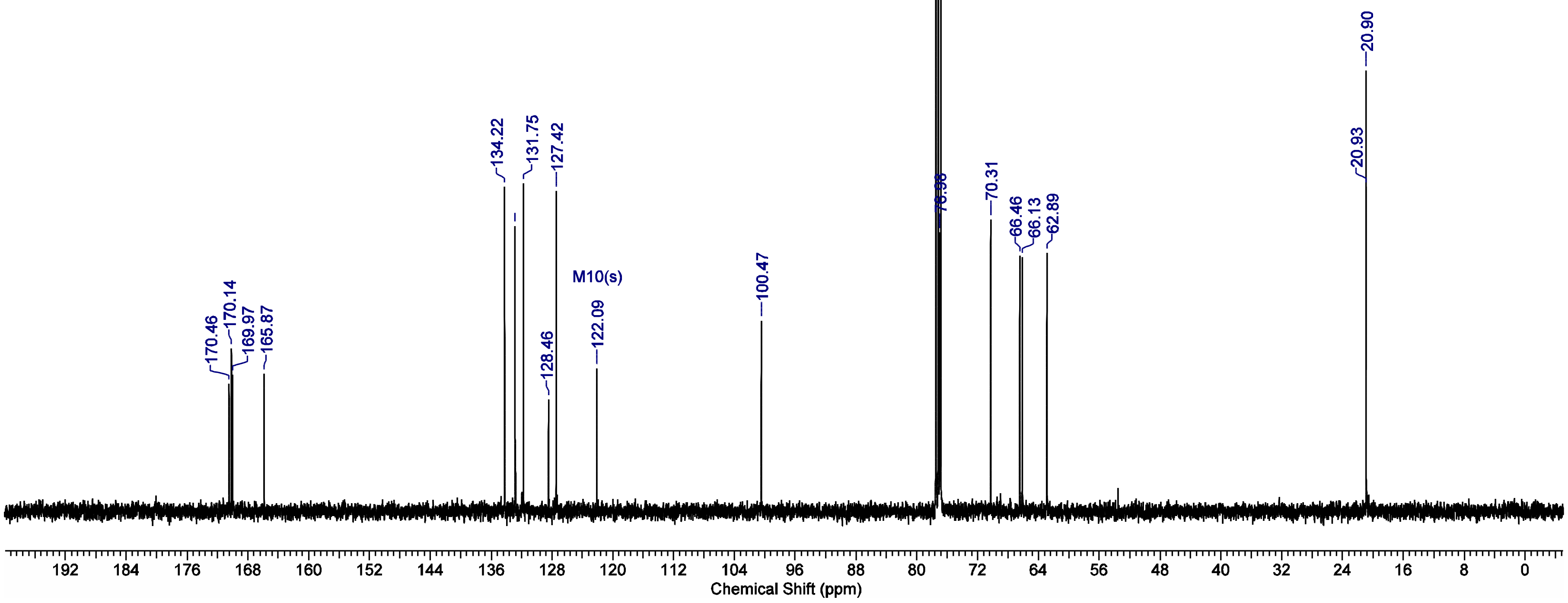

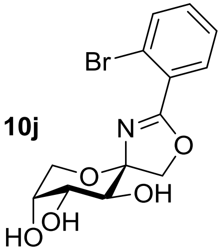

10j

^{1}H NMR (400 MHz, MeOD)

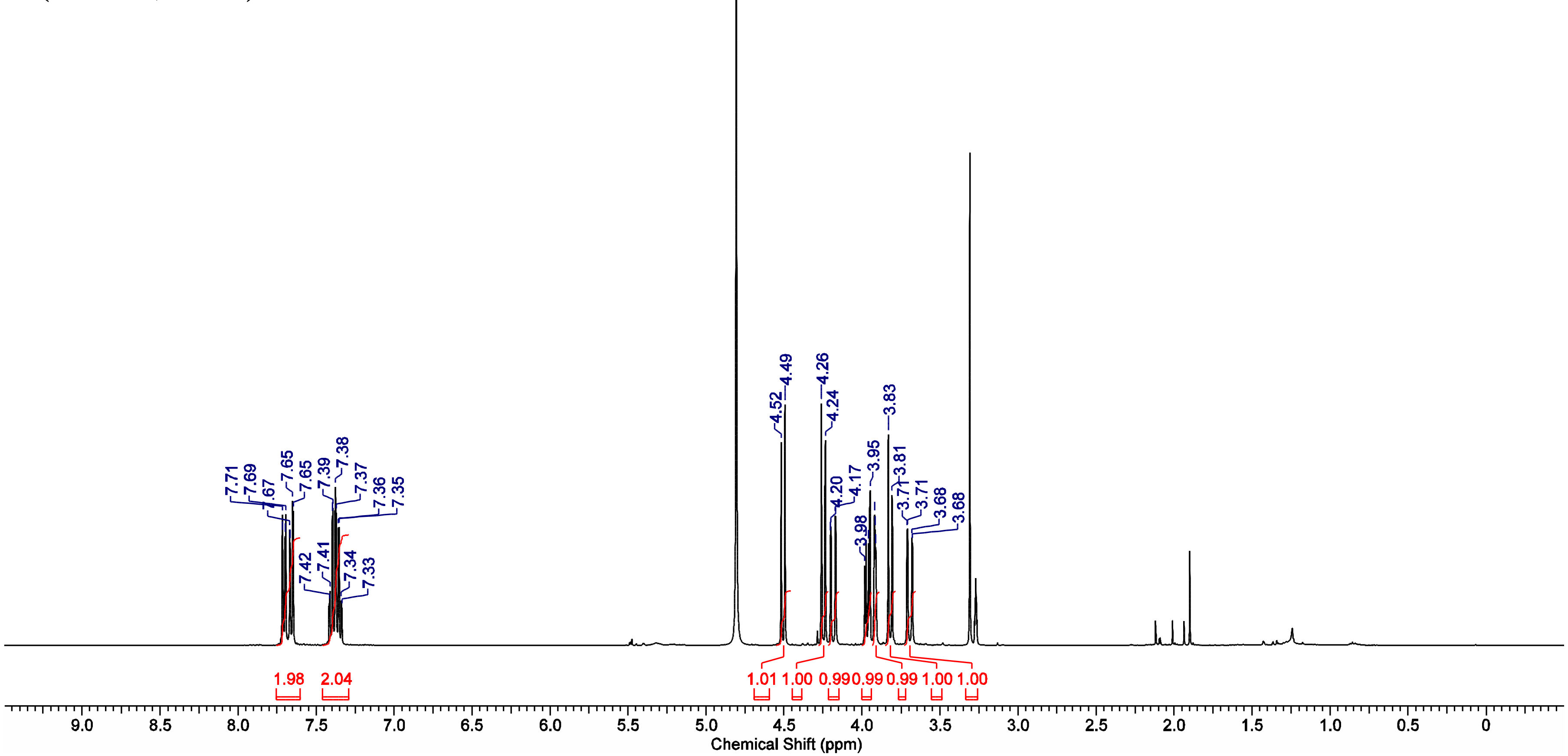

^{13}C NMR (101 MHz, MeOD)

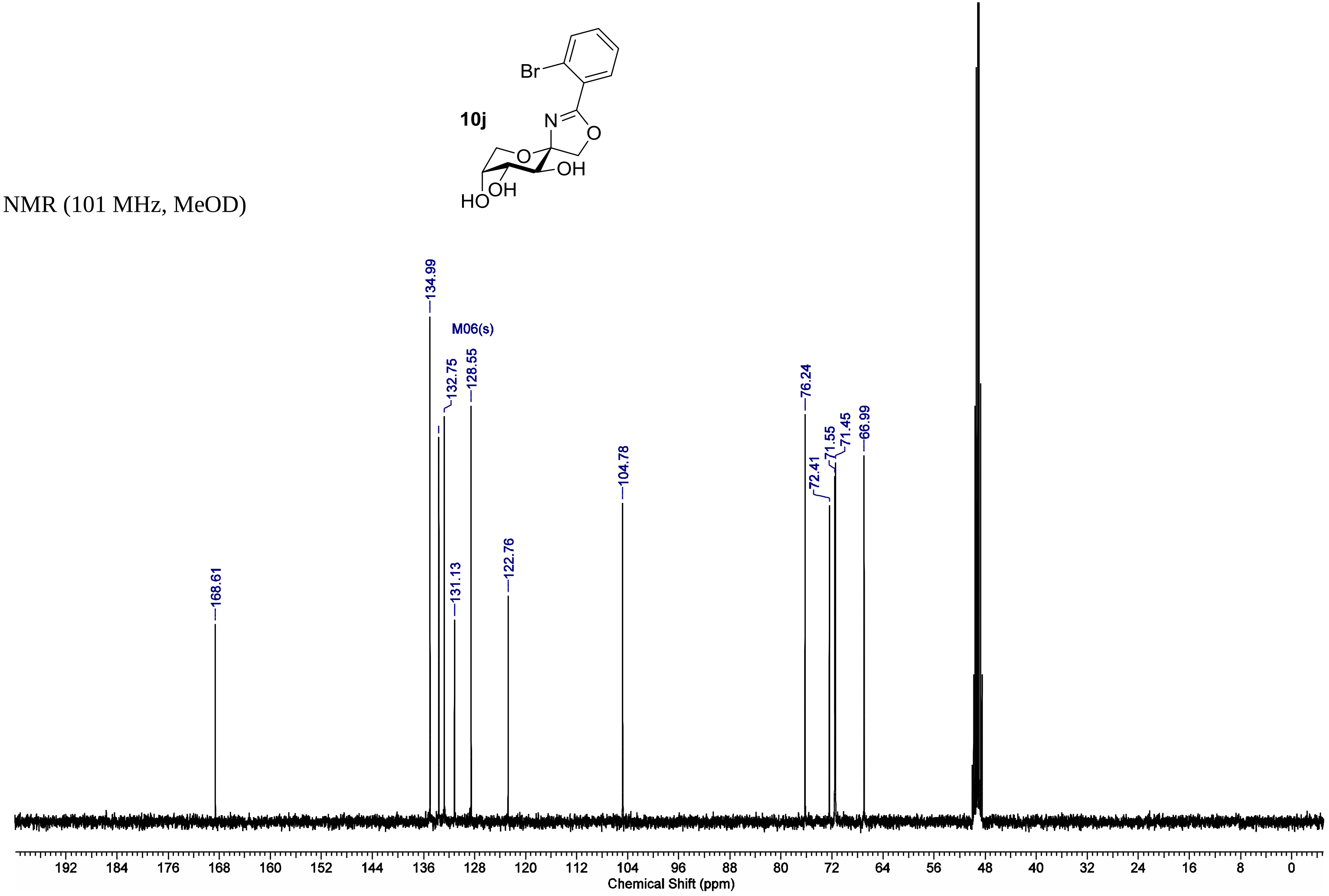

^{1}H NMR (400 MHz, $CDCl_3$)

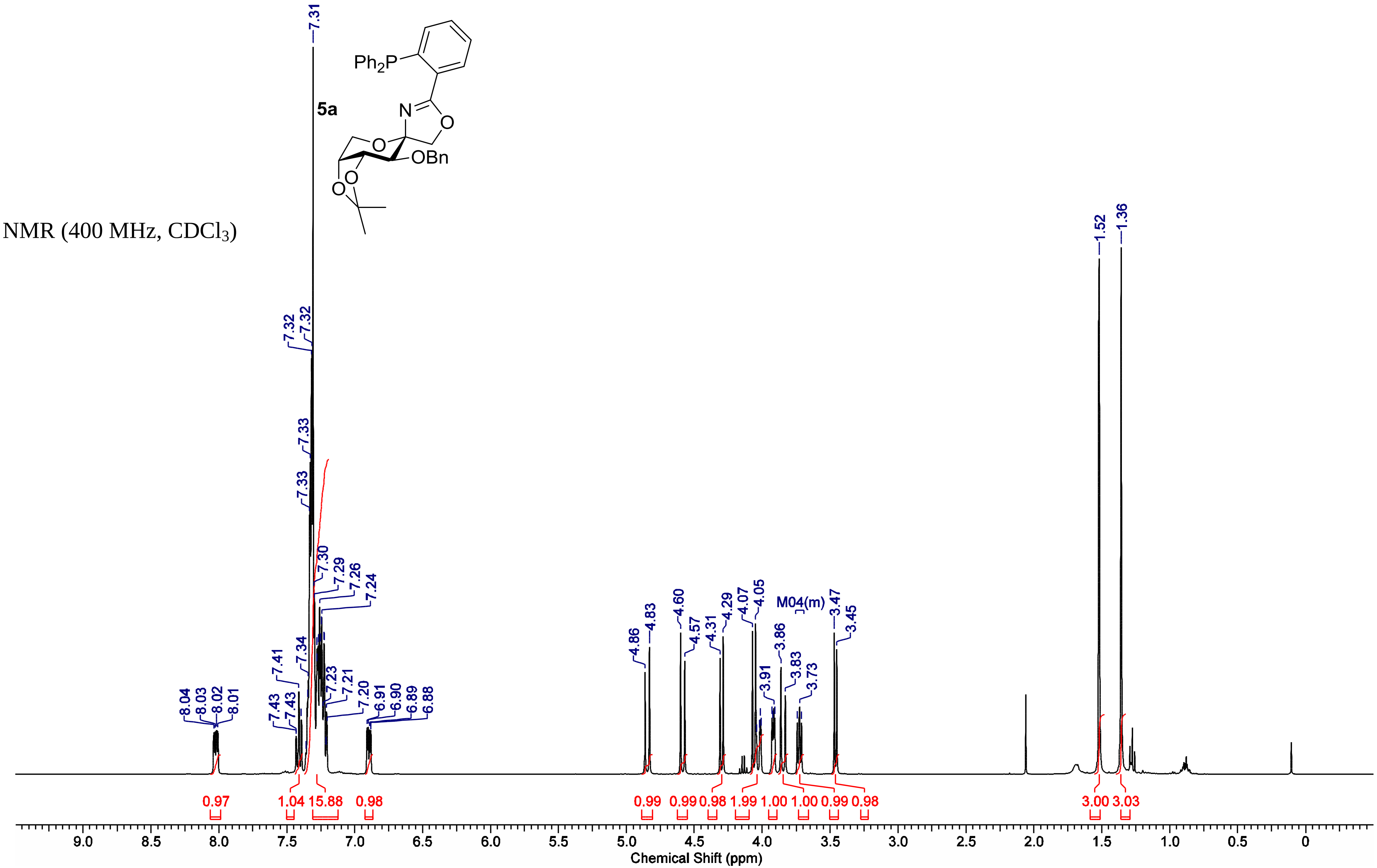

^{13}C NMR (101 MHz, $CDCl_3$)

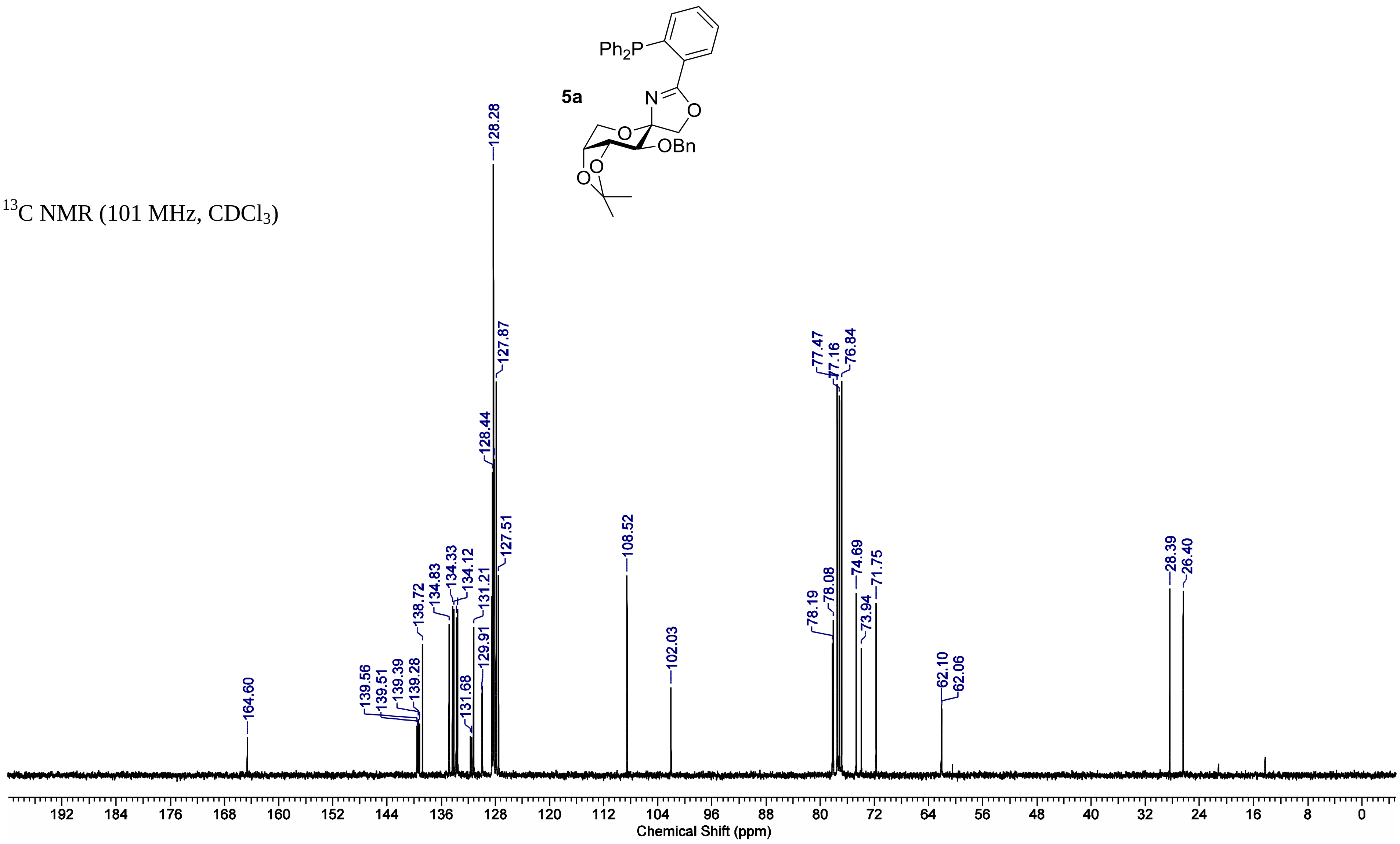

^{31}P NMR (162 MHz, $CDCl_3$)

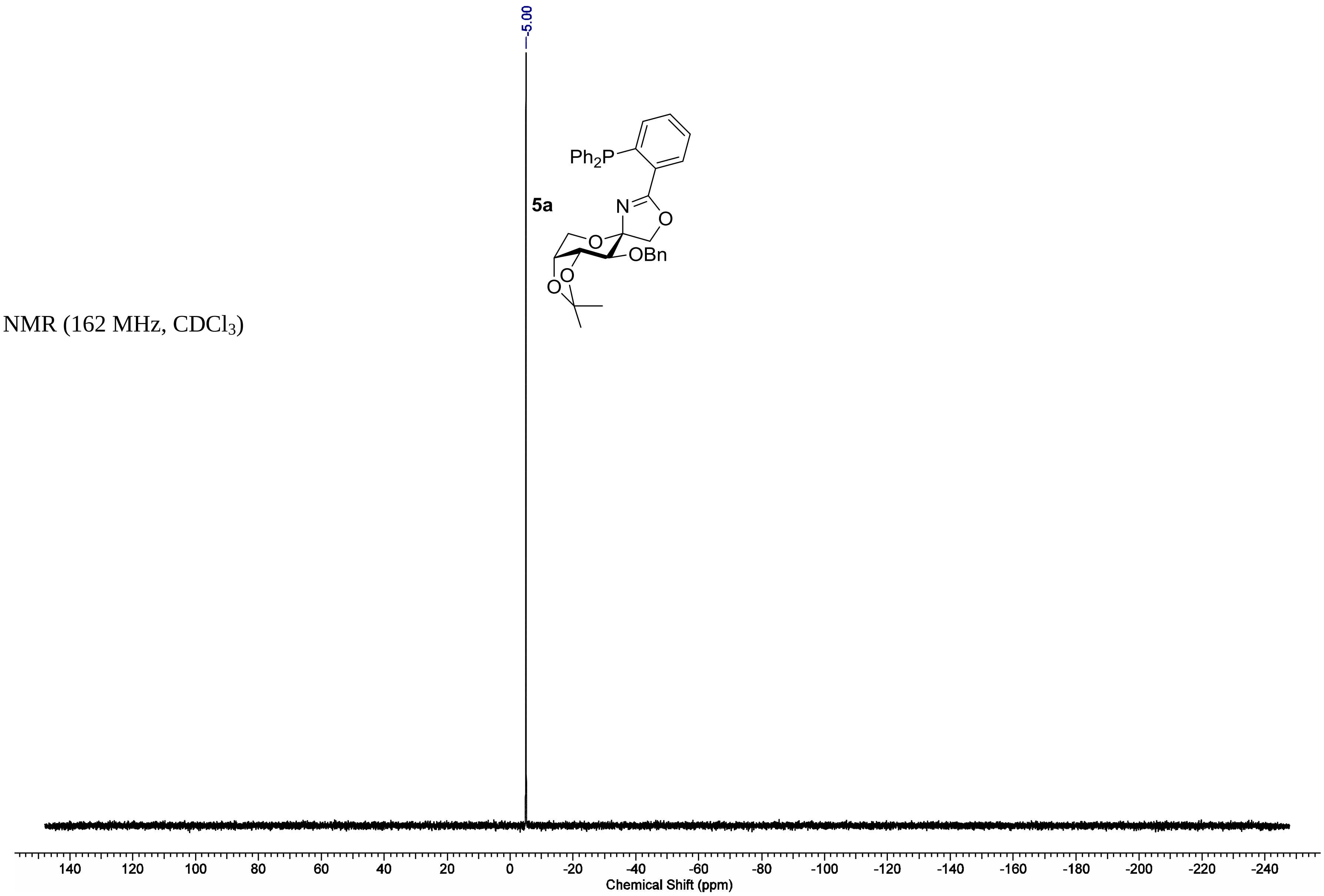

^{1}H NMR (400 MHz, $CDCl_3$)

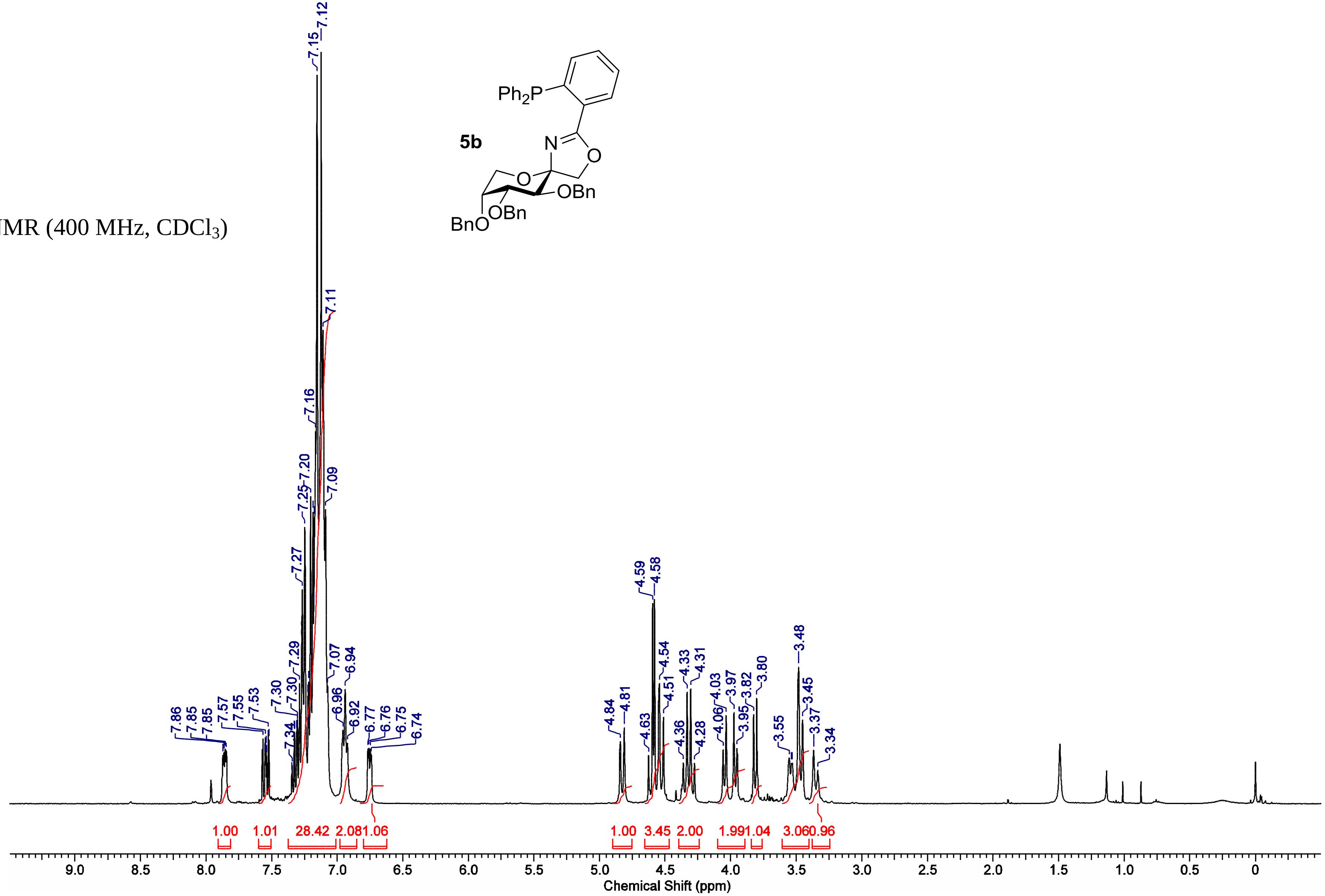

^{13}C NMR (101 MHz, $CDCl_3$)

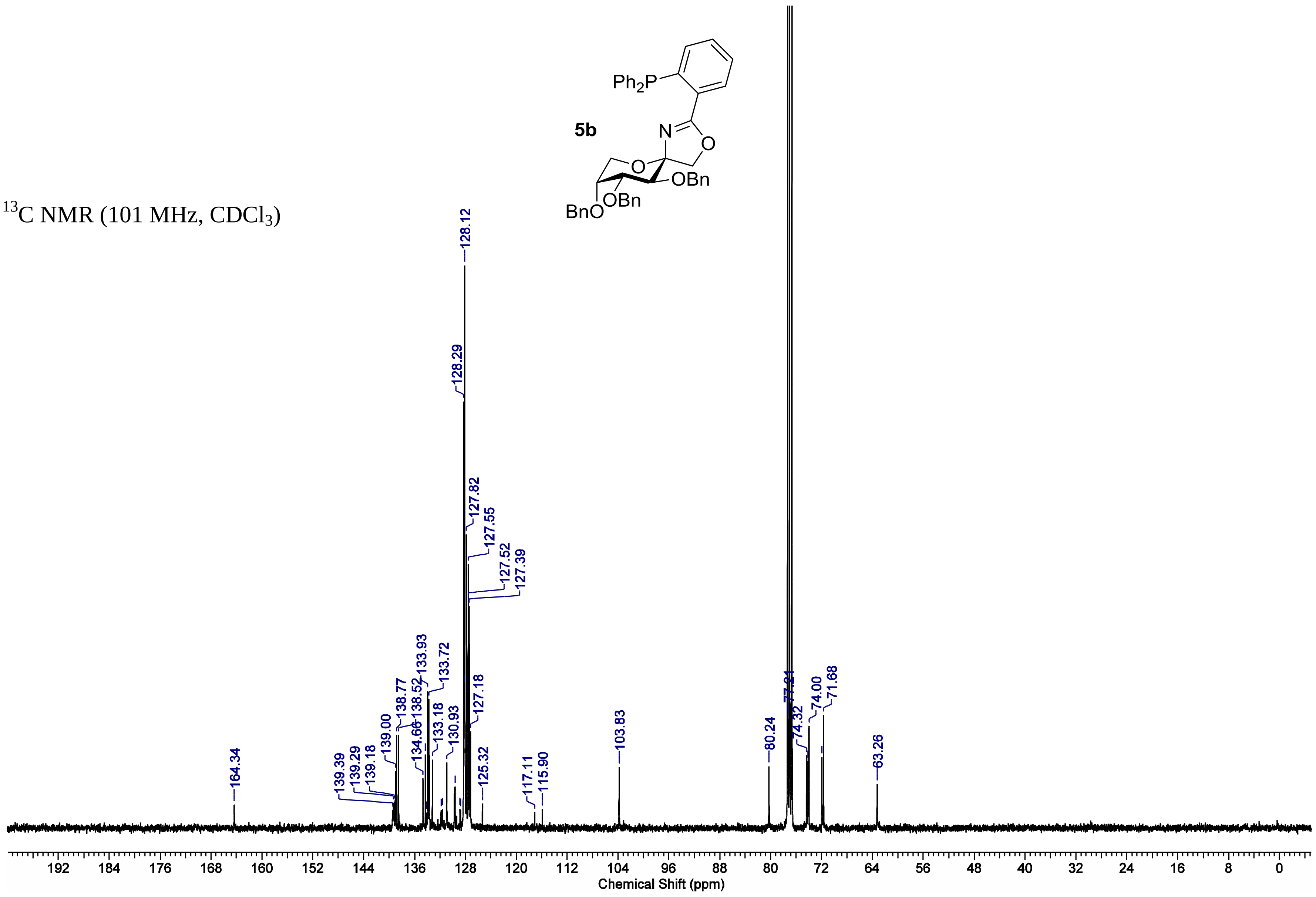

^{31}P NMR (162 MHz, $CDCl_3$)

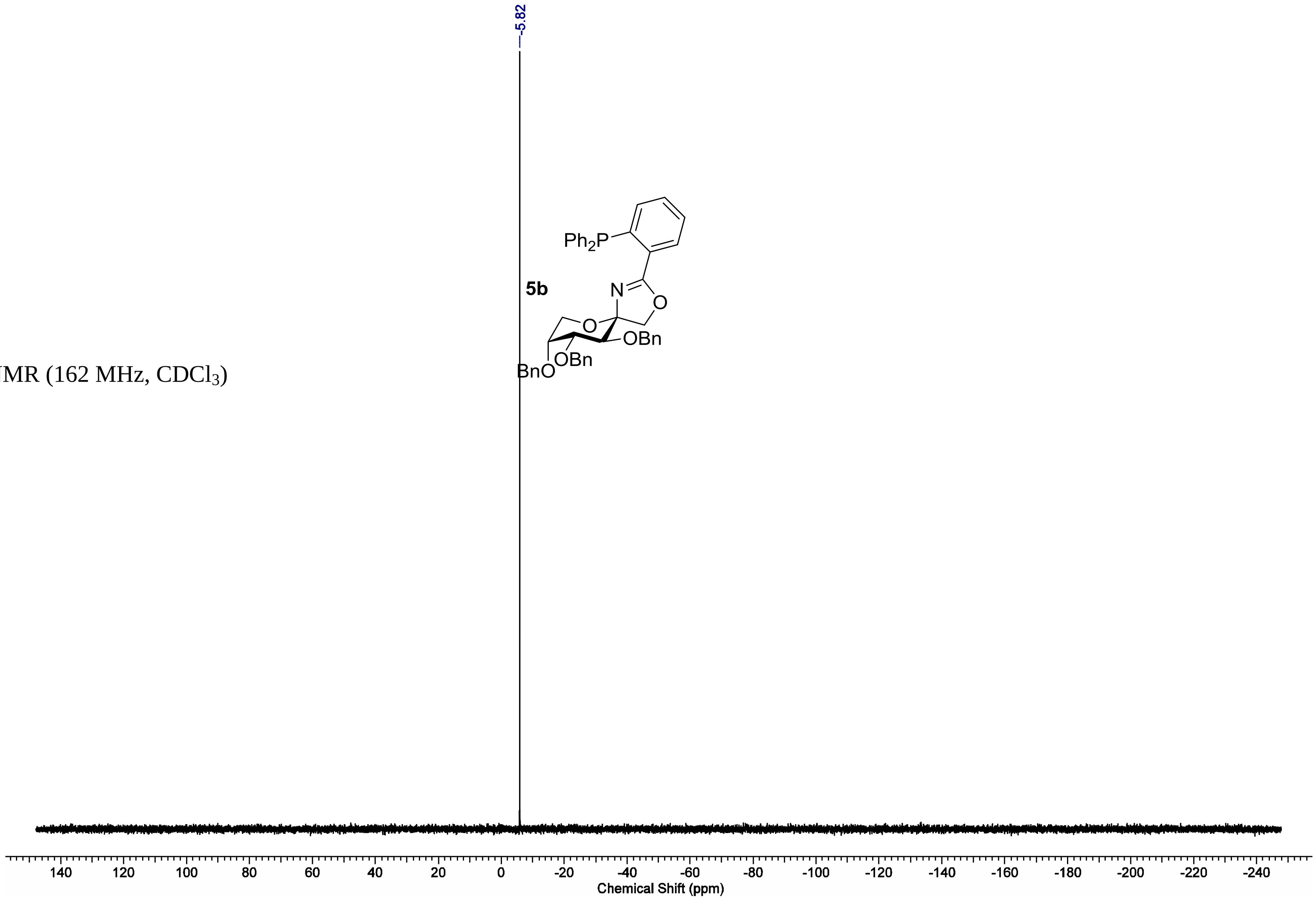

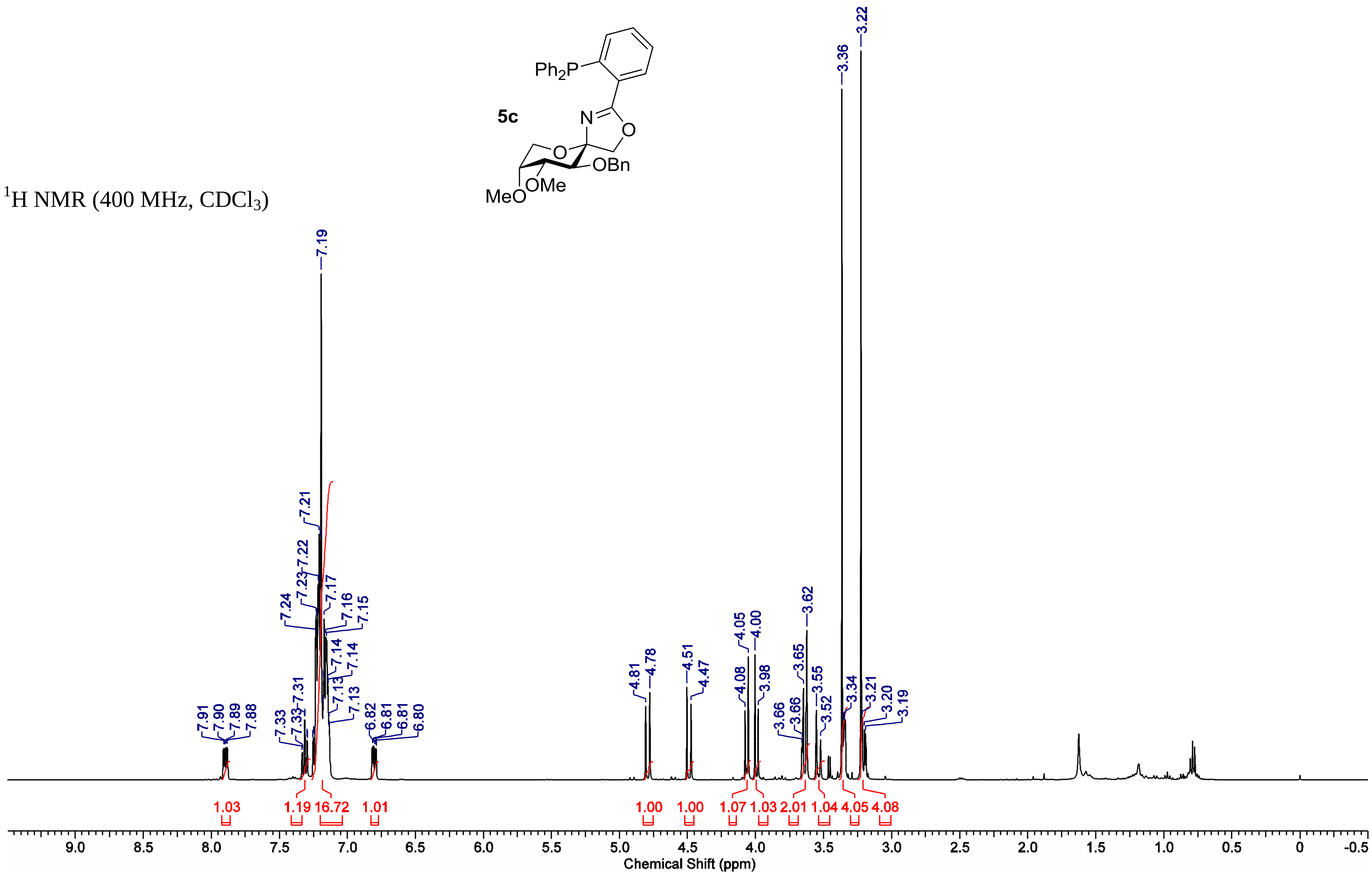
5c
Ph2P
N
O
OBn
OMe
MeO
^{1}H NMR (400 MHz, CDCl3)
Chemical Shift (ppm)

5c

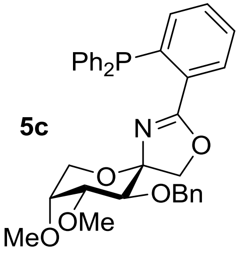

^{13}C NMR (101 MHz, $CDCl_3$)

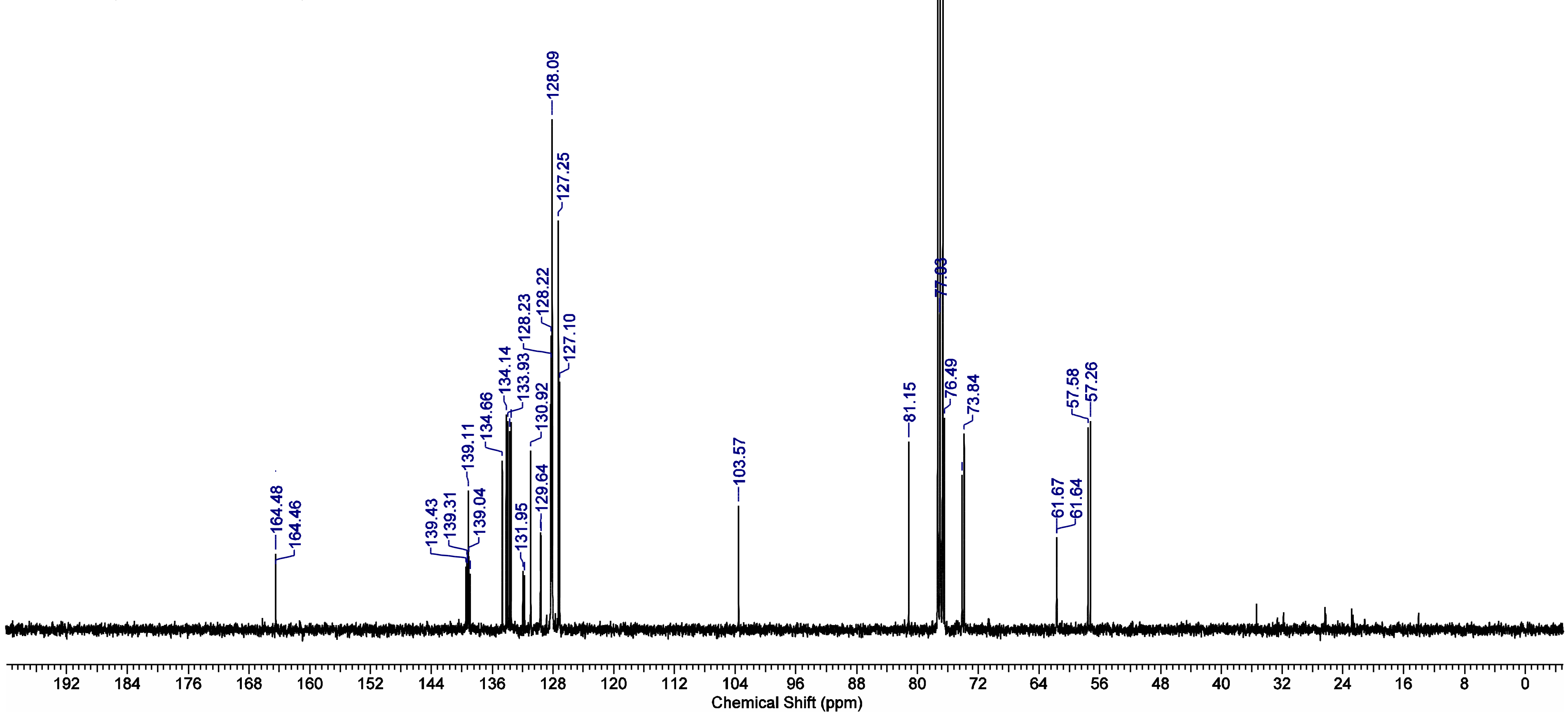

^{31}P NMR (162 MHz, $CDCl_3$)

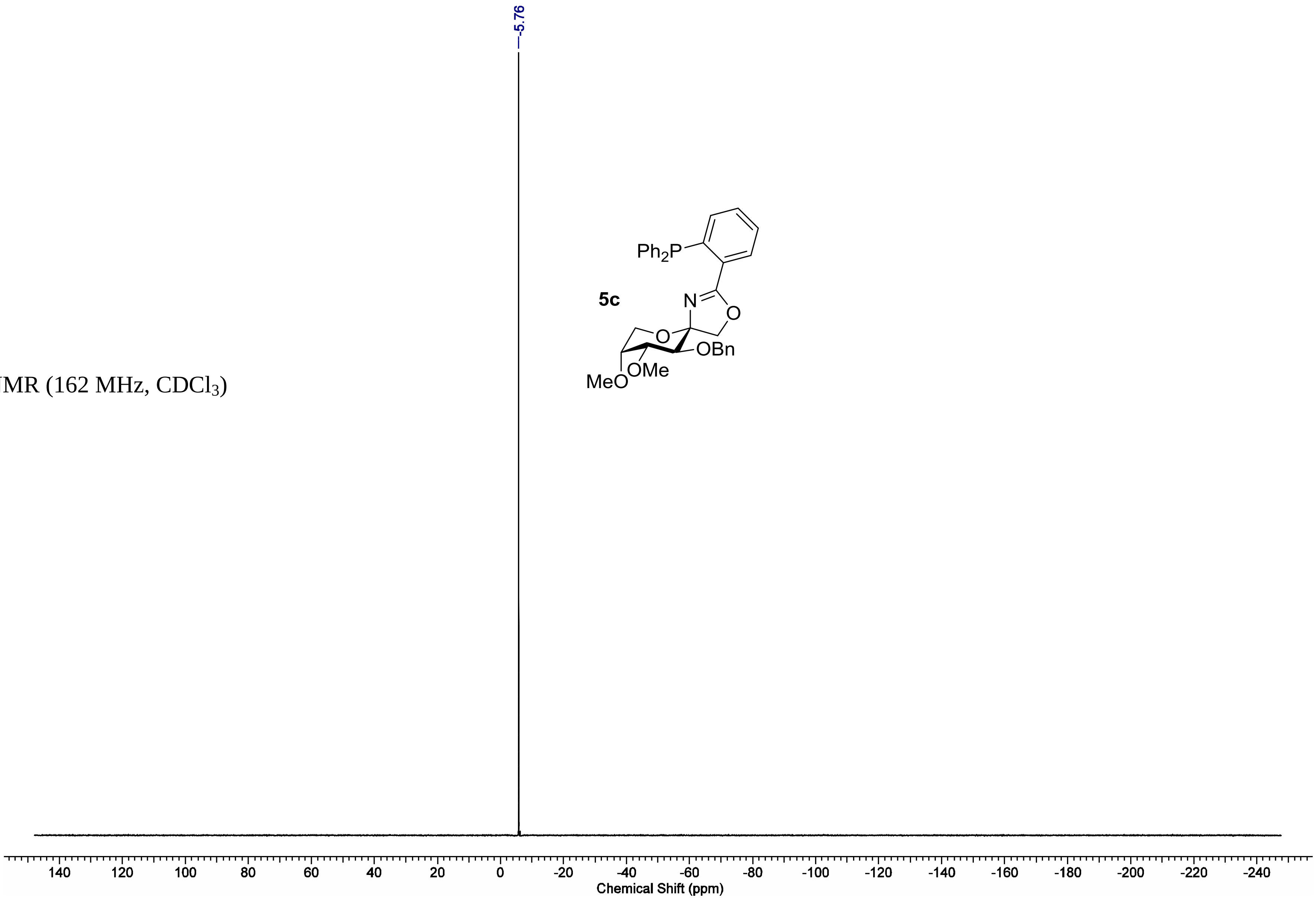

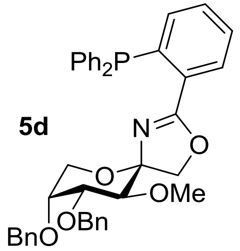

^{1}H NMR (400 MHz, $CDCl_3$)

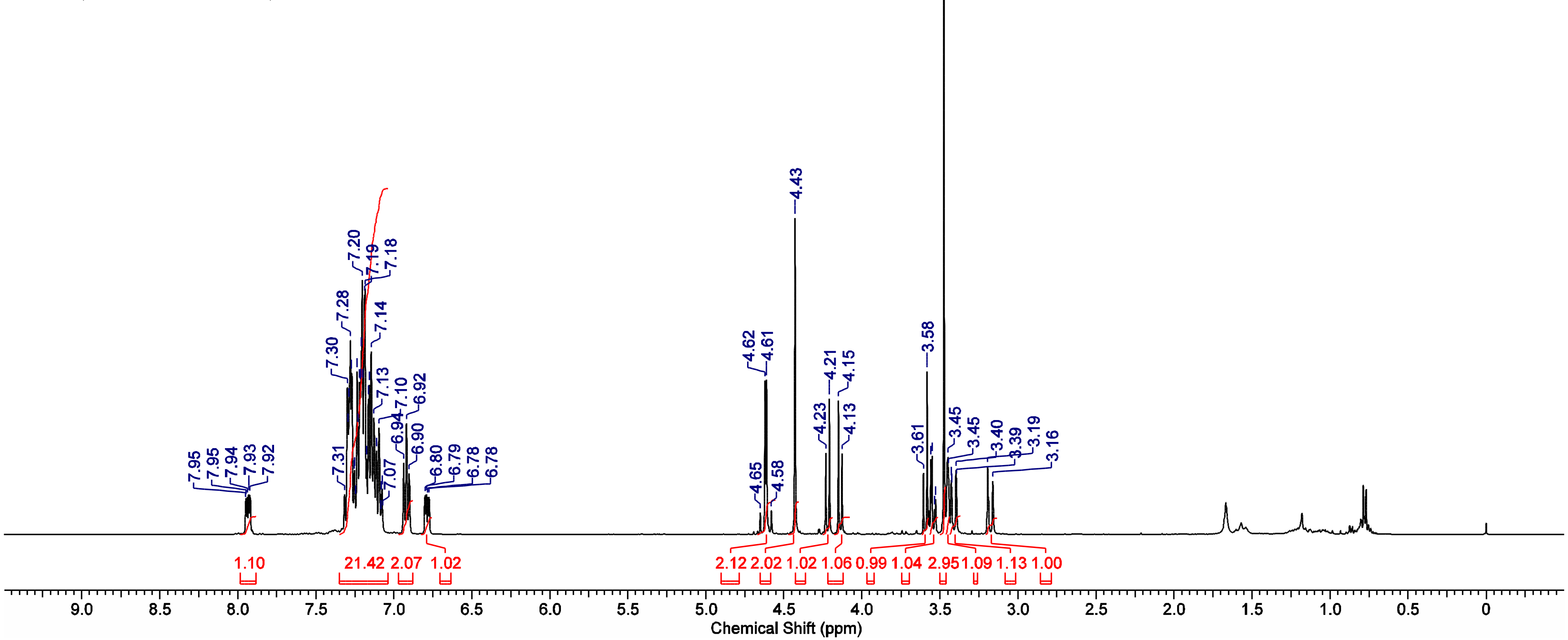

^{13}C NMR (101 MHz, $CDCl_3$)

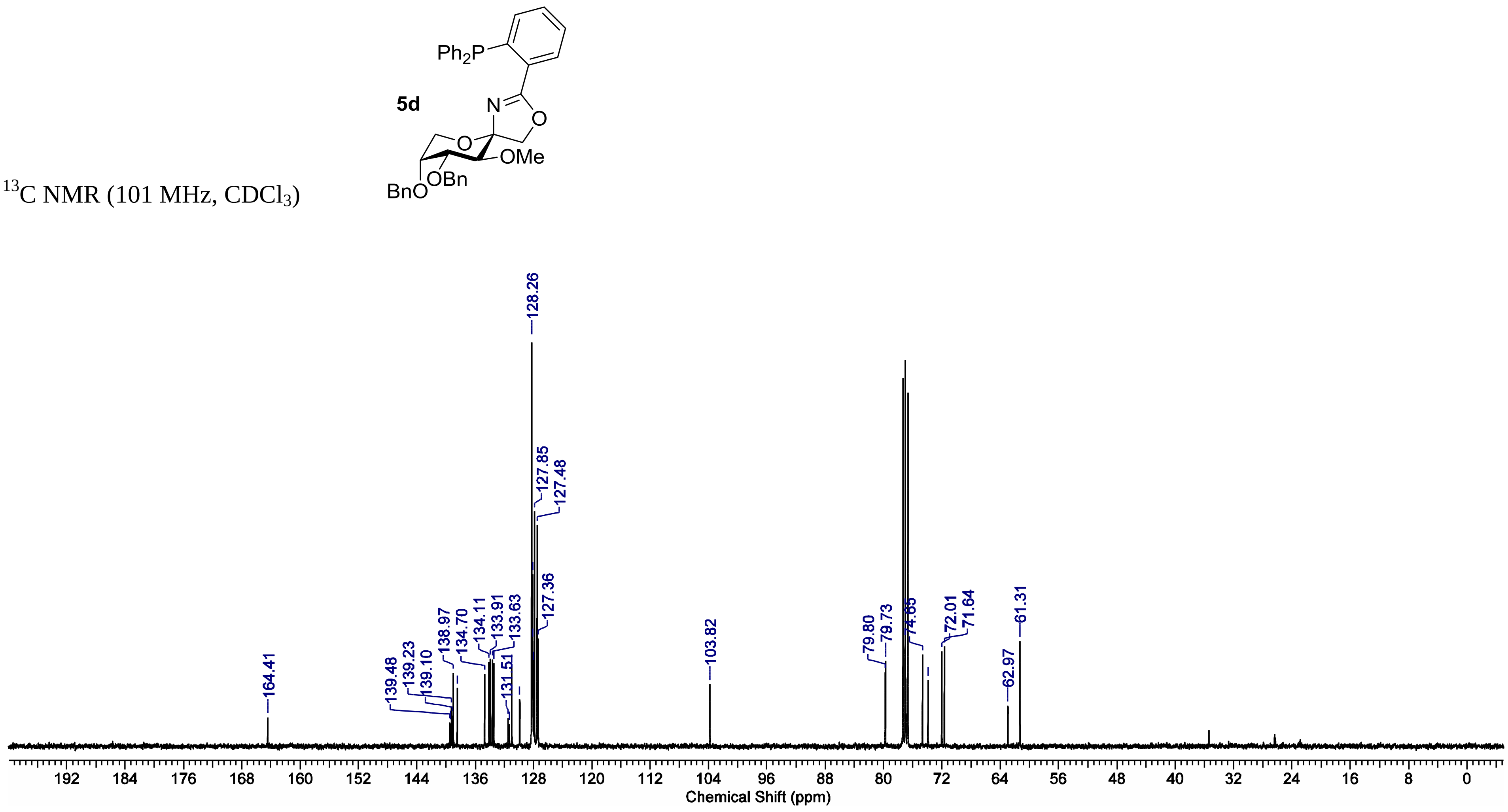

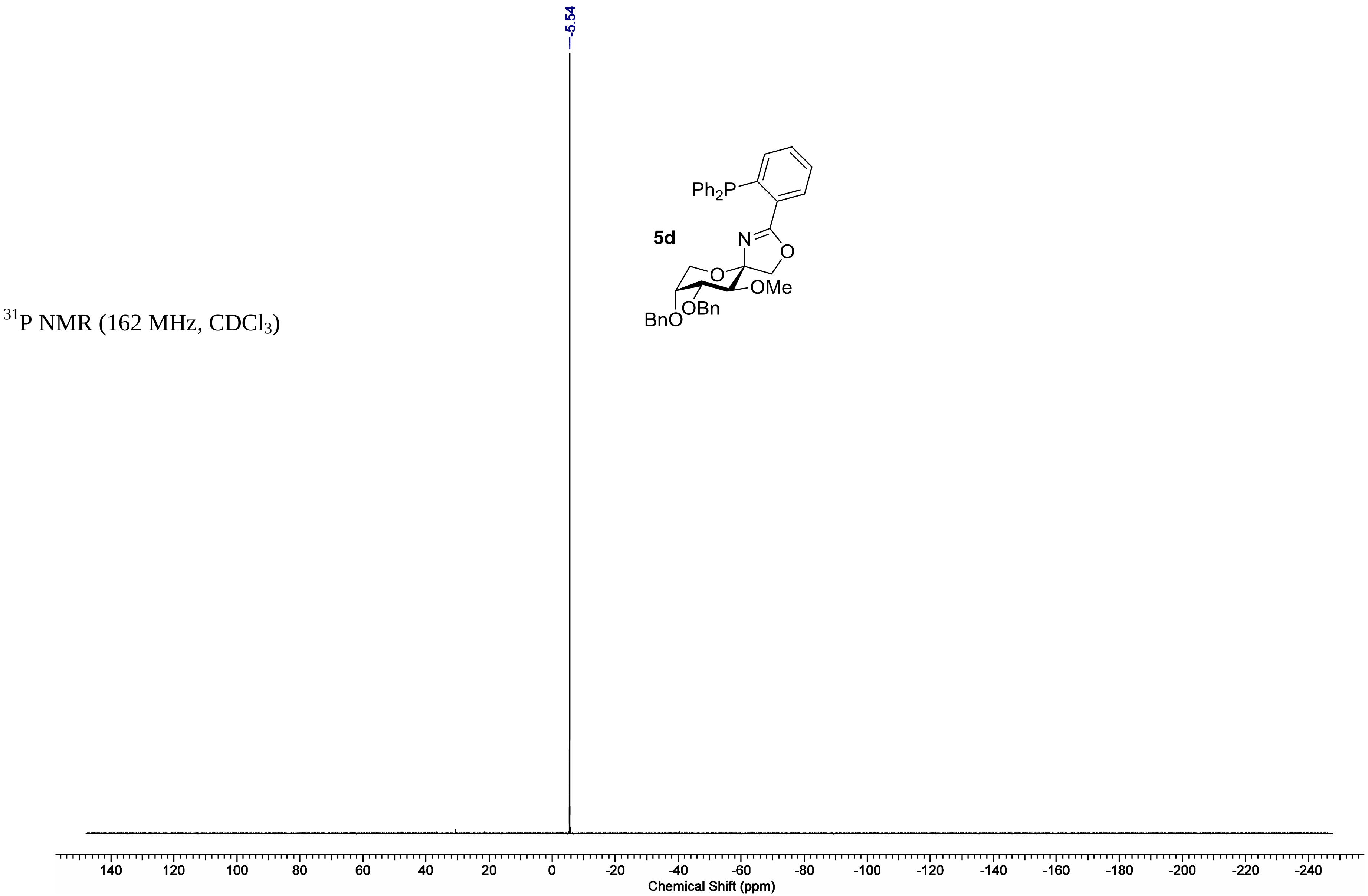
-5.54
31P NMR (162 MHz, CDCl3)
5d
Ph2P
N
O
OMe
OBn
BnO
140
120
100
80
60
40
20
0
-20
-40
-60
-80
-100
-120
-140
-160
-180
-200
-220
-240
Chemical Shift (ppm)

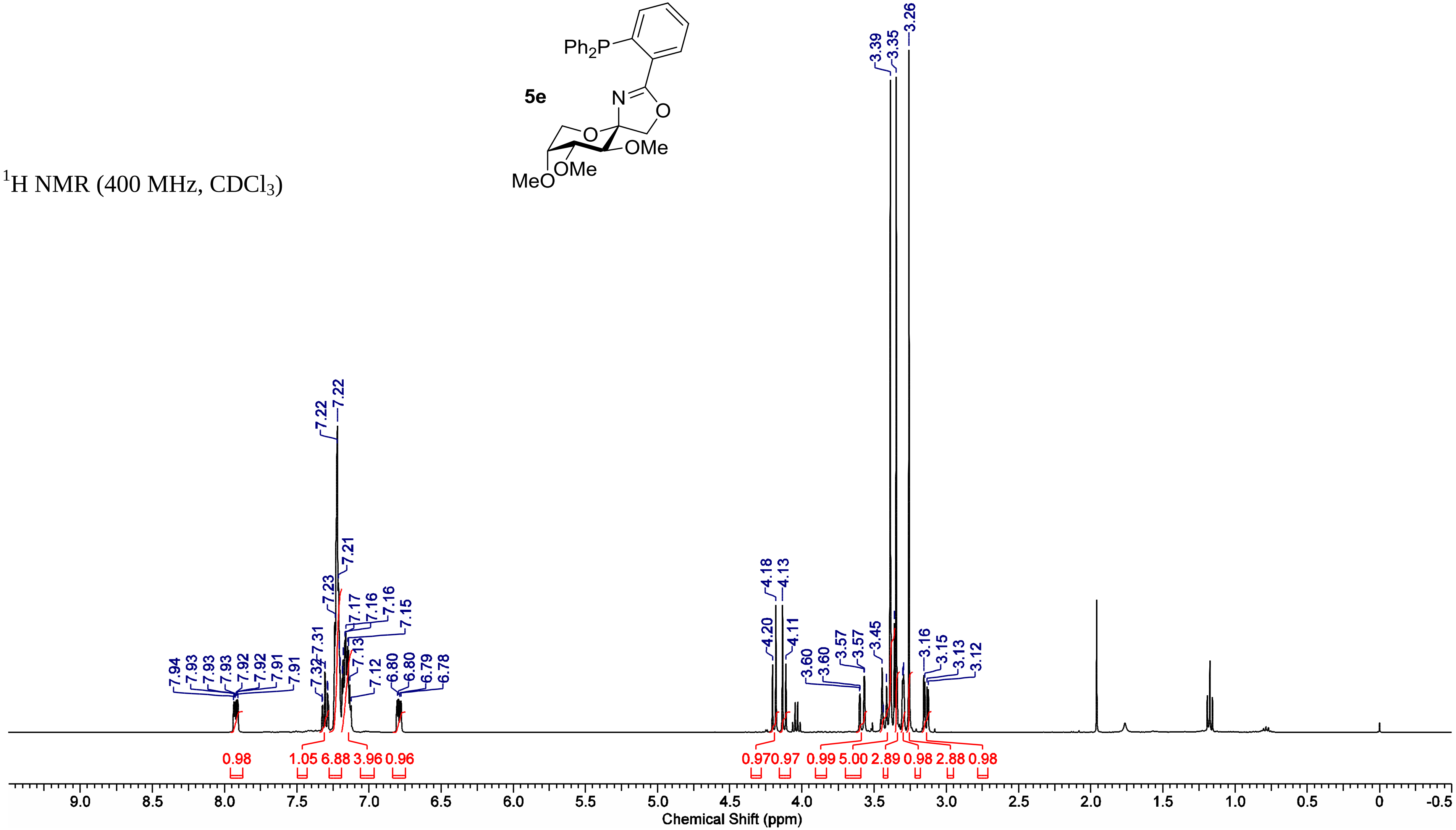

^{1}H NMR (400 MHz, $CDCl_3$)

^{13}C NMR (101 MHz, $CDCl_3$)

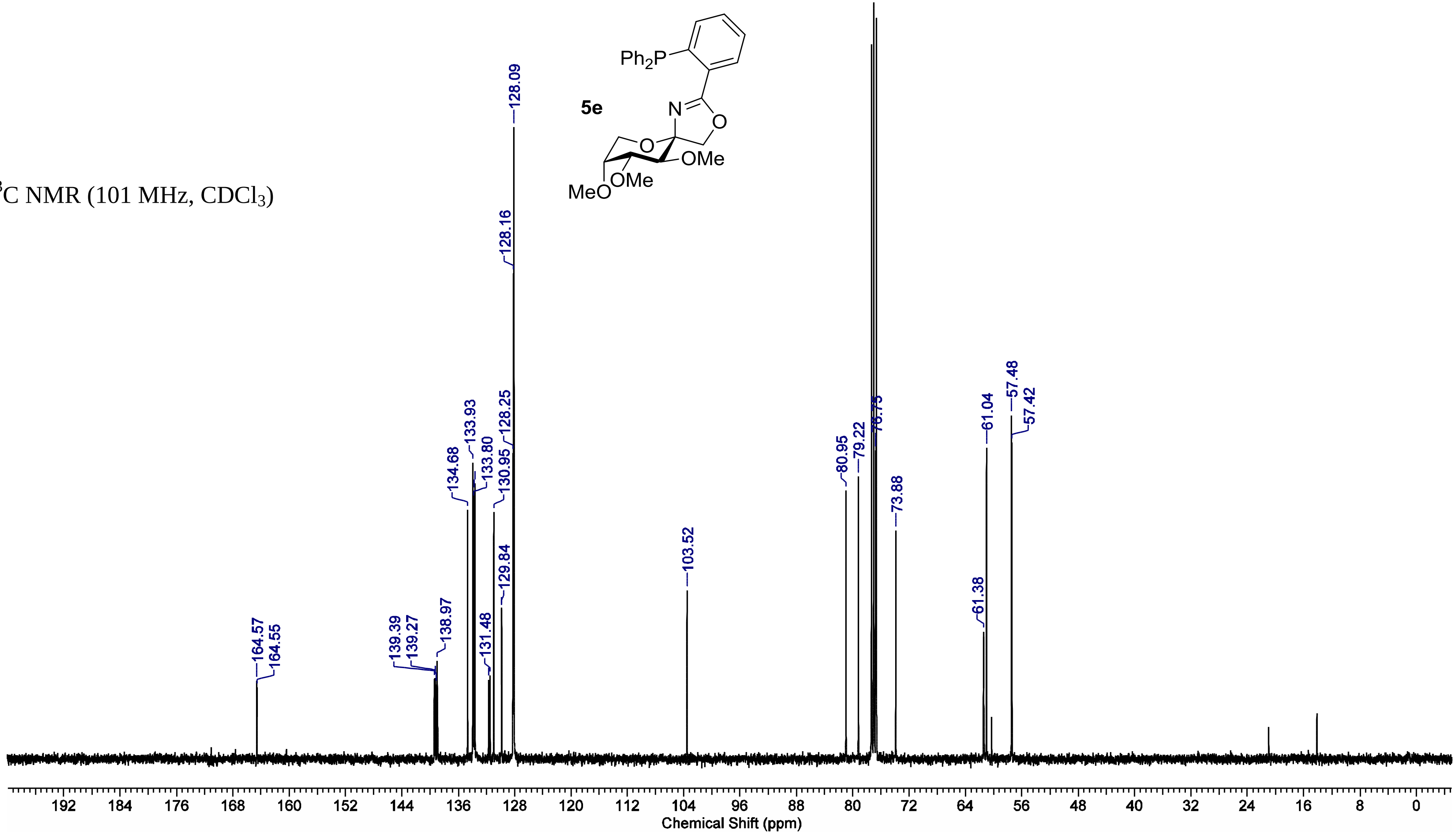

^{31}P NMR (162 MHz, $CDCl_3$)

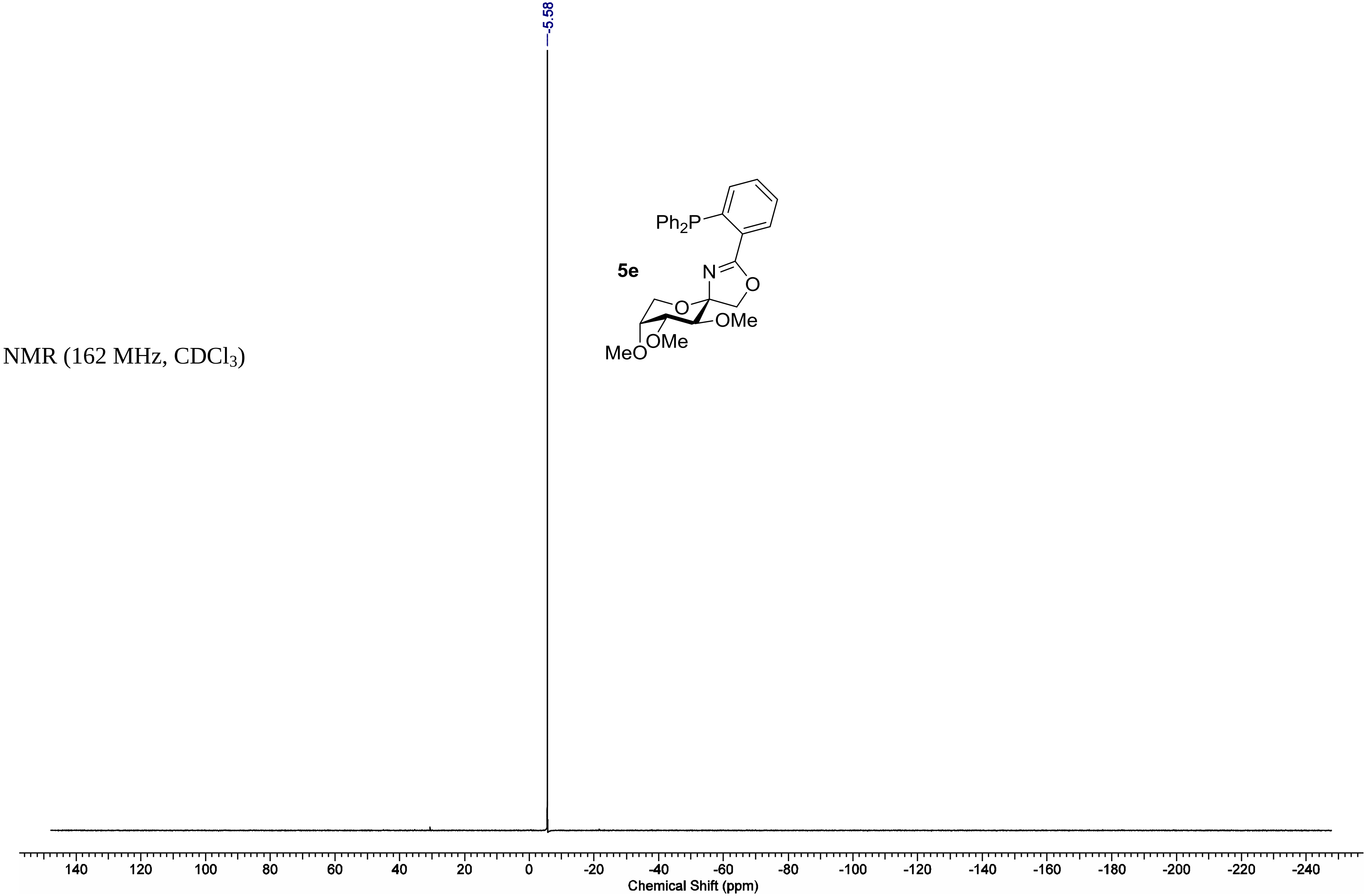

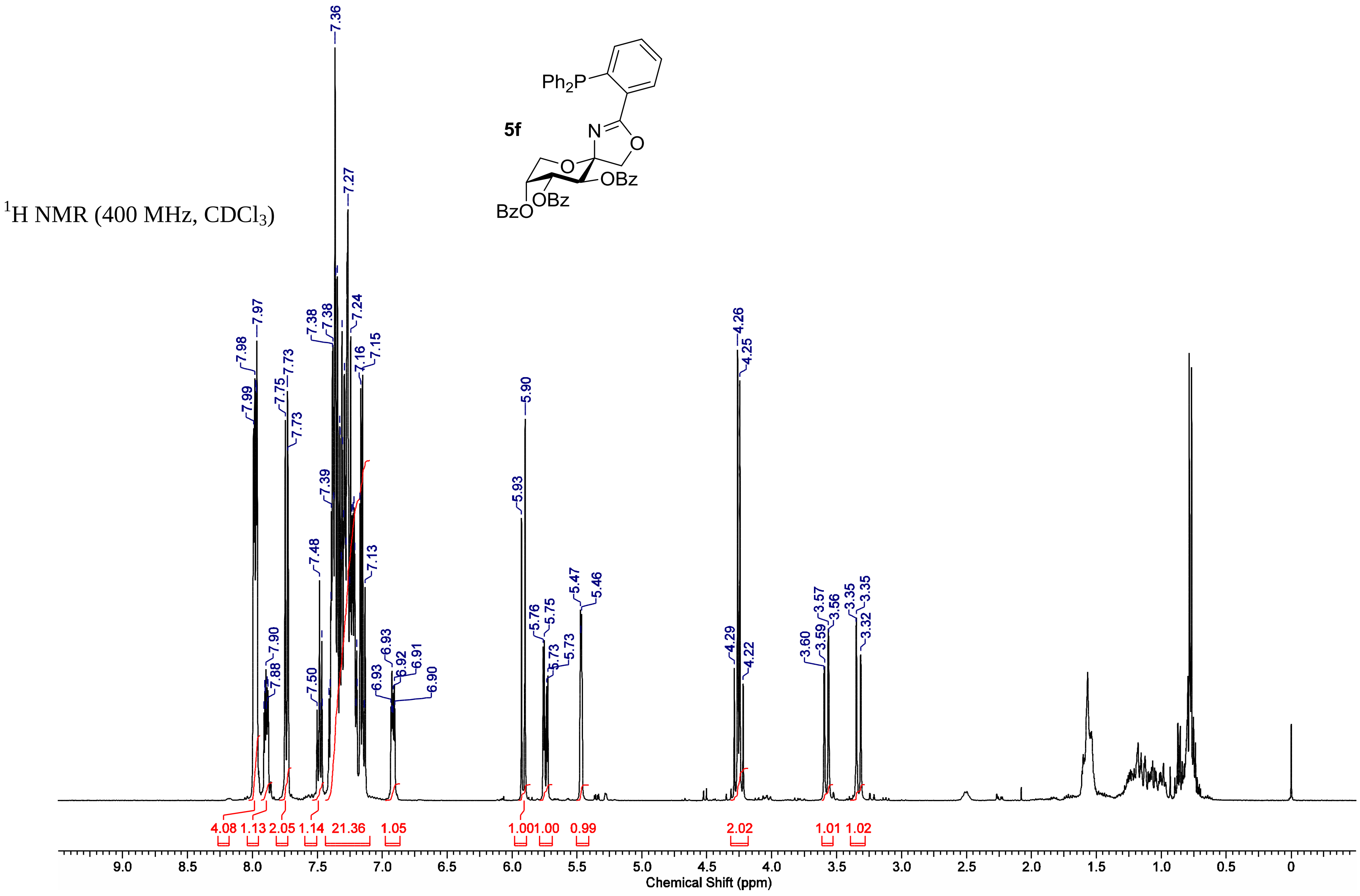

^{1}H NMR (400 MHz, CDCl$_3$)
5f
Ph$_2$P
N
O
O
OBz
OBz
BzO
Chemical Shift (ppm)

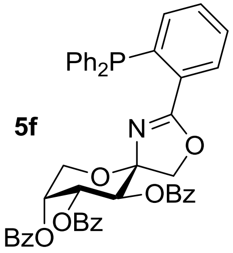

^{13}C NMR (101 MHz, $CDCl_3$)

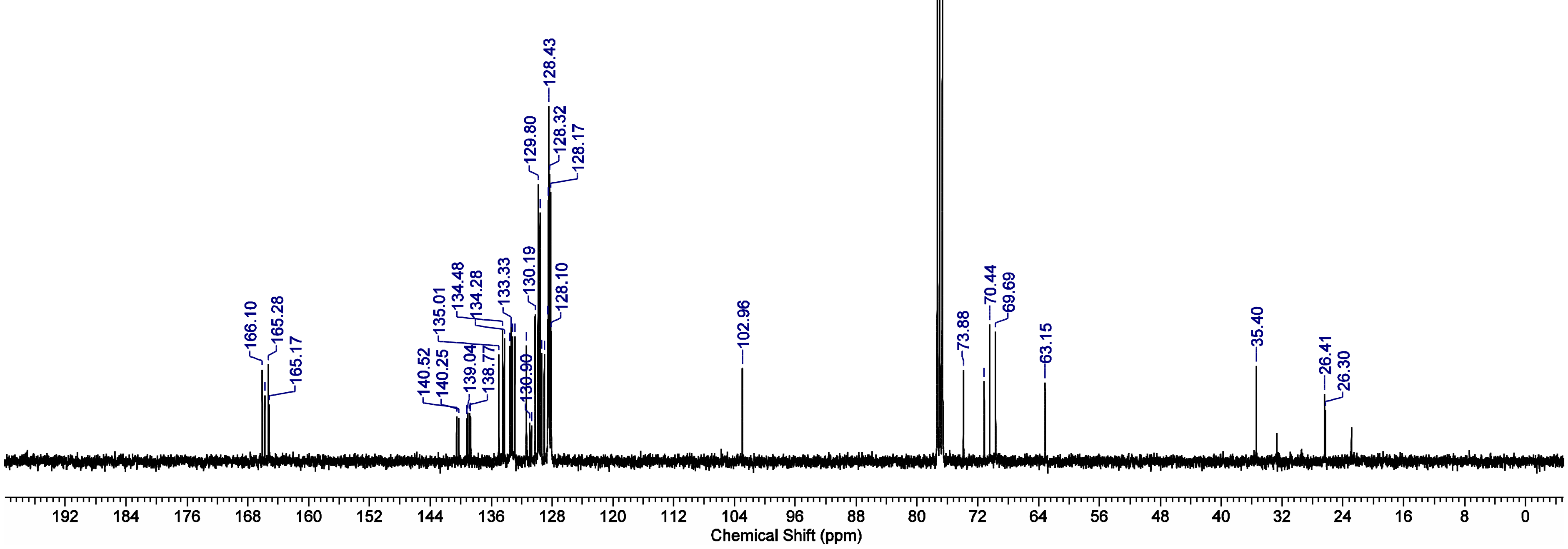

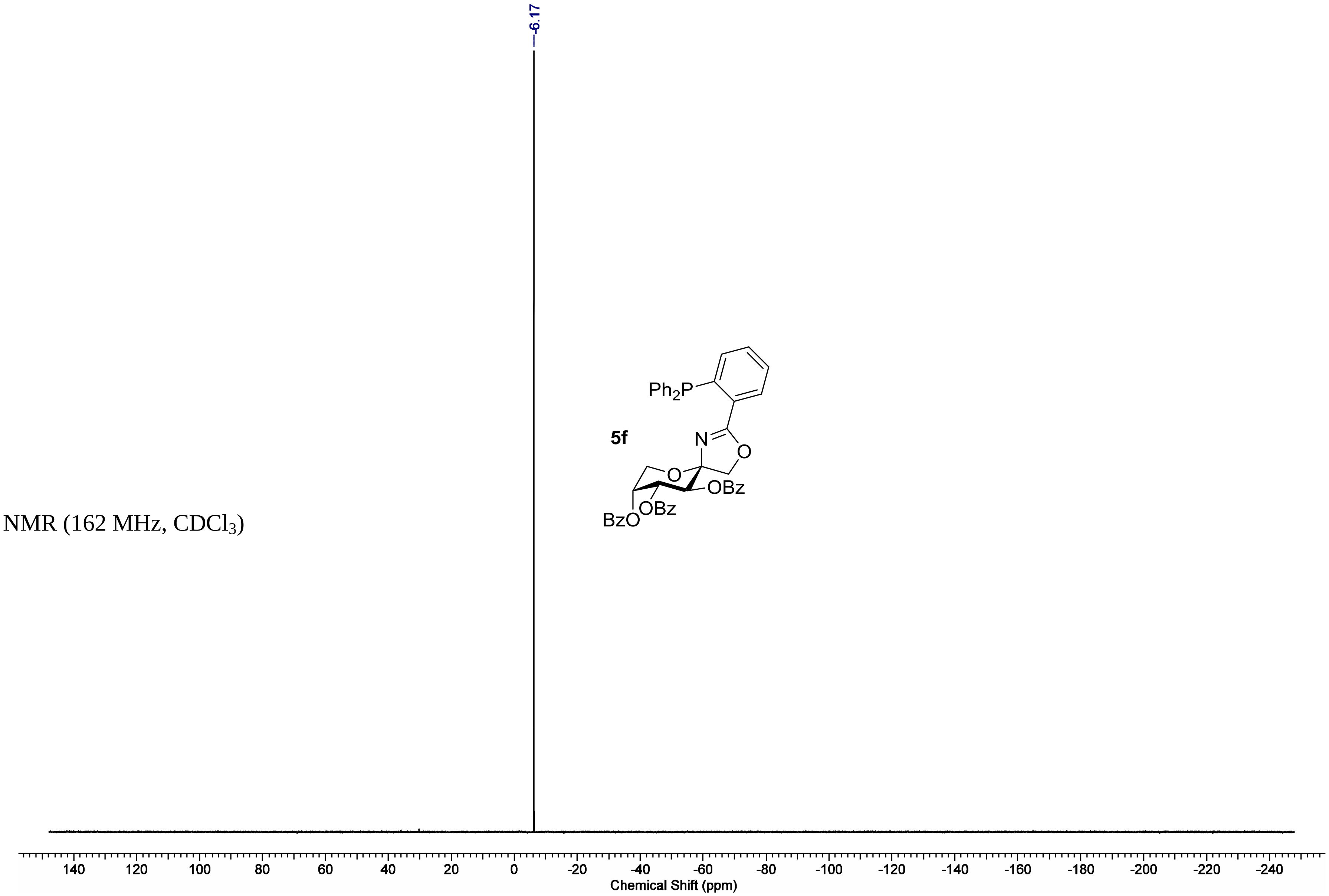

^{31}P NMR (162 MHz, $CDCl_3$)

^{1}H NMR (400 MHz, $CDCl_3$)

5g

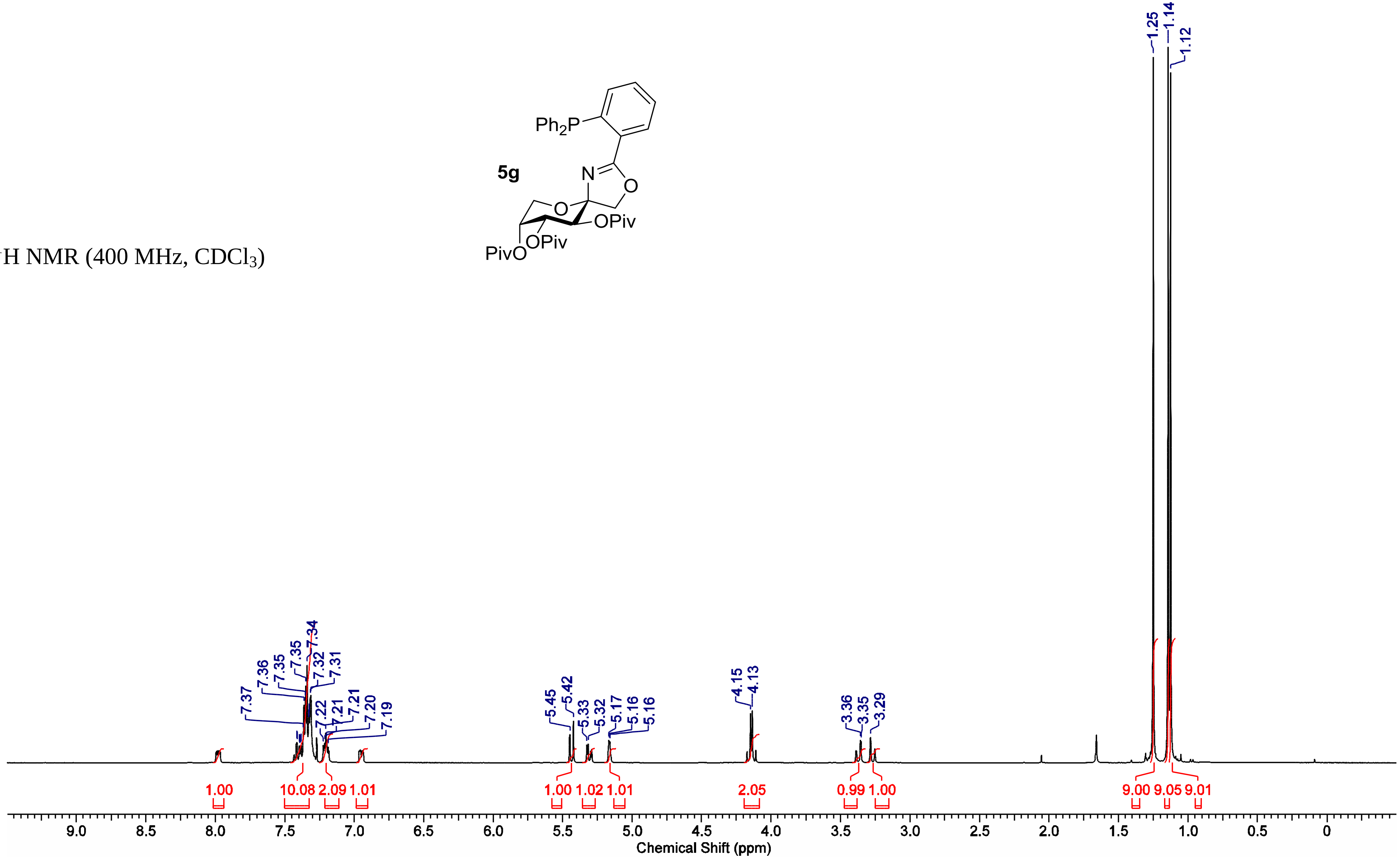

^{13}C NMR (101 MHz, CDCl$_3$)

5g

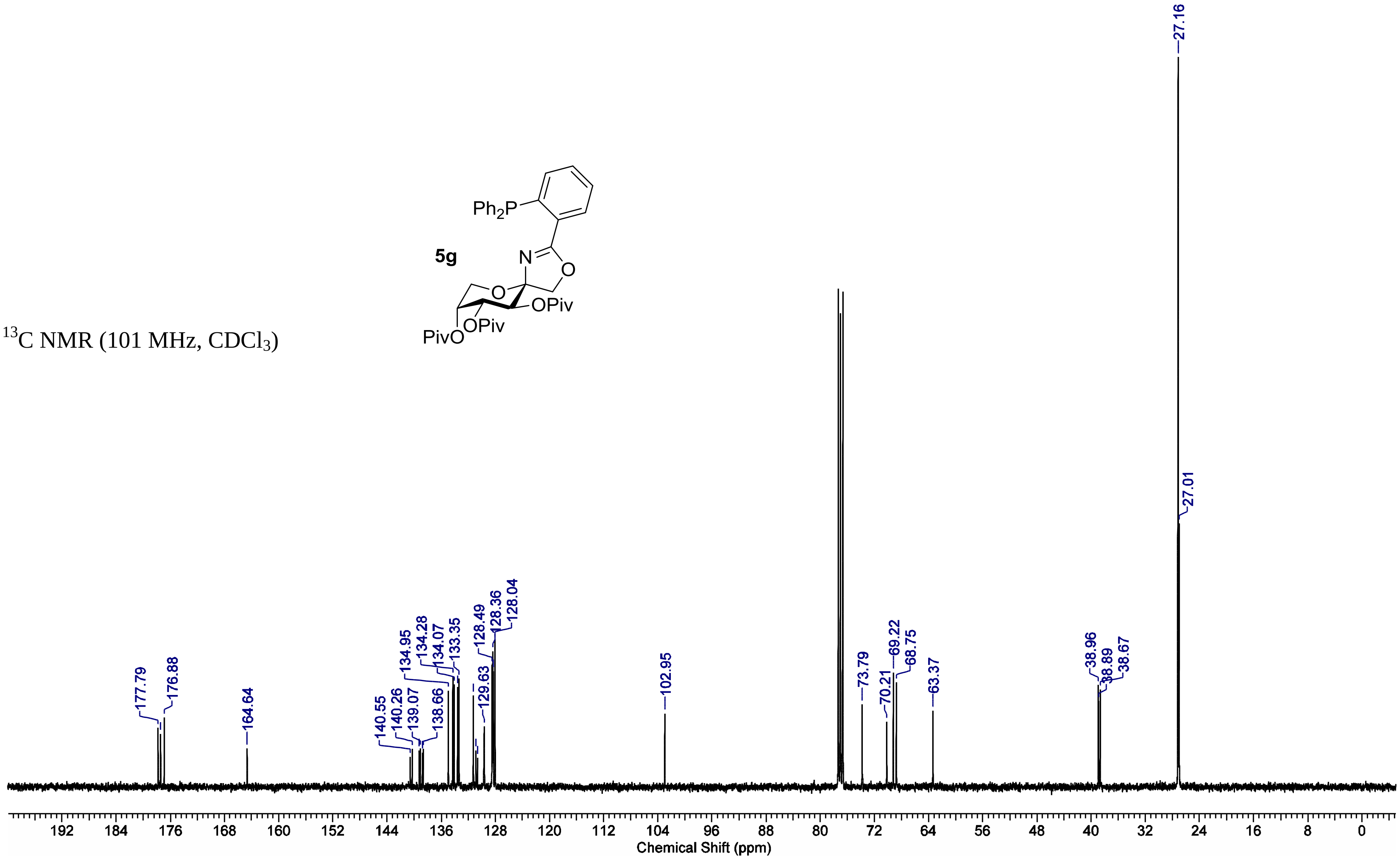

^{31}P NMR (162 MHz, $CDCl_3$)

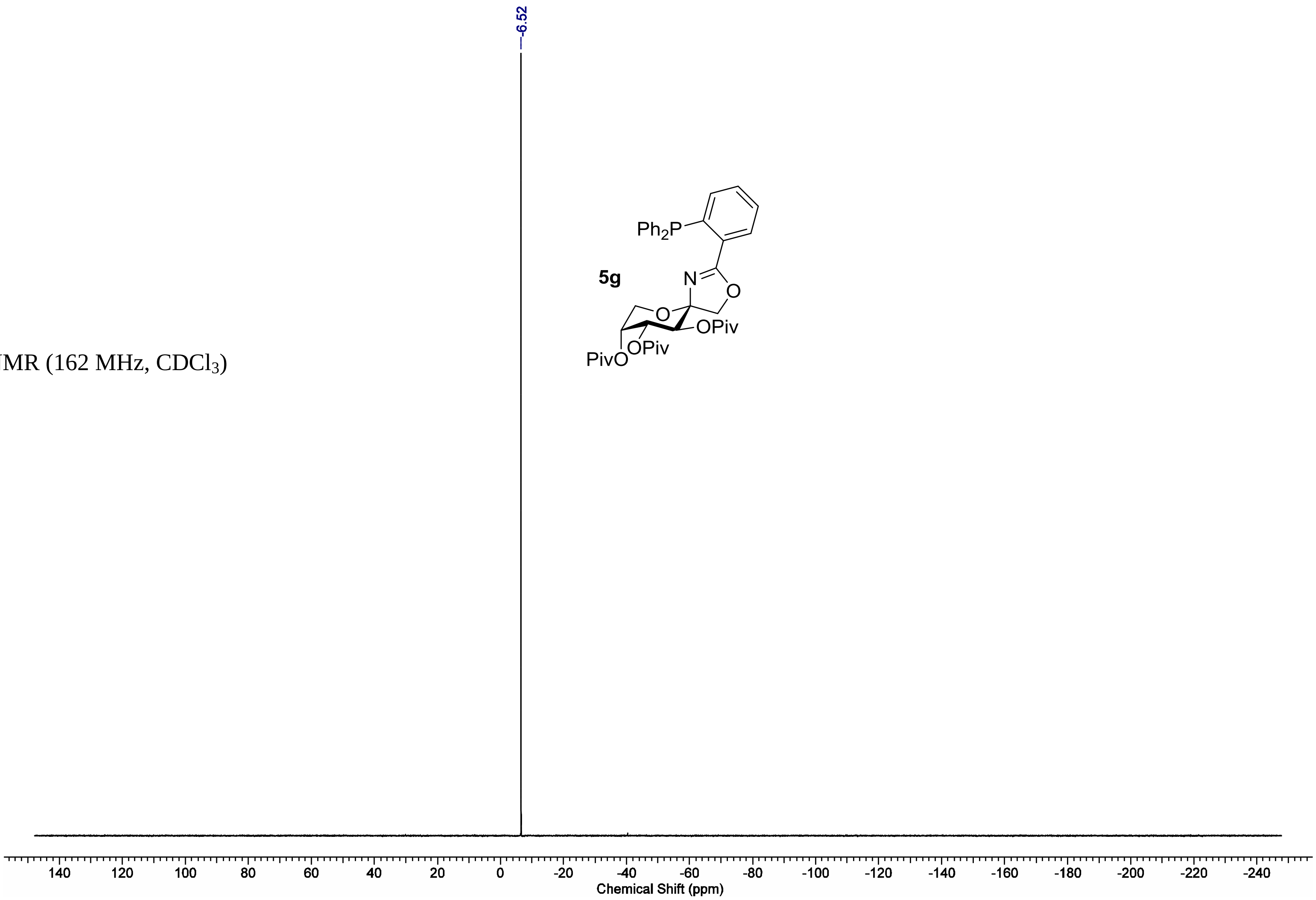

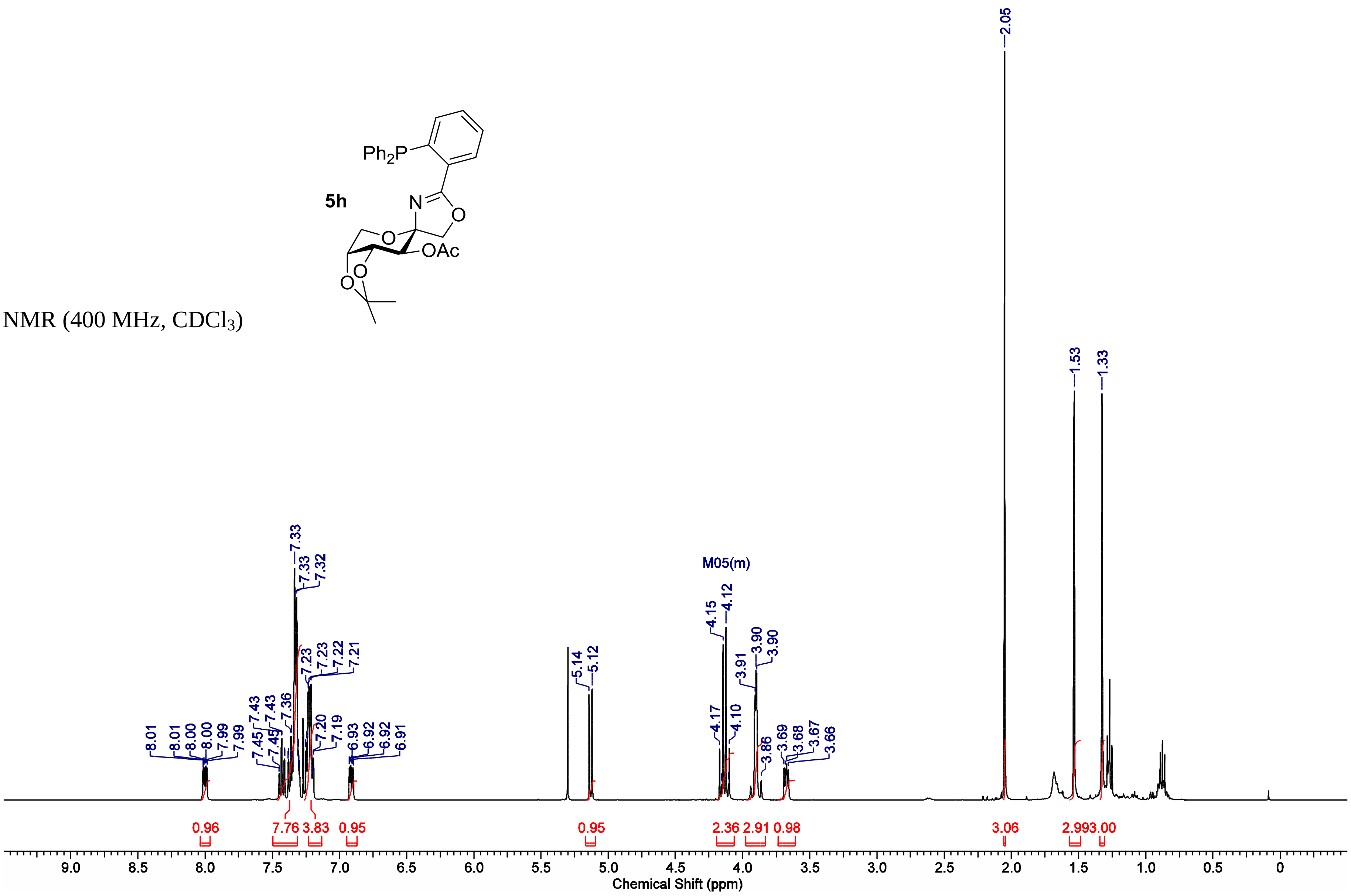

^{1}H NMR (400 MHz, $CDCl_3$)

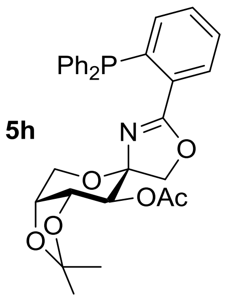

^{13}C NMR (101 MHz, $CDCl_3$)

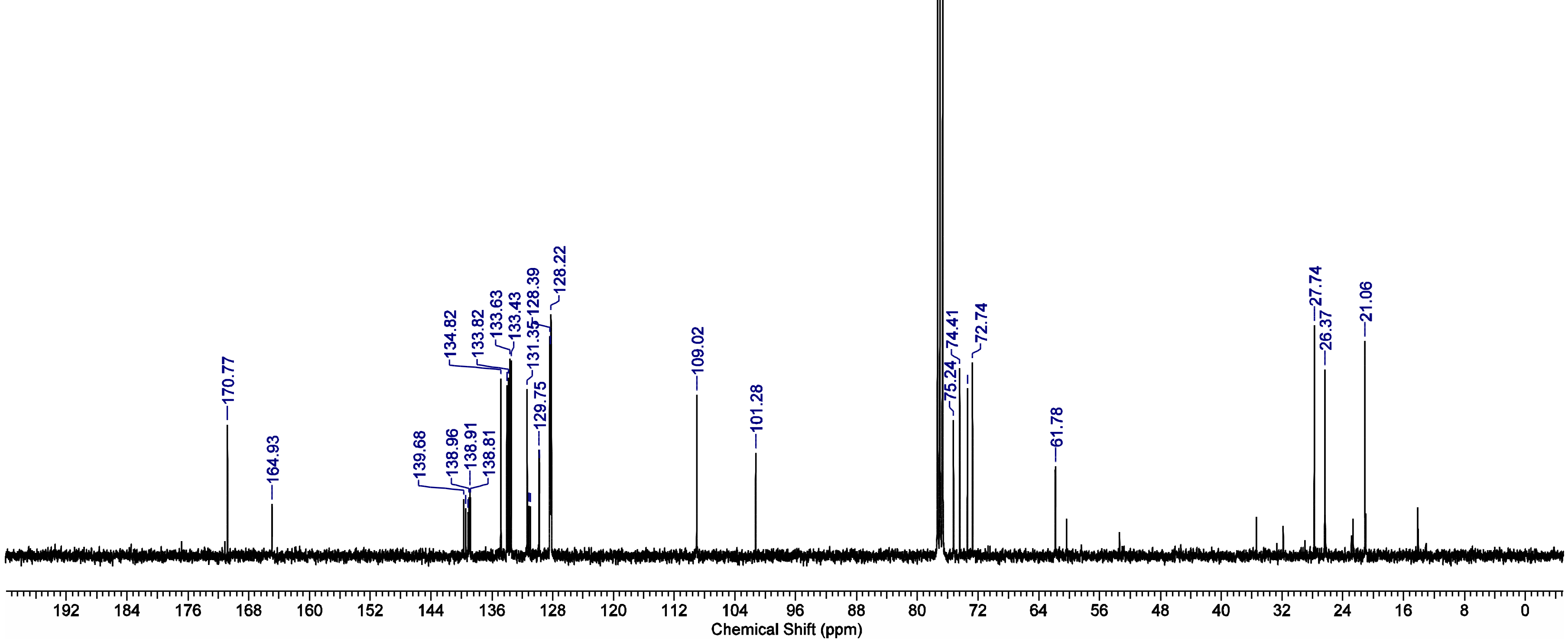

^{31}P NMR (162 MHz, $CDCl_3$)

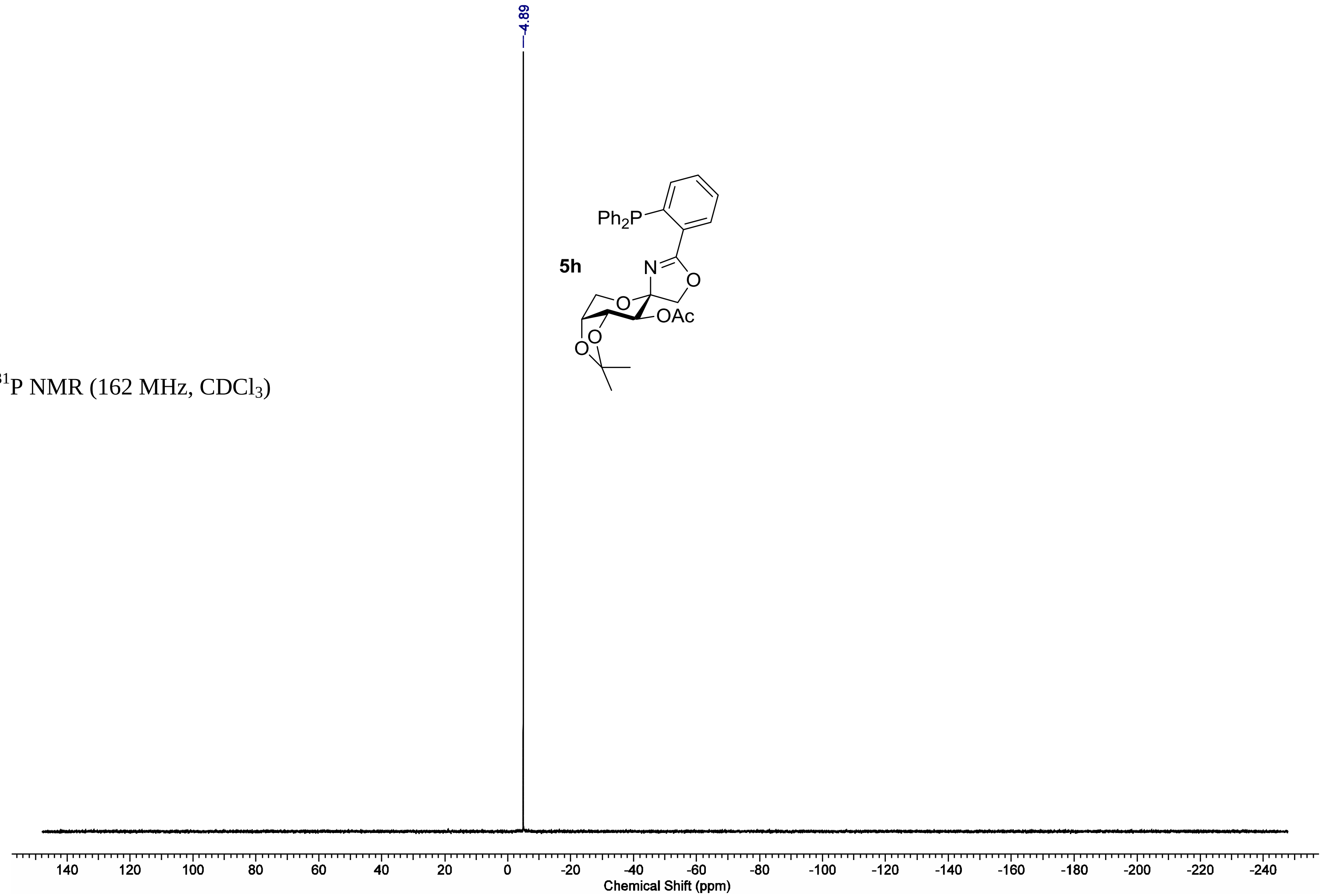

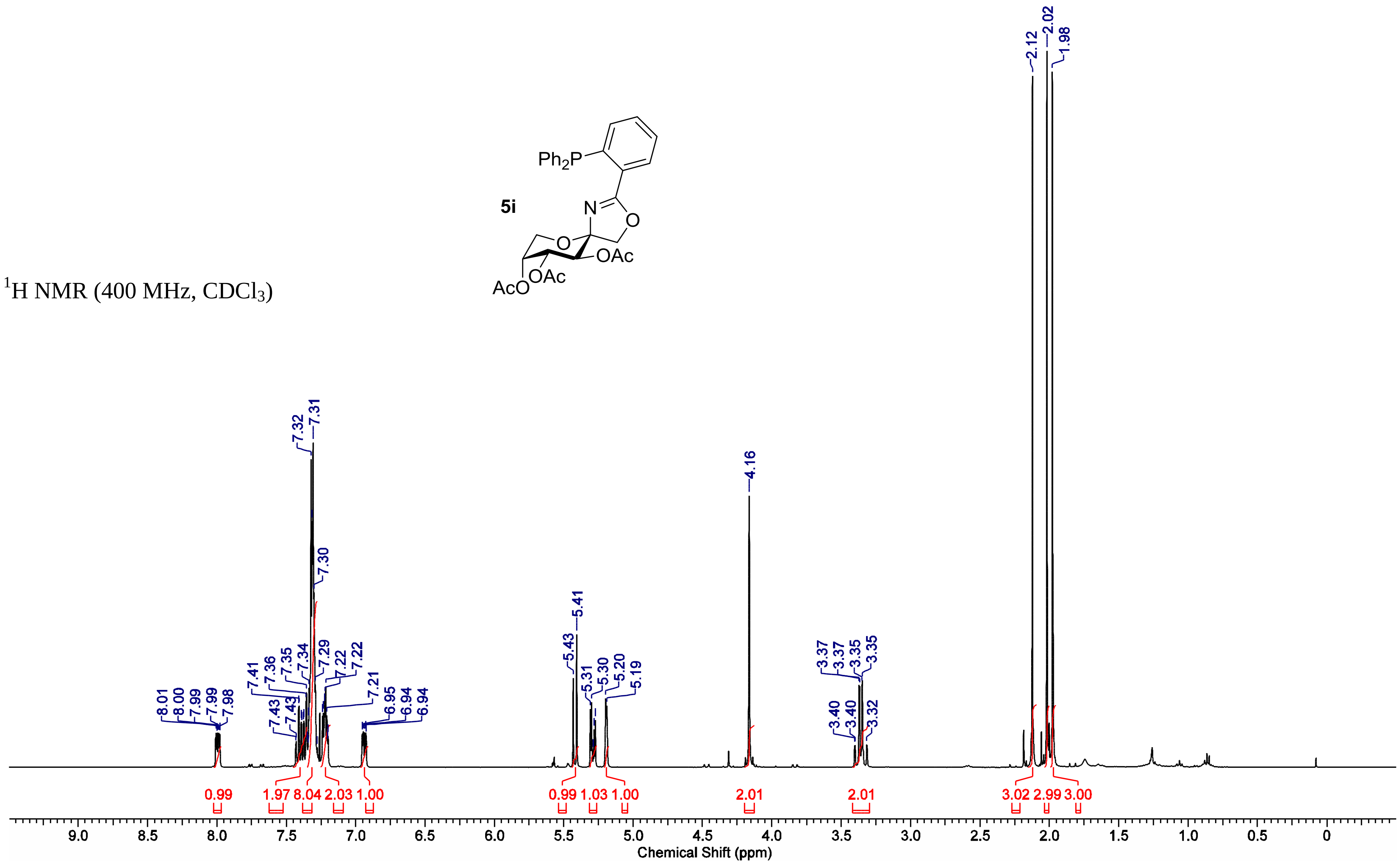

^{1}H NMR (400 MHz, CDCl$_3$)
5i
Ph$_2$P
N
O
OAc
AcO
OAc
2.12
2.02
1.98
7.32
7.31
7.30
4.16
5.41
5.43
5.31
5.30
5.20
5.19
3.37
3.37
3.35
3.35
3.40
3.40
3.32
8.01
8.00
7.99
7.99
7.98
7.41
7.36
7.35
7.34
7.43
7.43
7.29
7.22
7.22
7.21
6.95
6.94
6.94
0.99
1.97
8.04
2.03
1.00
0.99
1.03
1.00
2.01
2.01
3.02
2.99
3.00
9.0
8.5
8.0
7.5
7.0
6.5
6.0
5.5
5.0
4.5
4.0
3.5
3.0
2.5
2.0
1.5
1.0
0.5
0
Chemical Shift (ppm)

^{13}C NMR (101 MHz, $CDCl_3$)

5i

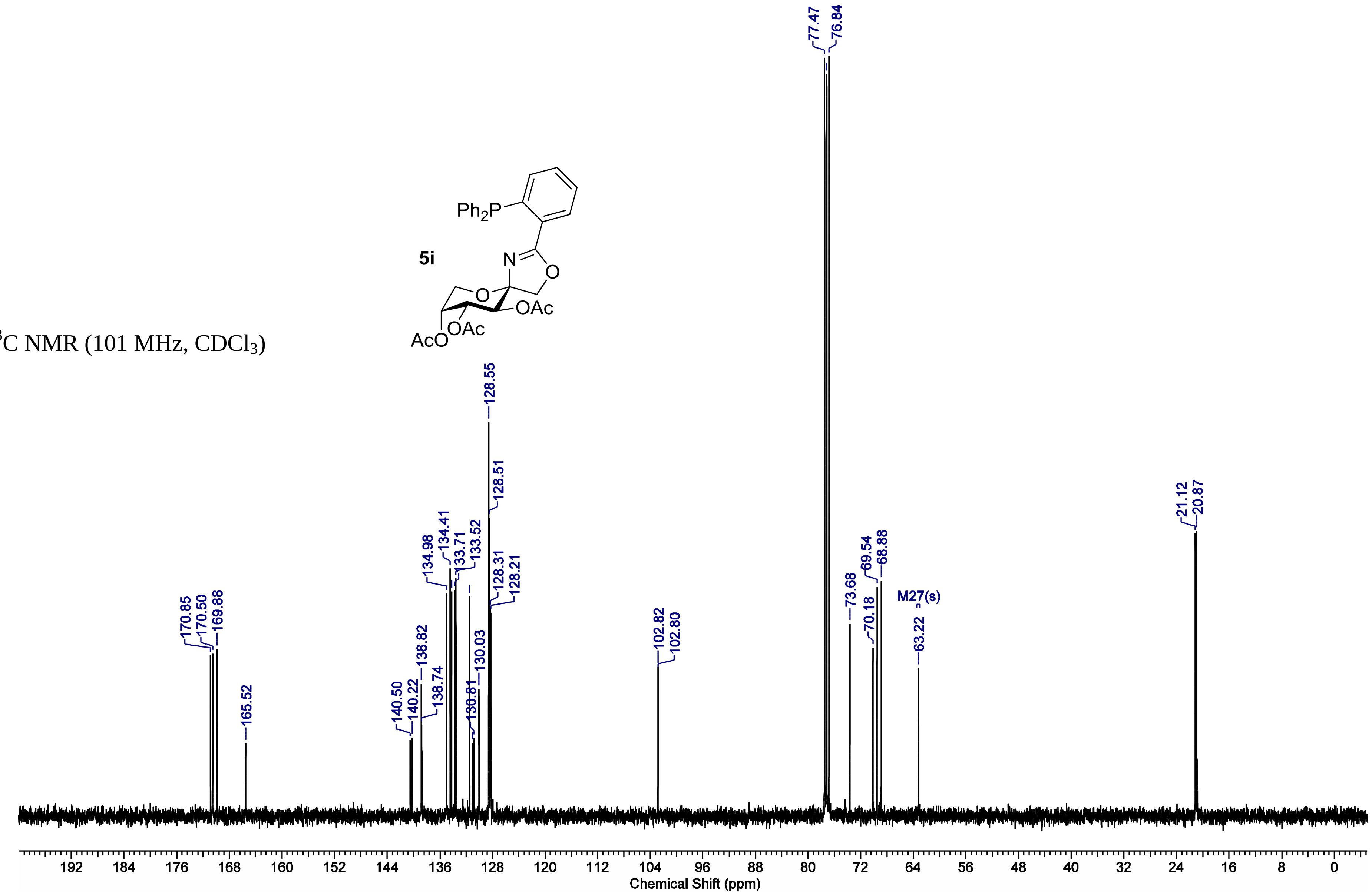

^{31}P NMR (162 MHz, $CDCl_3$)

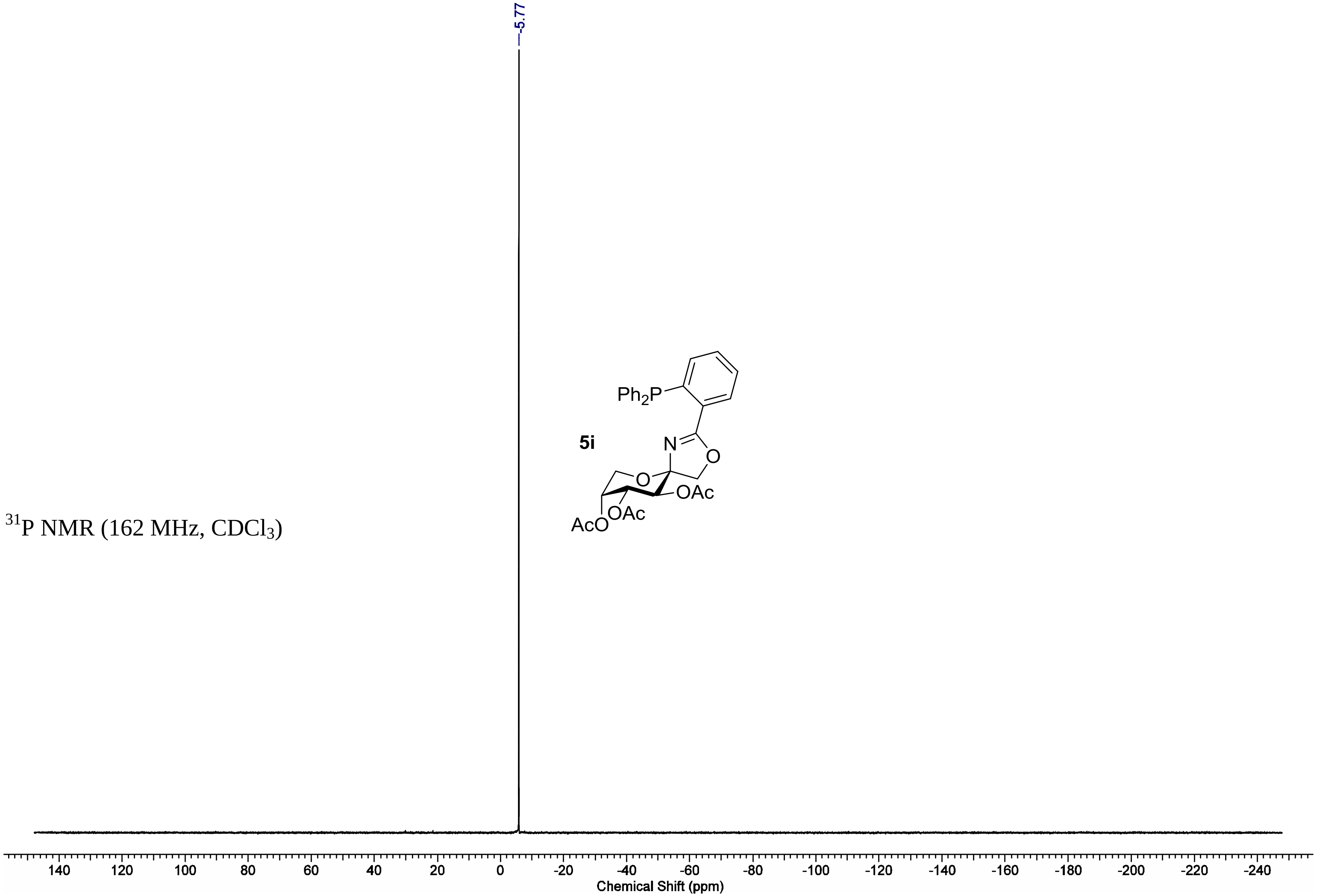

^{1}H NMR (400 MHz, $CDCl_3$)

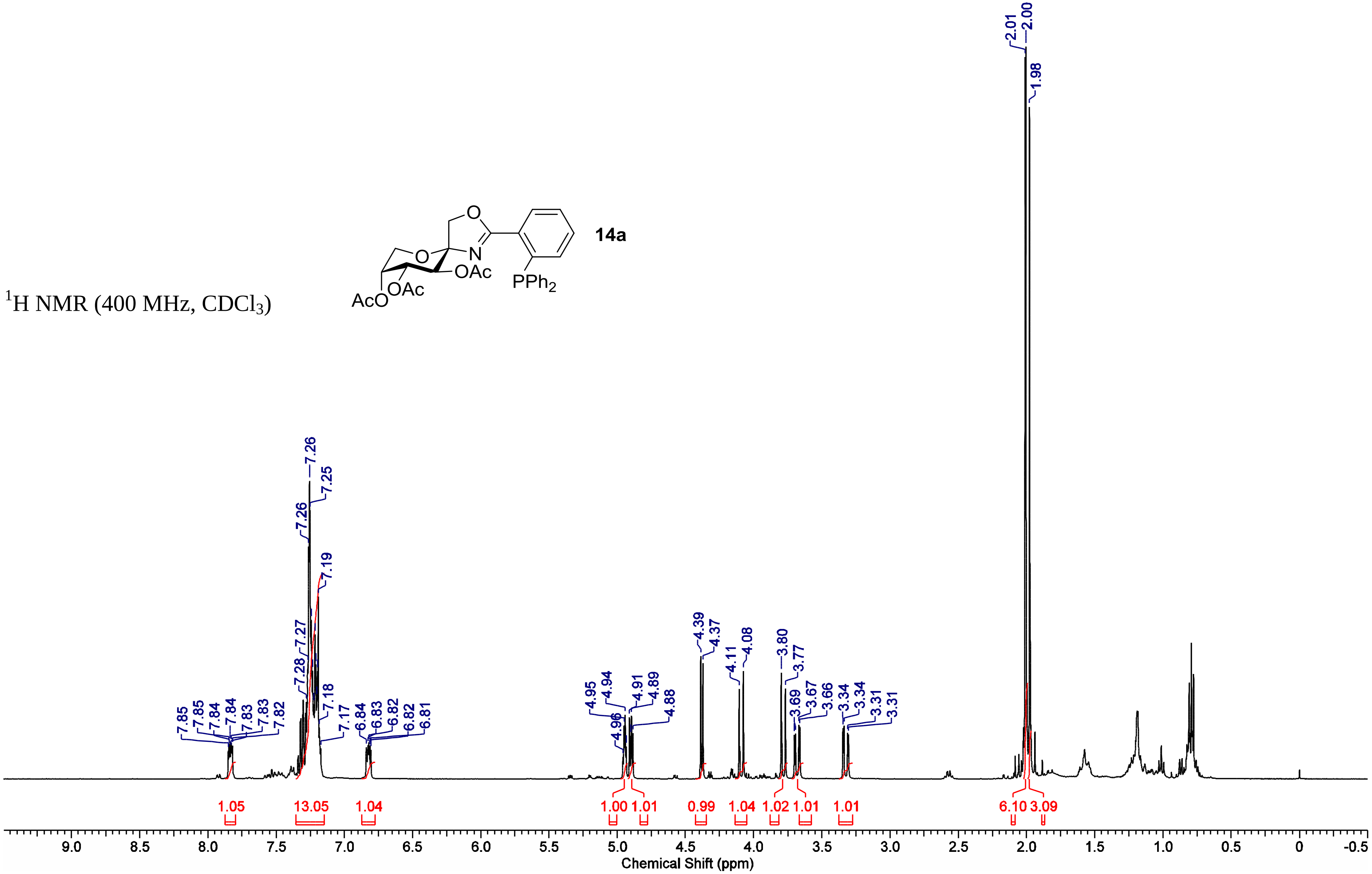

^{13}C NMR (101 MHz, $CDCl_3$)

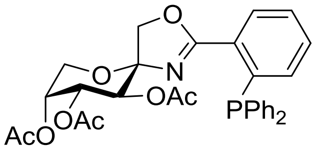

14a

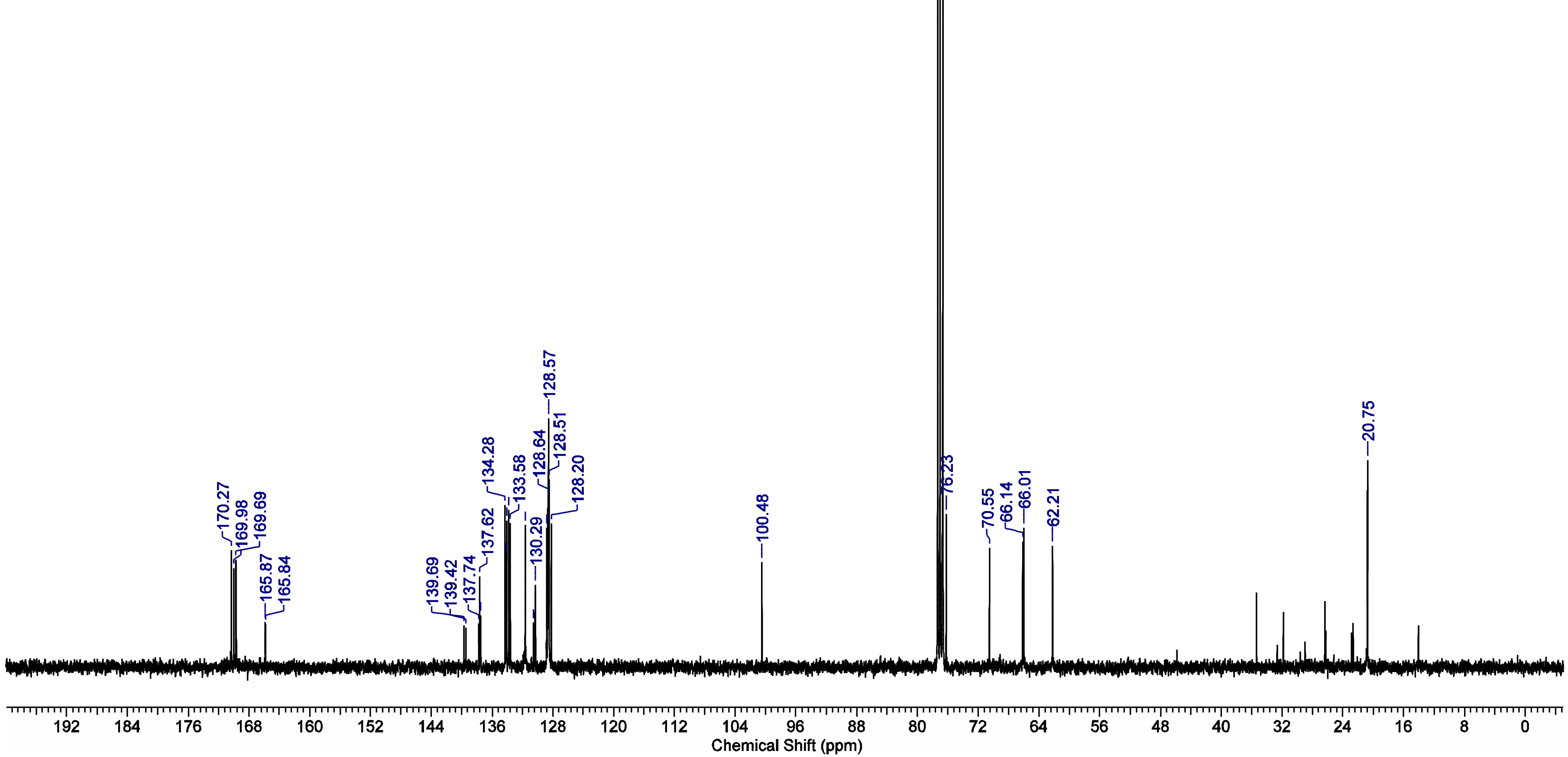

^{31}P NMR (162 MHz, $CDCl_3$)

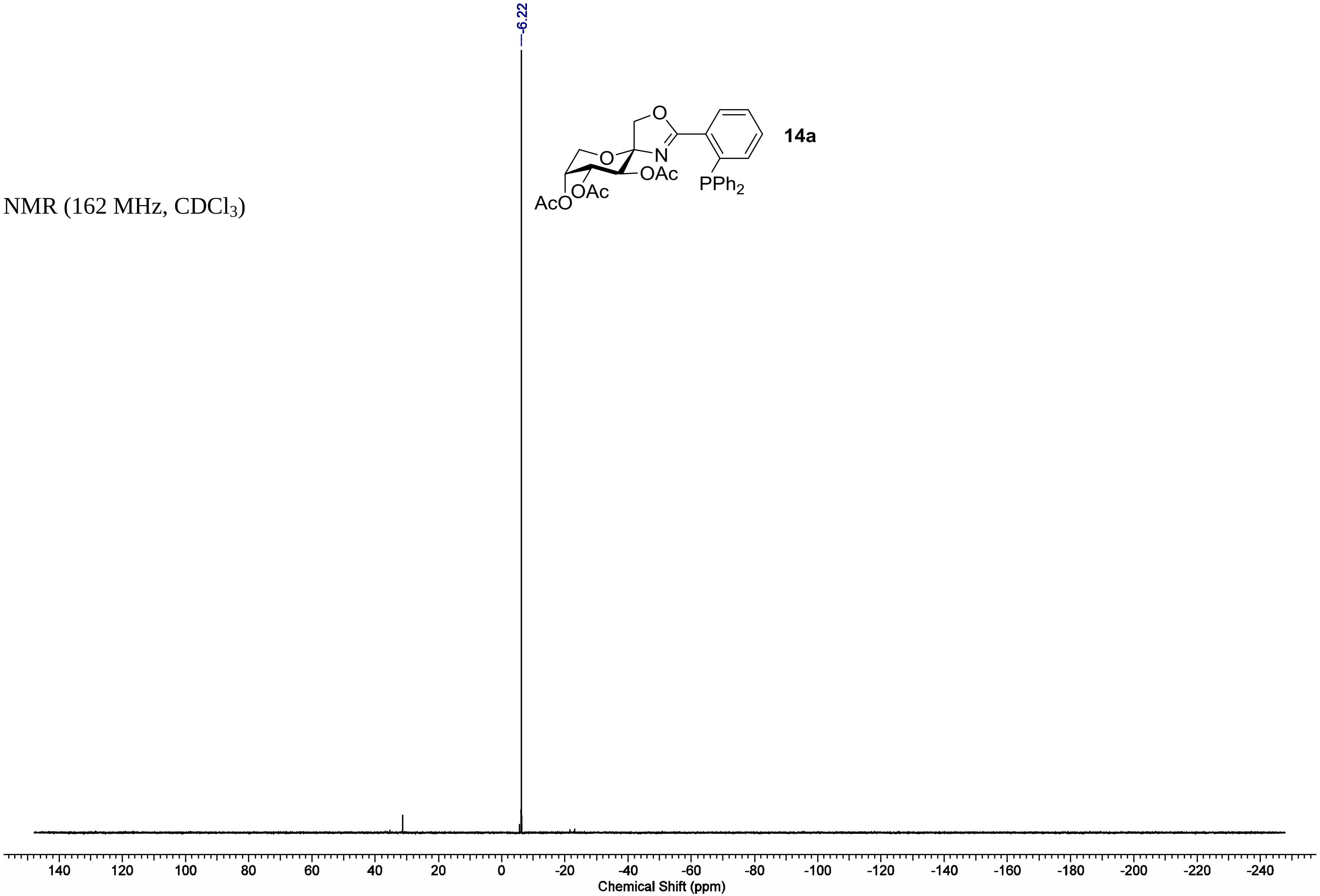

6 HPLC chromatograms

Table 3

entry 1

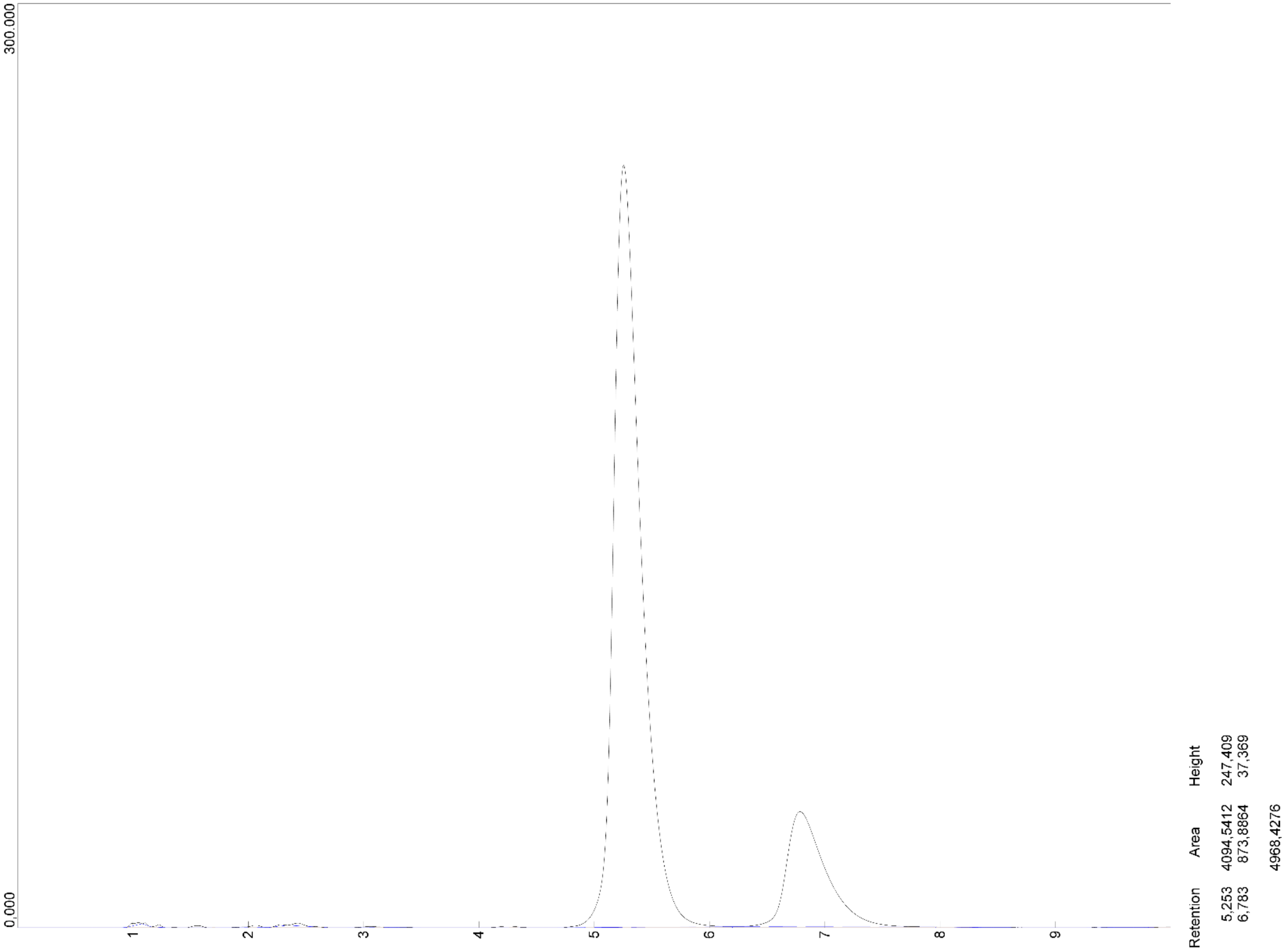

S113

Table 3

entry 2

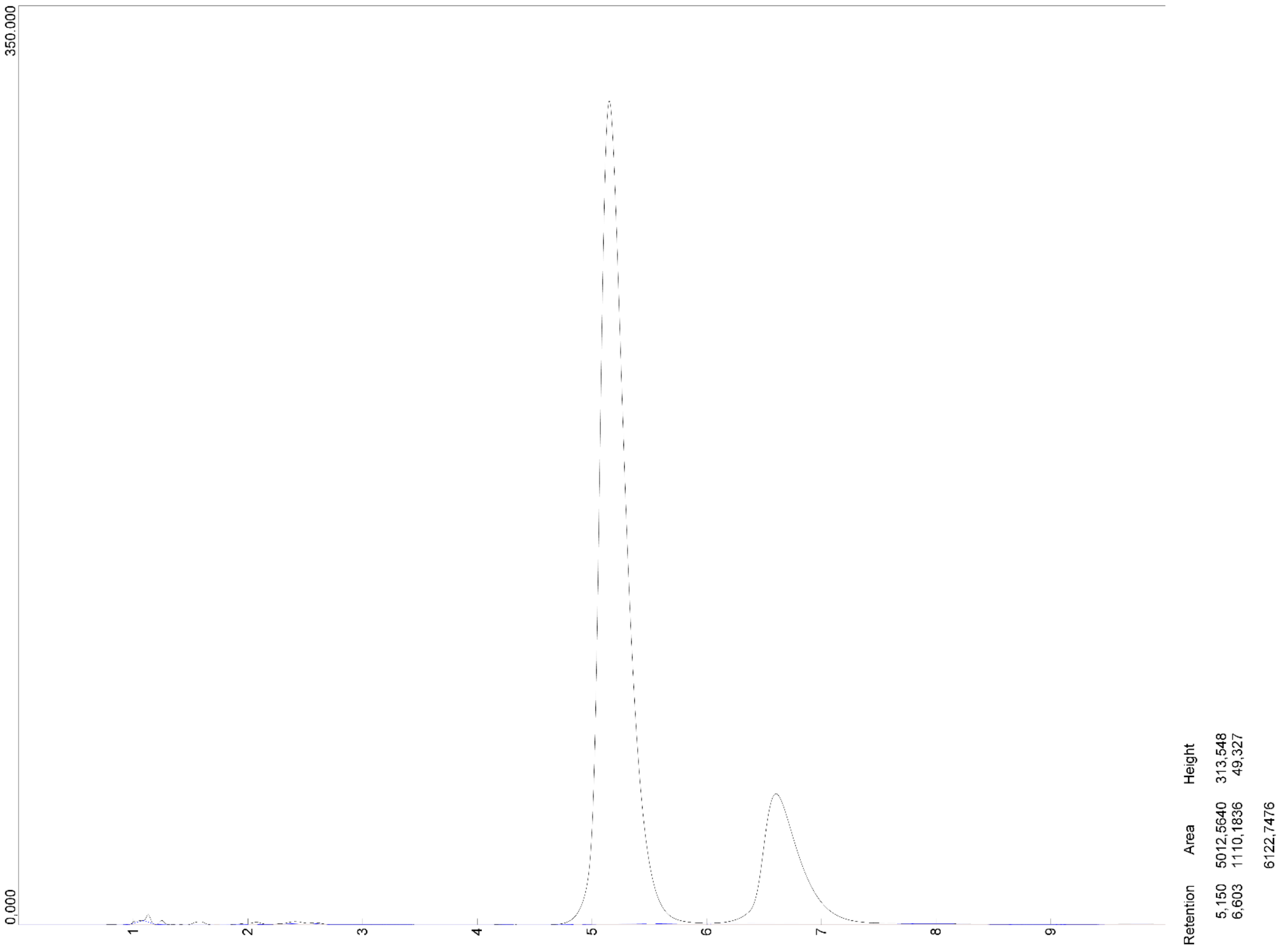

Table 3

entry 3

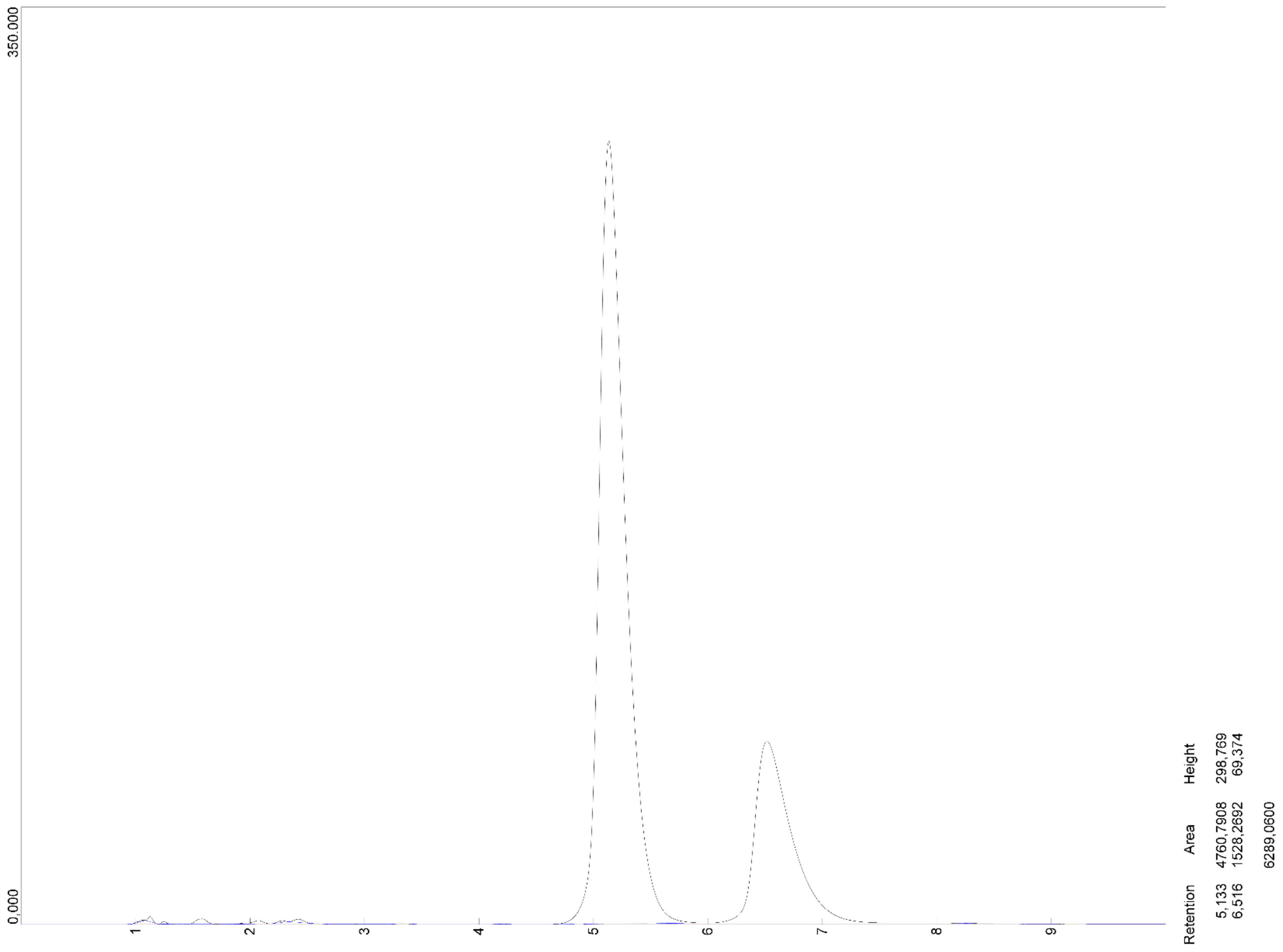

Table 3

entry 4

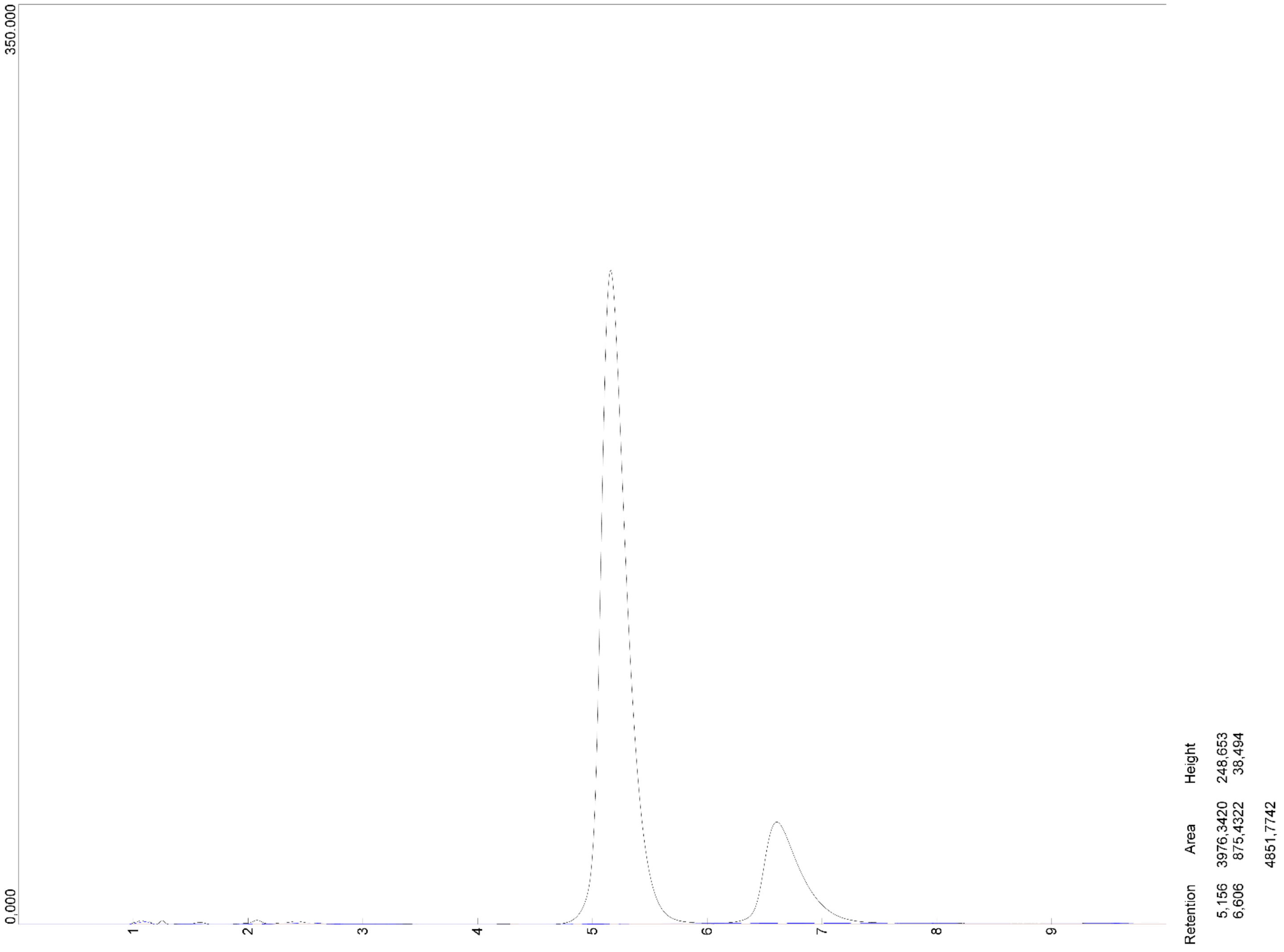

Table 3

entry 5

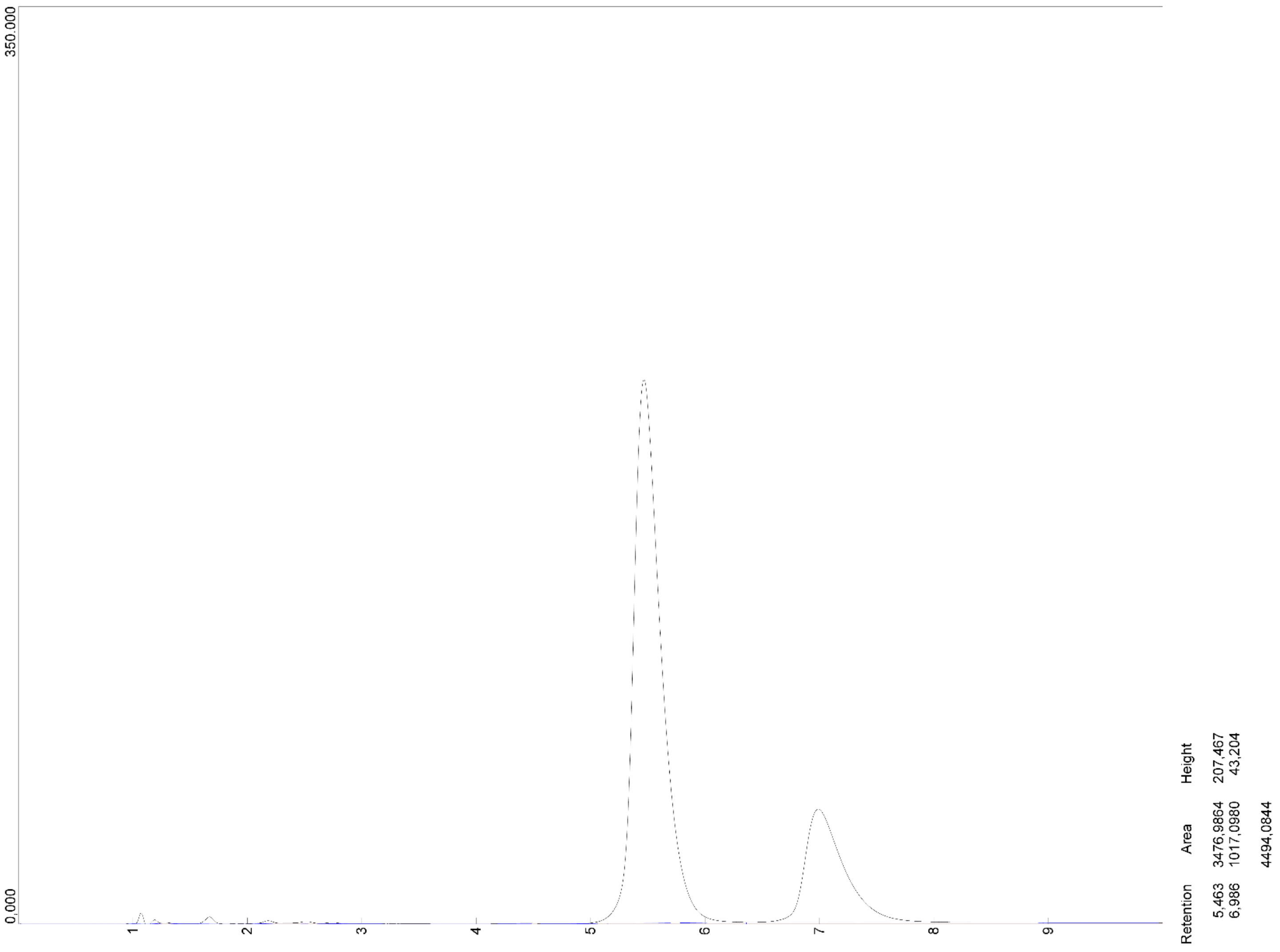

Table 3

entry 6

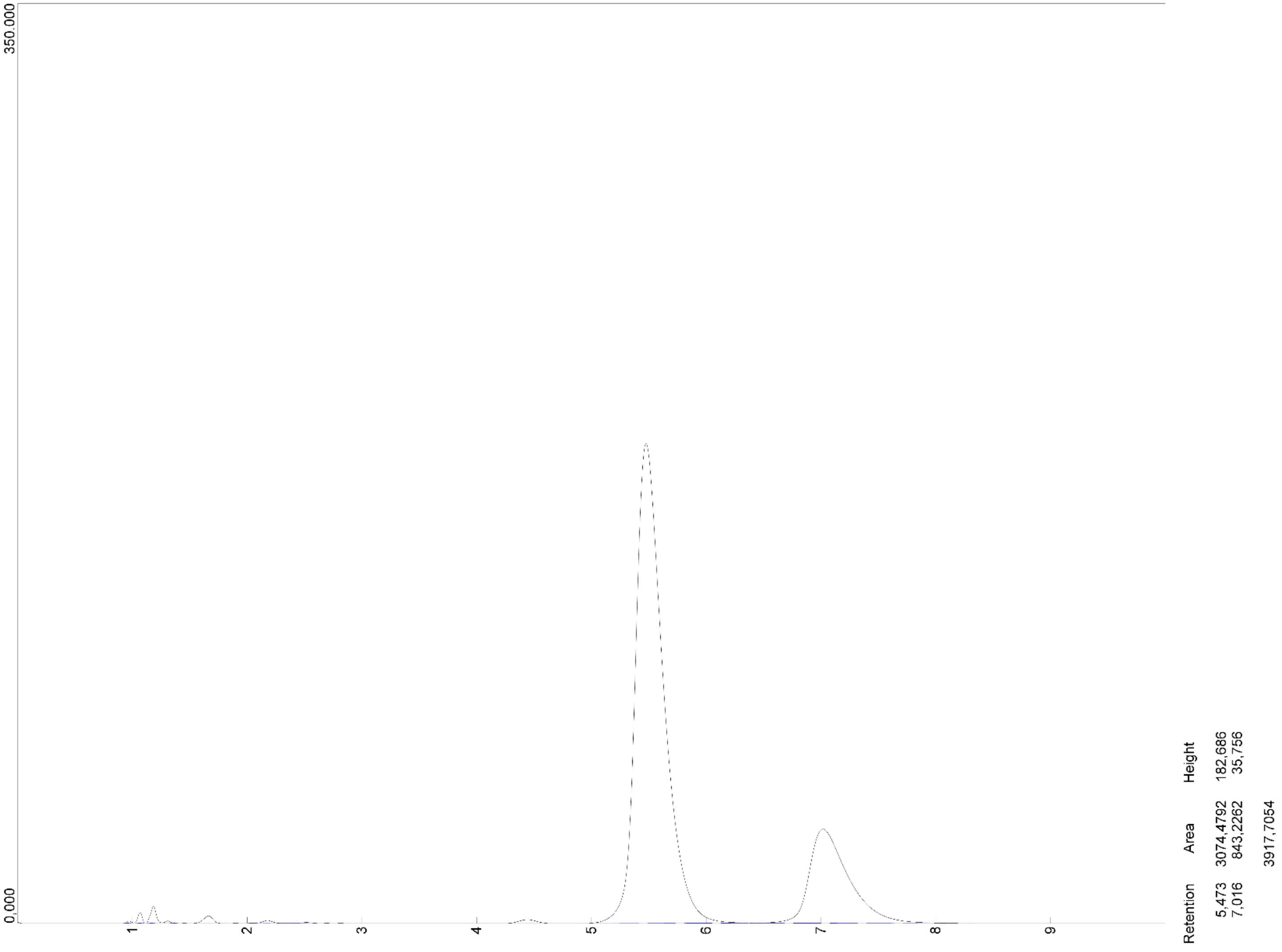

Table 3

entry 7

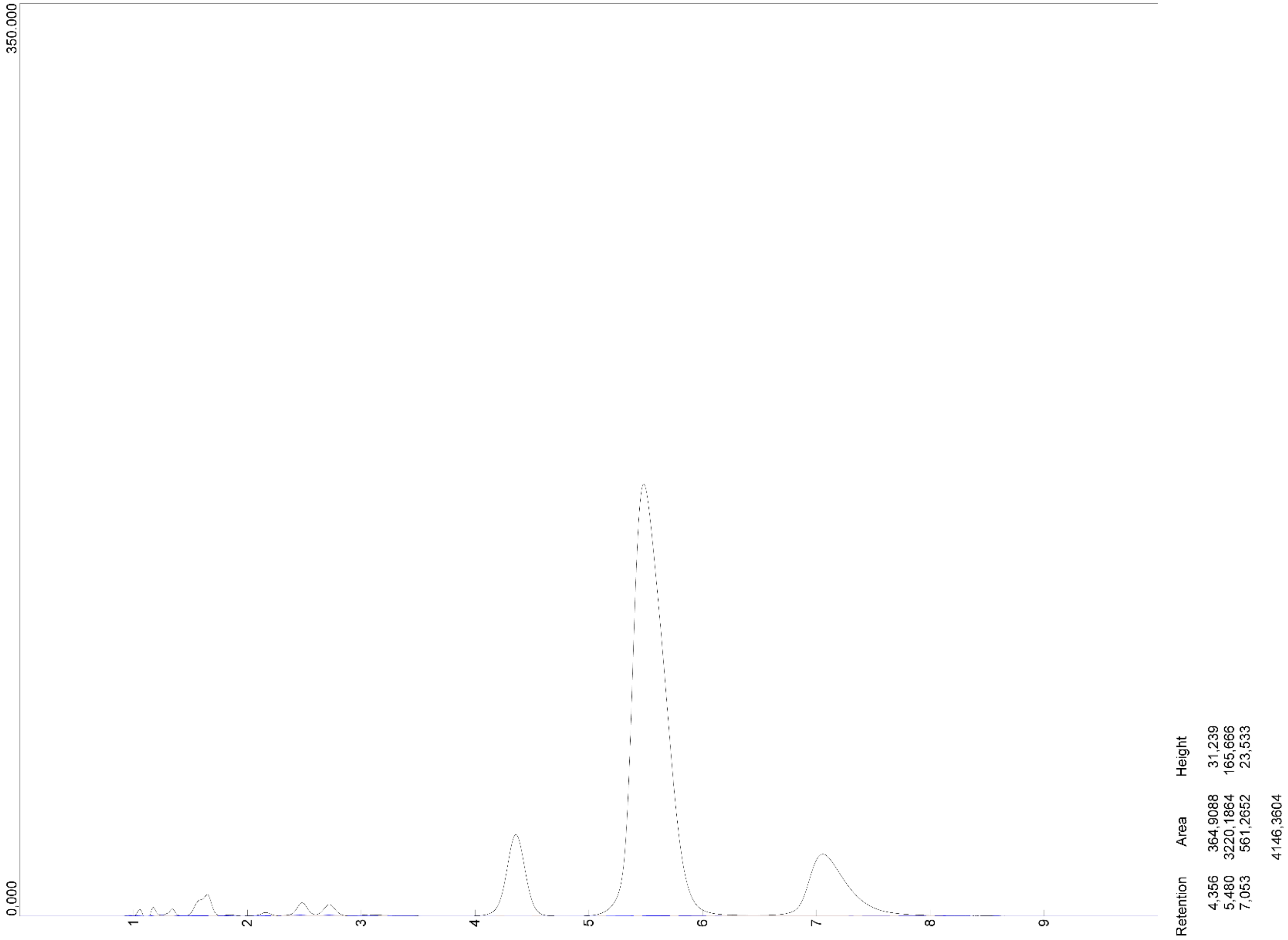

Conversion was at 83%, rac - diphenylallyl acetate shows peaks with t_R = 4.36 min and t_R = 5.38 min. For the calculation of the *er* of **16** the area of the peak at t_R = 5.48 min was corrected by 364.9 units.

Table 3

entry 8

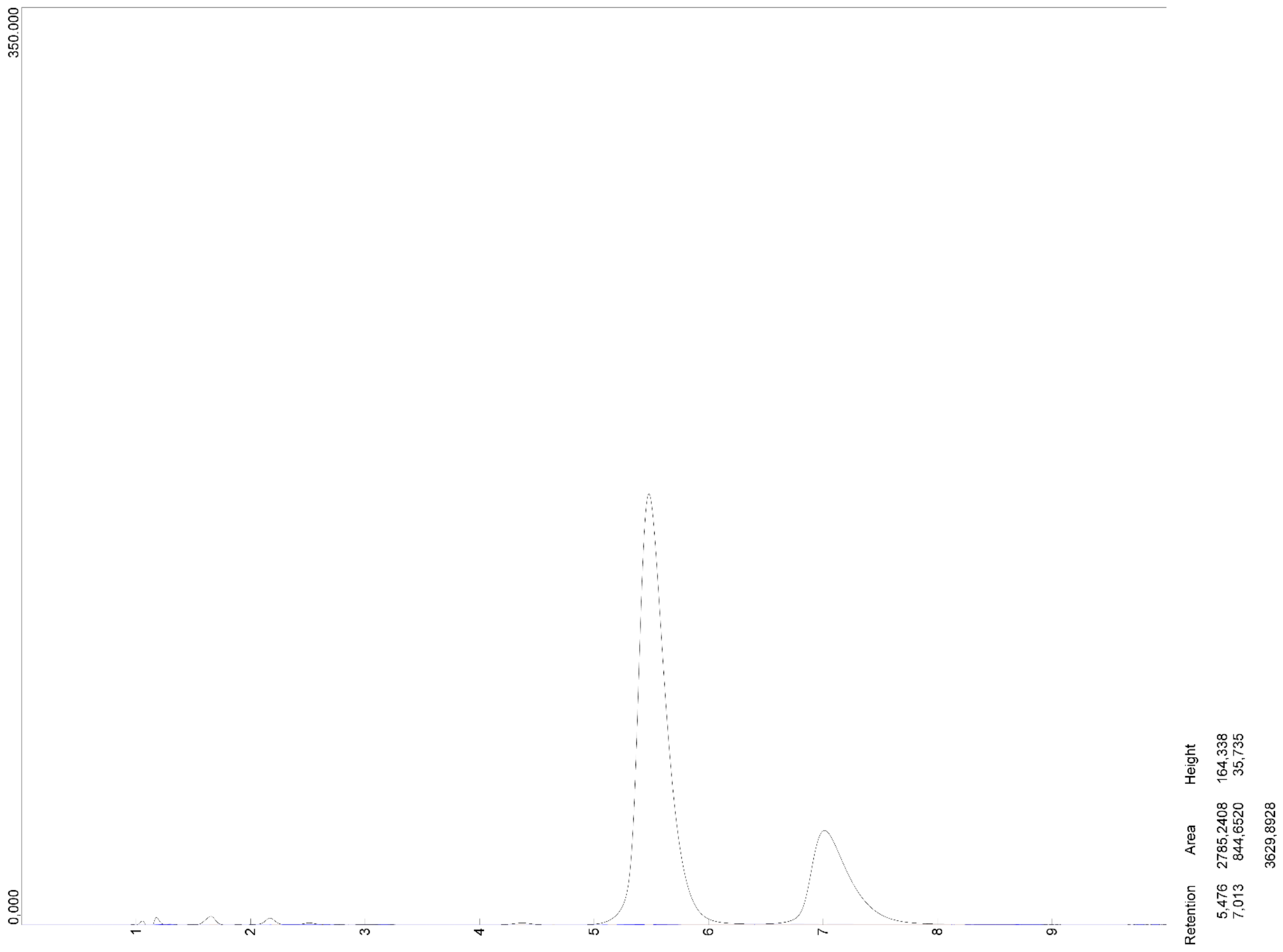

7 References

1. Brady Jr, R. F. *Carbohydr. Res.* **1970**, *15*, 35-40.

2. Veleti, S. K.; Lindenberger, J. J.; Thanna, S.; Ronning, D. R.; Sucheck, S. J. *J. Org. Chem.* **2014**, *79*, 9444-9450.

3. Kaji, E.; Kurimoto, E.; Saiga, R.; Matsuurra, A.; Harada, K.; Nishino, T. *Heterocycles* **2005**, *66*, 453-468.

4. Kuhn, R. K., Gerd; Seelinger, A. *Liebigs Ann. Chem.* **1958**, *618*, 82 -89.

5. Klemer, A.; Balkau, D. *Chem. Ber.* **1978**, *111*, 1514-1520.

6. Campbell, M. M.; Heffernan, G. D.; Lewis, T. *Carbohydr. Res.* **1994**, *251*, 243-250.

7. Zhang, P.; Ling, C.-C. *Carbohydr. Res.* **2017**, *445*, 7-13.

8. Kumar, P. S.; Kumar, G. D. K.; Baskaran, S. *Eur. J. Org. Chem.* **2008**, 6063-6067.

9. Kang, J.; Lim, G. J.; Yoon, S. K.; Kim, M. Y. *J. Org. Chem.* **1995**, *60*, 564-577.

10. *COSMO v. 1.61*, Bruker AXS Inc., Madison, WI, **2012**.

11. APEX 3 V. 2016.5-0, Bruker AXS Inc., Madison, WI, **2016**.

12. *SAINT v. 8.37A*, Bruker AXS Inc., Madison, WI, **2015**.

13. *SADABS* Krause, L., Herbst-Irmer, R., Sheldrick, G. M. & Stalke, D. **2015** *J. Appl. Cryst.* 48.

14. *SHELXTL Acta Cryst.* (2015), A71, 3-8, Shelxl *Acta Cryst.* **2015**, C71, 3-8

15. SHELXLE, C. B. Hubschle, G. M. Sheldrick, B. Dittrich, *J. Appl. Crystallogr*. **2011**, *44*, 1281-1284.
